# Nickel(ii)-catalyzed enantioselective cyclopropanation of 3-alkenyl-oxindoles with phenyliodonium ylide *via* free carbene†
†Electronic supplementary information (ESI) available. CCDC 1038443 and 1038445. For ESI and crystallographic data in CIF or other electronic format see DOI: 10.1039/c5sc03658e


**DOI:** 10.1039/c5sc03658e

**Published:** 2016-01-04

**Authors:** Jing Guo, Yangbin Liu, Xiangqiang Li, Xiaohua Liu, Lili Lin, Xiaoming Feng

**Affiliations:** a Key Laboratory of Green Chemistry & Technology , Ministry of Education , College of Chemistry , Sichuan University , Chengdu 610064 , China . Email: liuxh@scu.edu.cn ; Email: xmfeng@scu.edu.cn ; Fax: +86 28 85418249 ; Tel: +86 28 85418249; b Collaborative Innovation Center of Chemical Science and Engineering , Tianjin , China

## Abstract

A chiral Lewis acid-promoted enantioselective cyclopropanation using phenyliodonium ylide as the carbene precursor was developed. A variety of spirocyclopropane-oxindoles with contiguous tertiary and all carbon quaternary centers were obtained in excellent outcomes (up to 99% yield, >19 : 1 d.r., up to 99% ee). EPR spectroscopy study supported a stepwise biradical mechanism.

## Abstract 2

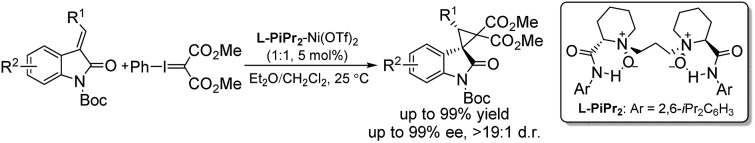
A chiral Lewis acid-promoted cyclopropanation using a phenyliodonium ylide as the carbene precursor was developed. An EPR spectroscopy study supported a stepwise biradical mechanism.

## Introduction

**Scheme 1:**
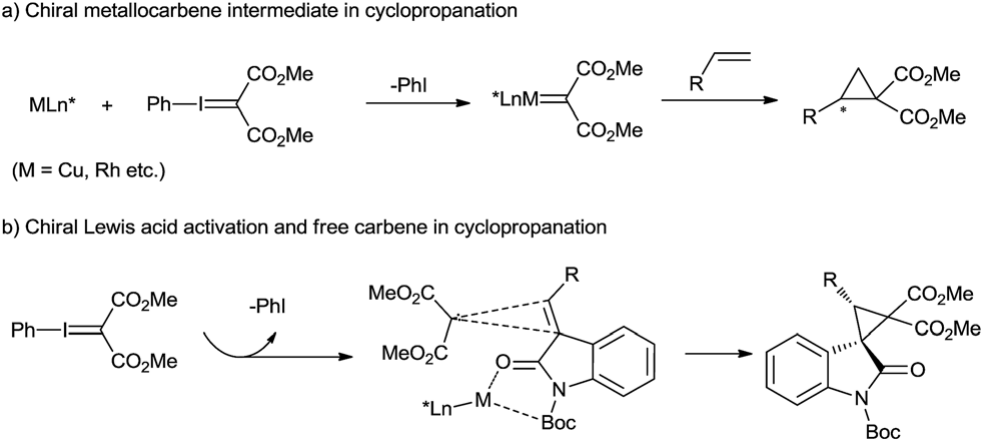
Catalytic enantioselective cyclopropanation of olefins with phenyliodonium ylide.

The catalytic asymmetric cyclopropanation of a C=C double bond provides efficient access to optically active cyclopropane derivatives that are essential motifs in natural products^1^ and valuable building blocks for various transformations.^2^ Chiral transition-metal-catalyzed carbene transfer, such as that using Cu(i)-, Rh(ii)-, Ru(ii)-, and Co(ii)-complexes with diazo compounds, permits such cylcopropanations in high yields and selectivities.^3^ However, in view of the mild, selective and non-toxic characteristics of phenyliodonium ylides, they could provide an alternative to diazo precursors in the cyclopropanation.^4^ The asymmetric cyclopropanation of simple olefins using phenyliodonium ylide was successfully developed, mainly with chiral [Rh_2_{(*S*)-nttl}_4_]^5^ and Cu(i)-bis(oxazoline) complexes.^6^ The reaction mechanism generally involves a metallocarbene intermediate surrounded by chiral ligands (Scheme 1a). However, the study of DeLuca and coworkers shows that thermal decomposition of phenyliodonium ylide malonate under mild conditions appears to be an efficient source of free singlet carbene,^7^ which can undergo uncatalyzed cyclopropanation stereospecifically. This presents an opportunity for asymmetric cyclopropanation *via* enantiocontrol of an olefin substrate in the carbene transfer step, but the difficulty of gaining high stereoselectivity and yield lies in the background reaction and the readiness of carbene dimer formation.

Spirocyclopropane-oxindoles are versatile building blocks for the synthesis of natural products and pharmaceuticals.^8^ The asymmetric catalytic cyclopropanations of electron-deficient oxindolic olefins were generally realized with Michael-Initiated Ring-Closing sequence (MIRC) reaction.^9^ Moreover, olefin cyclopropanation with diazooxindoles *via* the carbene transfer could also give access to these targets.^10^ Based on our long-term endeavor in the development of chiral catalysts stemmed from metal/*N*,*N*′-dioxide complexes^11^ as well as our previous study of oxindole derivatives,^12^ we envision that a chiral Lewis acid catalyst of *N*,*N*′-dioxide could bind 3-alkenyl-oxindoles into a perfect chiral environment, benefiting the cyclopropanation of a free carbene generated from spontaneous decomposition of phenyliodonium ylide malonate (Scheme 1b). Herein, we reported a chiral *N*,*N*′-dioxide/Ni(OTf)_2_ complex catalyzed asymmetric cyclopropanation of 3-alkenyl-oxindoles with phenyliodonium ylide. Excellent diastereo and enantioselectivity were achieved for a variety of substituted spirocyclopropane-oxindoles under mild reaction conditions. Free carbene species formation was confirmed from EPR and HRMS analysis of the reaction system.

## Results and discussion

Our investigation commenced with the cyclopropanation of (*E*)-*N*-Boc-3-alkenyl-oxindole **1a** as the model substrate with phenyliodonium ylide malonate **2** in CH_2_Cl_2_ at 25 °C. The known enantioselective activation of the substrate **1a** by metal complexes of chiral *N*,*N*′-dioxide **L-PiPr_2_** inspired us to examine it as the supporting ligand.^11^,^12^ CuBr only favored the carbene dimer of ethene-tetracarboxylate, and Cu(OTf)_2_ obtained a trace amount of the desired spirocyclopropane-oxindole **3a** (Table 1, entries 1 and 2). This indicated that the prior formation of metal–carbene intermediate was detrimental in this system. However, chiral Lewis acid catalysts of both Ni(OTf)_2_ and Zn(OTf)_2_ enabled access to the product **3a** in moderate yields and enantioselectivities yet high diastereoselectivities (>19 : 1 d.r.; entries 3 and 4). The results were appealingly consistent with our Lewis acid-activation pathway. The following survey of the ligands coordinated with Ni(OTf)_2_ showed that other *N*,*N*′-dioxides, including **L-PiPh**, **L-PrPr_2_** and **L-RaPr_2_**, were less competent than **L-PiPr_2_** in terms of the reactivities and enantioselectivities (entry 4 *vs.* entries 5–7). The reaction was further optimized through systematic study of several parameters. An improved enantioselectivity and moderate yield was observed in toluene (entry 8). An encouraging 85% yield with 98% ee of the product was given in Et_2_O (entries 9 and 10). It was suspected that Et_2_O may effectively prevent dimerization of the phenyliodonium ylide, but the poor solubility of the catalyst in Et_2_O led to the low performance of this catalytic system. Considering this question, a mixed solvent system was tested to improve the situation. A mixed solvent of CH_2_Cl_2_/Et_2_O (v/v = 1 : 4) was selected, and the asymmetric cyclopropanation gave a substantial improvement in catalytic yield with excellent diastereo and enantioselectivity (99% yield, >19 : 1 d.r., and 99% ee; entry 11).

**Table 1:** Optimization of the reaction conditions

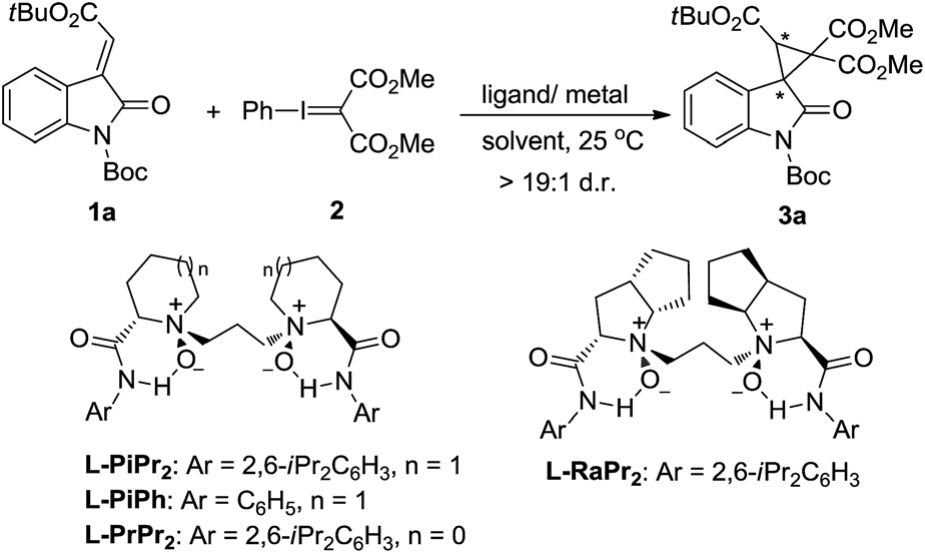					
Entry^*a*^	Metal salt	Ligand	Solvent	Yield^*b*^ (%)	ee^*c*^ (%)
1	CuBr	**L-PiPr_2_**	CH_2_Cl_2_	ND^*d*^	
2	Cu(OTf)_2_	**L-PiPr_2_**	CH_2_Cl_2_	Trace	—
3	Ni(OTf)_2_	**L-PiPr_2_**	CH_2_Cl_2_	65	65
4	Zn(OTf)_2_	**L-PiPr_2_**	CH_2_Cl_2_	33	77
5	Ni(OTf)_2_	**L-PiPh**	CH_2_Cl_2_	37	7^*e*^
6	Ni(OTf)_2_	**L-PrPr_2_**	CH_2_Cl_2_	39	22^*e*^
7	Ni(OTf)_2_	**L-RaPr_2_**	CH_2_Cl_2_	50	15
8	Ni(OTf)_2_	**L-PiPr_2_**	Toluene	58	97
9	Ni(OTf)_2_	**L-PiPr_2_**	THF	81	89
10	Ni(OTf)_2_	**L-PiPr_2_**	Et_2_O	85	98
11	Ni(OTf)_2_	**L-PiPr_2_**	CH_2_Cl_2_/Et_2_O (v/v = 1/4)	99	99

^*a*^The reactions were carried out with **1a** (0.1 mmol), metal/ligand (1 : 1, 5 mol%), and phenyliodonium ylide **2** (0.15 mmol) in a solvent (1.0 mL) at 25 °C for 24 h.

^*b*^Isolated yield.

^*c*^Determined by chiral HPLC analysis.

^*d*^Carbene dimer ethene-tetracarboxylate was the major product.

^*e*^The reverse of the enantioselectivity.

**Scheme 2:**
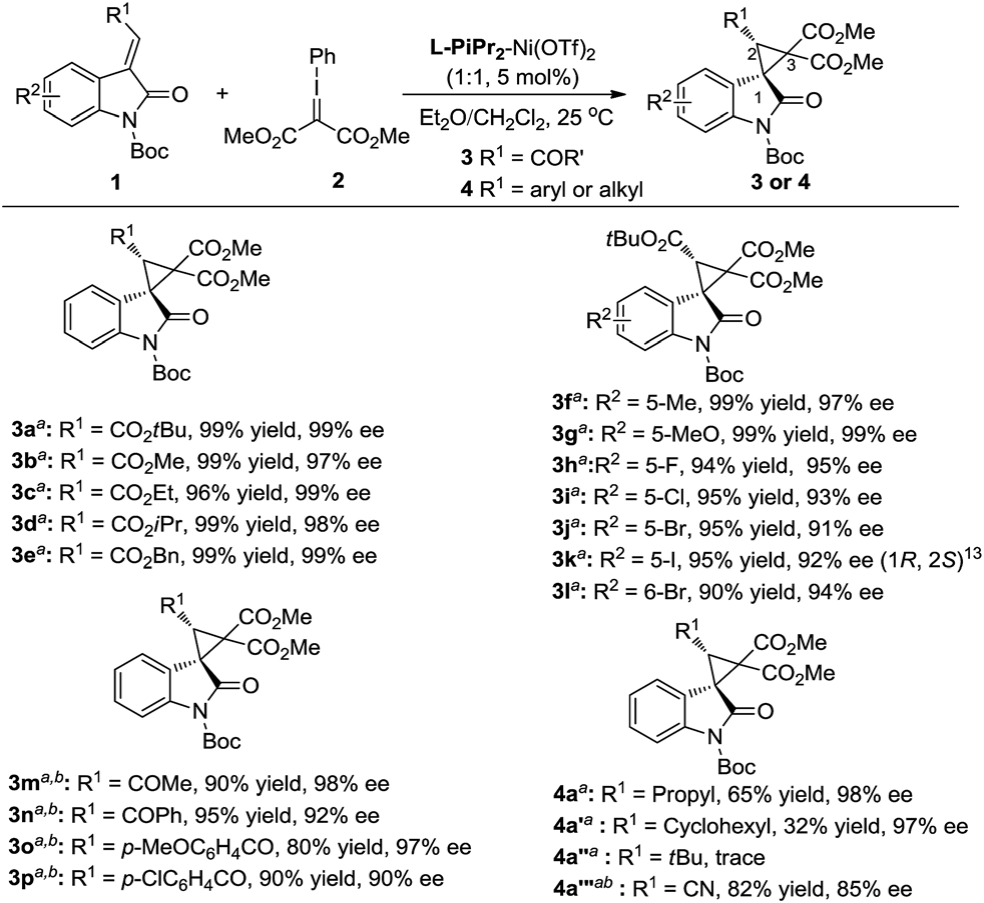
Substrate scope of the asymmetric cyclopropanation. ^*a*^ Reaction condition as in entry 11, Table 1.^*b*^ Reaction performed at 0 °C.

The generality of the catalytic cyclopropanation with a range of (*E*)-3-ester-substituted methyleneindolinone derivatives was next surveyed under the optimized reaction conditions (Scheme 2). The Me-, Et-, *i*Pr-, *t*Bu-, and Bn-substituted ester groups had no influence on the yields or the stereoselectivities (**3a–e**). The electron-donating substituents at the C5-position of the oxindole ring were well-tolerated, giving slightly higher enantioselectivities than the electron-withdrawing ones (**3f**, **g***vs.***3h–k**). A 6-bromo-substituent resulted in a good yield (90% yield, 94% ee; **3l**). 3-Acyl substituted methyleneindolinone derivatives produced the corresponding spirocyclopropane products **3m–p** in excellent yields and enantioselectivities at lower reaction temperature. The alkyl-substituted alkenes, such as propyl, cyclohexyl and cyano, could also undergo this reaction smoothly, affording the desired adducts in moderate yields and good enantioselectivities (**4a**, **4a′** and **4a′′′**). However, the reaction between (*E*)-1-Boc-3-*tert*-butylideneindolinone and phenyliodonium ylide **2** remained challenging due to the steric hindrance (**4a′′**).

**Scheme 3:**
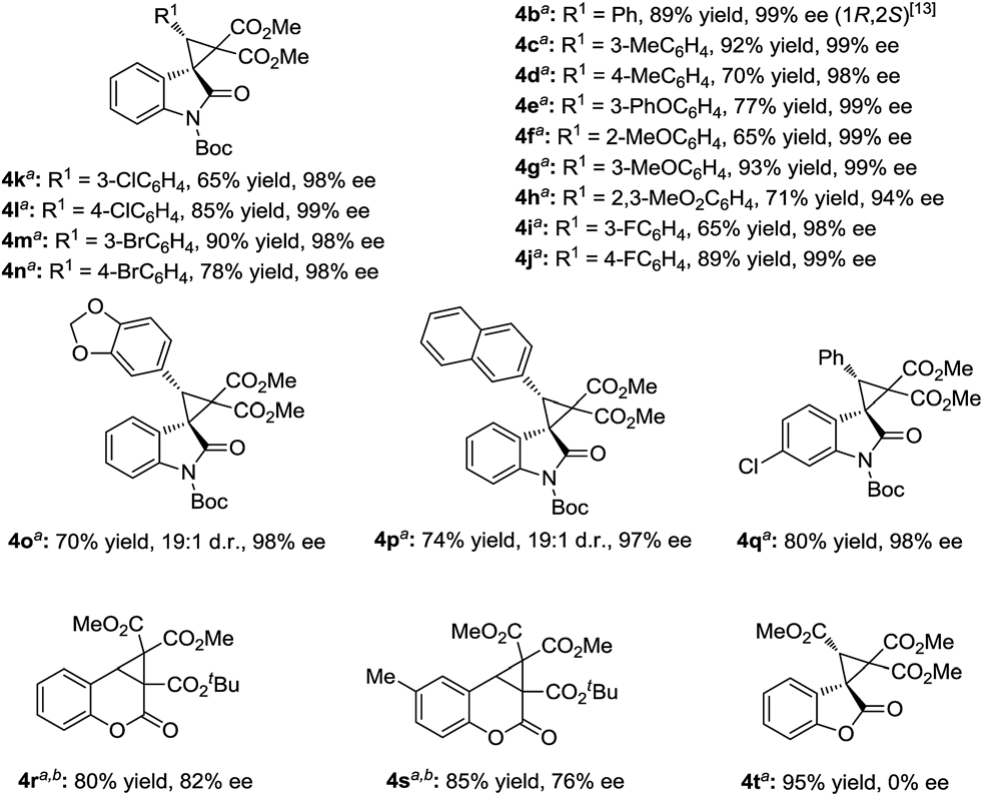
Substrate scope of aryl substituted olefins. ^*a*^ The reactions were carried out with **1a** (0.15 mmol), metal/ligand (1 : 1, 5 mol%), and phenyliodonium ylide **2** (0.1 mmol) in solvent (1.0 mL) at 25 °C for 48 h. ^*b*^ The solvent was changed from CH_2_Cl_2_/Et_2_O (v/v = 1 : 4) to MTBE.

However, the deprotection of the Boc-group of the 3-aryl-substituted methyleneindolinone derivatives occurred, which prevented the cyclopropanation process. Changing the ratio of the substrates **1** and phenyliodonium ylide **2** from 1 : 1.5 to 1.5 : 1 made the reaction more applicable. Therefore, a wide range of aryl substituted 3-alkenyl-oxindoles **1** was investigated (Scheme 3). The reactions went well, obtaining the corresponding products **4** in 60–93% yields with 94–99% ee. Both the electronic nature and the position of the substituents on the 3-aryl group of **1** had obvious influence on the yield but not the enantioselectivity (**4b–4n**). It is noteworthy that no diastereomers were detected in most cases (>19 : 1 d.r.), except for benzo[*d*][1,3]dioxole substituted **4o** and 2-naphthyl substituted **4p**. The sense of diastereoselectivity in the latter two cases was appreciably decreased, and a trace amount of the diastereomer was confirmed by ^1^H NMR spectroscopy (19 : 1 d.r.), whereas the enantioselectivity was unaffected. As a representative substituted, 3-benzylidene-indolinone underwent efficient cyclopropanation, giving **4q** in excellent yields. The absolute configuration of the product **3k** and **4b** was determined to be (1*R*, 2*S*) by X-ray analysis.^13^ When the catalytic system was applied to other non-oxindolic olefins, we found that coumarins were also able to provide the bridge ring derivatives with good yields and enantioselectivities (**4r–4s**). Benzofuran-2(3*H*)-one enabled access to the desired product with an excellent yield; however, the outcome of enantiocontrol was disappointing (**4t**). Compared with the *N*-Boc oxindoles, the loss of the necessary bidentate manner of two carbonyl groups might have led to poor chiral induction.

**Scheme 4:**
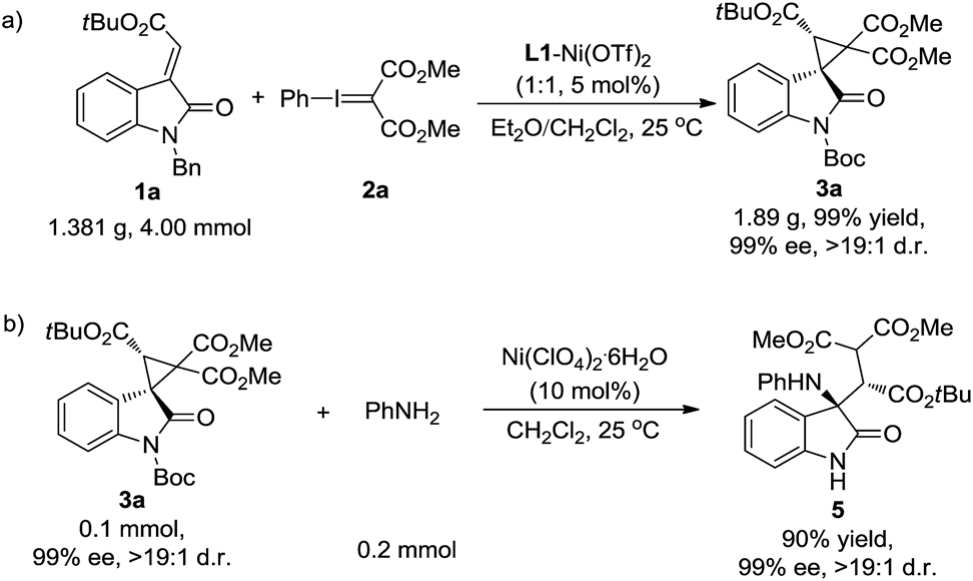
(a) Scaled-up version of the reaction and (b) the ring-open reaction of **3a** with aniline.

Furthermore, the synthetic value of the reaction was investigated. The cyclopropanation of oxindole **1a** with phenyliodonium ylide malonate **2** was carried out on a gram scale. The desired product **3a** was generated in 99% yield, >19 : 1 d.r. and 99% ee (Scheme 4a). On the other hand, the product **3a** could be easily transformed into the amino-functionalized acyclic β-amino acid derivative **5** (90% yield, 99% ee, and >19 : 1 d.r.) through a Lewis acid catalyzed nucleophilic ring-opening reaction using aniline as the nucleophile (Scheme 4b).

A series of experiments were conducted to probe into the reaction mechanism. The reaction between (*E*)-*N*-Boc-3-alkenyl-oxindole **1a** and phenyliodonium ylide malonate **2** proceeded smoothly in the absence of the catalyst, giving the desired product in 40% yield; neither the *N*,*N*′-dioxide nor Ni(OTf)_2_ could substantially enhance the reaction. The outcomes of the reaction were unaffected when carried out in the dark. Clearly, the catalytic system of *N*,*N*′-dioxide–Ni(OTf)_2_ is a ligand-accelerated process in view of the excellent yields previously discussed. The Ni(ii)-complex of **L-PiPr_2_** has been confirmed by X-ray analysis in our early study.11a–c The bonding of oxindole substrate **1a** or phenyl substituted 3-alkenyl-oxindole **1b′** to the metal cation of the chiral catalyst was detected from ESI-MS spectra. Peaks at *m*/*z* 1200.4629 and 1176.4937 were assigned to [Ni^2+^ + **L-PiPr_2_** + **1a** + TfO^–^]^+^ and [Ni^2+^ + **L-PiPr_2_** + **1b′** + TfO^–^]^+^, respectively (see ESI† for details).

**Fig. 1:**
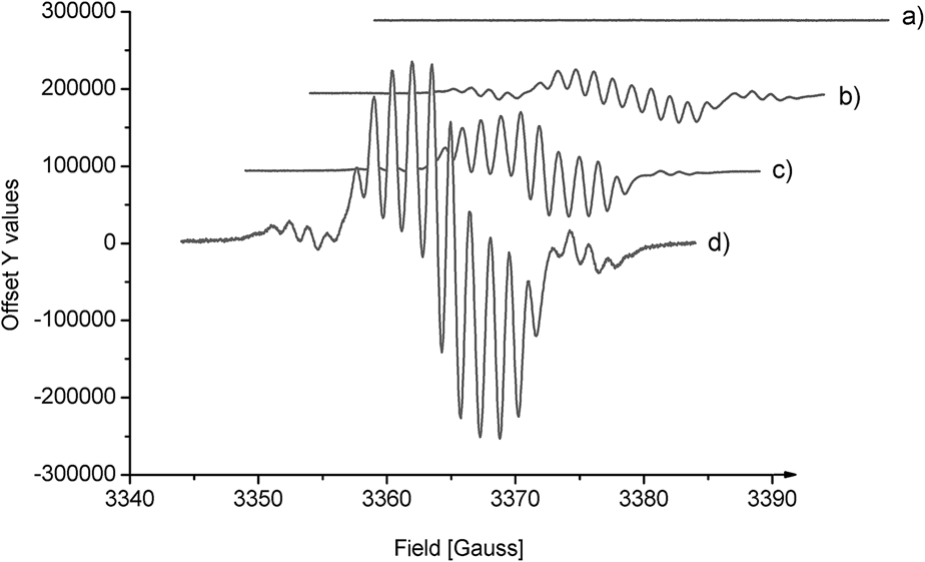
The electroparamagnetic resonance (EPR) spectra (X band, 9.43 GHz, RT, in Et_2_O/CH_2_Cl_2_ = 4/1). (a) **2**; (b) **2** and **1a** (1 : 1); (c) Ni(OTf)_2_ (20 mol%), **2** and **1a** (1 : 1); (d) Ni(OTf)_2_/**L-PiPr_2_** (20 mol%), **2** and **1a** (1 : 1).

To determine the carbene intermediate, the reaction system was further characterized by EPR spectroscopy. The EPR X-band spectrum of *N*,*N*′-dioxide–Ni(OTf)_2_ showed no signals, indicating that there is no unpaired electron on the nickel(ii) center due to the strong coordination of the supporting ligands. Interestingly, the EPR spectrum of the mixture of oxindole **1a** and phenyliodonium ylide **2** with or without the catalyst exhibits a similar rhombic band and is centered around *g* = 2.003 (Fig. 1b–d). The intensity of the band is stronger when the chiral catalyst is added (Fig. 1d *vs.*1b). The time profile of the reaction showed that the EPR band disappeared gradually. These results suggest the presence of unpaired electrons on the carbene intermediate. The reaction proceeded more likely *via* a free carbene intermediate than a metallocarbene, as in the studies of the Müller's^5^ and the Tang's.6*c* The intermediacy of such a free carbene upon thermal decomposition of phenyliodonium ylide **2** may be a singlet one ^1^:C(CO_2_Me)_2_ initially. The cyclopropanation was slower than intersystem crossing to the more stable triplet carbene ^3^:C(CO_2_Me)_2_, which exhibits two unpaired electrons. In this circumstance, the cyclopropanation occurs through a stepwise mechanism involving an analogous biradical intermediate. The unresolved hyperfine structure implied the interaction of ^3^:C(CO_2_Me)_2_ with the substrate.

**Scheme 5:**
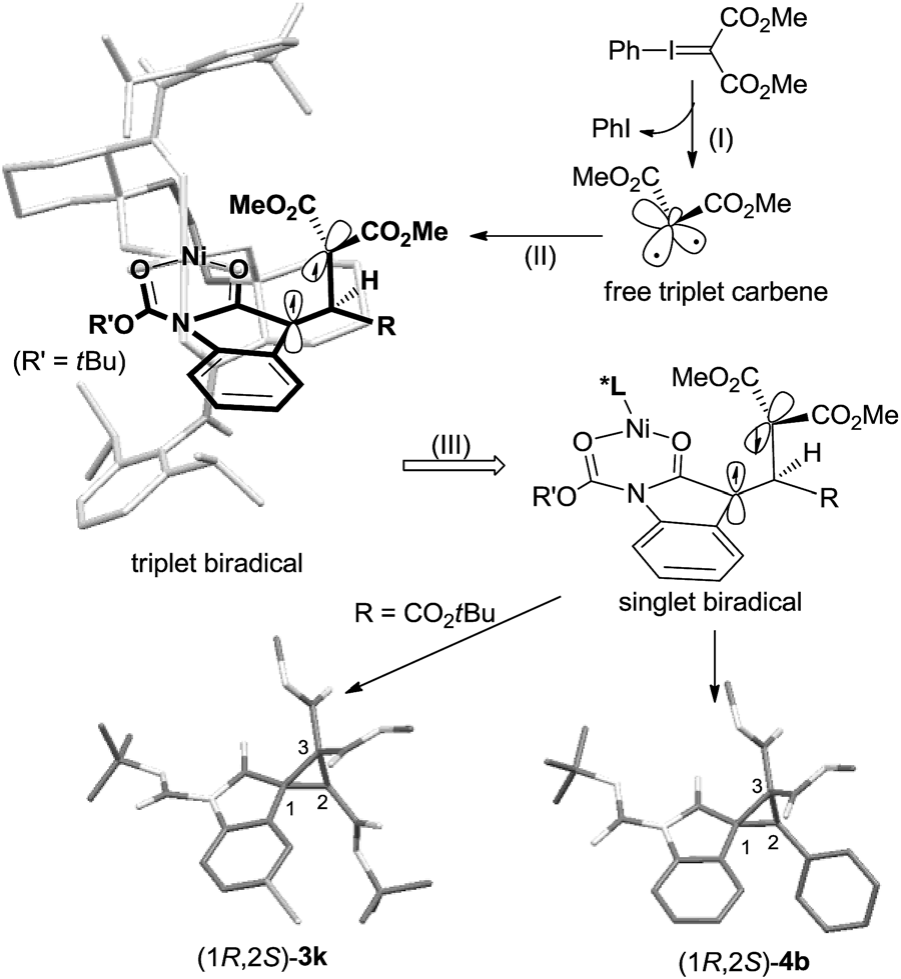
Proposed stereochemical model.

Therefore, in view of the aforementioned consequences as well as the structures of the catalyst^12^ and the products, we proposed a chiral Lewis acid-promoted asymmetric cyclopropanation mechanism *via* a free carbene intermediate (Scheme 5). Substrate **1** coordinates to the chiral *N*,*N*′-dioxide–Ni(ii) center in a bidentate manner with two carbonyl groups. The facial-control of the carbene addition was directed by the blocking of the amide unit underneath the ligand. Initially, the decomposition of the phenyliodonium ylide generated a more stable triplet carbene. It would prefer electronic addition to the outer C=C bond because of the low steric hindrance and the stability of the triplet biradical intermediate. Due to the steric hindrance of the substituents on the biradical intermediates, the C–C bond rotation is slower than spin flip of the intermediate. Therefore, high diastereo and enantioselectivity of the products were given.

## Conclusions

In conclusion, we have developed a new asymmetric catalytic strategy for cyclopropanation of olefins. The chiral *N*,*N*′-dioxide/Ni(OTf)_2_ complex exhibited excellent performance in the reaction of 3-alkenyl-oxindoles with phenyliodonium ylide malonate under mild reaction conditions. The desired spirocyclopropane-oxindoles with contiguous tertiary and all carbon quaternary centers were attained in high yields and stereoselectivities (up to 99% yield, >19 : 1 d.r., and 99% ee). At the same time, when the catalytic system was applied to other non-oxindolic olefins, coumarins were also able to provide the bridge ring derivatives with good yields and enantioselectivities. A stepwise biradical process is suggested based on EPR spectroscopy. Further application of iodonium ylides and chiral *N*,*N*′-dioxide–metal complexes in asymmetric transformations are underway.

## Supplementary Material

Supplementary information

Crystal structure data
