# Supplementary material for: Nickel(ii)-catalyzed enantioselective cyclopropanation of 3-alkenyl-oxindoles with phenyliodonium ylide via free carbene
Source: Chem Sci. 2016 Jan 4;7(4):2717–21. doi: 10.1039/c5sc03658e (PMC5477038; doi:10.1039/c5sc03658e)
Supplement: Supplementary file 1 [file SC-007-C5SC03658E-s001.pdf]

Electronic Supplementary Material (ESI) for Chemical Science.
This journal is © The Royal Society of Chemistry 2016

Supporting information

1. General remarks ……………………..………………………………………..……..……..……S2

2. General procedure for chiral *N*,*N*'-dioxide preparation………………………….…….………......S2

3. General procedure for the substractes **1** and **2**……………………..…….……….........................S2

4. General procedure for catalytic asymmetric cyclopropanation……....…......................................S3

5. Extra optimization of the reaction conditions…….……...…………………………......................S4

6. Preliminary mechanistic studies....………………..……………………..S6

7. The analytical and spectral characterization data of the cyclopropanation products…………….S11

8. References………………………………………………………………….............................S40

9. Copy of ^{1}H NMR and ^{13}C NMR spectra ……………………………..S41

1. General remarks

Reactions were carried out using commercial available reagents in over-dried apparatus. CH_2Cl_2 was dried over powdered CaH_2 and distilled under nitrogen just before use. Et_2O, THF, Toluene and PhOMe were directly distilled before use. Enantiomeric excesses (*ee*) were determined by HPLC analysis using the corresponding commercial chiral column as stated in the experimental procedures at 23 ºC with UV detector at 254 nm. Optical rotations were reported as follows: $[\alpha]^{25}_{D}$ (c g/100 mL, in solvent). ^{1}H NMR spectra were recorded on commercial instruments (400 MHz). Chemical shifts were reported in ppm from tetramethylsilane with the solvent resonance as the internal standard ($CDCl_3$, δ = 7.26). Spectra were reported as follows: chemical shift (δ ppm), multiplicity (s = singlet, d = doublet, t = triplet, q = quartet, m = multiplet), coupling constants (Hz), integration and assignment. ^{13}C NMR spectra were collected on commercial instruments (100 MHz) with complete proton decoupling. Chemical shifts are reported in ppm from the tetramethylsilane with the solvent resonance as internal standard ($CDCl_3$, δ = 77.0; DMSO, δ = 39.5). HRMS was recorded on a commercial apparatus (ESI Source).

2. General procedure for chiral *N,N'*-dioxide preparation

The *N,N'*-dioxide ligands were synthesized by the same procedure in the literature[1].

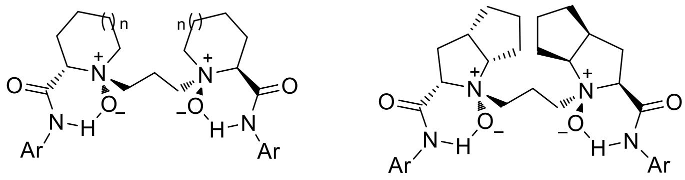

L-PiPr$_2$: Ar = 2,6-*i*Pr$_2$C$_6$H$_3$, n = 1
L-PiPh: Ar = C$_6$H$_5$, n = 1
L-PrPr$_2$: Ar = 2,6-*i*Pr$_2$C$_6$H$_3$, n = 0
L-RaPr$_2$: Ar = 2,6-*i*Pr$_2$C$_6$H$_3$

3. General procedure for substractes 1 and 2

A) Preparation of Boc-group of the 3-aryl/alkyl-substituted methyleneindolinone derivatives 1.[2]

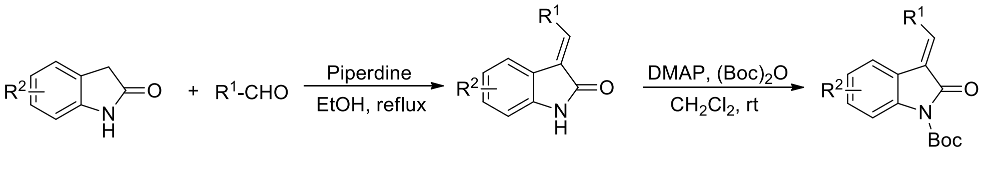

To oxindole (10 mmol) in EtOH (15 mL) was added the corresponding aldehyde (12 mmol) and piperdine (1 mmol). After refluxing for 8 hours, the reaction was cooled to room temperature. Crude product was purified by flash filter. The solid was dissolved by CH_2Cl_2 (30 mL), then DMAP (0.5 mmol) and $(Boc)_2O$ (12 mmol) were added. After stirring for 1 hour, the reaction was quenched by addition of 25 mL of cold water. The organic layer was then washed with cold water and brine. The organic layer was dried over $MgSO_4$, filtered, and concentrated. Crude product was purified by flash column silica gel chromatography to give the corresponding products.

Less than 5% of (*Z*)-isomer can be collected, and the 1H NMR was compared with the (*E*) isomer

shown below.

B) General procedure for preparation of Boc-protected arylidenoxindoles 1.[3]

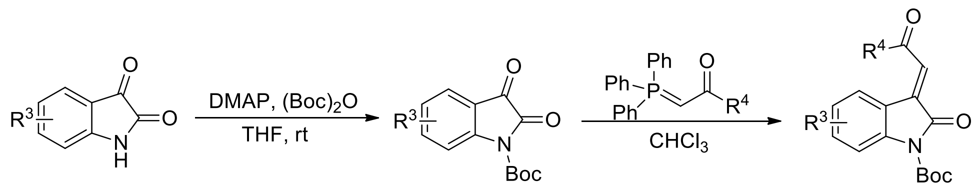

Wittig reagent (1 mmol, 1 equiv) was added to a solution of the Boc-protected isatin (2 mmol, 2 equiv) in $CHCl_3$ (5 mL) in a 25-mL round bottom flask. The solution was stirred at room temperature for 30 min. The mixture was purified by flash chromatography to afford the desired products about 60% yields.

C) General procedure for preparation of phenyliodonium ylide malonate 2.[4]

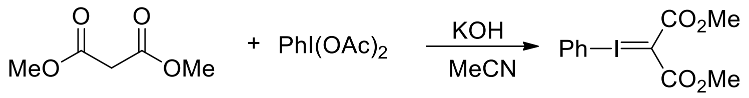

In a 50 mL flask under argon were added KOH (2.0g, 36.0 mmol) MeCN (20 mL) and dimethyl malonate (693 uL, 6.00mmol). The heterogenerous mixture was cooled at 0 ºC (ice/water bath) and stirred vigorously for 5 min to produce a milky white suspension. $PhI(OAc)_2$ (2.13g, 6.6 mmol) was then added in one portion and the reaction mixture was stirred vigorously for 2h at 0 ºC. The reaction mixture gradually became a thick creamy mixture. Water (10 mL) was then added and the mixture was stirred for 1 min. The beige/yellow biphasic solution containing a fluffy white suspension was filtered. The solid was washed with water. It is important that the solvent be completely removed between each wash. The solid was finally washed with EtOH and Et_2O then dried under high vacuum to yield **2** as an off-white solid.

D) General procedure for preparation of coumarins 1.[4]

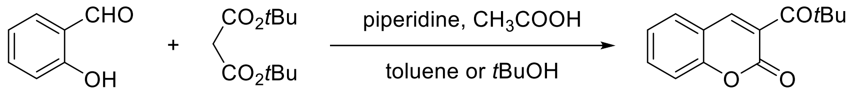

Salicylaldehyde (2.08 mL, 20 mmol), di-tert-butyl malonate (4.48 mL, 20 mmol) or prepared di-alkyl malonate, piperidine (0.25 mL, 12.5 mol%) and acetic acid (3 droplet) were added to 10 mL toluene or corresponding alcohol. The mixture was heated under reflux 12–14 h until salicylaldehyde disappeared. Then it was cooled to room temperature with the chemical salted out. Filter the desired product with ether and recrystallized from dicholormathane/n-hexane.

4. General procedure for the catalytic asymmetric cyclopropanation

Preparation of the chiral catalyst: *N,N'*-dioxide **L-PiPr₂** (0.1 mmol) and $Ni(OTf)_2$ (0.1 mmol) were stirred in 2.0 mL of CH_2Cl_2 at 30℃ for 30 min, and then dried under high vacuum.

General procedure for catalytic asymmetric reaction: A dry reaction tube was charged with **L-PiPr₂**-$Ni(OTf)_2$ (1:1, 5 mol%) and **1** (0.10 mmol) under N_2 atmosphere. Then, Et_2O (0.8 mL) and CH_2Cl_2 (0.2 mL) was added and the mixture was stirred at 25℃ for 15 min. Finally, phenylidonium ylide **2** (0.15 mmol) was added under stirring. The reaction mixture was stirred at 25℃ for 24–48 h. The residue was purified by flash chromatography (petroleum ether/ethyl acetate 8:1 to 4:1) on silica

gel to afford the product. The enantiomeric excess (*ee*) was determined by high-performance liquid chromatography (HPLC) with Chiralcel IA, Chiralcel ID.

Typical procedure for the scale-up reaction: A flask (100 mL) was charged with **L-PiPr$_2$**-Ni(OTf)$_2$ (1:1, 5 mol%) and **1a** (4.0 mmol) under N_2 atmosphere. Then, Et_2O (32 mL) and CH_2Cl_2 (8 mL) was added and the mixture was stirred at 25 ℃ for 15 min. Finally, phenylidonium ylide **2** (6.0 mmol) was added under stirring. The reaction mixture was stirred at 25 ℃ for 24 h. The residue was purified by flash chromatography (petroleum ether/ethyl acetate 8:1 to 4:1) on silica gel to afford the product **3a** as a white solid (1.900 g, >19:1 d.r. and 99% *ee*).

5. Optimization of the reaction conditions

A) Preliminary survey of the metal salts

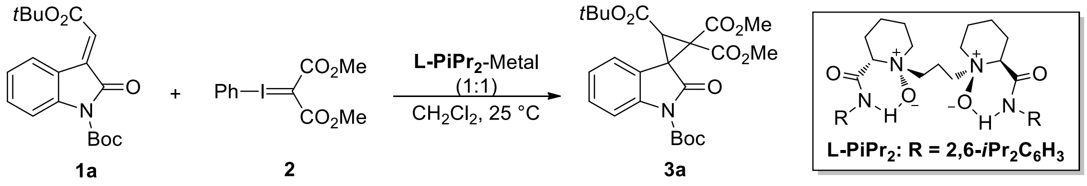

Entry[a]	Metal salt (mol%)	Yield (%)[b]	d.r.[c]	Ee (%)[d]
1	Sc(OTf)$_3$	trace	-	-
2	Cu(OTf)$_2$	trace	-	-
3	CuBr	N.D. [e]	-	-
4	Zn(OTf)$_2$	33	>19:1	77
5	Ni(OTf)$_2$	65	>19:1	65

[a] Unless otherwise noted, all reactions were performed with **L-PiPr$_2$**-metal salt (1:1, 5 mol%), **1a** (0.10 mmol), phenylidonium ylide **2** (0.15 mmol) in CH_2Cl_2 (1.0 mL) under N_2 at 25 ℃ for 24 h. [b] Isolated yield. [c] Determined by ^{1}H NMR spectroscopy and chiral HPLC analysis. [d] Determined by chiral HPLC analysis (Chiralcel IA). [e] Carbene dimer ethene-tetracarboxylate was the major product.

B) Survey of the chiral ligands

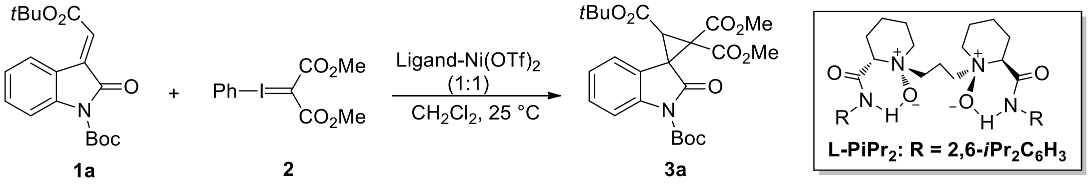

Entry[a]	Ligand (mol%)	Yield (%)[b]	d.r.[c]	Ee (%)[d]
1	**L-PiPr$_2$**	65	>19:1	65
2	**L-PiPh**	37	>19:1	7[e]
3	**L-PrPr$_2$**	39	>19:1	22[e]
4	**L-RaPr$_2$**	50	>19:1	15

[a] Unless otherwise noted, all reactions were performed with **L**-Ni(OTf)$_2$ (1:1, 5 mol%), **1a** (0.10 mmol), phenylidonium ylide **2** (0.15 mmol) in CH_2Cl_2 (1.0 mL) under N_2 at 25 ℃ for 24 h. [b] Isolated yield. [c]

Determined by ^{1}H NMR spectroscopy and chiral HPLC analysis. [d] Determined by chiral HPLC analysis (Chiralcel IA). [e] The reverse of the enantioselectivity.

C) Survey of the solvents

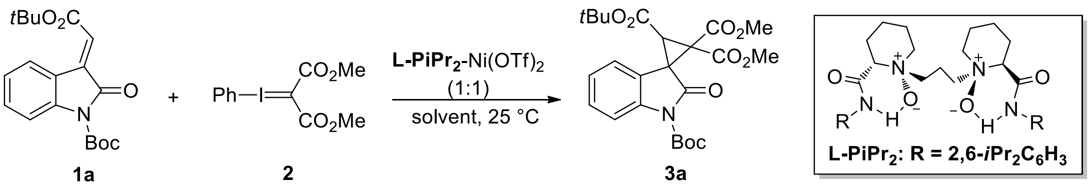

Entry[a]	Solvent	Yield (%)[b]	d.r.[c]	Ee (%)[d]
1	CH_2Cl_2	65	>19:1	65
2	THF	81	>19:1	89
3	Toluene	58	>19:1	97
4	Et_2O	85	>19:1	98
5	PhOMe	72	>19:1	99
6	MeO*t*Bu	79	>19:1	99
7	CH_2Cl_2 : Et_2O=1:4	99	>19:1	99

[a] Unless otherwise noted, all reactions were performed with **L-PiPr$_2$**-Ni(OTf)$_2$ (1:1, 5 mol%), **1a** (0.10 mmol), phenylidonium ylide **2** (0.15 mmol) in the corresponding solvent (1.0 mL) under N_2 at 25 ºC for 24 h. [b] Isolated yield. [c] Determined by ^{1}H NMR spectroscopy and chiral HPLC analysis. [d] Determined by chiral HPLC analysis (Chiralcel IA).

D) Optimization of the amount of the catalyst [a]

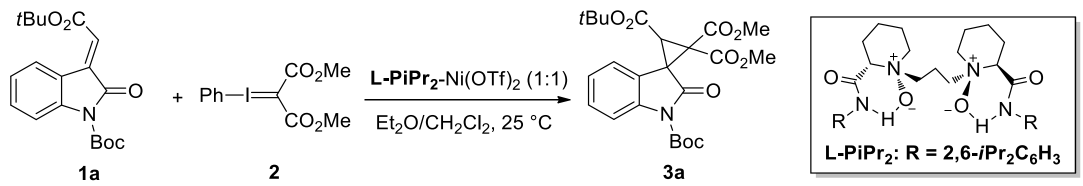

Entry[a]	Cat. Loading (x mol%)	Yield (%)[b]	d.r.[c]	Ee (%)[d]
1	5	99	>19:1	99
2	2.5	90	>19:1	97
3	1	80	>19:1	96
4	0.5	79	>19:1	95
5	0.1	45	>19:1	90

[a] Unless otherwise noted, all reactions were performed with **L-PiPr$_2$**-Ni(OTf)$_2$ (1:1, x mol%), **1a** (0.15 mmol), phenylidonium ylide **2** (0.1 mmol) in Et_2O (0.8 mL) and CH_2Cl_2 (0.2 mL) under N_2 at 25 ºC for 24 h. [b] Isolated

yield. [c] Determined by ^{1}H NMR spectroscopy and chiral HPLC analysis. [d] Determined by chiral HPLC analysis (Chiralcel IA).

6. Preliminary mechanism study

A) HRMS analysis

a) The mixture of **L-PiPr$_2$** and Ni(OTf)$_2$ (1:1).

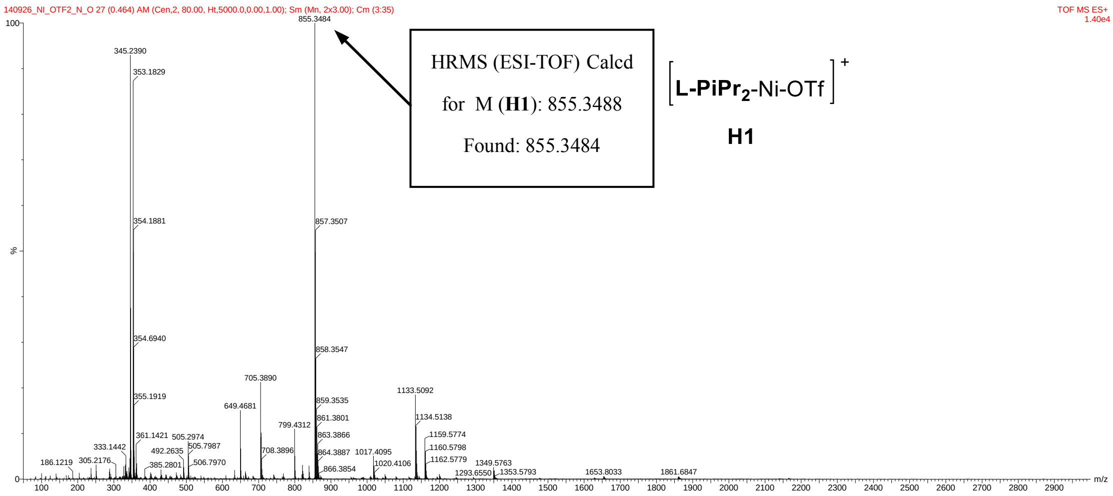

b) The mixture of **L-PiPr$_2$** , Ni(OTf)$_2$, **1a** and **2** (1:1:1:1).

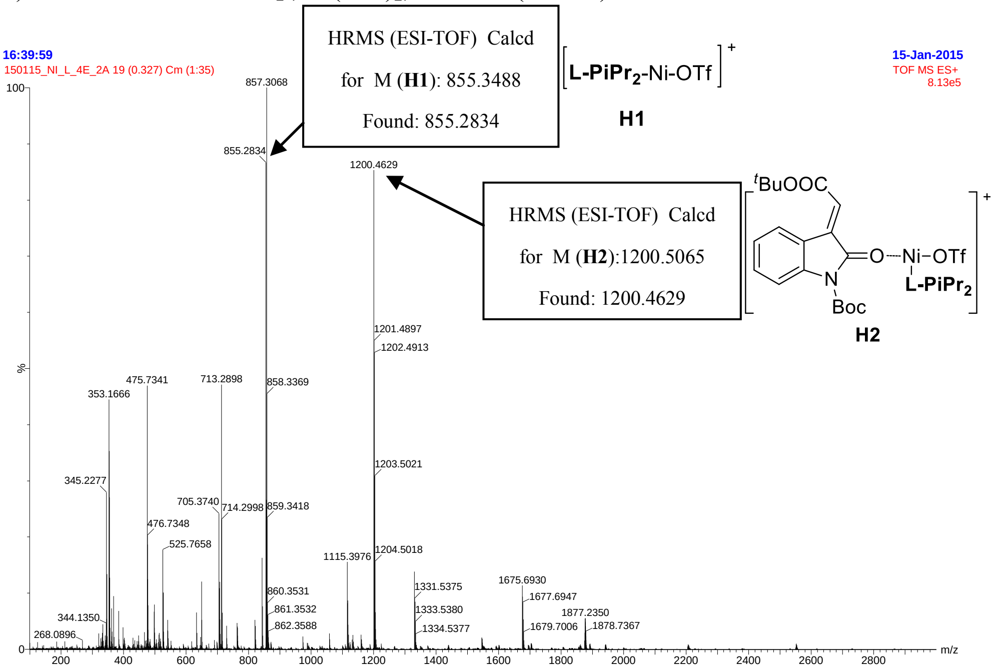

c) The mixture of **L-PiPr$_2$**, Ni(OTf)$_2$ and **1b'** (1:1:2).

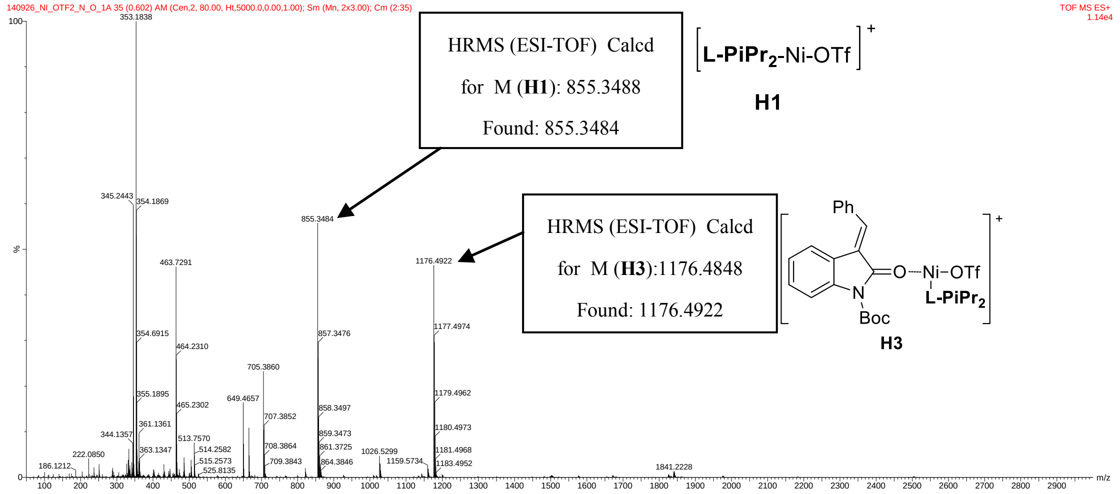

d) The mixture of **L-PiPr$_2$** , Ni(OTf)$_2$, **1b'** and **2** (1:1:2:3).

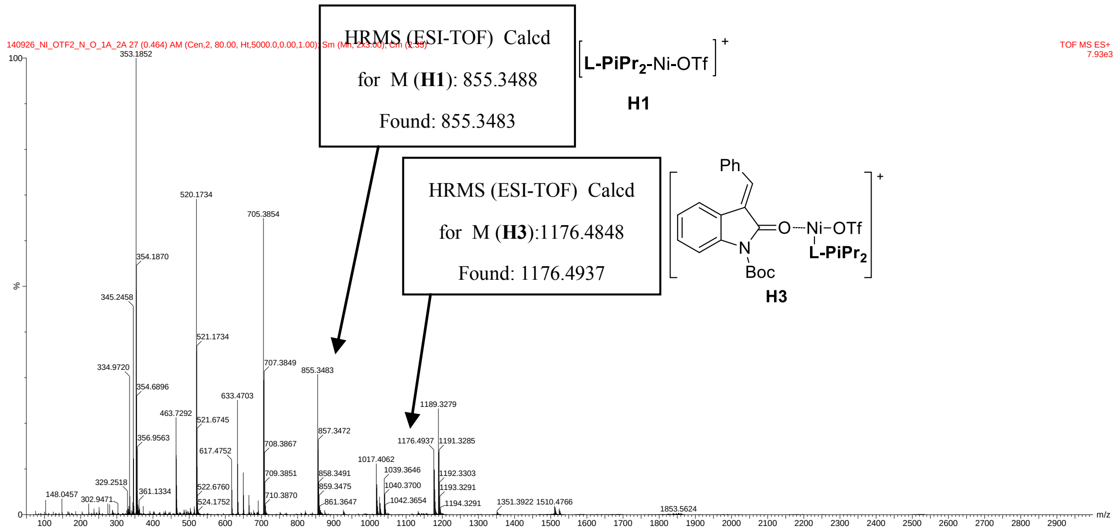

B) Electroparamagnetic resonance (EPR) analysis

EPR measurements: EPR spectra were recorded at room temperature on a Bruker ESP-300E: Receiver Gain = 1.78 e+003; Phase = 0 deg; Harmoni = 1; Mod. Frequency = 100.000 KHz; Mod. Amplitude = 0.50G; Center Field = 3364.010 G; Sweep width 40.000 G; Resolution = 2048 points; Conversion Time =40.00ms; Time const. = 20.48 m; Sweep time = 81.92s; Power = 29.55 mw.

No signal of the reagents as oxindoles **1a** or phenyliodonium ylide **2** appeared from 3344.010 G to 3384.010 G when **1a** or **2** (0.05 mmol) was stirred in Et_2O/CH_2Cl_2 (0.06 mL) at room temperature (Figure 1a and 1b). No signal appeared when Ni(OTf)$_2$ or **L-PiPr$_2$** or the complex of Ni(OTf)$_2$/**L-PiPr$_2$** (0.01 mmol) was stirred in Et_2O/CH_2Cl_2 (0.06 mL) at room temperature (Figure 1c–e). No signal appeared when Ni(OTf)$_2$ and phenyliodonium ylide **2** or the complex of Ni(OTf)$_2$/**L-PiPr$_2$** (0.01 mmol) and was phenyliodonium ylide **2** stirred in Et_2O/CH_2Cl_2 (0.06 mL) at room temperature (Figure 1f–g). Interestingly, the EPR spectrum of the mixture of oxindole **1a** and phenyliodonium ylide **2** with or without the catalyst exhibits a similar rhombic band and is centered around g = 2.003 (Figure 1h–k).

The intensity of the band is stronger when the chiral catalyst is added (Figure 1k vs 1h). After 5 hours, further as the reaction proceeded, the amount of carbenes gradually dropped (Figure 2). After the reaction was completed, the signal will disappear.

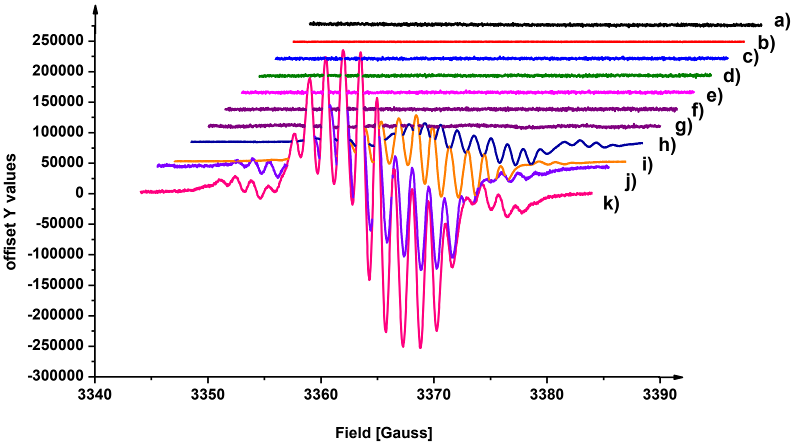

Figure 1 The electroparamagnetic resonance (EPR) spectra (X band, 9.43 GHz, RT; in Et_2O/CH_2Cl_2= 4/1 at room temperature) of a) **1a** (0.05 mmol); b) **2** (0.05 mmol); c) $Ni(OTf)_2$ (0.01 mmol); d) **L-PiPr₂** (0.01 mmol); e) the complex of $Ni(OTf)_2$/**L-PiPr₂** (0.01 mmol); f) $Ni(OTf)_2$ (0.01 mmol) and **2** (0.05 mmol); g) the complex of $Ni(OTf)_2$/**L-PiPr₂** (0.01 mmol) and **2** (0.05 mmol); h) **1a** (0.05 mmol) and **2** (0.05 mmol); i) $Ni(OTf)_2$ (0.01 mmol), **1a** (0.05 mmol) and **2** (0.05 mmol); j) **L-PiPr₂** (0.01 mmol), **1a** (0.05 mmol) and **2** (0.05 mmol); k) the complex of $Ni(OTf)_2$/**L-PiPr₂** (0.01 mmol), **1a** (0.05 mmol) and **2** (0.05 mmol).

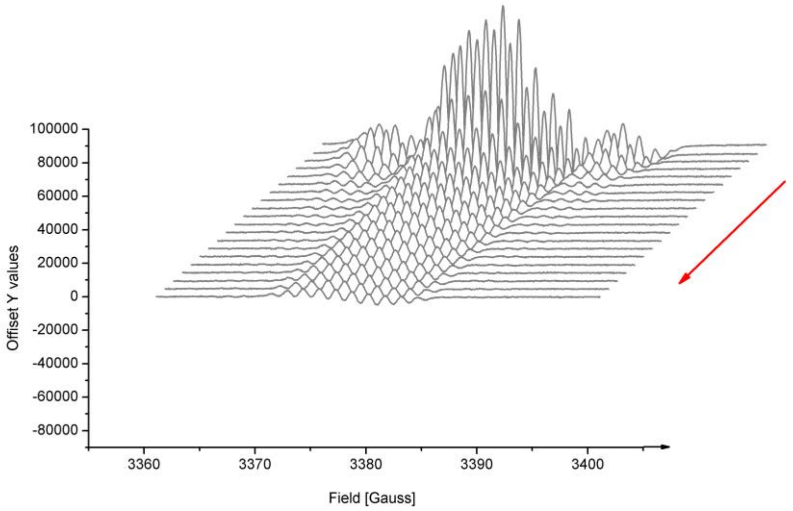

Figure 2 The electroparamagnetic resonance (EPR) spectra (X band, 9.43 GHz, RT) of the complex of $Ni(OTf)_2$/**L-PiPr₂** (0.01 mmol), **2** (0.05 mmol) and **1a** (0.05 mmol) in Et_2O/CH_2Cl_2= 4/1 at room temperature after 5h, scanning a spectrum every 5 min.

No signal of reagents as phenyl substituted 3-alkenyl-oxindole **1b’** or phenyliodonium ylide **2** appeared from 3344.010 G to 3384.010 G when **1b’** or **2** (0.05 mmol) was stirred in Et_2O/CH_2Cl_2 (0.06 mL) at room temperature (Figure 3a and 3b). Notably, the EPR spectrum of the mixture of oxindole **1b’** and phenyliodonium ylide **2** with or without the catalyst exhibits a similar rhombic band and is centered around g = 2.003 (Figure 3c–f). The intensity of the band is stronger when the chiral catalyst is added (Figure 3f vs 3c).

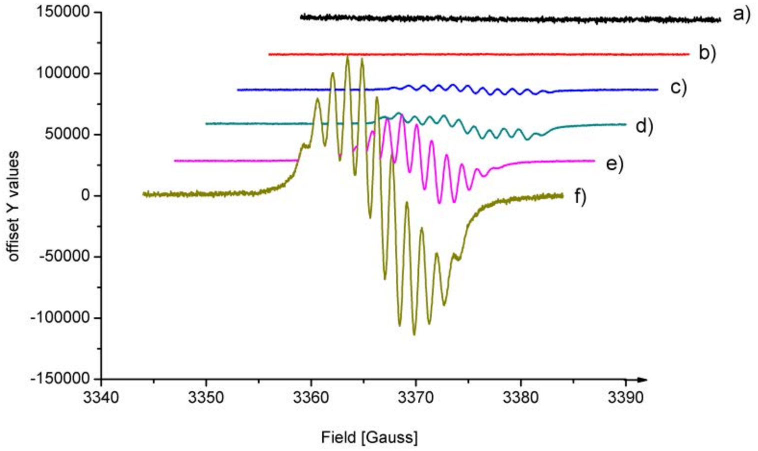

Figure 3 The electroparamagnetic resonance (EPR) spectra (X band, 9.43 GHz, RT; in Et_2O/CH_2Cl_2= 4/1 at room temperature) of a) **1r** (0.05 mmol); b) **2** (0.05 mmol); c) **1b’** (0.05 mmol) and **2** (0.05 mmol); d) **L-PiPr₂** (0.01 mmol), **1b’** (0.05 mmol) and **2** (0.05 mmol); e) $Ni(OTf)_2$ (0.01 mmol), **1b’** (0.05 mmol) and **2** (0.05 mmol); f) the complex of $Ni(OTf)_2$/**L-PiPr₂** (0.01 mmol), **1b’** (0.05 mmol) and **2** (0.05 mmol).

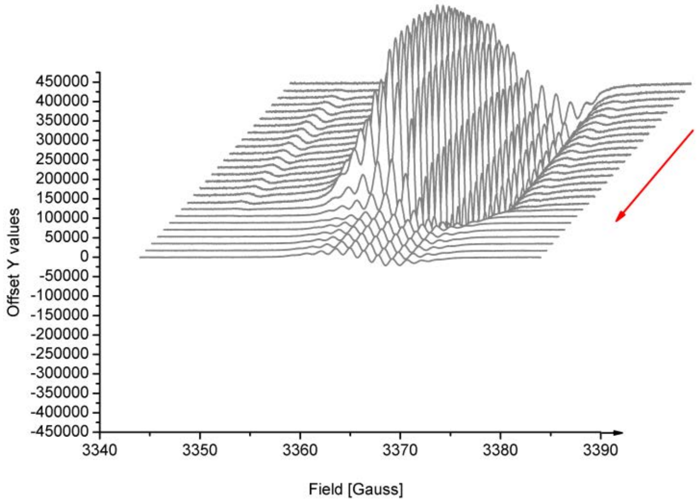

Figure 4 The electroparamagnetic resonance (EPR) spectra (X band, 9.43 GHz, RT) of the complex of $Ni(OTf)_2$/**L-PiPr₂** (0.01 mmol), **2a** (0.05 mmol) and **1b’** (0.05 mmol) in Et_2O/CH_2Cl_2= 4/1 at room temperature after 5 min, scanning a spectrum every 5 min.

C) Control experiment.

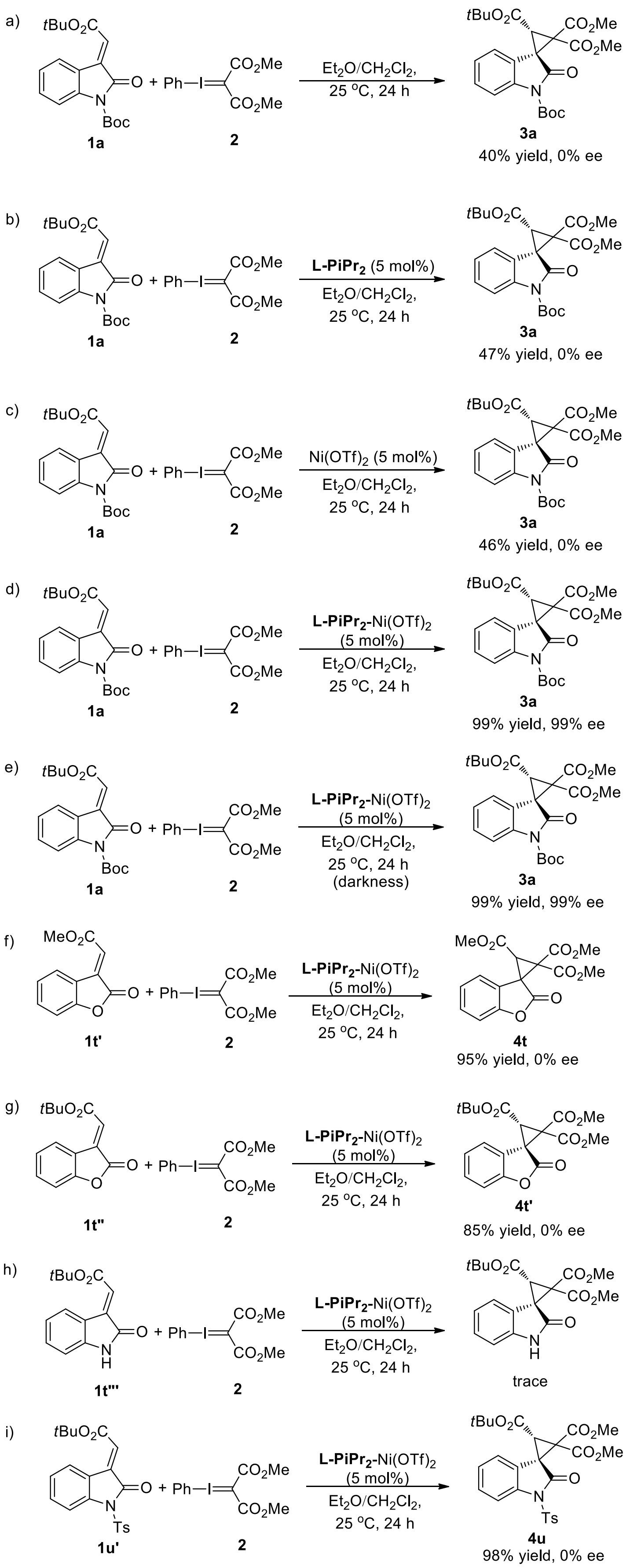

Scheme 1. Control experiment.

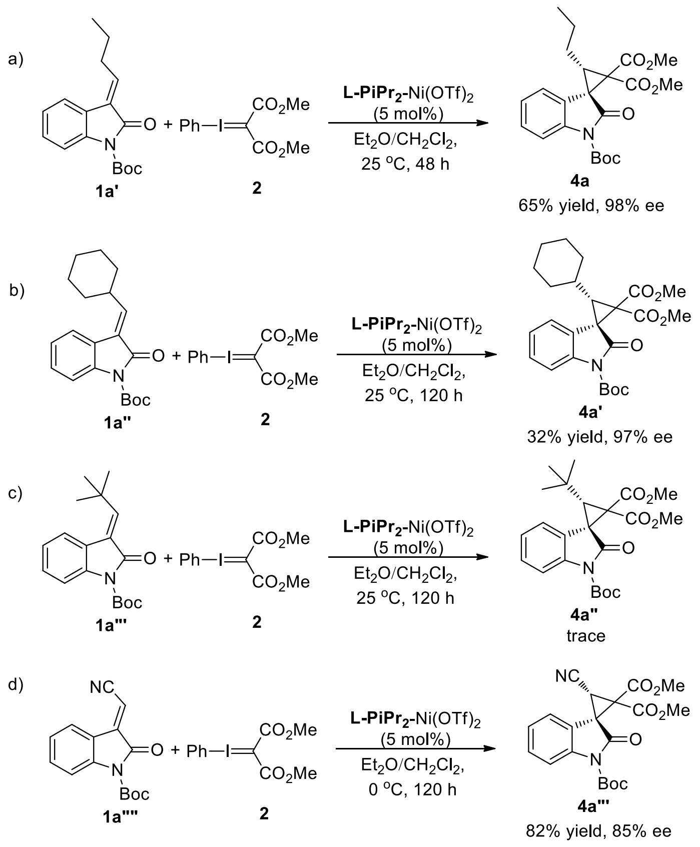

Scheme 2. Survey of other alkyl-substituted alkenes.

7. The analytical and spectral characterization data of the spirocyclopropanation products

(1*R*,3*S*)-1',3-di-tert-butyl 2,2-dimethyl 2'-oxospiro[cyclopropane-1,3'-indoline]-1',2,2,3-tetracarboxylate (3a):

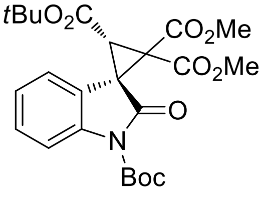

3a

Prepared according to the general procedure (24 h). The tittle compound **3a** was obtained as a white solid in 99% yield, >19:1 d.r., 99% *ee*. HPLC (Chiralcel IA, n-hexane/ *i*-PrOH = 98/2, flow rate 1.0 mL/min, λ = 254 nm) t_r (major) = 7.30 min, t_r (minor) = 11.52 min. $[\alpha]^{28.4}_D$ = −55.1 (*c* = 0.93, in CH_2Cl_2). ^1H NMR (400 MHz, $CDCl_3$) δ 7.88 (d, *J* = 8.4 Hz, 1H), 7.43 (d, *J* = 7.6 Hz, 1H), 7.37 – 7.31 (m, 1H), 7.14 – 7.08 (m, 1H), 3.80 (d, *J* = 4.4 Hz, 6H), 3.35 (s, 1H), 1.62 (s, 9H), 1.40 (s, 9H). ^{13}C NMR (100 MHz, $CDCl_3$) δ = 170.47, 164.76, 163.80, 163.10, 148.57, 140.79, 128.95, 126.60, 123.59, 119.71, 114.52, 84.88, 83.25, 53.59, 53.23, 47.41, 40.37, 39.26, 28.06, 27.96. HRMS (ESI-TOF) calcd for $C_{24}H_{29}NO_9$ ($[M]+Na^+$) = 498.1735, Found 498.1737.

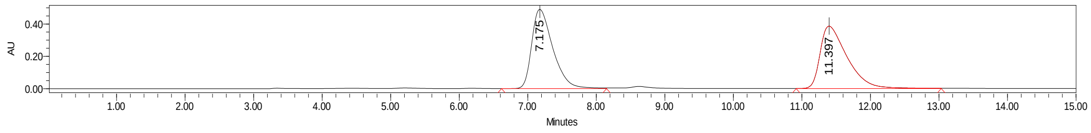

	Retention Time	Area	% Area

1	7.175	10542272	49.95
2	11.397	10561796	50.05

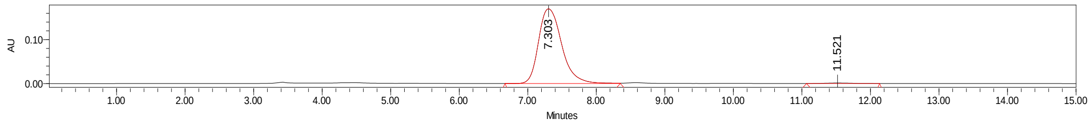

	Retention Time	Area	% Area
1	7.303	3984150	99.31
2	11.521	27512	0.69

Gram-scale synthesis of 1',3-di-tert-butyl 2,2-dimethyl 2'-oxospiro[cyclopropane-1,3'-indoline]-1',2,2,3- tetracarboxylate (3a):

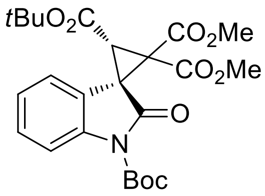

3a

Prepared according to the general procedure (24 h). The tittle compound **3a** was obtained as a white solid in 99% yield, >19:1 d.r., 99% *ee*. HPLC (Chiralcel IA, n-hexane/ *i*-PrOH = 98/2, flow rate 1.0 mL/min, λ = 254 nm) t_r (major) = 7.20 min, t_r (minor) = 11.60 min. ^{1}H NMR (400 MHz, $CDCl_3$) δ 7.89 (d, *J* = 8.2 Hz, 1H), 7.47 – 7.41 (m, 1H), 7.38 – 7.31 (m, 1H), 7.15 – 7.08 (m, 1H), 3.80 (d, *J* = 4.4 Hz, 6H), 3.36 (s, 1H), 1.63 (s, 9H), 1.41 (s, 9H). ^{13}C NMR (100 MHz, $CDCl_3$) δ = 170.46, 164.75, 163.80, 163.09, 148.57, 140.78, 128.95, 126.60, 123.59, 119.71, 114.51, 84.87, 83.24, 53.57, 53.21, 47.41, 40.37, 39.25, 28.05, 27.95.

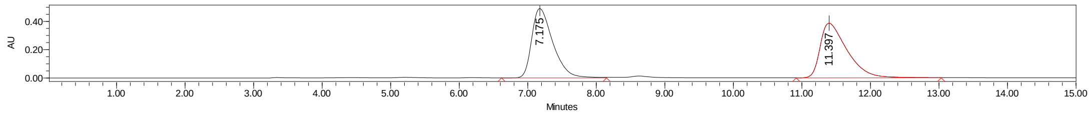

	Retention Time	Area	% Area
1	7.175	10542272	49.95
2	11.397	10561796	50.05

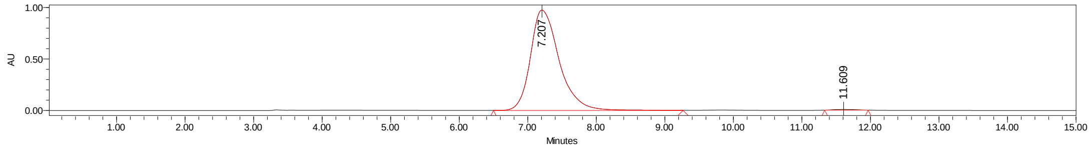

	Retention Time	Area	% Area
1	7.207	28358260	99.50
2	11.609	142440	0.50

(1*R*,3*S*)-1'-(tert-butyl) 2,2,3-trimethyl 2'-oxospiro[cyclopropane-1,3'-indoline]-1',2,2,3-tetracarboxylate (3b):

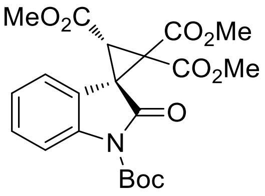

3b

Prepared according to the general procedure (24 h). The tittle compound **3b** was obtained as a colorless oil in 99% yield, >19:1 d.r., 97% *ee*. HPLC (Chiralcel IA, n-hexane/ *i*-PrOH = 98/2, flow rate 1.0 mL/min, λ = 254 nm)

t_r (major) = 13.16 min, t_r (minor) = 23.77 min. $[\alpha]^{28.1}_D$ = −61.8 (c = 0.67, in CH_2Cl_2). ^1H NMR (400 MHz, $CDCl_3$) δ 7.90 (d, J = 8.4 Hz, 1H), 7.42 – 7.34 (m, 2H), 7.13 (td, J = 7.6, 0.8 Hz, 1H), 3.81 (d, J = 0.8 Hz, 6H), 3.74 (s, 3H), 3.44 (s, 1H), 1.62 (s, 9H). ^{13}C NMR (100 MHz, $CDCl_3$) δ = 170.18, 165.40, 164.42, 162.84, 148.47, 140.91, 129.15, 126.43, 123.86, 119.54, 114.66, 84.97, 53.69, 53.43, 52.74, 47.66, 40.54, 37.97, 28.04. HRMS (ESI-TOF) calcd for $C_{21}H_{23}NO_9$ ($[M]+Na^+$) = 456.1265, Found 456.1268.

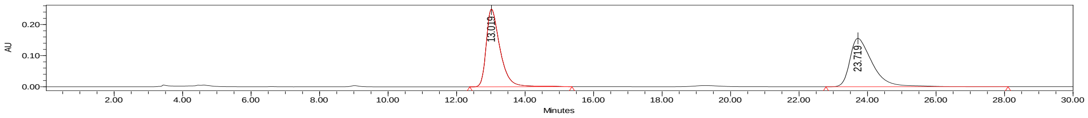

	Retention Time	Area	% Area
1	13.019	7006148	49.71
2	23.719	7088934	50.29

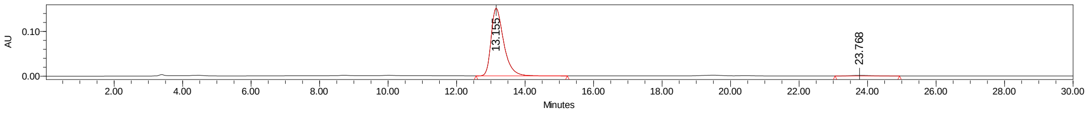

	Retention Time	Area	% Area
1	13.155	3844912	98.55
2	23.768	56656	1.45

(1*R*,3*S*)-1'-(tert-butyl) 3-ethyl 2,2-dimethyl 2'-oxospiro[cyclopropane-1,3'-indoline]-1',2,2,3-tetracarboxylate (3c):

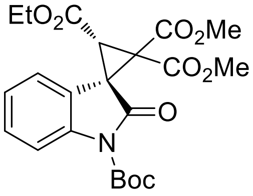

3c

Prepared according to the general procedure (24 h). The tittle compound **3c** was obtained as a colorless oil in 96% yield, >19:1 d.r., 99% *ee*. HPLC (Chiralcel IA, n-hexane/ *i*-PrOH = 98/2, flow rate 1.0 mL/min, λ = 254 nm) t_r (major) = 11.52 min, t_r (minor) = 20.25 min. $[\alpha]^{28.3}_D$ = −53.4 (c = -0.63, in CH_2Cl_2). ^1H NMR (400 MHz, $CDCl_3$) δ 7.89 (d, J = 8.4 Hz, 1H), 7.44 – 7.32 (m, 2H), 7.12 (td, J = 7.6, 0.8 Hz, 1H), 4.25 – 4.12 (m, 2H), 3.80 (s, 6H), 3.42 (s, 1H), 1.62 (s, 9H), 1.23 (t, J = 7.2 Hz, 3H). ^{13}C NMR (100 MHz, $CDCl_3$) δ = 170.26, 164.95, 164.52, 162.88, 148.50, 140.88, 129.11, 126.52, 123.80, 119.58, 114.62, 84.96, 62.05, 53.70, 53.39, 47.56, 40.50, 38.25, 28.05, 14.04. HRMS (ESI-TOF) calcd for $C_{22}H_{25}NO_9$ ($[M]+Na^+$) = 470.1422, Found 470.1424.

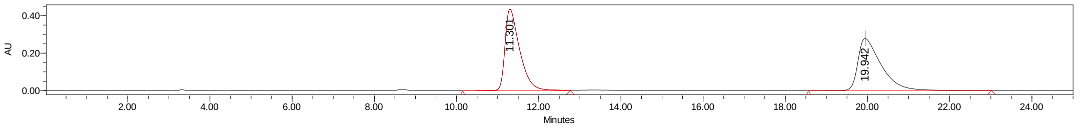

	Retention Time	Area	% Area
1	11.301	10823930	49.74
2	19.942	10938466	50.26

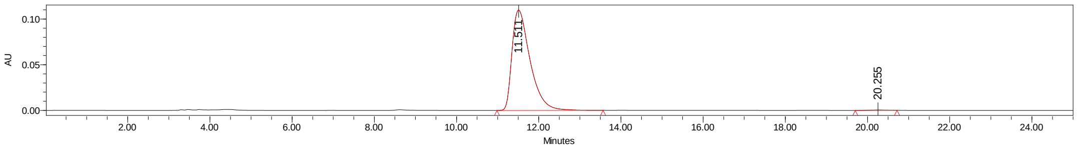

	Retention Time	Area	% Area
1	11.511	3348921	99.61
2	20.255	13208	0.39

(1*R*,3*S*)-1'-(tert-butyl) 3-isopropyl 2,2-dimethyl 2'-oxospiro[cyclopropane-1,3'-indoline]-1',2,2,3- tetracarboxylate (3d):

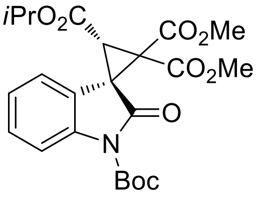

3d

Prepared according to the general procedure (24 h). The tittle compound **3d** was obtained as a colorless oil in 99% yield, >19:1 d.r., 98% *ee*. HPLC (Chiralcel IA, n-hexane/ *i*-PrOH = 98/2, flow rate 1.0 mL/min, λ = 254 nm) t_r (major) = 9.83 min, t_r (minor) = 16.51 min. $[\alpha]^{28.7}_{D}$ = −54.3(*c* = 0.60, in CH_2Cl_2). ^{1}H NMR (400 MHz, $CDCl_3$) δ 7.88 (d, *J* = 8.4 Hz, 1H), 7.41 (d, *J* = 8.0 Hz, 1H), 7.34 (t, *J* = 8.0 Hz, 1H), 7.10 (t, *J* = 7.6 Hz, 1H), 5.02 (hept, *J* = 6.4 Hz, 1H), 3.79 (s, 6H), 3.38 (s, 1H), 1.61 (s, 9H), 1.25 (d, *J* = 6.4 Hz, 3H), 1.12 (d, *J* = 6.4 Hz, 3H). ^{13}C NMR (100 MHz, $CDCl_3$) δ = 170.28, 164.57, 164.43, 162.93, 148.51, 140.84, 129.04, 126.53, 123.67, 119.59, 114.56, 84.90, 70.05, 53.61, 53.28, 47.49, 40.44, 38.46, 28.04, 21.74, 21.61. HRMS (ESI-TOF) calcd for $C_{23}H_{27}NO_9$ ([M]+Na$^+$) = 484.1578, Found 484.1579.

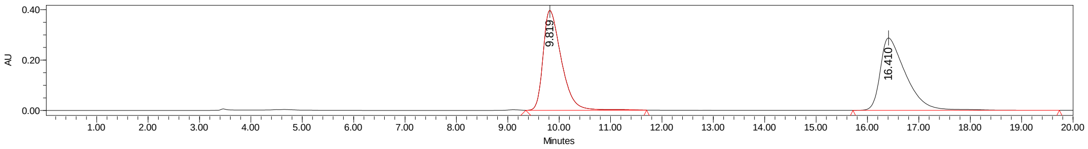

	Retention Time	Area	% Area
1	9.819	9783286	49.86
2	16.410	9837618	50.14

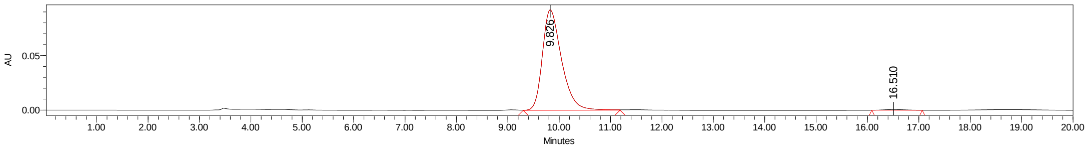

	Retention Time	Area	% Area
1	9.826	2383246	99.22
2	16.510	18824	0.78

(1*R*,3*S*)-3-benzyl 1'-tert-butyl 2,2-dimethyl 2'-oxospiro[cyclopropane-1,3'-indoline]-1',2,2,3-tetracarboxylate (3e):

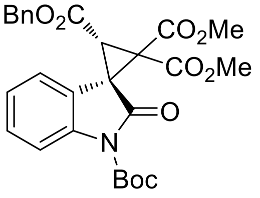

3e

Prepared according to the general procedure (24 h). The tittle compound **3e** was obtained as a colorless oil in 99% yield, >19:1 d.r., 99% *ee*. HPLC (Chiralcel IA, n-hexane/ *i*-PrOH = 98/2, flow rate 1.0 mL/min, λ = 254 nm) t_r (major) = 20.93 min, t_r (minor) = 27.49 min. $[\alpha]^{28.5}_{D}$ = −46.2 (*c* = 1.20., in CH_2Cl_2). ^{1}H NMR (400 MHz, $CDCl_3$) δ 7.89 (d, *J* = 8.4 Hz, 1H), 7.42 – 7.26

(m, 7H), 7.08 (t, J = 7.6 Hz, 1H), 5.16 (s, 2H), 3.80 (s, 3H), 3.76 (s, 3H), 3.47 (s, 1H), 1.62 (s, 9H). ^{13}C NMR (100 MHz, $CDCl_3$) δ = 170.13, 164.84, 164.43, 162.83, 148.47, 140.90, 134.74, 129.12, 128.58, 128.52, 126.50, 123.80, 119.46, 114.63, 84.95, 67.70, 53.68, 53.35, 47.58, 40.60, 38.16, 28.04. HRMS (ESI-TOF) calcd for $C_{27}H_{27}NO_9$ ([M]+Na$^+$) = 532.1578, Found 532.1587.

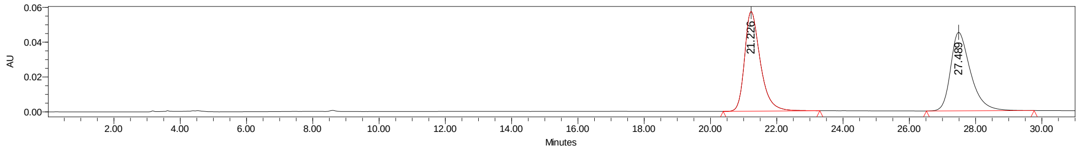

	Retention Time	Area	% Area
1	21.226	1882461	50.09
2	27.489	1875929	49.91

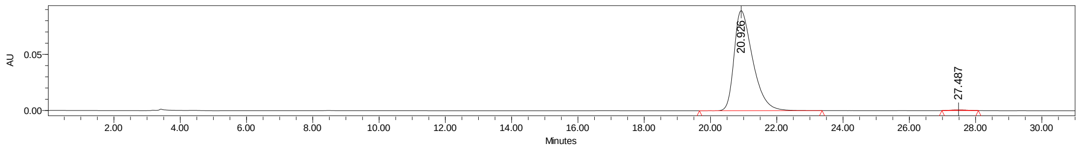

	Retention Time	Area	% Area
1	20.926	3476619	99.46
2	27.487	18968	0.54

(1*R*,3*S*)-1',3-di-tert-butyl 2,2-dimethyl 5'-methyl 2'-oxospiro[cyclopropane-1,3'-indoline] -1',2,2,3- tetracarboxylate (3f):

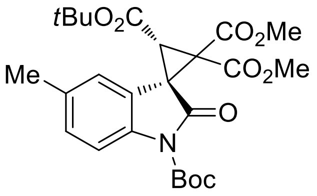

3f

Prepared according to the general procedure (24 h). The tittle compound **3f** was obtained as a colorless oil in 99% yield, >19:1 d.r., 97% *ee*. HPLC (Chiralcel IA, n-hexane/ *i*-PrOH = 98/2, flow rate 1.0 mL/min, λ = 254 nm) t_r (major) = 8.15 min, t_r (minor) = 10.43 min. $[\alpha]^{28.8}_D$ = −54.0 (c = 1.07, in CH_2Cl_2). ^{1}H NMR (400 MHz, $CDCl_3$) δ 7.75 (d, J = 8.4 Hz, 1H), 7.21 (s, 1H), 7.14 (dd, J = 8.4, 1.2 Hz, 1H), 3.79 (d, J = 9.6 Hz, 6H), 3.33 (s, 1H), 2.31 (s, 3H), 1.61 (s, 9H), 1.39 (s, 9H). ^{13}C NMR (100 MHz, $CDCl_3$) δ = 170.62, 164.87, 163.77, 163.12, 148.61, 138.41, 133.07, 129.45, 126.95, 119.64, 114.26, 84.70, 83.16, 53.56, 53.20, 47.23, 40.33, 39.29, 28.06, 27.92, 21.24. HRMS (ESI-TOF) calcd for $C_{25}H_{31}NO_9$ ([M]+Na$^+$) = 512.1891, Found 512.1895.

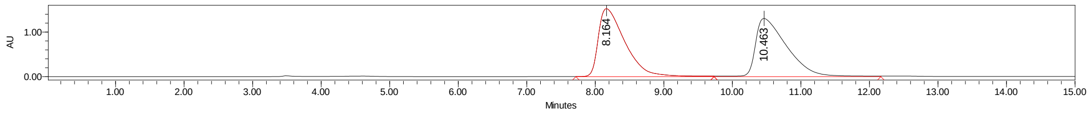

	Retention Time	Area	% Area
1	8.164	41215644	49.85
2	10.463	41457034	50.15

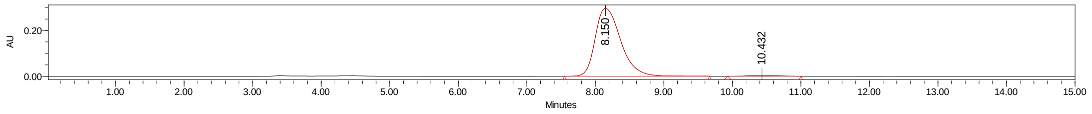

	Retention Time	Area	% Area
1	8.150	7647242	98.53
2	10.432	113821	1.47

(1*R*,3*S*)-1',3-di-tert-butyl 2,2-dimethyl 5'-methoxy 2'-oxospiro[cyclopropane-1,3'-indoline] -1',2,2,3- tetracarboxylate (3g):

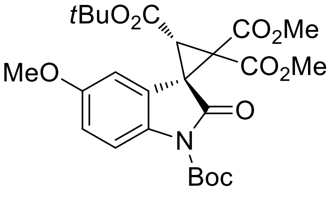

3g

Prepared according to the general procedure (24 h). The tittle compound **3g** was obtained as a colorless oil in 99% yield, >19:1 d.r., 99% *ee*. HPLC (Chiralcel IA, n-hexane/ *i*-PrOH = 98/2, flow rate 1.0 mL/min, λ = 254 nm) t_r (major) = 14.55 min, t_r (minor) = 13.36 min. $[\alpha]^{28.8}{}_D$ = −58.9 (*c* = 0.44, in CH_2Cl_2). ^{1}H NMR (400 MHz, $CDCl_3$) δ 7.80 (d, *J* = 9.2 Hz, 1H), 7.06 (d, *J* = 2.4 Hz, 1H), 6.88 (dd, *J* = 8.8, 2.8 Hz, 1H), 3.80 (d, *J* = 6.4 Hz, 6H), 3.77 (s, 3H), 3.35 (s, 1H), 1.62 (s, 9H), 1.42 (s, 9H). ^{13}C NMR (100 MHz, $CDCl_3$) δ = 170.54, 164.78, 163.80, 163.03, 155.92, 148.63, 134.23, 120.95, 115.22, 114.50, 112.61, 84.67, 83.23, 55.61, 53.60, 53.25, 47.44, 40.50, 39.32, 28.08, 27.97. HRMS (ESI-TOF) calcd for $C_{25}H_{31}NO_{10}$ ([M]+Na$^+$) = 528.1840, Found 528.1842.

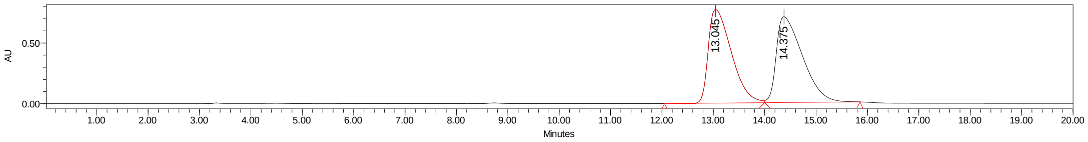

	Retention Time	Area	% Area
1	13.045	24134768	49.10
2	14.375	25019990	50.90

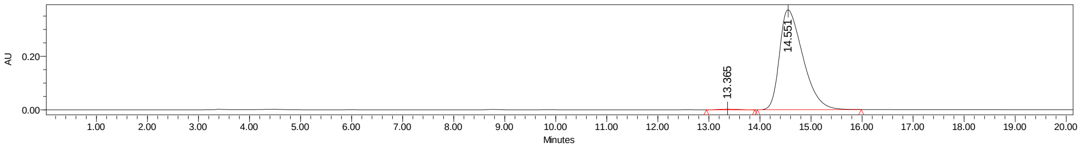

	Retention Time	Area	% Area
1	13.365	59479	0.50
2	14.551	11880044	99.50

(1*R*,3*S*)-1',3-di-tert-butyl 2,2-dimethyl 5'-fluoro 2'-oxospiro[cyclopropane-1,3'-indoline] -1',2,2,3- tetracarboxylate (3h):

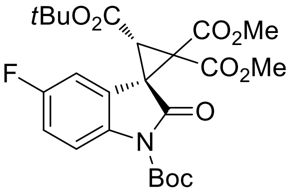

3h

Prepared according to the general procedure (24 h). The tittle compound **3h** was obtained as a colorless oil in 94% yield, >19:1 d.r., 95% *ee*. HPLC (Chiralcel IA, n-hexane/ *i*-PrOH = 98/2, flow rate 1.0 mL/min, λ = 254 nm) t_r (major) = 7.02 min, t_r (minor) = 8.93 min. $[\alpha]^{29.4}{}_D$ = −51.3 (*c* = 0.86, in CH_2Cl_2). ^{1}H NMR (400 MHz, $CDCl_3$) δ 7.87 (dd, *J* = 9.2, 4.8 Hz, 1H), 7.23 (dd, *J* = 9.6, 2.8 Hz, 1H), 7.05 (td, *J* = 8.8, 2.8 Hz, 1H), 3.80 (d, *J* = 7.6 Hz, 6H), 3.35 (s, 1H), 1.61 (s, 9H), 1.42 (s, 9H). ^{13}C NMR (100 MHz, $CDCl_3$) δ = 170.09, 164.47, 163.60, 162.94, 159.07 (d, *J* = 240), 148.52, 136.84 (d, *J* = 2), 121.63 (d, *J* = 10), 115.61 (d, *J* = 11) , 115.45 (d, *J* = 4), 114.45 (d, *J* = 28), 85.07, 83.59, 53.63, 53.36, 47.68, 40.24, 39.49, 28.04, 27.93. HRMS (ESI-TOF) calcd for $C_{24}H_{28}FNO_9$ ([M]+Na$^+$) = 516.1640, Found 516.1647.

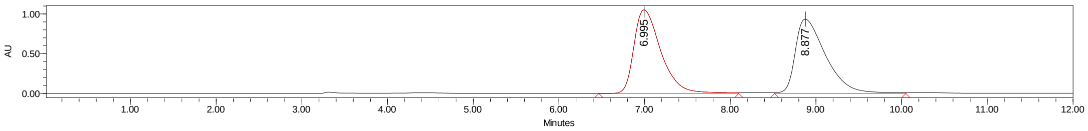

	Retention Time	Area	% Area
1	6.995	23085482	49.90
2	8.877	23179583	50.10

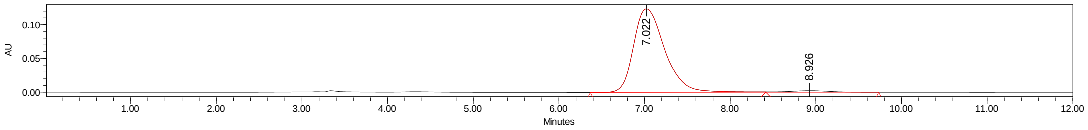

	Retention Time	Area	% Area
1	7.022	3134168	97.61
2	8.926	76770	2.39

(1*R*,3*S*)-1',3-di-tert-butyl 2,2-dimethyl 5'-chluoro 2'-oxospiro[cyclopropane-1,3'-indoline]-1',2,2,3- tetracarboxylate (3i):

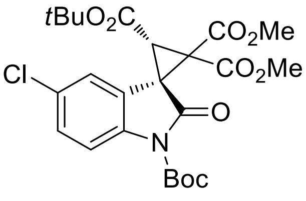

3i

Prepared according to the general procedure (24 h). The tittle compound **3i** was obtained as a white solid in 95% yield, >19:1 d.r., 93% *ee*. HPLC (Chiralcel IA, n-hexane/ *i*-PrOH = 98/2, flow rate 1.0 mL/min, λ = 254 nm) t_r (major) = 7.62 min, t_r (minor) = 9.71 min. $[\alpha]^{29.2}_D$ = −55.4 (*c* = 0.70, in CH_2Cl_2). ^1H NMR (400 MHz, $CDCl_3$) δ 7.85 (d, *J* = 8.8 Hz, 1H), 7.44 (d, *J* = 2.4 Hz, 1H), 7.32 (dd, *J* = 8.8, 2.4 Hz, 1H), 3.81 (d, *J* = 11.6 Hz, 6H), 3.35 (s, 1H), 1.61 (s, 9H), 1.42 (s, 9H). ^{13}C NMR (100 MHz, $CDCl_3$) δ = 169.82, 164.46, 163.49, 162.95, 148.40, 139.33, 129.29, 128.89, 126.82, 121.56, 115.61, 85.23, 83.72, 53.63, 53.38, 47.65, 40.02, 39.55, 28.03, 27.93. HRMS (ESI-TOF) calcd for $C_{24}H_{28}{}^{34.9589}ClNO_9$ ([M]+Na^+) = 532.1350, Found 532.1356; HRMS (ESI-TOF) calcd for $C_{24}H_{28}{}^{36.9659}ClNO_9$ ([M]+Na^+) = 534.1321, Found 532.1340.

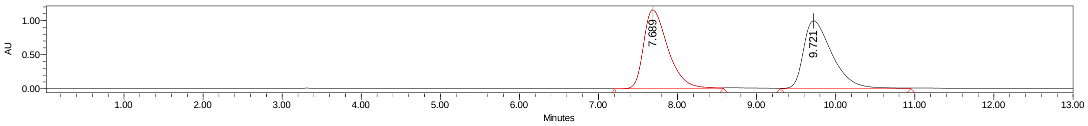

	Retention Time	Area	% Area
1	7.689	24859308	49.71
2	9.721	25146258	50.29

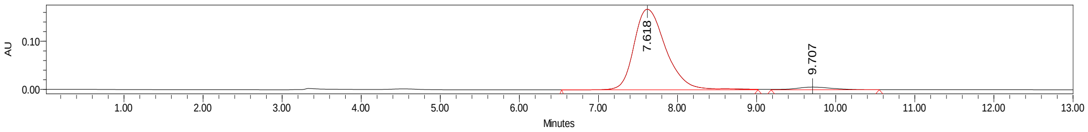

	Retention Time	Area	% Area
1	7.618	4708595	96.39
2	9.707	176451	3.61

(1*R*,3*S*)-1',3-di-tert-butyl 2,2-dimethyl 5'-bromo 2'-oxospiro[cyclopropane-1,3'-indoline]-1',2,2,3-tetracarboxylate (3j):

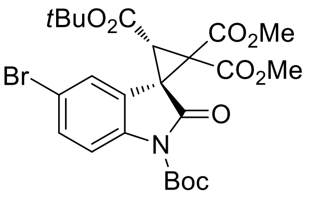

3j

Prepared according to the general procedure (24 h). The tittle compound **3j** was obtained as a white solid in 95% yield, >19:1 d.r., 91% *ee*. HPLC (Chiralcel IA, n-hexane/ *i*-PrOH = 98/2, flow rate 1.0 mL/min, λ = 254 nm) t_r (major) = 8.19 min, t_r (minor) = 10.29 min. $[\alpha]^{27.4}_{D}$ = −45.7 (*c* = 0.42, in CH_2Cl_2). ^{1}H NMR (400 MHz, $CDCl_3$) δ 7.80 (d, *J* = 8.8 Hz, 1H), 7.57 (d, *J* = 2.0 Hz, 1H), 7.47 (dd, *J* = 8.8, 2.0 Hz, 1H), 3.81 (d, *J* = 12.8 Hz, 6H), 3.35 (s, 1H), 1.61 (s, 9H), 1.43 (s, 9H). ^{13}C NMR (100 MHz, $CDCl_3$) δ = 169.70, 164.46, 163.46, 162.96, 148.37, 139.82, 131.81, 129.57, 121.90, 116.80, 116.03, 85.28, 83.78, 53.64, 53.40, 47.67, 39.90, 39.58, 28.03, 27.94. HRMS (ESI-TOF) calcd for $C_{24}H_{28}{}^{78.9183}BrNO_9$ ([M]+Na^+) = 576.0840, Found 576.0845; HRMS (ESI-TOF) calcd for $C_{24}H_{28}{}^{80.9163}BrNO_9$ ([M]+Na^+) = 578.0825, Found 578.0835.

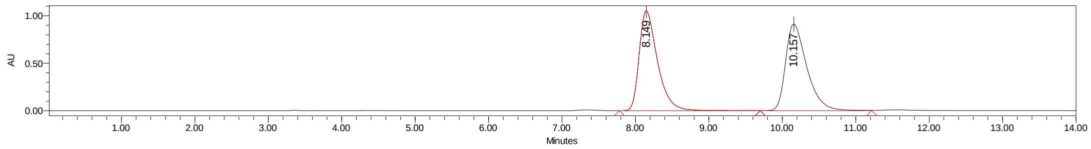

	Retention Time	Area	% Area
1	8.149	18317012	49.91
2	10.157	18379432	50.09

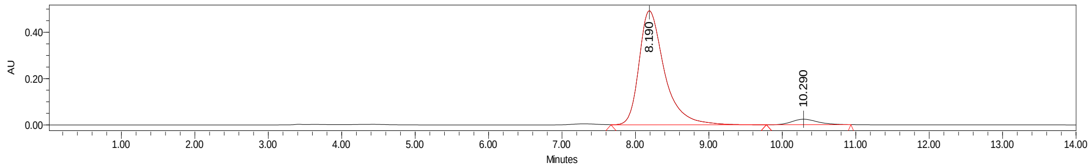

	Retention Time	Area	% Area
1	8.190	11785845	95.37
2	10.290	572108	4.63

(1*R*,3*S*)-1',3-di-tert-butyl 2,2-dimethyl 5'-iodo 2'-oxospiro[cyclopropane-1,3'-indoline] -1',2,2,3- tetracarboxylate (3k):

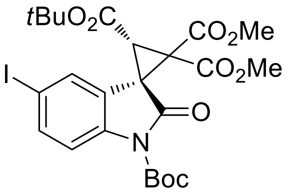

3k

Prepared according to the general procedure (24 h). The tittle compound **3k** was obtained as a white solid in 95% yield, >19:1 d.r., 92% *ee*. HPLC (Chiralcel IA, n-hexane/ *i*-PrOH = 98/2, flow rate 1.0 mL/min, λ = 254 nm) t_r (major) = 8.48 min, t_r (minor) = 10.47 min. $[\alpha]^{27.4}_{D}$ = −39.5(*c* = 1.03, in CH_2Cl_2). ^{1}H NMR (400 MHz, $CDCl_3$) δ 7.71 (s, 1H), 7.69 – 7.63 (m, 2H), 3.82 (s, 3H), 3.78 (s, 3H), 3.34 (s, 1H), 1.60 (s, 9H), 1.42 (s, 9H). 13C NMR (100 MHz, CDCl3) δ = 169.53, 164.46, 163.42, 162.97, 148.34, 140.52, 137.74, 135.19, 122.10, 116.47, 87.13, 85.28, 83.81, 53.64, 53.38, 47.64, 39.65, 39.55, 28.02, 27.97. HRMS (ESI-TOF) calcd for $C_{24}H_{28}INO_9$ ([M]+Na^+) = 642.0709, Found 642.0701.

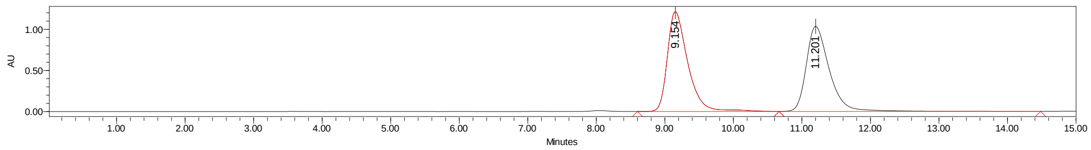

	Retention Time	Area	% Area
1	9.154	25120421	50.79
2	11.201	24339505	49.21

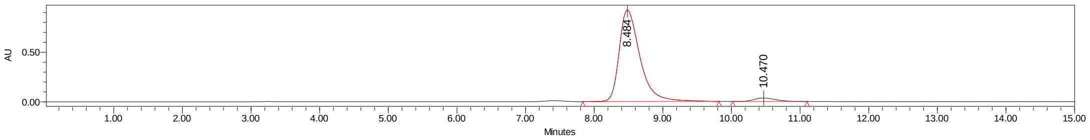

	Retention Time	Area	% Area
1	8.484	19378821	96.13
2	10.470	779496	3.87

(1*R*,3*S*)-1',3-di-tert-butyl 2,2-dimethyl 6'-bromo 2'-oxospiro[cyclopropane-1,3'-indoline]-1',2,2,3- tetracarboxylate (3l):

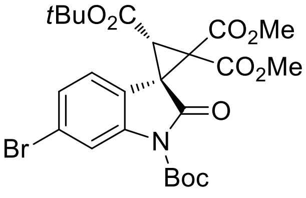

3l

Prepared according to the general procedure (24 h). The tittle compound **3l** was obtained as a white solid in 90% yield, >19:1 d.r., 94% *ee*. HPLC (Chiralcel IA, n-hexane/ *i*-PrOH = 98/2, flow rate 1.0 mL/min, λ = 254 nm) t_r (major) = 5.97 min, t_r (minor) = 9.77 min. $[\alpha]^{27.4}_D$ = −62.7 (*c* = 0.29, in CH_2Cl_2). ^1H NMR (400 MHz, $CDCl_3$) δ 8.13 (d, *J* =2.0 Hz, 1H), 7.31 (d, *J* = 8.4 Hz, 1H), 7.26 – 7.24 (m, 1H), 3.79 (d, *J* = 4.8 Hz, 6H), 3.34 (s, 1H), 1.62 (s, 9H), 1.41 (s, 9H). ^{13}C NMR (101 MHz, $CDCl_3$) δ = 169.93, 164.45, 163.71, 162.97, 148.30, 141.75, 127.88, 126.66, 123.00, 118.69, 118.07, 85.44, 83.55, 53.68, 53.35, 47.43, 40.11, 39.30, 28.01, 27.96. HRMS (ESI-TOF) calcd for $C_{24}H_{28}{}^{78.9183}BrNO_9$ ($[M]+Na^+$) = 576.0840, Found 576.0851; HRMS (ESI-TOF) calcd for $C_{24}H_{28}{}^{80.9163}BrNO_9$ ($[M]+Na^+$) = 578.0825, Found 578.0832.

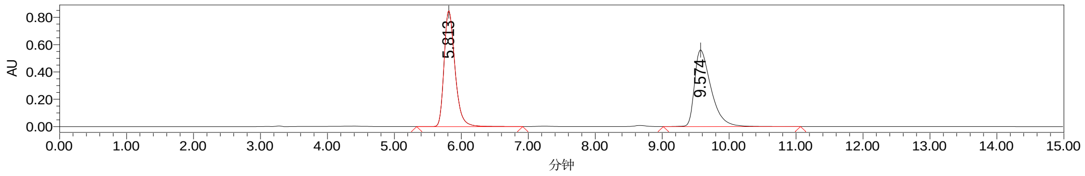

	Retention Time	Area	% Area
1	5.813	9585901	49.87
2	9.574	9637088	50.13

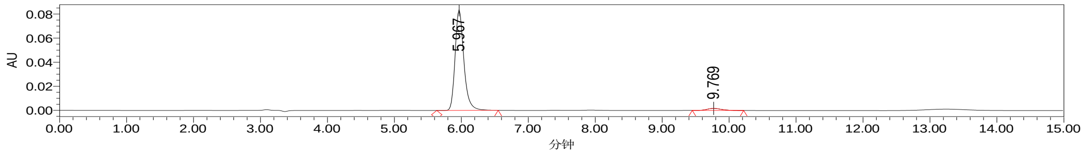

	Retention Time	Area	% Area
1	5.967	756620	97.37
2	9.769	20411	2.63

(1*R*,3*S*)-1'-tert-butyl 2,2-dimethyl 3-acetyl 2'-oxospiro[cyclopropane-1,3'-indoline]-1',2,2-tricarboxylate (3m):

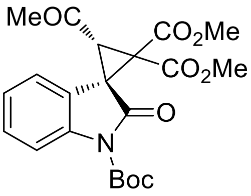

3m

Prepared according to the general procedure (15 h) below 0 °C. The tittle compound **3m** was obtained as a colorless oil in 90% yield, >19:1 d.r., 98% *ee*. HPLC (Chiralcel IA, n-hexane/ *i*-PrOH = 98/2, flow rate 1.0 mL/min, λ = 254 nm) t_r (major) = 8.15 min, t_r (minor) = 9.69 min. $[\alpha]^{26.4}_D$ = −73.2 (*c* = 0.46, in CH_2Cl_2). ^1H NMR (400 MHz, $CDCl_3$) δ 7.89 (d, *J* = 8.0 Hz, 1H), 7.40 – 7.31 (m, 1H), 7.22 (dd, *J* = 8.0, 1.2 Hz, 1H), 7.11 (td, *J* = 7.6, 0.8 Hz, 1H), 3.80 (d, *J* = 4.2 Hz, 6H), 3.55 (s, 1H), 2.31 (s, 3H), 1.63 (s, 9H). ^{13}C NMR (101 MHz, $CDCl_3$) δ = 198.51, 170.46, 164.66, 163.19, 148.48, 140.65, 129.12, 126.22, 123.96, 119.41, 114.66, 85.02, 53.71, 53.40, 48.41, 44.11, 41.50, 31.48, 28.04. HRMS (ESI-TOF) calcd for $C_{21}H_{23}NO_8$ ([M]+Na$^+$) = 440.1321, Found 440.1324.

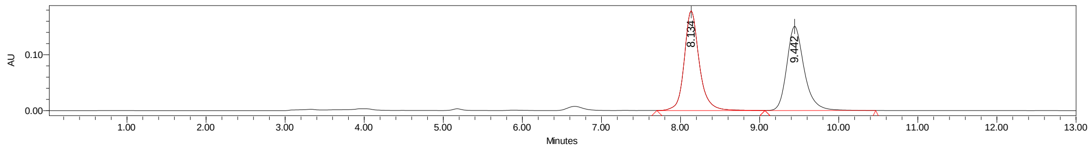

	Retention Time	Area	% Area
1	8.134	2343280	50.55
2	9.442	2291957	49.45

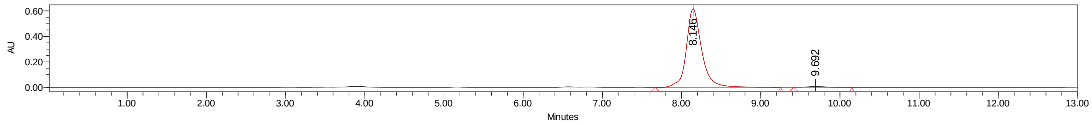

	Retention Time	Area	% Area
1	8.146	8252100	98.80
2	9.692	100215	1.20

(1*R*,3*S*)-1'(tert-butyl) 2,2-dimethyl 3-benzoyl 2'-oxospiro[cyclopropane-1,3'-indoline] -1',2,2-tricarboxylate (3n):

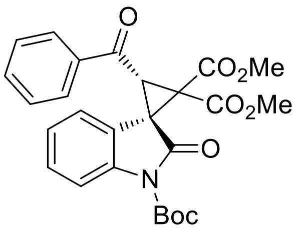

3n

Prepared according to the general procedure (24 h) below 0 °C. The tittle compound **3n** was obtained as a white solid in 95% yield, >19:1 d.r., 92% *ee*. HPLC (Chiralcel IA, n-hexane/ *i*-PrOH = 90/10, flow rate 1.0 mL/min, λ = 254 nm) t_r (major) = 11.67 min, t_r (minor) = 8.52 min. $[\alpha]^{26.5}_D$ = +18.3 (*c* = 0.93, in CH_2Cl_2). ^1H NMR (400 MHz, $CDCl_3$) δ 8.21 – 8.05 (m, 2H), 7.89 (d, *J* = 8.0 Hz, 1H), 7.58 (t, *J* = 7.2 Hz, 1H), 7.48 (t, *J* = 8.0 Hz, 2H), 7.37 – 7.27 (m, 2H), 7.14 – 7.06 (m, 1H), 4.09 (s, 1H), 3.85 (s, 3H), 3.69 (s, 3H), 1.64 (s, 9H). ^{13}C NMR (100 MHz, $CDCl_3$) δ = 189.84, 170.72, 165.25, 163.45, 148.53, 140.81, 136.43, 133.93, 129.06, 128.87, 128.48, 126.43, 123.93, 119.71, 114.66, 84.95, 53.67, 53.37, 49.48, 41.58, 41.01, 28.07. HRMS (ESI-TOF) calcd for $C_{26}H_{25}NO_8$ ([M]+Na$^+$) = 502.1478, Found 502.1485.

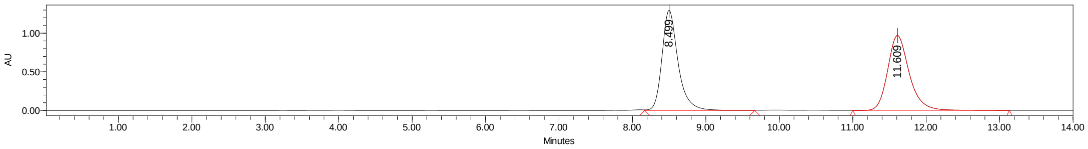

	Retention Time	Area	% Area
1	8.499	19715533	50.05
2	11.609	19677165	49.95

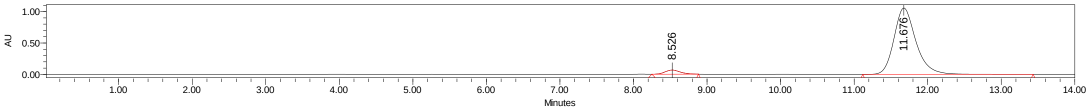

	Retention Time	Area	% Area
1	8.526	957268	4.19
2	11.676	21908578	95.81

(1*R*,3*S*)-1'(tert-butyl) 2,2-dimethyl 3-(4-methoxybenzoyl) 2'-oxospiro[cyclopropane-1,3'-indoline]-1',2,2- tricarboxylate (3o):

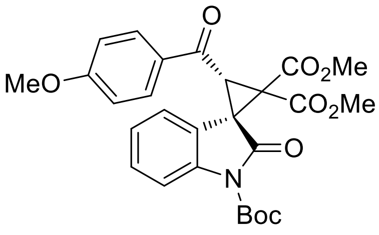

3o

Prepared according to the general procedure (48 h) below 0 °C. The tittle compound **3o** was obtained as a white solid in 80% yield, >19:1 d.r., 97% *ee*. HPLC (Chiralcel IA, n-hexane/ *i*-PrOH = 90/10, flow rate 1.0 mL/min, λ = 254 nm) t_r (major) = 22.69min, t_r (minor) = 17.81 min. $[\alpha]^{20.6}_D$ = −4.7 (*c* = 2.13, in CH_2Cl_2). ^1H NMR (400 MHz, $CDCl_3$) δ 8.13 – 8.02 (m, 2H), 7.93 – 7.82 (m, 1H), 7.33 – 7.29 (m, 2H), 7.11 – 7.04 (m, 1H), 6.98 – 6.86 (m, 2H), 4.07 (s, 1H), 3.84 (d, *J* = 2.8 Hz, 6H), 3.71 (s, 3H), 1.63 (s, 9H). ^{13}C NMR (100 MHz, $CDCl_3$) δ = 188.14, 170.85, 165.39, 164.18, 163.55, 148.55, 140.74, 130.89, 129.59, 128.95, 126.57, 123.87, 119.81, 114.58, 114.07, 84.90, 55.54, 53.64, 53.31, 49.20, 41.56, 41.04, 28.07. HRMS (ESI-TOF) calcd for $C_{27}H_{27}NO_9$ ($[M]+Na^+$) = 532.1584, Found 532.1593.

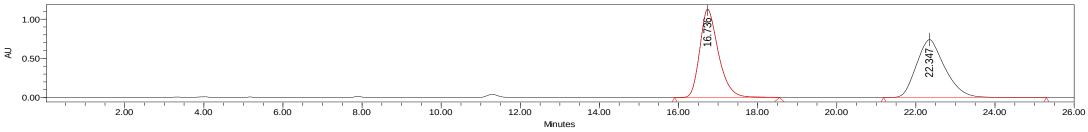

	Retention Time	Area	% Area
1	16.736	36921060	50.13
2	22.347	36735273	49.87

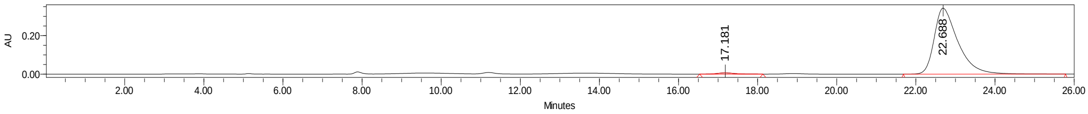

	Retention Time	Area	% Area
1	17.181	192934	1.33
2	22.688	14308297	98.67

(1*R*,3*S*)-1'(tert-butyl) 2,2-dimethyl 3-(4-chlorobenzoyl) 2'-oxospiro[cyclopropane-1,3'-

indoline]-1',2,2- tricarboxylate (3p):

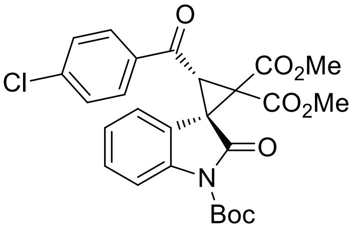

3p

Prepared according to the general procedure (48 h) below 0 °C. The tittle compound **3p** was obtained as a white solid in 90% yield, >19:1 d.r., 90% *ee*. HPLC (Chiralcel IA, n-hexane/ *i*-PrOH = 90/10, flow rate 1.0 mL/min, λ = 254 nm) t_r (major) = 13.35 min, t_r (minor) = 9.50 min. $[\alpha]^{23.3}_D$ = +50.4 (*c* = 1.01, in CH_2Cl_2). ^{1}H NMR (400 MHz, $CDCl_3$) δ 8.15 – 8.08 (m, 2H), 7.90 (d, *J* = 8.4 Hz, 1H), 7.51 – 7.42 (m, 2H), 7.38 – 7.31 (m, 1H), 7.24 (s, 1H), 7.14 – 7.07 (m, 1H), 3.99 (s, 1H), 3.85 (s, 3H), 3.67 (s, 3H), 1.64 (s, 9H). ^{13}C NMR (100 MHz, $CDCl_3$) δ = 188.61, 170.60, 165.21, 163.35, 148.49, 140.86, 140.44, 134.82, 129.86, 129.24, 129.19, 126.21, 123.98, 119.56, 114.74, 85.00, 53.72, 53.49, 49.74, 41.43, 40.63, 28.07. HRMS (ESI-TOF) calcd for $C_{26}H_{24}{}^{34.9589}ClNO_8$ ([M]+Na$^+$) = 536.1088, Found 536.1092; HRMS (ESI-TOF) calcd for $C_{26}H_{24}{}^{36.9659}ClNO_8$ ([M]+Na$^+$) = 538.1059, Found 538.1074.

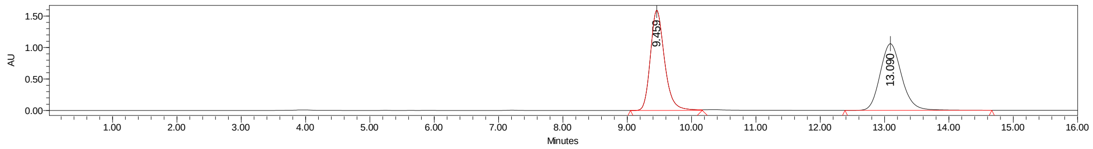

	Retention Time	Area	% Area
1	9.459	24228135	49.79
2	13.090	24434384	50.21

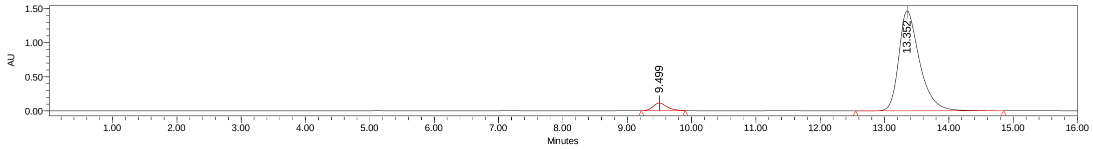

	Retention Time	Area	% Area
1	9.499	1687724	4.88
2	13.352	32887394	95.12

(1*R*,3*S*)-1'-tert-butyl 2,2-dimethyl-2'-oxo-3-propylspiro[cyclopropane-1,3'-indoline]-1',2,2-tricarboxylate (4a):

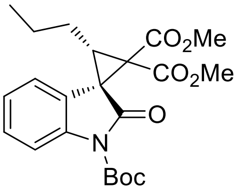

4a

Prepared according to the general procedure (48 h). The tittle compound **4a** was obtained as a colorless oil in 65% yield, >19:1 d.r., 98% *ee*. HPLC (Chiralcel IA, n-hexane/*i*-PrOH = 98/2, flow rate 1.0 mL/min, λ = 254 nm) t_r (major) = 6.58 min, t_r (minor) = 7.50 min. $[\alpha]^{13.0}_D$ = +718.1 (*c* = 0.65, in CH_2Cl_2). ^{1}H NMR (400 MHz, $CDCl_3$) δ 7.92 (d, *J* = 8.4 Hz, 1H), 7.47 (d, *J* = 8.0 Hz, 1H), 7.33 (t, *J* = 8.0 Hz, 1H), 7.13 (t, *J* = 7.6 Hz, 1H), 3.74 (d, *J* = 15.6 Hz, 6H), 2.67 (t, *J* = 7.4 Hz, 1H), 2.10 – 2.01 (m, 1H), 1.84 – 1.75 (m, 1H), 1.61 (s, 9H), 1.49 – 1.29 (m, 2H), 0.89 (t, *J* = 7.4 Hz, 3H). ^{13}C NMR (100 MHz, $CDCl_3$) δ = 171.74, 166.33, 164.90, 148.80, 140.68, 128.28, 125.07, 123.70, 121.86, 114.79, 84.55, 53.17, 52.95, 49.04, 41.13, 38.69, 28.07, 24.03, 21.90, 13.53. HRMS (ESI-TOF) calcd for $C_{22}H_{27}NO_7$ ([M]+Na$^+$) = 440.1685, Found 440.1686.

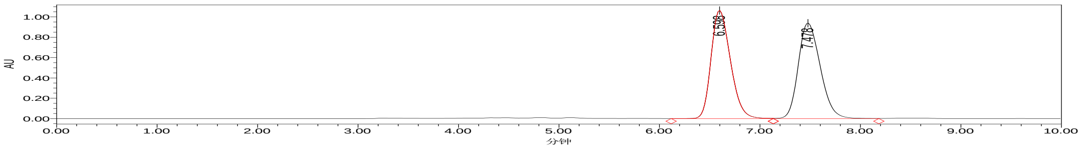

	Retention Time	Area	% Area
1	6.598	14265992	49.90
2	7.478	14325044	50.10

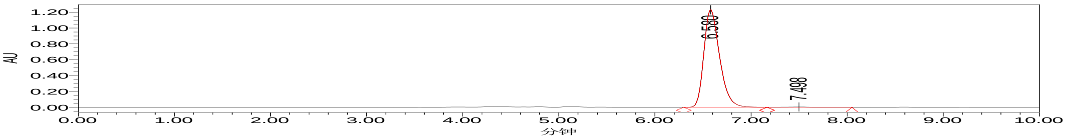

	Retention Time	Area	% Area
1	6.580	14008239	99.26
2	7.498	104444	0.74

(1*R*,3*S*)-1'-tert-butyl 2,2-dimethyl 3-cyclopentyl-2'-oxospiro[cyclopropane-1,3'-indoline]-1',2,2-tricarboxylate (4a'):

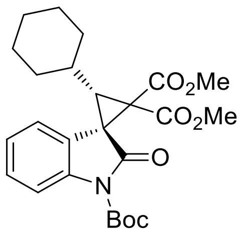

4a'

Prepared according to the general procedure (120 h). The tittle compound **4a'** was obtained as a colorless oil in 32% yield, >19:1 d.r., 97% *ee*. HPLC (Chiralcel IA, n-hexane/*i*-PrOH = 90/10, flow rate 1.0 mL/min, λ = 254 nm) t_r (major) = 3.92 min, t_r (minor) = 5.34 min. $[\alpha]^{18.1}_D$ = +27.9 (*c* = 0.24 in CH_2Cl_2). ^1H NMR (400 MHz, $CDCl_3$) δ 7.99 – 7.85 (m, 1H), 7.55 (dd, *J* = 7.6, 0.8 Hz, 1H), 7.37 – 7.30 (m, 1H), 7.14 (td, *J* = 7.6, 0.8 Hz, 1H), 3.76 (s, 3H), 3.72 (s, 3H), 2.50 (d, *J* = 11.2 Hz, 1H), 2.20 – 2.12 (m, 1H), 1.90 – 1.89 (m, 1H), 1.83 – 1.75 (m, 1H), 1.62 (s, 9H), 1.59 – 1.52 (m, 1H), 1.40 – 1.09 (m, 6H), 0.97 – 0.85 (m, 1H). ^{13}C NMR (100 MHz, $CDCl_3$) δ = 171.68, 166.35, 165.01, 148.81, 140.59, 128.25, 124.73, 123.79, 121.76, 114.78, 84.53, 53.15, 52.92, 49.05, 44.44, 41.06, 32.02, 31.80, 31.15, 28.08, 26.01, 25.73, 25.55. HRMS (ESI-TOF) calcd for $C_{25}H_{31}NO_7$ ($[M]+Na^+$) = 480.1998, Found 480.1999.

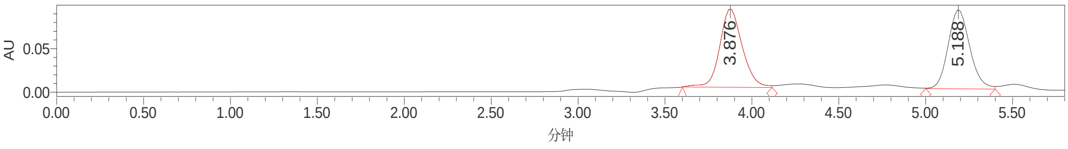

	Retention Time	Area	% Area
1	3.876	810007	50.83
2	5.188	783418	49.17

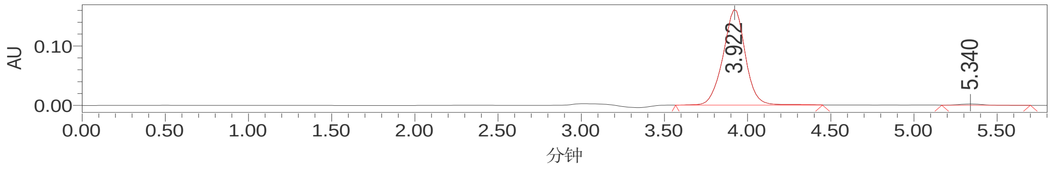

	Retention Time	Area	% Area

1	3.922	1507803	98.58
2	5.340	21701	1.42

(1*R*,3*S*)-1'-tert-butyl 2,2-dimethyl 3-cyano-2'-oxospiro[cyclopropane-1,3'-indoline] -1',2,2-tricarboxylate (4a'''):

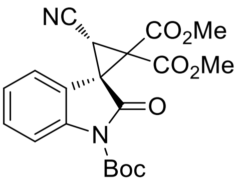

4a'''

Prepared according to the general procedure (120 h). The tittle compound **4a'''** was obtained as a colorless oil in 82% yield, >19:1 d.r., 85% *ee*. HPLC (Chiralcel ID, n-hexane/*i*-PrOH = 80/20, flow rate 1.0 mL/min, λ = 254 nm) t_r (major) = 3.92 min, t_r (minor) = 5.34 min. $[\alpha]^{18.1}_D$ = +59.5 (*c* = 0.20, in CH_2Cl_2). ^1H NMR (400 MHz, $CDCl_3$) δ 7.95 (d, *J* = 8.0 Hz, 1H), 7.58 (dd, *J* = 8.0, 0.8 Hz, 1H), 7.48 – 7.41 (m, 1H), 7.23 (td, *J* = 7.6, 0.8 Hz, 1H), 3.82 (d, *J* = 4.0 Hz, 6H), 3.33 (s, 1H), 1.62 (s, 10H). ^{13}C NMR (100 MHz, $CDCl_3$) δ = 168.59, 162.90, 161.88, 148.14, 141.06, 130.31, 124.79, 124.45, 118.41, 115.28, 112.26, 85.47, 54.07, 53.97, 47.30, 40.10, 28.01, 22.39. HRMS (ESI-TOF) calcd for $C_{20}H_{20}N_2O_7$ ([M]+Na^+) = 423.1168, Found 423.1162.

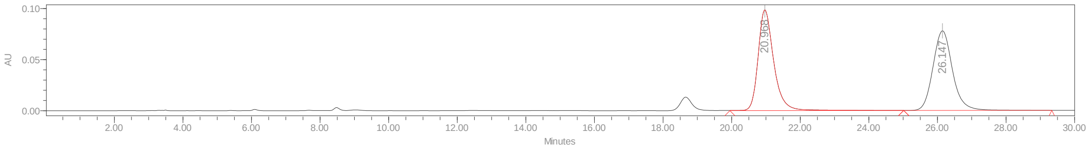

	Retention Time	Area	% Area
1	20.968	3043260	49.99
2	26.147	3044584	50.01

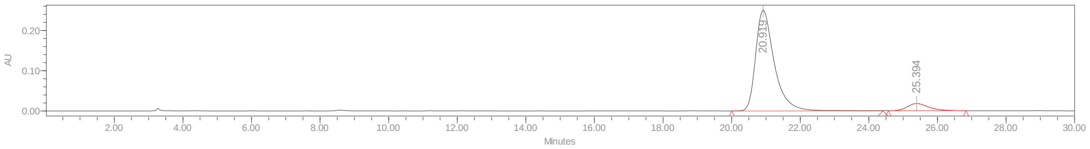

	Retention Time	Area	% Area
1	20.919	9464086	92.46
2	25.394	771266	7.54

(1*R*,3*S*)-1'-tert-butyl 2,2-dimethyl-2'-oxo-3-phenylspiro[cyclopropane-1,3'-indoline]-1',2,2-tricarboxylate (4b):

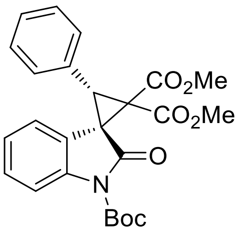

4b

Prepared according to the general procedure (48 h). The tittle compound **4b** was obtained as a white solid in 89% yield, >19:1 d.r., 99% *ee*. HPLC (Chiralcel IA, n-hexane/*i*-PrOH = 98/2, flow rate 1.0 mL/min, λ = 254 nm) t_r (major) = 10.77 min, t_r (minor) = 13.51 min. $[\alpha]^{20.6}_D$ = +77.4 (*c* = 0.70, in CH_2Cl_2). ^1H NMR (400 MHz, $CDCl_3$) δ 7.93 (d, *J* = 8.0 Hz, 1H), 7.30 (t, *J* = 7.6 Hz, 4H), 7.08 (d, *J* = 6.0 Hz, 2H), 6.93 (t, *J* = 8.0 Hz, 1H), 6.70 (d, *J* = 8.0 Hz, 1H), 4.06 (s, 1H), 3.83 (s, 3H), 3.69 (s, 3H), 1.65 (s, 9H). ^{13}C NMR (100 MHz, $CDCl_3$) δ = 171.47, 166.29, 164.30, 148.80, 140.84, 130.00, 129.78, 128.43, 128.22, 127.89, 122.99, 120.50, 114.37, 84.69, 53.32, 52.89, 50.06, 42.19, 40.07, 28.11. HRMS (ESI-TOF) calcd for $C_{25}H_{25}NO_7$ ([M]+K^+) = 474.1523,

Found 474.1527.

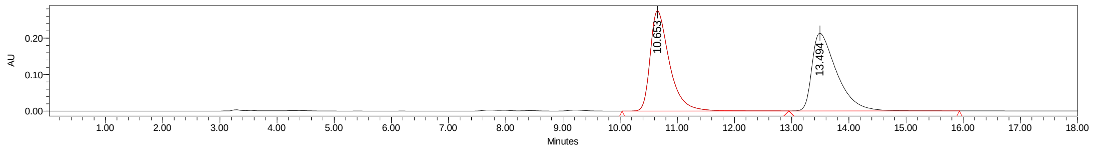

	Retention Time	Area	% Area
1	10.653	6414960	50.05
2	13.494	6402517	49.95

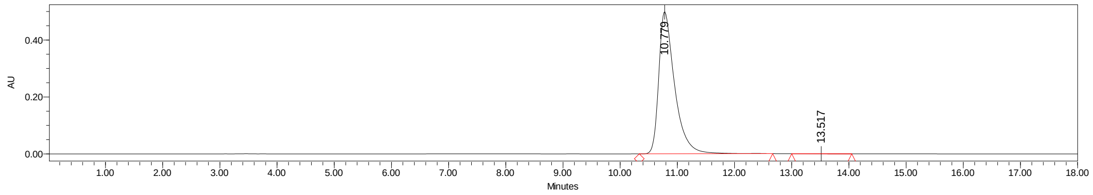

	Retention Time	Area	% Area
1	10.779	9618135	99.99
2	13.517	700	0.01

(1*R*,3*S*)-1'-tert-butyl-2,2-dimethyl-2'-oxo-3-(m-tolyl)spiro[cyclopropane-1,3'-indoline]-1',2,2-tricarboxylate (4c):

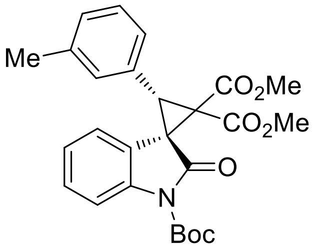

4c

Prepared according to the general procedure (48 h). The tittle compound **4c** was obtained as a white solid in 92% yield, >19:1 d.r., 99% *ee*. HPLC (Chiralcel IA, n-hexane/ *i*-PrOH = 98/2, flow rate 1.0 mL/min, λ = 254 nm) t_r (major) = 8.15 min, t_r (minor) = 9.97 min. $[\alpha]^{25.9}_D$ = +75.8 (*c* = 0.33, in CH_2Cl_2). ^1H NMR (400 MHz, $CDCl_3$) δ 7.93 (d, *J* = 8.4 Hz, 1H), 7.34 – 7.27 (m, 1H), 7.17 (t, *J* = 7.6 Hz, 1H), 7.11 (d, *J* = 7.6 Hz, 1H), 6.97 – 6.88 (m, 2H), 6.85 (d, *J* = 7.2 Hz, 1H), 6.73 (d, *J* = 8.0 Hz, 1H), 4.03 (s, 1H), 3.83 (s, 3H), 3.69 (s, 3H), 2.27 (s, 3H), 1.65 (s, 9H). ^{13}C NMR (100 MHz, $CDCl_3$) δ = 171.52, 166.39, 164.35, 148.83, 140.82, 137.88, 130.62, 129.64, 128.63, 128.40, 128.10, 128.03, 127.00, 122.89, 120.53, 114.33, 84.66, 53.32, 52.84, 50.08, 42.18, 40.07, 28.11, 21.33. HRMS (ESI-TOF) calcd for $C_{26}H_{27}NO_7$ ($[M]+K^+$) = 504.1419, Found 504.1424.

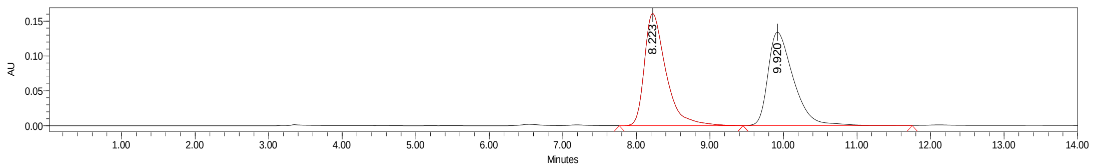

	Retention Time	Area	% Area
1	8.233	3276842	50.48
2	9.920	3214866	49.52

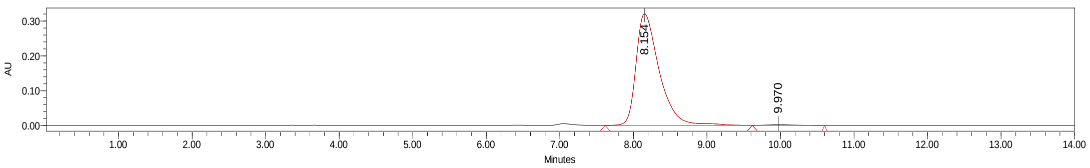

	Retention Time	Area	% Area
1	8.154	7027919	99.36
2	9.970	45419	0.64

(1*R*,3*S*)-1'-(tert-butyl) 2,2-dimethyl 2'-oxo-3-(p-tolyl)spiro[cyclopropane-1,3'-indoline]-1',2,2-tricarboxylate (4d):

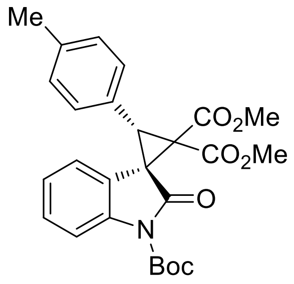

4d

Prepared according to the general procedure (48 h). The tittle compound **4d** was obtained as a white solid in 70% yield, >19:1 d.r., 98% *ee*. HPLC (Chiralcel IA, n-hexane/ *i*-PrOH = 98/2, flow rate 1.0 mL/min, λ = 254 nm) t_r (major) = 14.49 min, t_r (minor) = 17.84 min. $[\alpha]^{20.8}_D$ = +56.3 (*c* = 0.57, in CH_2Cl_2). ^1H NMR (400 MHz, $CDCl_3$) δ 7.92 (d, *J* = 8.4 Hz, 1H), 7.34 – 7.28 (m, 1H), 7.09 (d, *J* = 8.0 Hz, 2H), 6.96 – 6.92 (m, 3H), 6.73 (dd, *J* = 7.6, 0.8 Hz, 1H), 4.01 (s, 1H), 3.83 (s, 3H), 3.69 (s, 3H), 2.34 (s, 3H), 1.65 (s, 9H). ^{13}C NMR (101 MHz, $CDCl_3$) δ = 171.56, 166.39, 164.36, 148.82, 140.79, 137.64, 129.84, 128.96, 128.37, 127.97, 126.58, 122.96, 120.57, 114.33, 84.68, 53.33, 52.91, 50.10, 42.20, 39.95, 28.11, 21.25. HRMS (ESI-TOF) calcd for $C_{26}H_{27}NO_7$ ($[M]+Na^+$) = 488.1680, Found 488.1687.

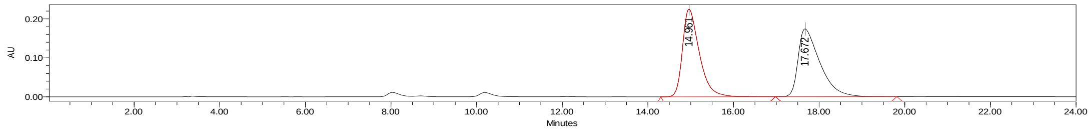

	Retention Time	Area	% Area
1	14.961	6143863	49.91
2	17.672	6165689	50.09

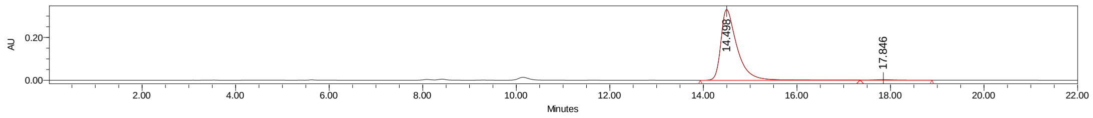

	Retention Time	Area	% Area
1	14.498	7921517	99.10
2	17.846	71969	0.90

(1*R*,3*S*)-1'-(tert-butyl) 2,2-dimethyl 2'-oxo-3-(3-phenoxyphenyl) spiro[cyclopropane-1,3' -indoline] -1',2,2- tricarboxylate (4e):

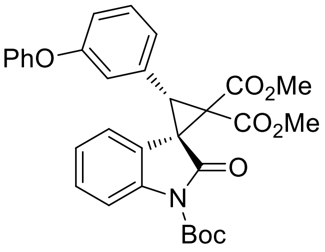

4e

Prepared according to the general procedure (48 h). The tittle compound **4e** was obtained as a white solid in 77% yield, >19:1 d.r., 99% *ee*. HPLC (Chiralcel ID, n-hexane/ *i*-PrOH = 80/20, flow rate 1.0 mL/min, λ = 254 nm) t_r (major) = 15.26 min, t_r (minor) = 9.67 min. $[\alpha]^{20.7}_D$ = +29.0 (*c* = 1.47, in CH_2Cl_2). ^1H NMR (400 MHz, $CDCl_3$) δ

7.91 (d, J = 8.0 Hz, 1H), 7.35 – 7.26 (m, 3H), 7.25 (d, J = 5.2 Hz, 1H), 7.05 (t, J = 7.2 Hz, 1H), 7.01 – 6.90 (m, 4H), 6.81 (dd, J = 12.4, 7.6 Hz, 2H), 6.70 (s, 1H), 4.01 (s, 1H), 3.81 (s, 3H), 3.62 (s, 3H), 1.64 (s, 9H). ^{13}C NMR (100 MHz, $CDCl_3$) δ = 171.33, 166.16, 164.16, 156.98, 156.95, 148.74, 140.84, 131.67, 129.72, 129.67, 128.50, 127.83, 124.95, 123.30, 123.09, 120.40, 120.24, 118.77, 114.43, 84.72, 53.34, 52.92, 49.84, 42.21, 39.84, 28.10. HRMS (ESI-TOF) calcd for $C_{31}H_{29}NO_8$ ($[M]+Na^+$) = 566.1791, Found 566.1797.

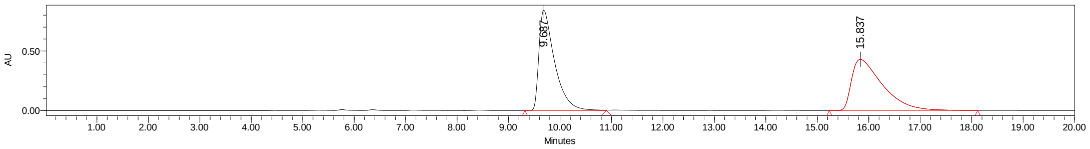

	Retention Time	Area	% Area
1	9.687	17833250	49.99
2	15.837	17843482	50.01

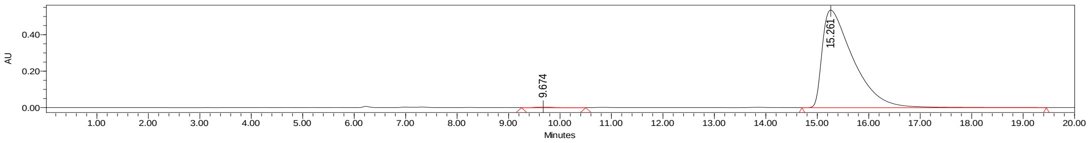

	Retention Time	Area	% Area
1	9.674	66469	0.30
2	15.261	22362313	99.70

(1*R*,3*S*)-1'-(tert-butyl) 2,2-dimethyl-3-(2-methoxyphenyl) 2'-oxospiro[cyclopropane-1,3'-indoline] -1',2,2- tricarboxylate (4f):

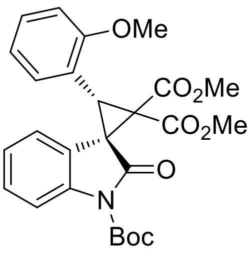

4f

Prepared according to the general procedure (48 h). The tittle compound **4f** was obtained as a white solid in 65% yield, >19:1 d.r., 99% *ee*. HPLC (Chiralcel IA, n-hexane/ *i*-PrOH = 98/2, flow rate 1.0 mL/min, λ = 254 nm) t_r (major) = 15.15 min, t_r (minor) = 17.17 min. $[\alpha]^{29.7}_D$ = +94.5 (c = 0.27, in CH_2Cl_2). ^{1}H NMR (400 MHz, $CDCl_3$) δ 7.90 (d, J = 8.0 Hz, 1H), 7.32 – 7.28 (m, 1H), 7.26 – 7.24 (m, 1H), 7.00 – 6.94 (m, 1H), 6.91 – 6.84 (m, 2H), 6.80 (d, J = 8.4 Hz, 1H), 6.71 (dd, J = 8.0, 0.8 Hz, 1H), 3.82 (s, 3H), 3.81 (s, 1H), 3.69 (s, 3H), 3.48 (s, 3H), 1.65 (s, 9H). ^{13}C NMR (100 MHz, $CDCl_3$) δ = 171.73, 166.53, 164.85, 158.12, 148.93, 140.64, 130.82, 129.40, 128.05, 127.09, 122.89, 121.51, 120.00, 118.27, 114.12, 110.66, 84.52, 55.01, 53.16, 52.70, 49.85, 42.47, 36.94, 28.12. HRMS (ESI-TOF) calcd for $C_{26}H_{27}NO_8$ ($[M]+K^+$) = 504.1629, Found 504.1636.

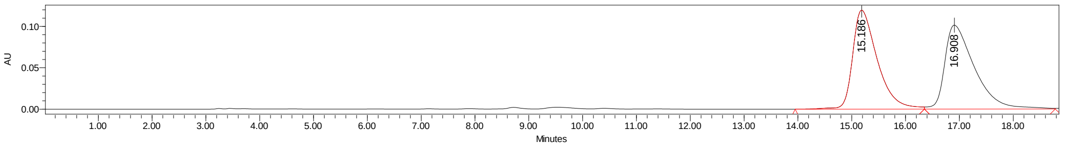

	Retention Time	Area	% Area
1	15.186	3813467	49.49
2	16.908	3892219	50.51

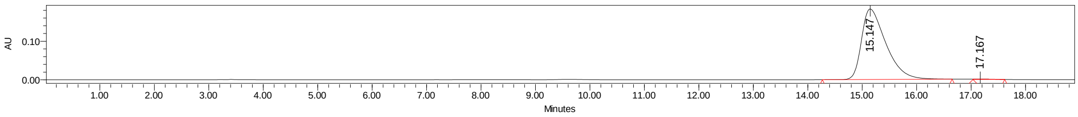

	Retention Time	Area	% Area
1	15.147	5650157	99.58
2	17.167	23815	0.42

(1*R*,3*S*)-1'-(tert-butyl) 2,2-dimethyl-3-(3-methoxyphenyl) 2'-oxospiro[cyclopropane-1,3'-indoline] -1',2,2- tricarboxylate (4g):

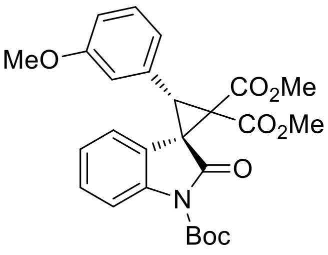

4g

Prepared according to the general procedure (48 h). The tittle compound **4g** was obtained as a white solid in 93% yield, >19:1 d.r., 99% *ee*. HPLC (Chiralcel IA, n-hexane/ *i*-PrOH = 98/2, flow rate 1.0 mL/min, λ = 254 nm) t_r (major) = 26.22 min, t_r (minor) = 18.25 min. $[\alpha]^{29.7}_D$ = +78.6 (*c* = 0.48, in CH_2Cl_2). ^{1}H NMR (400 MHz, $CDCl_3$) δ 7.92 (d, *J* = 8.0 Hz, 1H), 7.33 – 7.28 (m, 1H), 7.20 (t, *J* = 8.0 Hz, 1H), 6.97 – 6.92 (m, 1H), 6.84 (dd, *J* = 8.4, 2.4 Hz, 1H), 6.77 (d, *J* = 8.0 Hz, 1H), 6.66 (d, *J* = 7.6 Hz, 1H), 6.61 (s, 1H), 4.03 (s, 1H), 3.83 (s, 3H), 3.69 (d, *J* = 3.6 Hz, 6H), 1.65 (s, 9H). ^{13}C NMR (100 MHz, $CDCl_3$) δ = 171.47, 166.27, 164.26, 159.33, 148.80, 140.81, 131.21, 129.29, 128.48, 128.01, 122.95, 122.25, 120.42, 115.40, 114.35, 113.78, 84.71, 55.19, 53.34, 52.92, 50.06, 42.16, 40.06, 28.10. HRMS (ESI-TOF) calcd for $C_{26}H_{27}NO_8$ ($[M]+K^+$) = 504.1629, Found 504.1632.

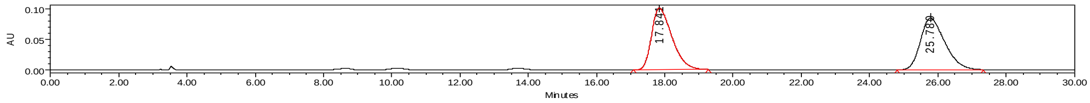

	Retention Time	Area	% Area
1	17.841	4200300	49.94
2	25.780	4209856	50.06

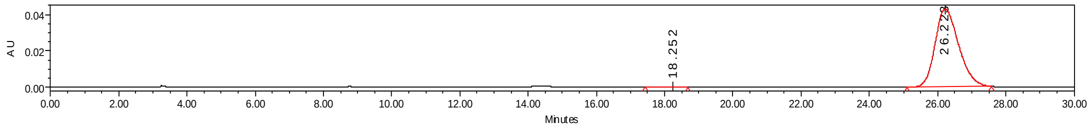

	Retention Time	Area	% Area
1	18.252	4920	0.24
2	26.223	2057510	99.76

(1*R*,3*S*)-1'-(tert-butyl) 2,2-dimethyl-3-(2,3-dimethoxyphenyl) 2'-oxospiro[cyclopropane -1,3'-indoline] -1',2,2- tricarboxylate (4h):

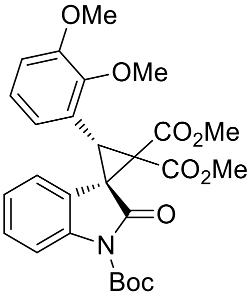

4h

Prepared according to the general procedure (48 h). The tittle compound **4h** was obtained as a white solid in 71% yield, >19:1 d.r., 94% *ee*. HPLC (Chiralcel IA, n-hexane/ *i*-PrOH = 98/2, flow rate 1.0 mL/min, λ = 254 nm) t_r (major) = 14.49 min, t_r (minor) = 16.21 min. $[\alpha]^{29.0}_D$ = +80.4 (*c* = 0.49, in CH_2Cl_2). ^{1}H NMR (400 MHz, $CDCl_3$) δ 7.92 (d, *J* = 8.0 Hz, 1H), 7.43 – 7.27 (m, 1H), 7.02 – 6.83 (m, 4H), 6.47 – 6.45 (m, 1H), 3.87 (d, *J* = 2.0 Hz, 1H),

3.84 (s, 3H), 3.81 (s, 3H), 3.76 (s, 3H), 3.67 (s, 3H), 1.63 (s, 9H). ^{13}C NMR (100 MHz, $CDCl_3$) δ = 171.81, 166.66, 164.63, 152.85, 148.85, 148.41, 140.86, 128.33, 128.03, 123.90, 123.25, 123.08, 122.85, 120.87, 114.22, 112.58, 84.56, 60.50, 55.84, 53.08, 52.87, 50.11, 42.19, 36.49, 28.11. HRMS (ESI-TOF) calcd for $C_{27}H_{29}NO_8$ ([M]+K^+) = 550.1474, Found 550.1478.

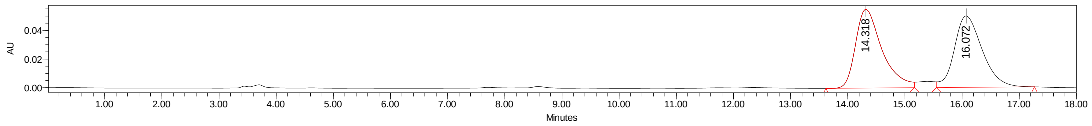

	Retention Time	Area	% Area
1	14.318	1785690	49.81
2	16.072	1799027	50.19

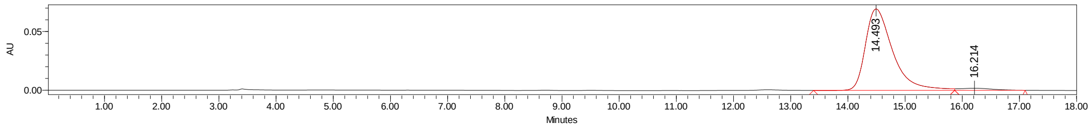

	Retention Time	Area	% Area
1	14.493	2301789	97.02
2	16.214	70635	2.98

(1*R*,3*S*)-1'-(tert-butyl) 2,2-dimethyl 3-(3-fluorophenyl)-2'-oxospiro[cyclopropane-1,3'-indoline]-1',2,2- tricarboxylate (4i):

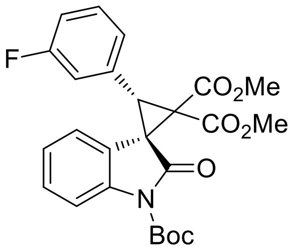

4i

Prepared according to the general procedure (48 h). The tittle compound **4i** was obtained as a white solid in 65% yield, >19:1 d.r., 98% *ee*. HPLC (Chiralcel IA, n-hexane/ *i*-PrOH = 98/2, flow rate 1.0 mL/min, λ = 254 nm) t_r (major) = 9.25 min, t_r (minor) = 11.51 min. $[\alpha]^{23.6}_D$ = +84.6 (*c* = 0.85, in CH_2Cl_2). ^{1}H NMR (400 MHz, $CDCl_3$) δ 7.94 (d, *J* = 8.4 Hz, 1H), 7.36 – 7.29 (m, 1H), 7.26 – 7.12 (m, 1H), 7.04 – 6.93 (m, 2H), 6.89 – 6.79 (m, 2H), 6.75 (d, *J* = 7.6 Hz, 1H), 4.01 (s, 1H), 3.83 (s, 3H), 3.69 (s, 3H), 1.65 (s, 9H). ^{13}C NMR (100 MHz, $CDCl_3$) δ = 171.23, 166.02, 164.04, 162.43 (d, *J* = 245), 148.72, 140.90, 132.23 (d, *J* = 8), 129.77 (d, *J* = 8), 128.69, 127.58, 125.80, 125.77, 123.20, 120.10, 117.05 (d, *J* = 22), 114.98 (d, *J* = 21), 114.53, 84.81, 53.38, 53.01, 49.92, 42.07, 39.34, 28.09. HRMS (ESI-TOF) calcd for $C_{25}H_{24}FNO_7$ ([M]+Na^+) = 492.1429, Found 492.1430.

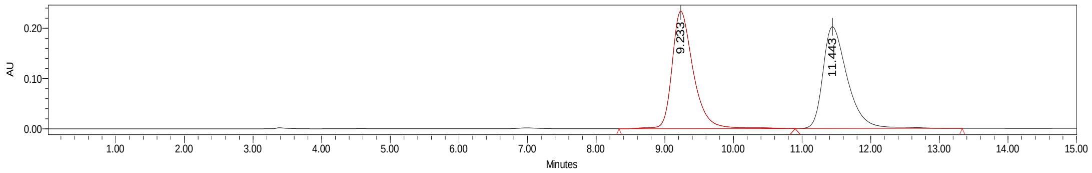

	Retention Time	Area	% Area
1	9.233	5026555	50.17
2	11.443	4993238	49.83

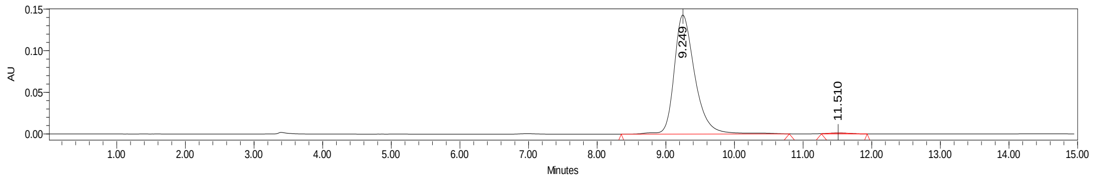

	Retention Time	Area	% Area
1	9.249	3014359	99.18
2	11.510	24788	0.82

(1*R*,3*S*)-1'-(tert-butyl) 2,2-dimethyl 3-(4-fluorophenyl)-2'-oxospiro[cyclopropane-1,3'-indoline]-1',2,2- tricarboxylate (4j):

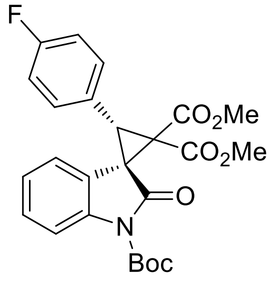

4j

Prepared according to the general procedure (48 h). The tittle compound **4j** was obtained as a white solid in 89% yield, >19:1 d.r., 99% *ee*. HPLC (Chiralcel IA, n-hexane/ *i*-PrOH = 98/2, flow rate 1.0 mL/min, λ = 254 nm) t_r (major) = 10.90 min, t_r (minor) = 14.70 min. $[\alpha]^{25.9}_D$ = +81.6 (*c* = 0.94, in CH_2Cl_2). ^{1}H NMR (400 MHz, $CDCl_3$) δ 7.93 (d, *J* = 8.0 Hz, 1H), 7.34 – 7.29 (m, 1H), 7.05 (dd, *J* = 8.0, 5.6 Hz, 2H), 7.00 – 6.93 (m, 3H), 6.72 – 6.66 (m, 1H), 3.98 (s, 1H), 3.83 (s, 3H), 3.68 (s, 3H), 1.65 (s, 9H). ^{13}C NMR (100 MHz, $CDCl_3$) δ = 171.31, 166.11, 164.14, 162.32 (d, *J* = 245), 148.74, 140.88, 131.78 (d, *J* = 8), 128.60, 127.57, 125.50 (d, *J* = 3), 123.13, 120.30, 115.28 (d, *J* = 22), 114.51, 84.79, 53.38, 52.96, 50.07, 42.11, 39.25, 28.09. HRMS (ESI-TOF) calcd for $C_{25}H_{24}FNO_7$ ($[M]+Na^+$) = 492.1429, Found 492.1438.

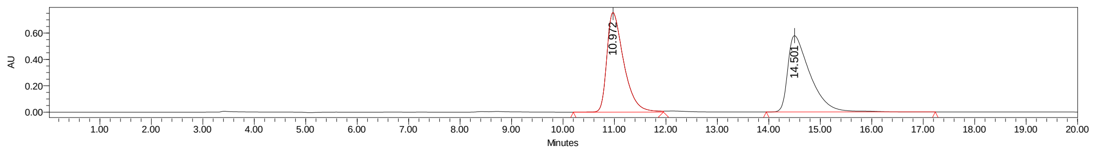

	Retention Time	Area	% Area
1	10.972	17198702	49.69
2	14.501	17415296	50.31

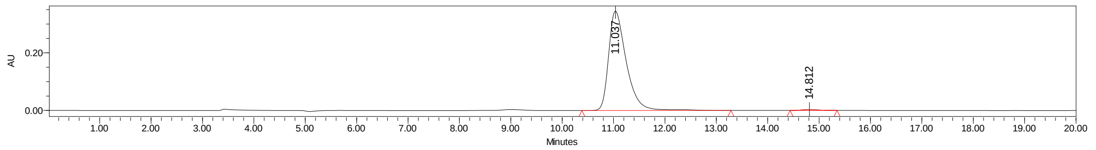

	Retention Time	Area	% Area
1	10.904	14346867	99.53
2	14.697	68005	0.47

(1*R*,3*S*)-1'-tert-butyl 2,2-dimethyl-3-(3-chlorophenyl)-2'-oxospiro[cyclopropane-1,3'-indoline]-1',2,2-tricarboxylate (4k):

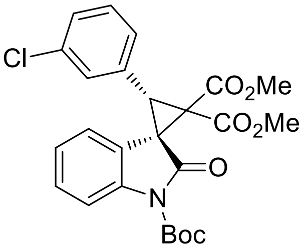

4k

Prepared according to the general procedure (48 h). The tittle compound **4k** was obtained as a white solid in 65% yield, >19:1 d.r., 98% *ee*.

HPLC (Chiralcel IA, n-hexane/ *i*-PrOH = 98/2, flow rate 1.0 mL/min, λ = 254 nm) t_r (major) = 8.35 min, t_r (minor) = 10.76 min. $[\alpha]^{23.8}_D$ = +71.1 (*c* = 1.07, in CH_2Cl_2). ^{1}H NMR (400 MHz, $CDCl_3$) δ 7.94 (d, *J* = 8.4 Hz, 1H), 7.32 (dd, *J* = 17.2, 8.8 Hz, 2H), 7.22 (t, *J* = 7.8 Hz, 1H), 7.11 (s, 1H), 6.96 (dd, *J* = 13.6, 7.2 Hz, 2H), 6.74 (d, *J* = 8.0 Hz, 1H), 3.99 (s, 1H), 3.83 (s, 3H), 3.69 (s, 3H), 1.65 (s, 9H). ^{13}C NMR (100 MHz, $CDCl_3$) δ = 171.19, 165.98, 164.01, 148.71, 140.91, 134.05, 131.84, 130.10, 129.46, 128.73, 128.30, 128.14, 127.55, 123.21, 120.07, 114.57, 84.82, 53.41, 53.02, 49.88, 41.98, 39.20, 28.09. HRMS (ESI-TOF) calcd for $C_{25}H_{24}{}^{34.9689}ClNO_7$ ($[M]+Na^+$) = 508.1134, Found 508.1143; $C_{21}H_{17}{}^{36.9659}ClNO$ ($[M]+Na^+$) = 510.1104, Found 510.1124.

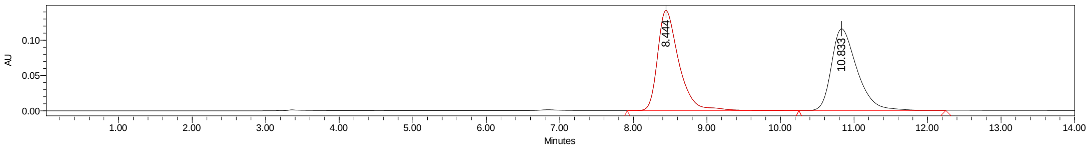

	Retention Time	Area	% Area
1	8.444	2834382	50.35
2	10.833	2795378	49.65

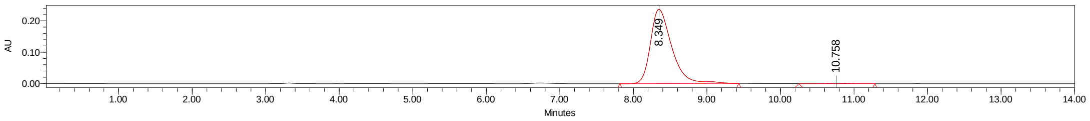

	Retention Time	Area	% Area
1	8.349	4754608	98.98
2	10.758	48780	1.02

(1*R*,3*S*)-1'-tert-butyl 2,2-dimethyl-3-(4-chlorophenyl)-2'-oxospiro[cyclopropane-1,3'-indoline]-1',2,2-tricarboxylate (4l):

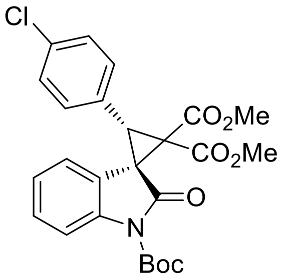

4l

Prepared according to the general procedure (48 h). The tittle compound **4l** was obtained as a white solid in 85% yield, >19:1 d.r., 99% *ee*. HPLC (Chiralcel IA, n-hexane/ *i*-PrOH = 98/2, flow rate 1.0 mL/min, λ = 254 nm) t_r (major) = 12.22 min, t_r (minor) = 16.04 min. $[\alpha]^{25.7}_D$ = +58.2 (*c* = 0.84, in CH_2Cl_2). ^{1}H NMR (400 MHz, $CDCl_3$) δ 7.94 (d, *J* = 8.0 Hz, 1H), 7.35 – 7.30 (m, 1H), 7.27 (d, *J* = 6.0 Hz, 2H), 7.02 (d, *J* = 8.0 Hz, 2H), 6.97 (t, *J* = 7.6 Hz, 1H), 6.73 (d, *J* = 7.6 Hz, 1H), 3.98 (s, 1H), 3.83 (s, 3H), 3.68 (s, 3H), 1.65 (s, 9H). ^{13}C NMR (100 MHz, $CDCl_3$) δ = 171.24, 166.04, 164.06, 148.72, 140.90, 133.91, 131.44, 128.67, 128.47, 128.31, 127.52, 123.21, 120.20, 114.55, 84.82, 53.39, 53.00, 49.99, 42.02, 39.23, 28.09. HRMS (ESI-TOF) calcd for $C_{25}H_{24}{}^{34.9689}ClNO_7$ ($[M]+Na^+$) = 508.1134, Found 508.1132; $C_{21}H_{17}{}^{36.9659}ClNO$ ($[M]+Na^+$) = 510.1104, Found 510.1121.

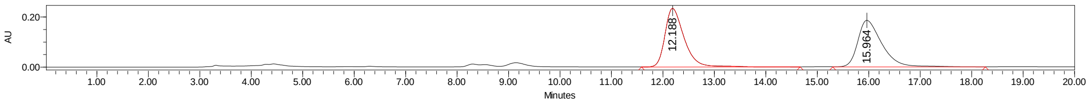

	Retention Time	Area	% Area
1	12.188	6079581	49.94

2	15.964	6093290	50.06

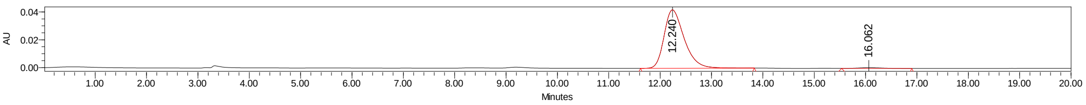

	Retention Time	Area	% Area
1	12.223	5026372	99.50
2	16.036	25109	0.50

(1*R*,3*S*)-1'-(tert-butyl) 2,2-dimethyl 3-(3-bromophenyl)-2'-oxospiro[cyclopropane-1,3'-indoline]-1',2,2- tricarboxylate (4m):

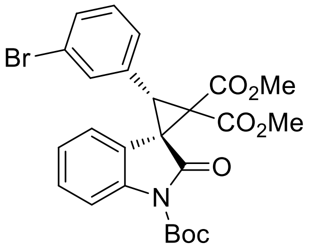

4m

Prepared according to the general procedure (48 h). The tittle compound **4m** was obtained as a white solid in 90% yield, >19:1 d.r. (96:4 d.r.), 98% *ee*. HPLC (Chiralcel IA, n-hexane/ *i*-PrOH = 98/2, flow rate 1.0 mL/min, λ = 254 nm) t_r (major) = 8.79 min, t_r (minor) = 11.05 min. $[\alpha]^{25.9}_D$ = +72.8 (*c* = 0.32, in CH_2Cl_2). ^1H NMR (400 MHz, $CDCl_3$) δ 7.94 (d, *J* = 8.0 Hz, 1H), 7.44 (d, *J* = 8.0 Hz, 1H), 7.33 (t, *J* = 7.6 Hz, 1H), 7.27 (s, 1H), 7.16 (t, *J* = 8.0 Hz, 1H), 6.97 (t, *J* = 8.0 Hz, 2H), 6.74 (d, *J* = 7.6 Hz, 1H), 3.99 (s, 1H), 3.83 (s, 3H), 3.69 (s, 3H), 1.65 (s, 9H). ^{13}C NMR (100 MHz, $CDCl_3$) δ = 171.17, 165.97, 163.99, 148.71, 140.91, 132.99, 132.10, 131.05, 129.72, 128.79, 128.74, 127.56, 123.21, 122.10, 120.05, 114.57, 84.82, 53.41, 53.02, 49.87, 41.95, 39.10, 28.09. HRMS (ESI-TOF) calcd for $C_{25}H_{24}{}^{78.9183}BrNO_7$ ($[M]+Na^+$) = 552.0628, Found 552.0633; $C_{25}H_{24}{}^{80.9163}BrNO_7$ ($[M]+Na^+$) = 554.0615, Found 554.0608.

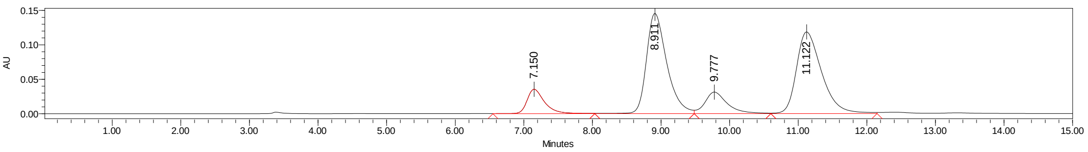

	Retention Time	Area	% Area
1	7.150	634797	8.61
2	8.911	2996484	40.67
3	9.777	709897	9.63
4	11.122	3027361	41.08

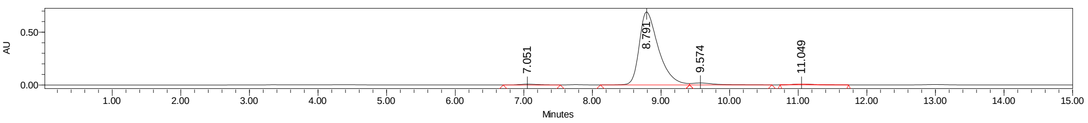

	Retention Time	Area	% Area
1	7.051	143596	1.00
2	8.791	13481271	94.29
3	9.574	464046	3.25
4	11.049	208695	1.46

(1*R*,3*S*)-1'-(tert-butyl) 2,2-dimethyl 3-(4-bromophenyl)-2'-oxospiro[cyclopropane-1,3'-indoline] -1',2,2- tricarboxylate (4n):

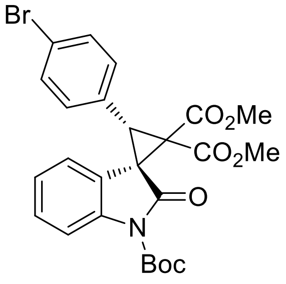

4n

Prepared according to the general procedure (48 h). The tittle compound **4n** was obtained as a white solid in 78% yield, >19:1 d.r., 98% *ee*. HPLC (Chiralcel IA, n-hexane/ *i*-PrOH = 98/2, flow rate 1.0 mL/min, λ = 254 nm) t_r (major) = 12.94 min, t_r (minor) = 16.84 min. $[\alpha]^{25.9}_D$ = +47.6 (*c* = 0.33, in CH_2Cl_2). ^1H NMR (400 MHz, $CDCl_3$) δ 7.93 (d, *J* = 8.4 Hz, 1H), 7.42 (d, *J* = 8.4 Hz, 2H), 7.35 – 7.30 (m, 1H), 6.97 (t, *J* = 7.2 Hz, 3H), 6.73 (d, *J* = 7.6 Hz, 1H), 3.95 (s, 1H), 3.82 (s, 3H), 3.67 (s, 3H), 1.65 (s, 9H). ^{13}C NMR (100 MHz, $CDCl_3$) δ = 171.23, 166.02, 164.04, 148.71, 140.90, 131.76, 131.41, 128.84, 128.68, 127.51, 123.23, 122.10, 120.19, 114.55, 84.82, 53.39, 53.00, 49.96, 41.98, 39.27, 28.09. HRMS (ESI-TOF) calcd for $C_{25}H_{24}{}^{78.9183}BrNO_7$ ($[M]+Na^+$) = 552.0628, Found 552.0639; $C_{25}H_{24}{}^{80.9163}BrNO_7$ ($[M]+Na^+$) = 554.0615, Found 554.0616.

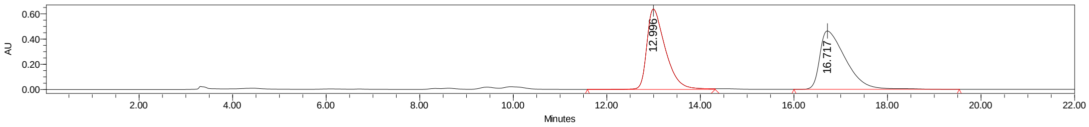

	Retention Time	Area	% Area
1	12.996	17484924	49.69
2	16.717	17703328	50.31

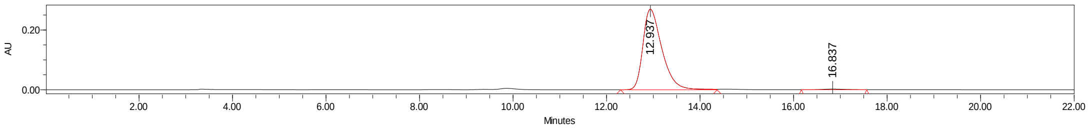

	Retention Time	Area	% Area
1	12.937	7610017	99.13
2	16.837	66444	0.87

(1*R*,3*S*)-1'-(tert-butyl) 2,2-dimethyl 3-(benzo[*d*][1,3]dioxol-5-yl) 2'-oxospiro[cyclopropane -1,3'-indoline] -1',2,2- tricarboxylate (4o):

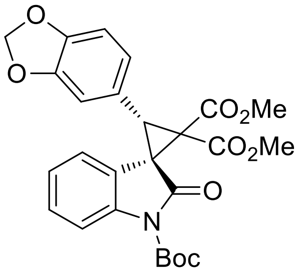

4o

Prepared according to the general procedure (48 h). The tittle compound **4o** was obtained as a white solid in 70% yield, 19:1 d.r., 98% *ee*. HPLC (Chiralcel IA, n-hexane/ *i*-PrOH = 98/2, flow rate 1.0 mL/min, λ = 254 nm) t_r (major) = 45.86 min, t_r (minor) = 27.67 min. $[\alpha]^{24.4}_D$ = +35.1 (*c* = 1.08, in CH_2Cl_2). ^1H NMR (400 MHz, $CDCl_3$) δ 7.92 (d, *J* = 8.4 Hz, 1H), 7.32 (dd, *J* = 16.0, 7.6 Hz, 1H), 6.97 (t, *J* = 7.6 Hz, 1H), 6.81 (d, *J* = 8.0 Hz, 1H), 6.70 (d, *J* = 8.0 Hz, 1H), 6.58 – 6.47 (m, 2H), 5.94 – 5.91 (m, 2H), 3.95 (s, 1H), 3.81 (s, 3H), 3.69 (s, 3H), 1.64 (s, 9H). ^{13}C NMR (100 MHz, $CDCl_3$) δ = 171.40, 166.22, 164.21, 148.77, 147.49, 147.25, 140.82, 128.47, 127.88, 123.48, 123.19, 123.05, 122.30, 120.37, 114.78, 114.39, 110.88, 110.29, 108.10, 101.18, 84.70, 53.32, 52.93, 50.20, 42.28, 39.87, 28.09. HRMS (ESI-TOF) calcd for $C_{26}H_{25}NO_9$ ($[M]+Na^+$) = 518.1422, Found 518.1431.

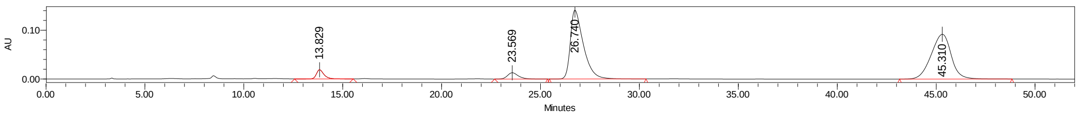

	Retention Time	Area	% Area
1	13.829	522765	3.75
2	23.569	499236	3.58
3	26.740	6460346	46.36
4	45.310	6453426	46.31

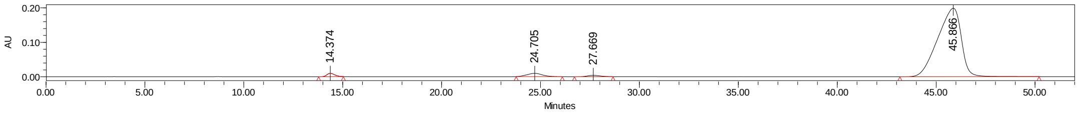

	Retention Time	Area	% Area
1	14.374	274251	1.57
2	24.705	528955	3.03
3	27.669	176069	1.01
4	45.866	16455121	94.38

(1*R*,3*S*)-1'-(tert-butyl) 2,2-dimethyl 3-(benzo[*d*][1,3]dioxol-5-yl) 2'-oxospiro[cyclopropane-1,3'-indoline] -1',2,2- tricarboxylate (4p):

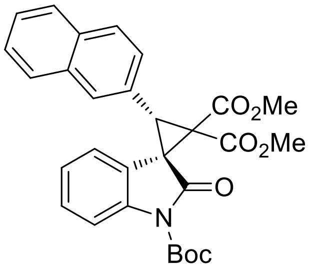

4p

Prepared according to the general procedure (48 h). The tittle compound **4p** was obtained as a white solid in 74% yield, >19:1 d.r., 97% *ee*. HPLC (Chiralcel IA, n-hexane/ *i*-PrOH = 98/2, flow rate 1.0 mL/min, λ = 254 nm) t_r (major) = 13.93 min, t_r (minor) = 17.81 min. $[\alpha]^{24.7}_D$ = +30.6 (*c* = 1.36, in CH_2Cl_2). ^{1}H NMR (400 MHz, $CDCl_3$) δ 7.96 (d, *J* = 8.4 Hz, 1H), 7.87 – 7.80 (m, 1H), 7.80 – 7.75 (m, 1H), 7.71 – 7.65 (m, 1H), 7.55 (s, 1H), 7.52 – 7.41 (m, 2H), 7.34 – 7.28 (m, 1H), 7.20 (dd, *J* = 8.4, 1.6 Hz, 1H), 6.87 (td, *J* = 7.6, 1.0 Hz, 1H), 6.69 (dd, *J* = 7.9, 1.0 Hz, 1H), 4.35 (s, 0.05H), 4.19 (s, 1H), 3.86 (s, 3H), 3.81 (s, 0.05H), 3.69 (s, 3H), 3.68 (s, 0.15H), 1.67 (s, 9H), 1.62 (s, 0.45H). ^{13}C NMR (100 MHz, $CDCl_3$) δ = 171.51, 166.34, 164.28, 148.84, 140.87, 132.94, 132.74, 129.11, 128.52, 127.96, 127.81, 127.76, 127.68, 127.31, 126.35, 126.27, 123.09, 120.52, 114.46, 84.76, 53.43, 52.94, 50.20, 42.18, 40.13, 28.13. HRMS (ESI-TOF) calcd for $C_{26}H_{25}NO_9$ ([M]+Na^+) = 524.1685, Found 524.1681.

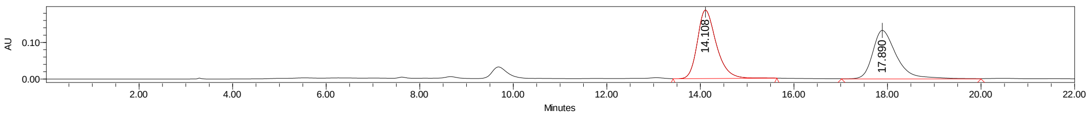

	Retention Time	Area	% Area
1	14.108	5018939	51.48
2	17.890	4729978	48.52

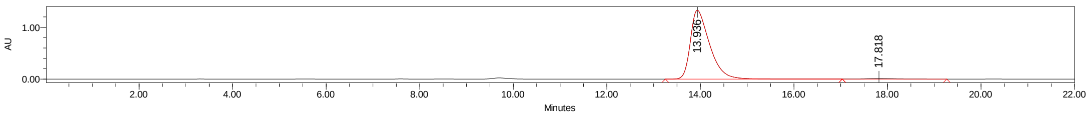

	Retention Time	Area	% Area
1	13.936	38004628	98.40
2	17.818	617656	1.60

(1*R*,3*S*)-1'-(tert-butyl) 2,2-dimethyl 6'-chloro-2'-oxo-3-phenylspiro[cyclopropane-1,3'-indoline]-1',2,2- tricarboxylate (4q):

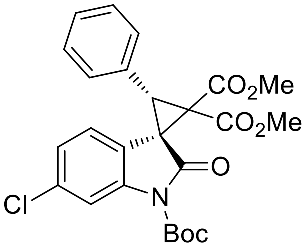

4q

Prepared according to the general procedure (48 h). The tittle compound **4q** was obtained as a white solid in 80% yield, >19:1 d.r., 98% *ee*. HPLC (Chiralcel IA, n-hexane/ *i*-PrOH = 98/2, flow rate 1.0 mL/min, λ = 254 nm) t_r (major) = 10.81 min, t_r (minor) = 9.03 min. $[\alpha]^{13.5}_D$ = +41.2 (*c* = 0.92, in CH_2Cl_2). ^1H NMR (400 MHz, $CDCl_3$) δ 8.01 (d, *J* = 2.0 Hz, 1H), 7.35 – 7.27 (m, 3H), 7.04 (dd, *J* = 6.8, 1.4 Hz, 2H), 6.91 (dd, *J* = 8.4, 2.0 Hz, 1H), 6.63 (d, *J* = 8.4 Hz, 1H), 4.05 (s, 1H), 3.83 (s, 3H), 3.70 (s, 3H), 1.65 (s, 9H). ^{13}C NMR (100 MHz, $CDCl_3$) δ = 171.09, 166.07, 164.30, 148.54, 141.64, 134.50, 129.88, 129.45, 128.87, 128.37, 128.10, 123.16, 118.86, 115.11, 85.23, 53.39, 53.04, 50.06, 41.99, 40.20, 28.06. HRMS (ESI-TOF) calcd for $C_{25}H_{24}{}^{34.9689}ClNO_7$ ([M]+Na^+) = 508.1139, Found 508.1137; $C_{21}H_{17}{}^{36.9659}ClNO$ ([M]+Na^+) = 510.1109, Found 510.1120.

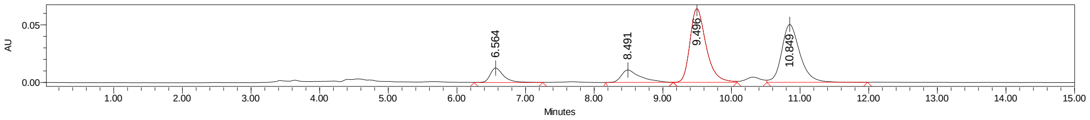

	Retention Time	Area	% Area
1	6.564	175554	7.63
2	8.491	184658	8.02
3	9.496	992026	43.10
4	10.849	949547	41.25

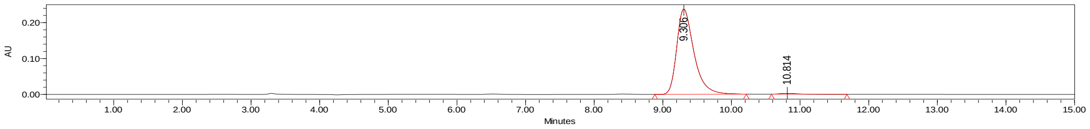

	Retention Time	Area	% Area
3	9.306	4132631	99.09
4	10.814	37794	0.91

1a-*tert*-buty 1,1-dimethyl 2-oxo-1a,2-dihydrocyclopropa[*c*]chromene-1,1,1a(7b*H*)-tricarboxylate (4r):

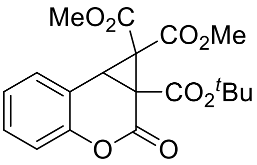

4r

Prepared according to the general procedure (72 h). The tittle compound **4r** was obtained as a white solid in 80% yield, >19:1 d.r., 82% *ee*. $[\alpha]^{23..8}_D$ = −12.5 (*c* = 0.40, in CH_2Cl_2). HPLC (Chiralcel IA, n-hexane/ *i*-PrOH = 98/2,

flow rate 1.0 mL/min, λ = 254 nm) t_r (major) = 8.96 min, t_r (minor) = 12.14 min. ^{1}H NMR (400 MHz, $CDCl_3$) δ 7.46 – 7.39 (m, 1H), 7.34 – 7.27 (m, 1H), 7.16 (t, J = 7.6 Hz, 1H), 7.02 (d, J = 8.4 Hz, 1H), 3.84 (s, 3H), 3.79 (s, 1H), 3.47 (s, 3H), 1.47 (s, 9H). ^{13}C NMR (100 MHz, $CDCl_3$) δ = 165.75, 163.62, 162.48, 159.37, 150.16, 129.87, 129.37, 125.00, 116.92, 113.99, 84.25, 53.81, 53.39, 43.58, 41.62, 33.57, 27.66.

HRMS (ESI-TOF) calcd for $C_{19}H_{20}O_8$ ([M]+Na$^+$) = 399.1050, Found 399.1051.

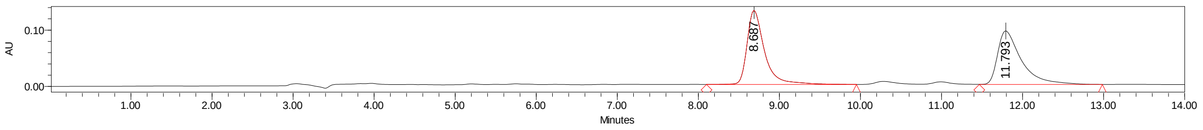

	Retention Time	Area	% Area
1	8.687	1970358	50.43
2	11.793	1936795	49.57

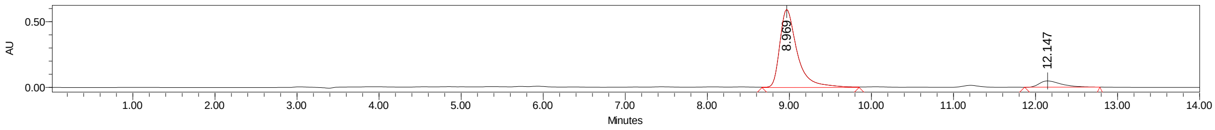

	Retention Time	Area	% Area
3	8.969	8764290	90.92
4	12.147	875189	9.08

1a-*tert*-buty 1,1-dimethyl 6-methyl-2-oxo-1a,2-dihydrocyclopropa[*c*]chromene-1,1,1a(7b*H*)-tricarboxylate (4s):

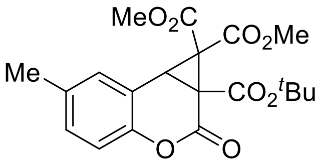

4s

Prepared according to the general procedure (72 h). The tittle compound **5b** was obtained as a white solid in 85% yield, >19:1 d.r., 76% *ee*. $[\alpha]^{23.7}_D$ = −6.7 (c = 0.18, in CH_2Cl_2). HPLC (Chiralcel IA, n-hexane/ *i*-PrOH = 90/10, flow rate 1.0 mL/min, λ = 254 nm) t_r (major) = 8.72 min, t_r (minor) = 10.03 min. ^{1}H NMR (400 MHz, $CDCl_3$) δ 7.24 (d, J = 1.2 Hz, 1H), 7.09 (dd, J = 8.4, 1.6 Hz, 1H), 6.91 (d, J = 8.4 Hz, 1H), 3.85 (s, 3H), 3.75 (s, 1H), 3.51 (s, 3H), 2.33 (s, 3H), 1.48 (s, 9H). ^{13}C NMR (100 MHz, $CDCl_3$) δ = 165.84, 163.64, 162.56, 159.56, 148.11, 134.73, 130.46, 129.55, 116.62, 113.62, 84.14, 53.73, 53.36, 43.54, 41.64, 33.74, 27.67, 20.67. HRMS (ESI-TOF) calcd for $C_{20}H_{22}O_8$ ([M]+Na$^+$) = 413.1207, Found 413.1216.

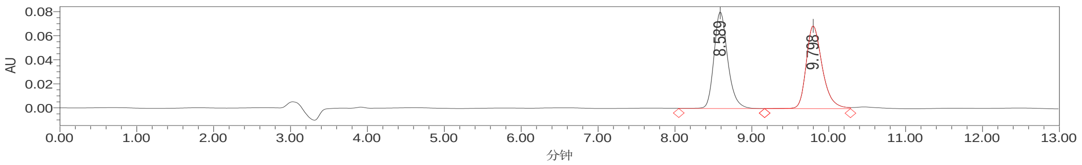

	Retention Time	Area	% Area
1	8.589	1001807	50.07
2	9.798	998817	49.93

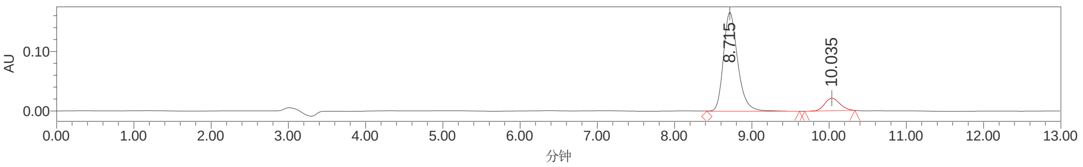

	Retention Time	Area	% Area
3	8.715	2132086	88.04
4	10.035	289538	11.96

Trimethyl 2-oxo-2*H*-spiro[benzofuran-3,1'-cyclopropane]-2',2',3'- tricarboxylate (4t):

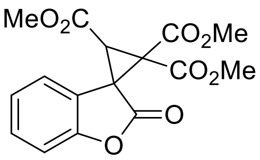

4t

Prepared according to the general procedure (24 h). The tittle compound **4t** was obtained as a white solid in 95% yield, >19:1 d.r., 0% *ee*. HPLC (Chiralcel IA, n-hexane/ *i*-PrOH = 90/10, flow rate 1.0 mL/min, λ = 254 nm) t_r = 9.76 min, t_r = 11.03 min. ^{1}H NMR (400 MHz, $CDCl_3$) δ 7.44 – 7.35 (m, 2H), 7.18 – 7.13 (m, 2H), 3.82 (d, *J* = 2.0 Hz, 6H), 3.75 (s, 3H), 3.47 (s, 1H). ^{13}C NMR (101 MHz, $CDCl_3$) δ = 171.98, 164.98, 163.69, 162.58, 154.60, 129.92, 127.32, 124.04, 119.47, 110.77, 53.93, 53.58, 52.93, 47.27, 38.28, 37.96. HRMS (ESI-TOF) calcd for $C_{16}H_{14}O_8$ ([M]+Na^+) = 357.0581, Found 357.0579.

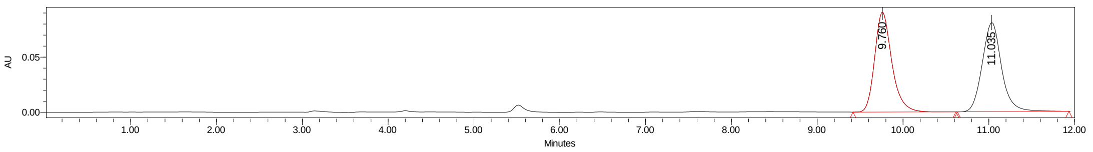

	Retention Time	Area	% Area
1	9.760	1256548	49.91
2	11.035	1261012	50.09

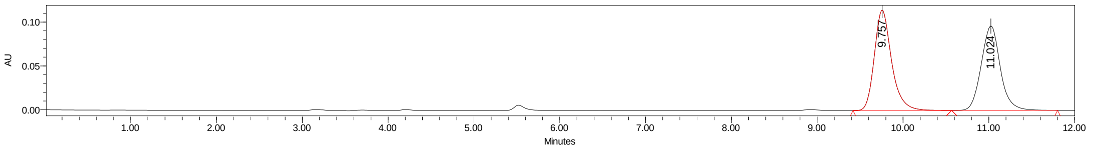

	Retention Time	Area	% Area
3	9.757	1596707	51.81
4	11.024	1485045	48.19

3'-tert-butyl 2',2'-dimethyl 2-oxo-2*H*-spiro[benzofuran-3,1'-cyclopropane]-2',2',3'-tricarboxylate (4t'):

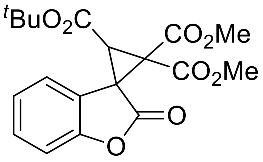

4t'

Prepared according to the general procedure (24 h). The tittle compound **4t'** was obtained as a white solid in 85% yield, >19:1 d.r., 0% *ee*. HPLC (Chiralcel IC, n-hexane/ *i*-PrOH = 90/10, flow rate 1.0 mL/min, λ = 254 nm) t_r = 11.16 min, t_r = 11.51 min. ^{1}H NMR (400 MHz, $CDCl_3$) δ 7.46 (dd, *J* = 8.0, 1.2 Hz, 1H), 7.37 (td, *J* = 8.0, 1.6 Hz, 1H), 7.20 – 7.11 (m, 2H), 3.82 (d, *J* = 6.0 Hz, 6H), 3.39 (s, 1H), 1.42 (s, 9H). ^{13}C NMR (100 MHz, $CDCl_3$) δ

= 172.29, 164.02, 163.33, 162.86, 154.53, 129.71, 127.45, 123.78, 119.61, 110.68, 83.69, 53.85, 53.38, 47.06, 39.19, 38.10, 27.94.

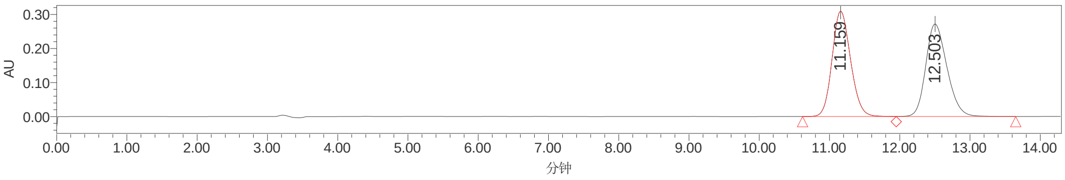

	Retention Time	Area	% Area
1	11.159	5661339	49.90
2	12.503	5684111	50.10

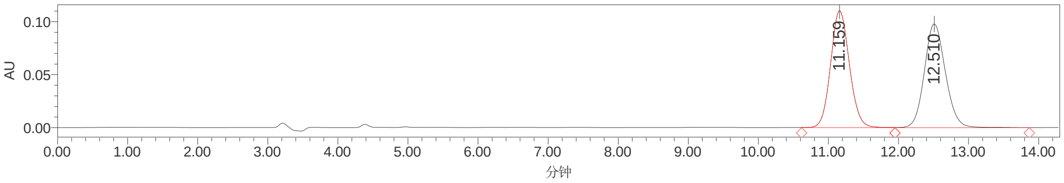

	Retention Time	Area	% Area
3	11.159	2057718	50.13
4	12.510	2047332	49.87

3-tert-butyl 2,2-dimethyl 2'-oxo-1'-tosylspiro[cyclopropane-1,3'-indoline]-2,2,3-tricarboxylate (4u):

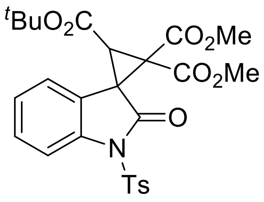

4u

Prepared according to the general procedure (24 h). The tittle compound **4u** was obtained as a white solid in 98% yield, >19:1 d.r., 0% *ee*. HPLC (Chiralcel IC, n-hexane/ *i*-PrOH = 90/10, flow rate 1.0 mL/min, λ = 254 nm) t_r = 12.94 min, t_r = 17.87 min. ^{1}H NMR (400 MHz, $CDCl_3$) δ 7.99 (t, *J* = 8.4 Hz, 3H), 7.47 – 7.31 (m, 4H), 7.13 (td, *J* = 8.0, 1.2 Hz, 1H), 3.76 (s, 3H), 3.61 (s, 3H), 3.30 (s, 1H), 2.43 (s, 3H), 1.41 (s, 9H). ^{13}C NMR (101 MHz, $CDCl_3$) δ = 170.40, 164.25, 163.43, 162.96, 145.93, 140.32, 134.89, 129.79, 129.39, 128.22, 127.25, 124.00, 119.72, 113.24, 83.50, 53.45, 53.31, 47.64, 40.05, 38.82, 27.93, 21.75.

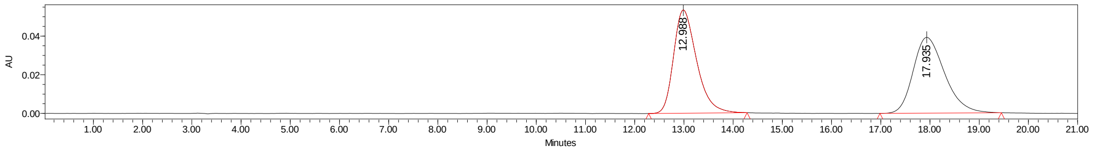

	Retention Time	Area	% Area
1	12.988	1730474	49.83
2	17.935	1742434	50.17

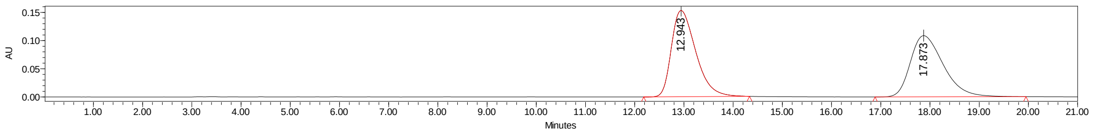

	Retention Time	Area	% Area
3	12.943	5249385	50.99
4	17.873	5044710	49.01

2-(tert-butyl) 1,1-dimethyl –2-((R)-2-oxo-3-(phenylamino)indolin-3-yl)ethane-1,1,2,-tricarboxylate (5):

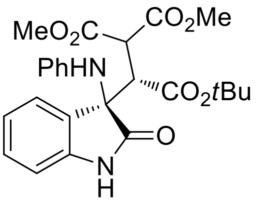

5

Prepared according to the general procedure (24 h). The tittle compound **5** was obtained as a yellow oil in 90% yield, >19:1 d.r. (97:3 d.r.), 99% *ee*. HPLC (Chiralcel IA, n-hexane/ *i*-PrOH = 90/10, flow rate 1.0 mL/min, λ = 254 nm) t_r (major) = 27.03 min, t_r (minor) = 35.2 min. $[\alpha]^{14.0}_D$ = +12.5 (*c* = 0.45, in CH_2Cl_2). ^{1}H NMR (400 MHz, $CDCl_3$) δ 8.50 (s, 1H), 7.40 (d, *J* = 7.5 Hz, 1H), 7.23 (t, *J* = 7.7 Hz, 1H), 7.02 (t, *J* = 7.5 Hz, 1H), 6.93 (t, *J* = 7.8 Hz, 2H), 6.82 (d, *J* = 7.8 Hz, 1H), 6.67 (t, *J* = 7.3 Hz, 1H), 6.37 (d, *J* = 7.9 Hz, 2H), 5.14 (s, 1H), 4.28 (d, *J* = 8.8 Hz, 1H), 3.95 (d, *J* = 8.9 Hz, 1H), 3.74 (s, 3H), 3.60 (s, 3H), 1.29 (s, 9H). ^{13}C NMR (100 MHz, $CDCl_3$) δ = 177.14, 169.12, 168.76, 167.96, 144.31, 140.66, 129.91, 128.97, 127.20, 126.28, 122.95, 120.15, 116.79, 110.51, 82.57, 65.17, 53.26, 52.82, 52.34, 50.00, 27.52. Dept^{135} NMR (100 MHz, $CDCl_3$) δ = 129.91, 128.97, 126.28, 122.95, 120.15, 116.78, 110.51, 77.24, 53.26, 52.82, 52.34, 49.99, 27.52. HRMS (ESI-TOF) calcd for $C_{25}H_{28}N_2O_7$ ($[M]+Na^+$) = 491.1794, Found 491.1786.

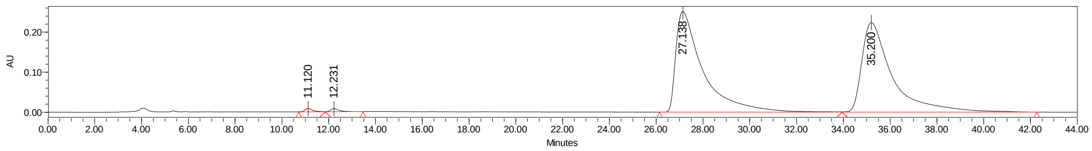

	Retention Time	Area	% Area
1	11.120	194812	0.48
2	12.231	201813	0.50
3	27.138	20237292	49.68
4	35.200	20104186	49.35

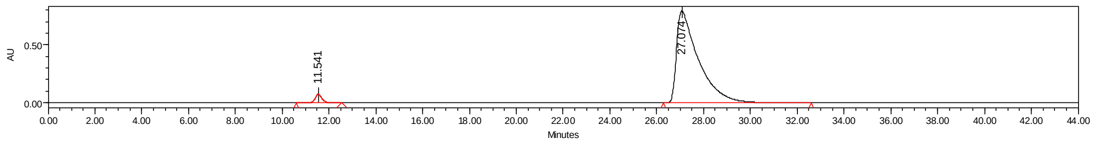

	Retention Time	Area	% Area
1	11.541	1473502	2.77
3	27.074	51650253	97.23

8. References

1 a) Y. H. Wen, X. Huang, J. L. Huang, Y. Xiong, B. Qin, X. M. Feng, *Synlett* 2005, 2445; b) Z. P. Yu, X. H. Liu, Z. H. Dong, M. S. Xie, X. M. Feng, *Angew. Chem. Int. Ed.* 2008, **47**, 1308; c) K. Zheng, B. Qin, X. H. Liu, X. M. Feng, *J. Org. Chem.* 2007, **72**, 8478; d) X. Zhang, D. H. Chen, X. H. Liu, X. M. Feng, *J. Org. Chem.* 2007, **72**, 5227; e) X. Zhou, D. J. Shang, Q. Zhang, L. L. Lin, X. H. Liu, X. M. Feng, *Org. Lett.* 2009, **11**, 1401.

2 a) B. M. Trost, N. Cramer, S. M. Silverman *J. Am. Chem. Soc.* 2007, **129**, 12396; b) X. H. Chen, Q. Wei, S. W. Luo, H. Xiao, L. -Z. Gong, *J. Am. Chem. Soc.* 2009 , **131** , 13819; c) G. Wang, X. H. Liu, T. Y. Huang, Y. L. Kuang, L. L. Lin, X. M. Feng, *Org. Lett.* 2013, **15**, 76.

3 a) G. Wille, W. Steglich, Synthesis, 2001, 759; b) B. Tan, N. R. Candeias, C. F. Barbas III, *J. Am. Chem. Soc.* 2011, **133**, 4672; c) A. Noole, N. S. Sucman, M. A. Kabeshov, T. Kanger, F. Z. Macaev, A. V. Malkov, *Chem. Eur. J.* 2012, **18**, 14929.

4 a) P. Müller, D. Fernández, *Helv. Chim. Acta* **1995**, *78*, 947; b) S. R. Goudreau, D. Marcoux, A. B. Charette, *J. Org. Chem.* 2009, **74,** 8939.

5 Y. L. Kuang, X. H. Liu, L. Chang, M. Wang, L. L. Lin, X. M. Feng, *Org. Lett.* 2011, **13**, 3814.

9. Copy of ^{1}H NMR and ^{13}C NMR spectra

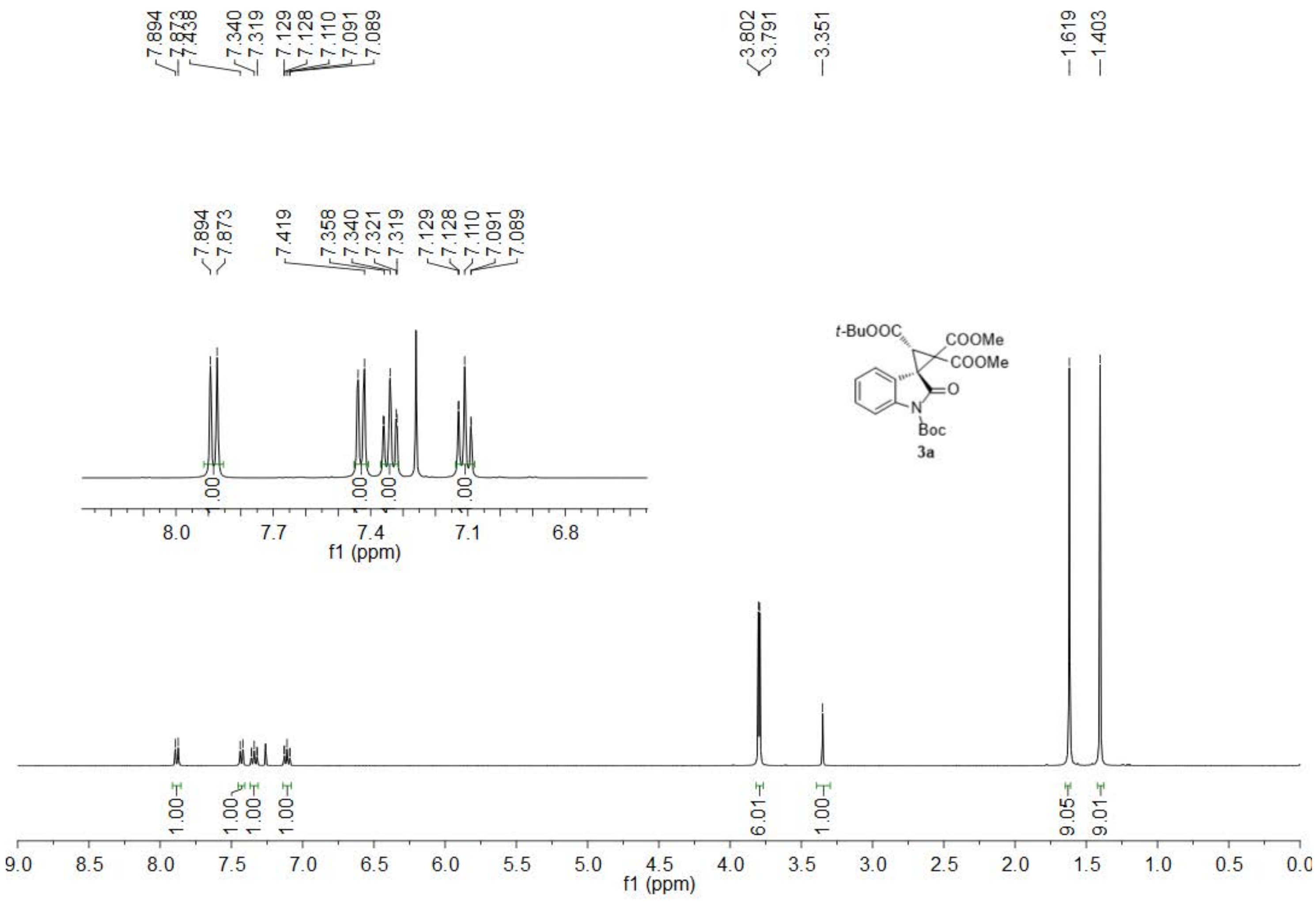

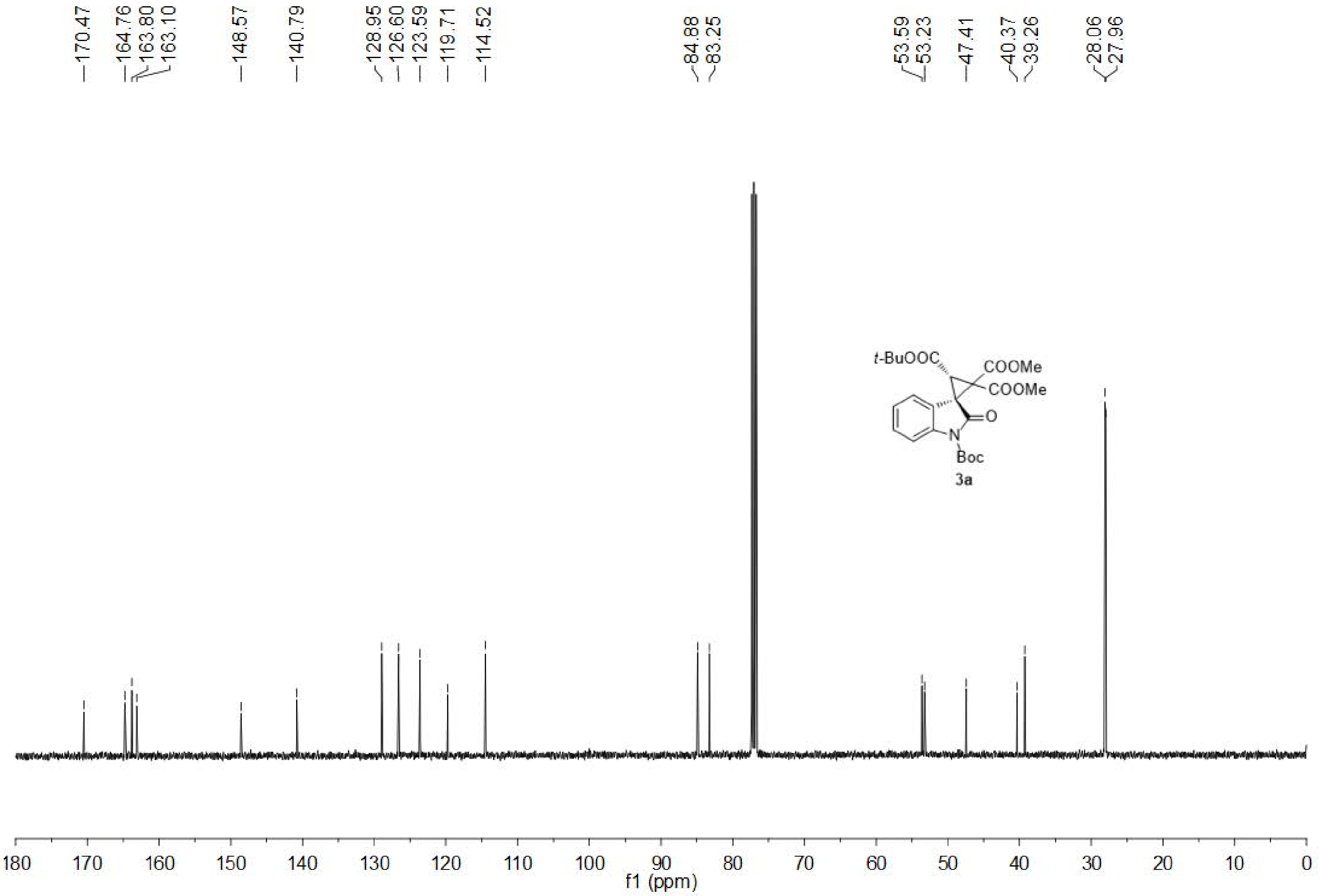

170.47
164.76
163.80
163.10
148.57
140.79
128.95
126.60
123.59
119.71
114.52
84.88
83.25
53.59
53.23
47.41
40.37
39.26
28.06
27.96
t-BuOOC
COOMe
COOMe
O
N
Boc
3a
180 170 160 150 140 130 120 110 100 90 80 70 60 50 40 30 20 10 0
f1 (ppm)

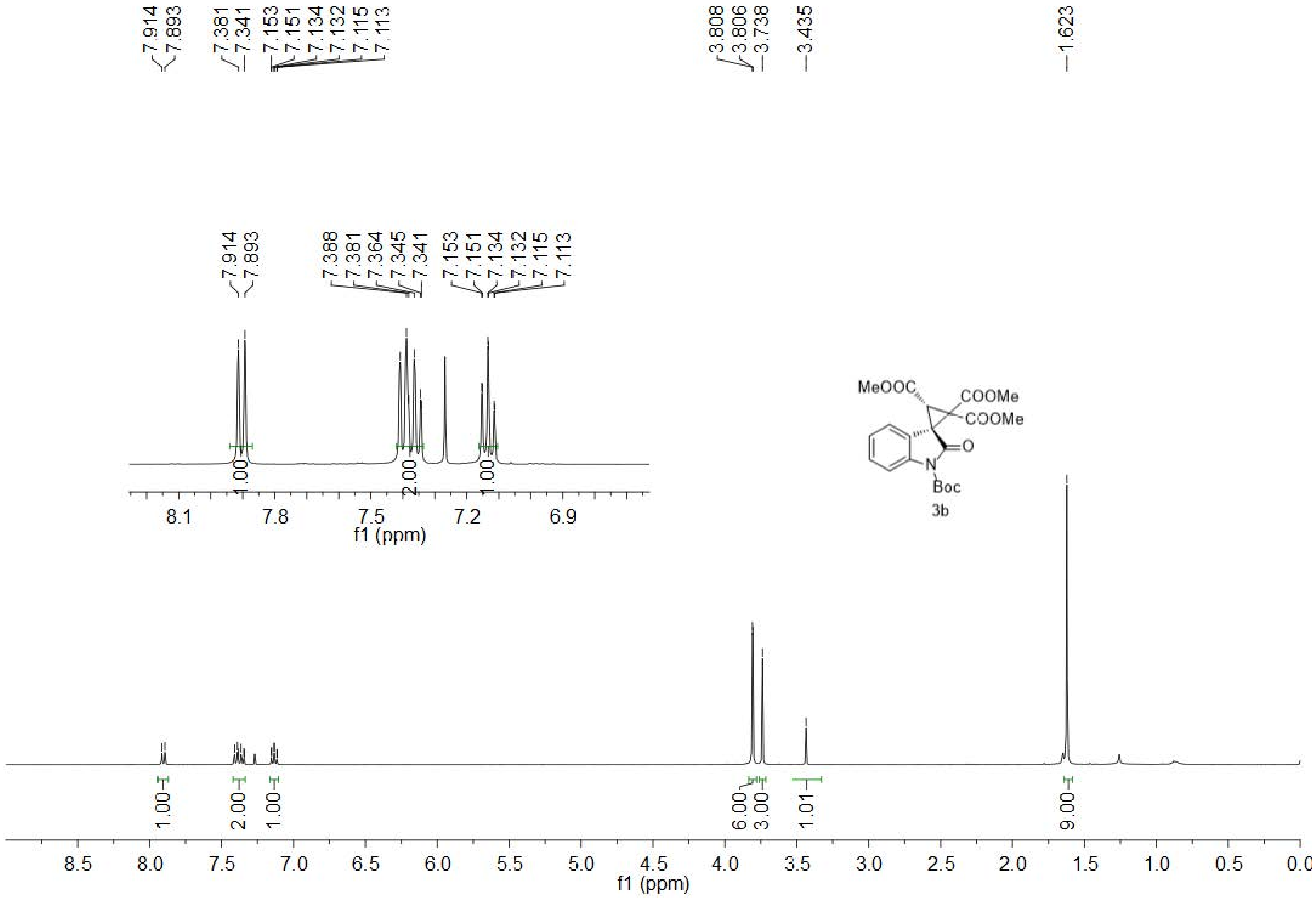
7.914
7.893
7.381
7.341
7.153
7.151
7.134
7.132
7.115
7.113
3.808
3.806
3.738
3.435
1.623
7.388
7.364
7.345
1.00
2.00
6.00
3.00
1.01
9.00
8.1
7.8
7.5
7.2
6.9
f1 (ppm)
8.5
8.0
7.0
6.5
6.0
5.5
5.0
4.5
4.0
3.5
3.0
2.5
2.0
1.5
1.0
0.5
0.0
MeOOC
COOMe
COOMe
N
O
Boc

3b

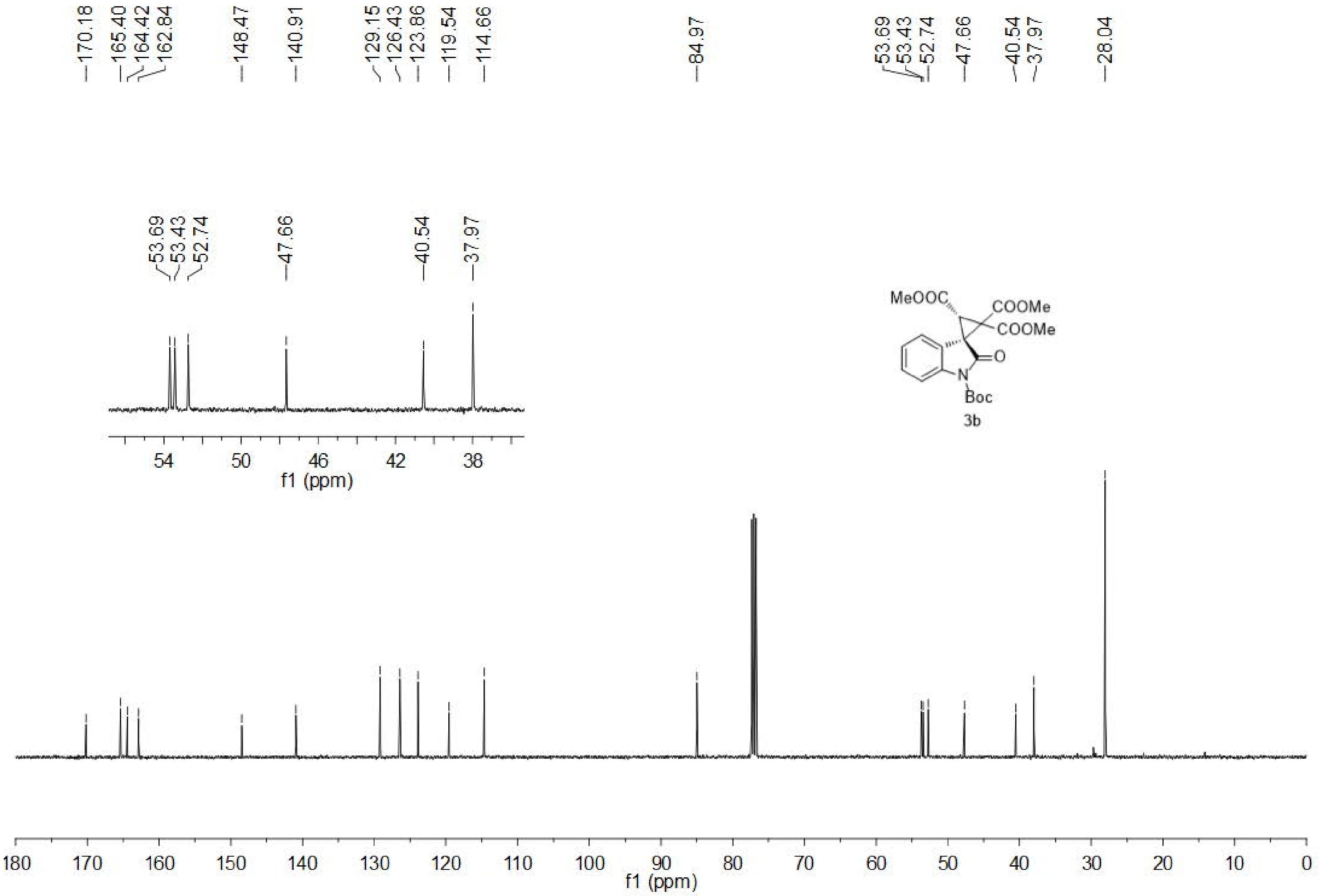
170.18
165.40
164.42
162.84
148.47
140.91
129.15
126.43
123.86
119.54
114.66
84.97
53.69
53.43
52.74
47.66
40.54
37.97
28.04
MeOOC
COOMe
COOMe
O
N
Boc
3b
54
50
46
42
38
f1 (ppm)
180
170
160
150
140
130
120
110
100
90
80
70
60
50
40
30
20
10
0
f1 (ppm)

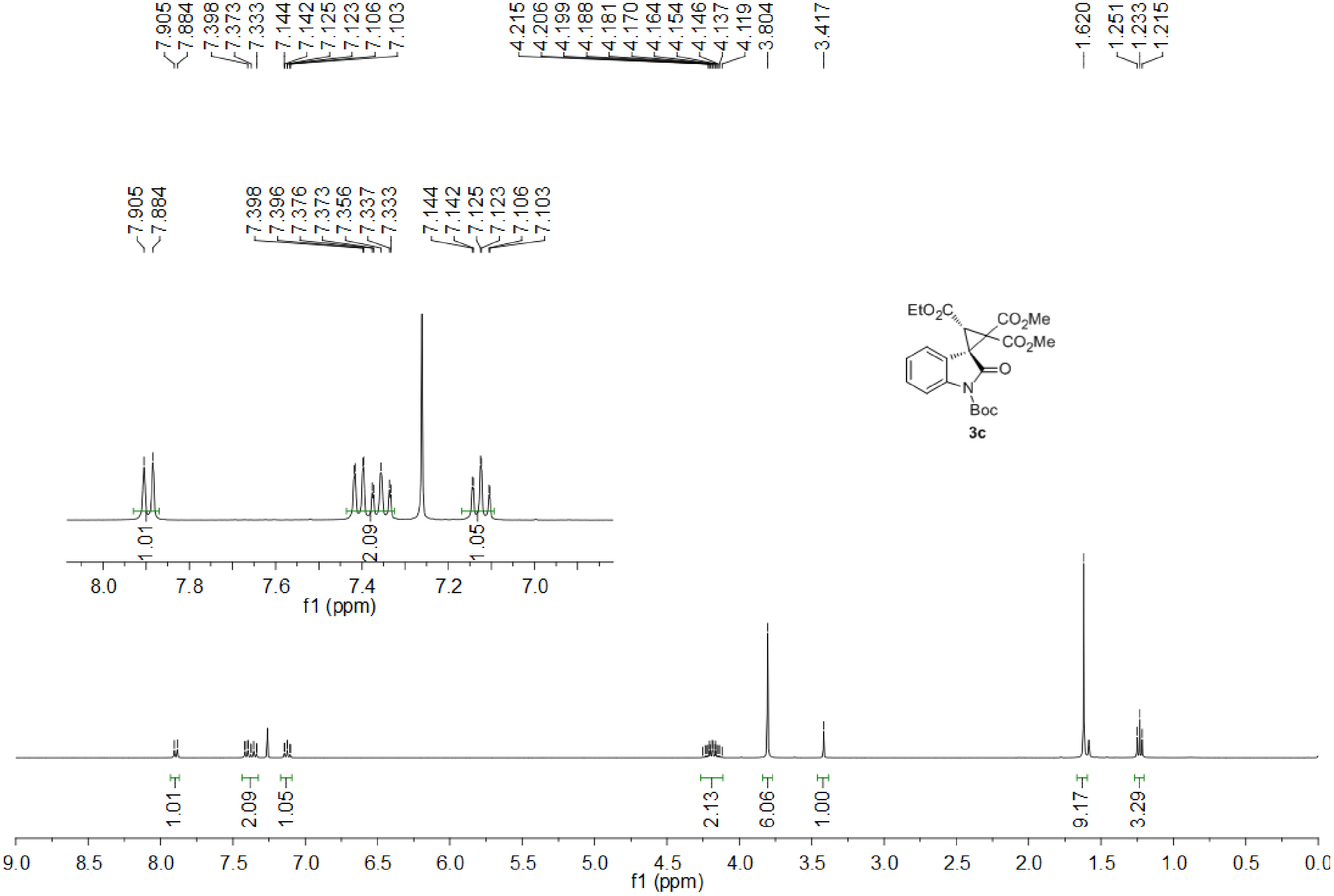
7.905 7.884 7.398 7.373 7.333 7.144 7.142 7.125 7.123 7.106 7.103
4.215 4.206 4.199 4.188 4.181 4.170 4.164 4.154 4.146 4.137 4.119 3.804
3.417
1.620 1.251 1.233 1.215
7.905 7.884
7.398 7.396 7.376 7.373 7.356 7.337 7.333
7.144 7.142 7.125 7.123 7.106 7.103
EtO2C
CO2Me
CO2Me
O
N
Boc
3c
1.01
2.09
1.05
8.0 7.8 7.6 7.4 7.2 7.0
f1 (ppm)
1.01 2.09 1.05 2.13 6.06 1.00 9.17 3.29
9.0 8.5 8.0 7.5 7.0 6.5 6.0 5.5 5.0 4.5 4.0 3.5 3.0 2.5 2.0 1.5 1.0 0.5 0.0
f1 (ppm)

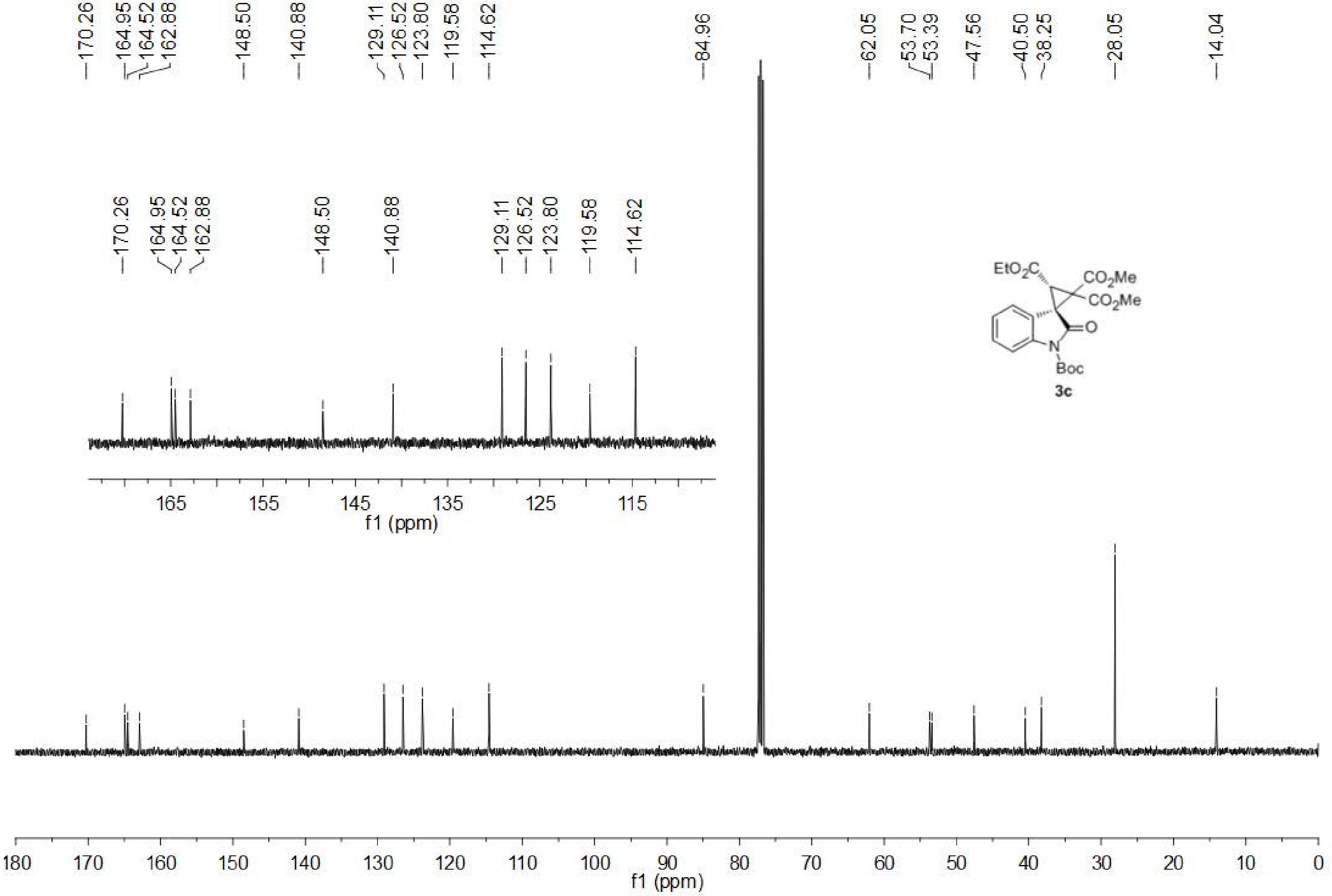
170.26
164.95
164.52
162.88
148.50
140.88
129.11
126.52
123.80
119.58
114.62
84.96
62.05
53.70
53.39
47.56
40.50
38.25
28.05
14.04
EtO2C
CO2Me
CO2Me
O
N
Boc
3c
f1 (ppm)
f1 (ppm)

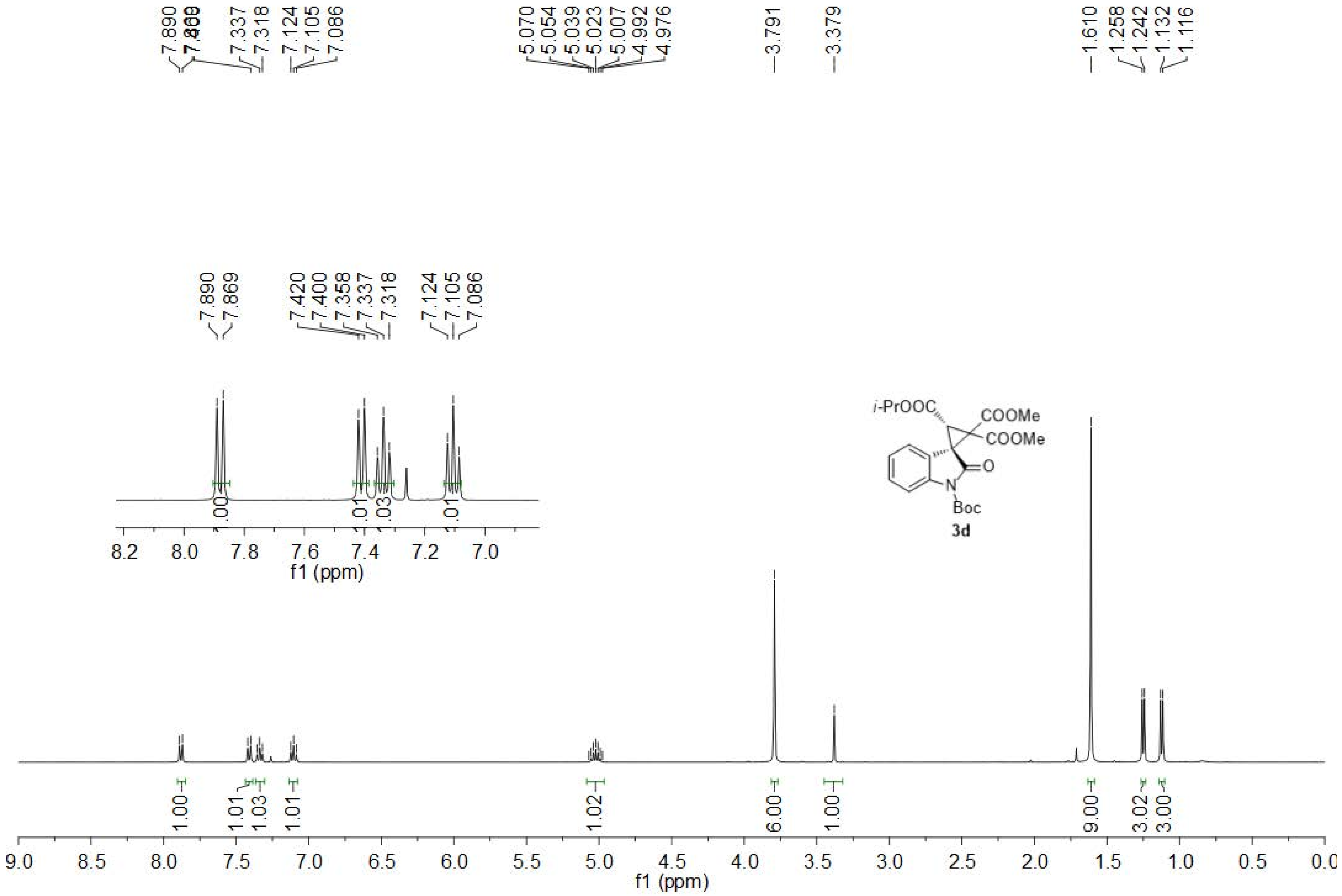

7.890
7.337
7.318
7.124
7.105
7.086
5.070
5.054
5.039
5.023
5.007
4.992
4.976
3.791
3.379
1.610
1.258
1.242
1.132
1.116
7.890
7.869
7.420
7.400
7.358
7.337
7.318
7.124
7.105
7.086
1.00
1.01
1.03
1.01
8.2
8.0
7.8
7.6
7.4
7.2
7.0
f1 (ppm)
i-PrOOC
COOMe
COOMe
O
N
Boc
3d
1.00
1.01
1.03
1.01
1.02
6.00
1.00
9.00
3.02
3.00
9.0
8.5
8.0
7.5
7.0
6.5
6.0
5.5
5.0
4.5
4.0
3.5
3.0
2.5
2.0
1.5
1.0
0.5
f1 (ppm)

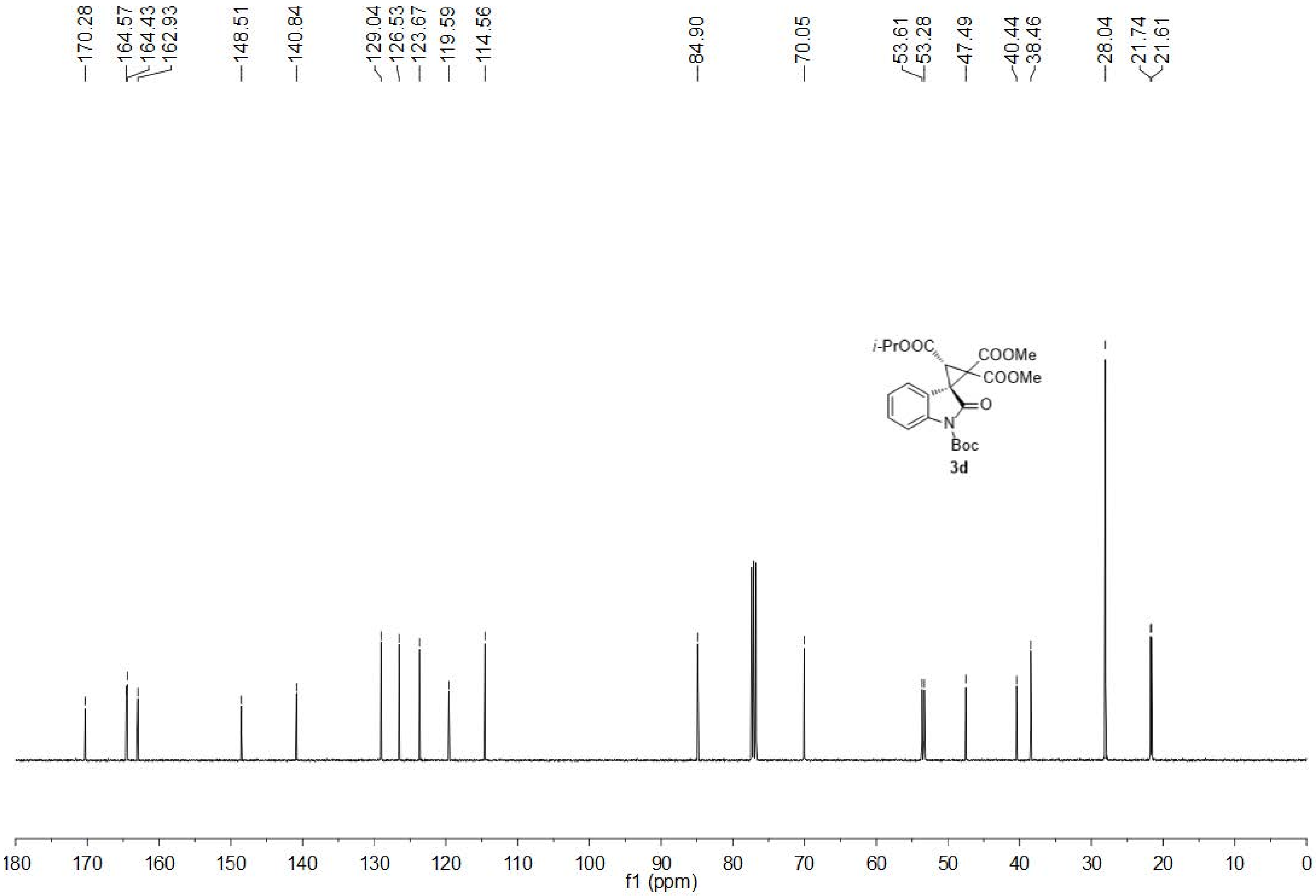

170.28
164.57
164.43
162.93
148.51
140.84
129.04
126.53
123.67
119.59
114.56
84.90
70.05
53.61
53.28
47.49
40.44
38.46
28.04
21.74
21.61
i-PrOOC
COOMe
COOMe
O
N
Boc
3d
180 170 160 150 140 130 120 110 100 90 80 70 60 50 40 30 20 10 0
f1 (ppm)

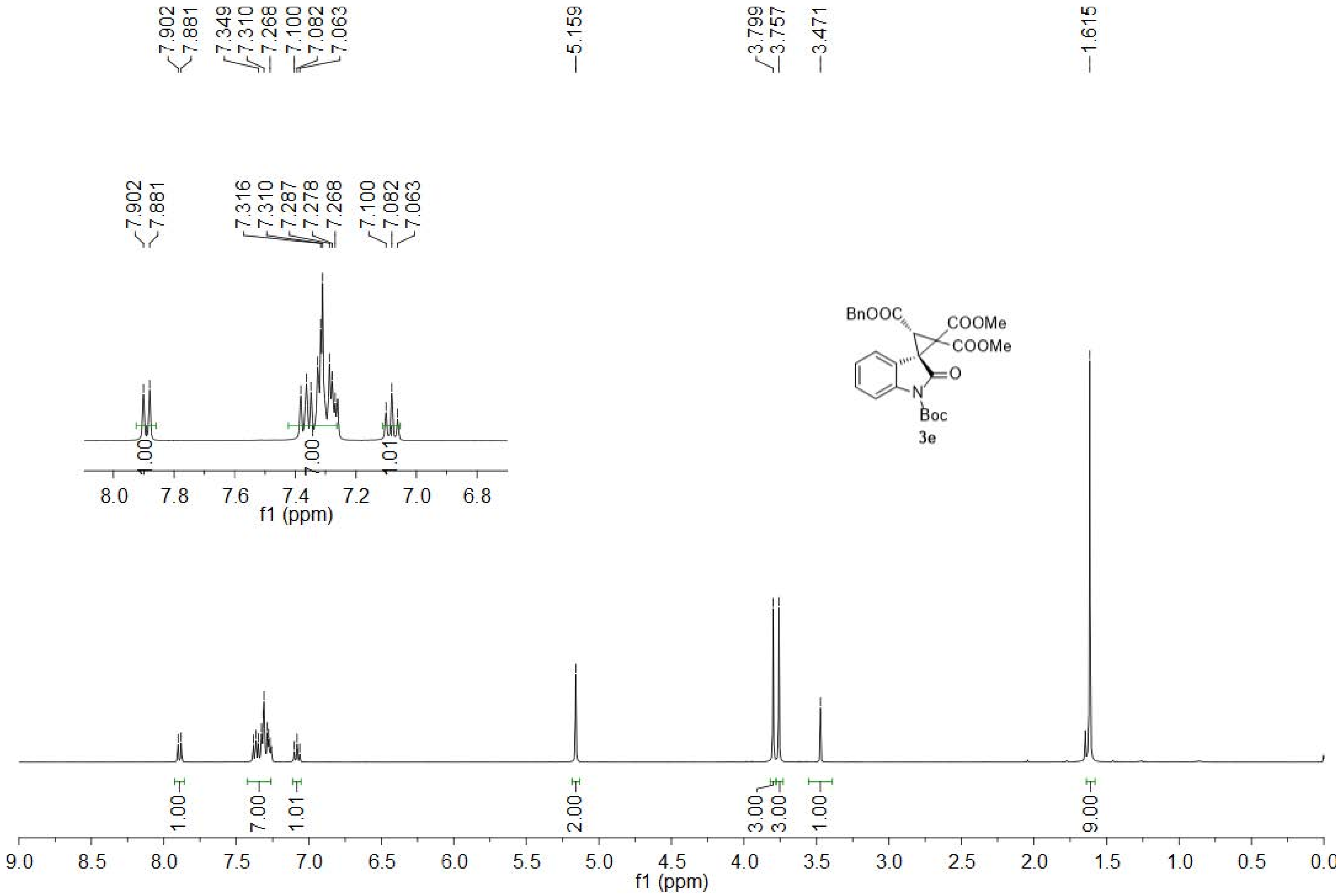
7.902
7.881
7.349
7.310
7.268
7.100
7.082
7.063
5.159
3.799
3.757
3.471
1.615
7.902
7.881
7.316
7.310
7.287
7.278
7.268
7.100
7.082
7.063
1.00
7.00
1.01
8.0 7.8 7.6 7.4 7.2 7.0 6.8
f1 (ppm)
BnOOC
COOMe
COOMe
O
N
Boc
3e
1.00
7.00
1.01
2.00
3.00
3.00
1.00
9.00
9.0 8.5 8.0 7.5 7.0 6.5 6.0 5.5 5.0 4.5 4.0 3.5 3.0 2.5 2.0 1.5 1.0 0.5 0.0
f1 (ppm)

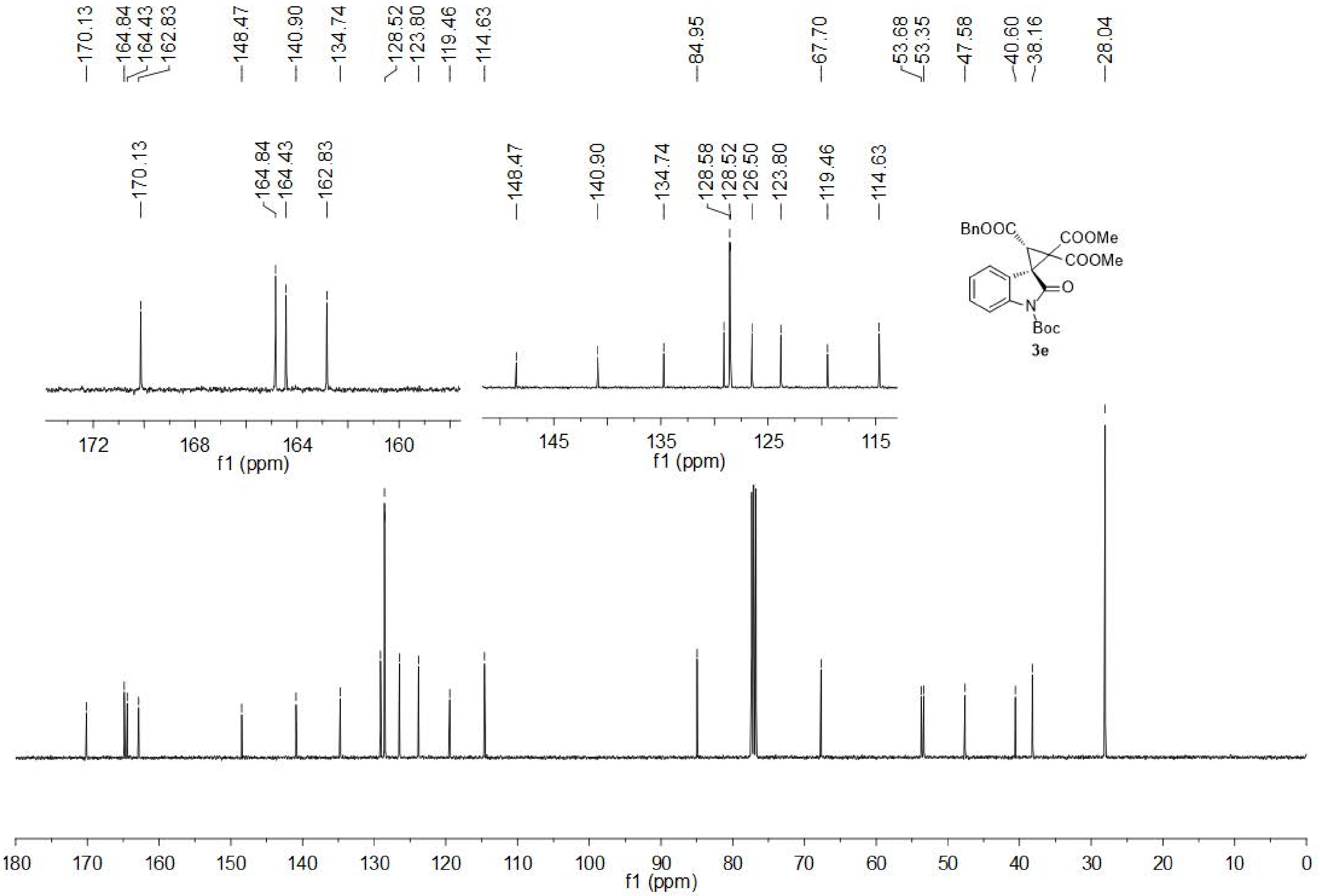
170.13
164.84
164.43
162.83
148.47
140.90
134.74
128.52
123.80
119.46
114.63
84.95
67.70
53.68
53.35
47.58
40.60
38.16
28.04
128.58
126.50
f1 (ppm)
BnOOC
COOMe
COOMe
O
N
Boc
3e

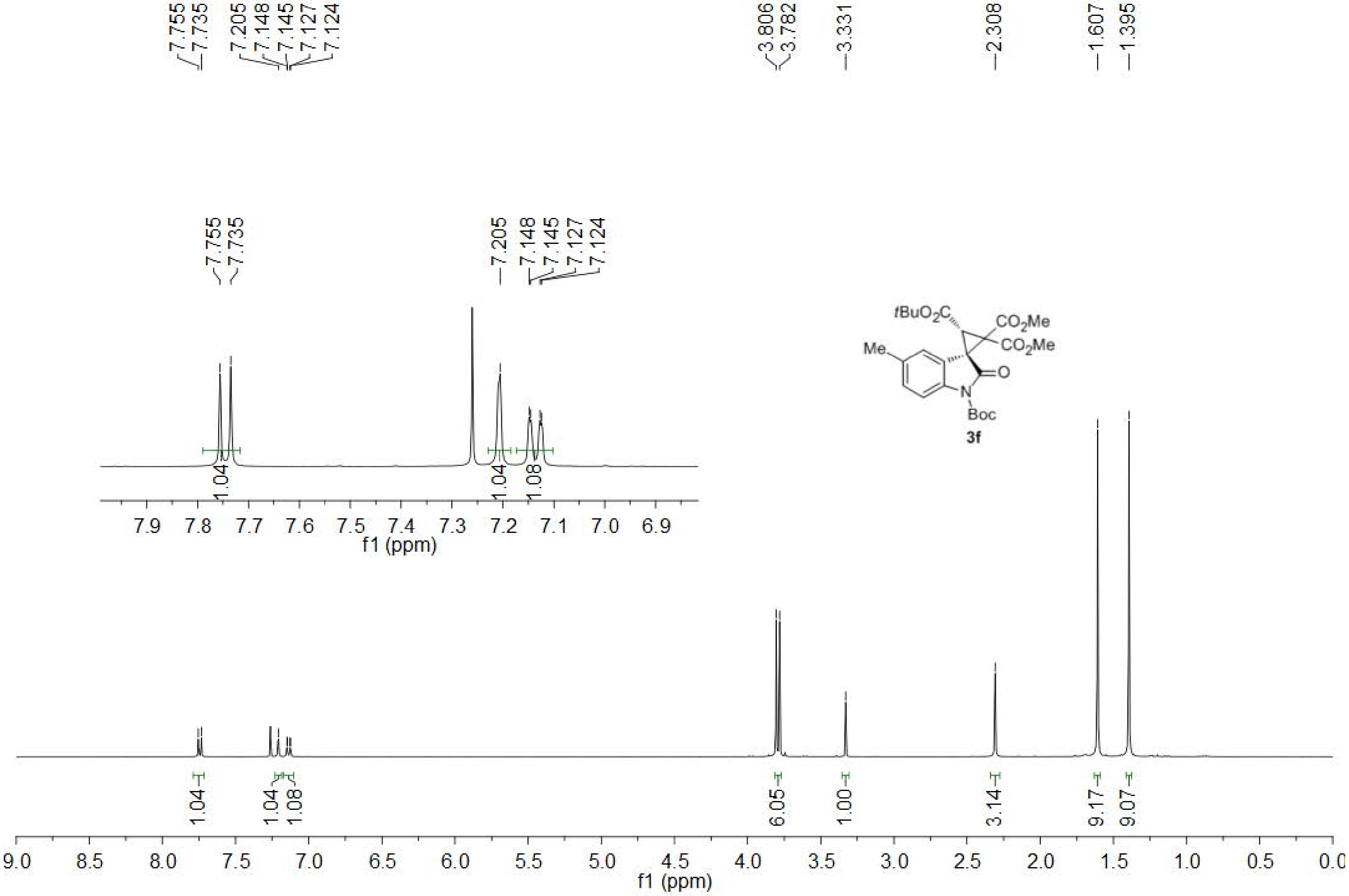
7.755
7.735
7.205
7.148
7.145
7.127
7.124
3.806
3.782
3.331
2.308
1.607
1.395
tBuO2C
CO2Me
CO2Me
Me
O
N
Boc
3f
1.04
1.04
1.08
6.05
1.00
3.14
9.17
9.07
7.9 7.8 7.7 7.6 7.5 7.4 7.3 7.2 7.1 7.0 6.9
f1 (ppm)
9.0 8.5 8.0 7.5 7.0 6.5 6.0 5.5 5.0 4.5 4.0 3.5 3.0 2.5 2.0 1.5 1.0 0.5 0.0
f1 (ppm)

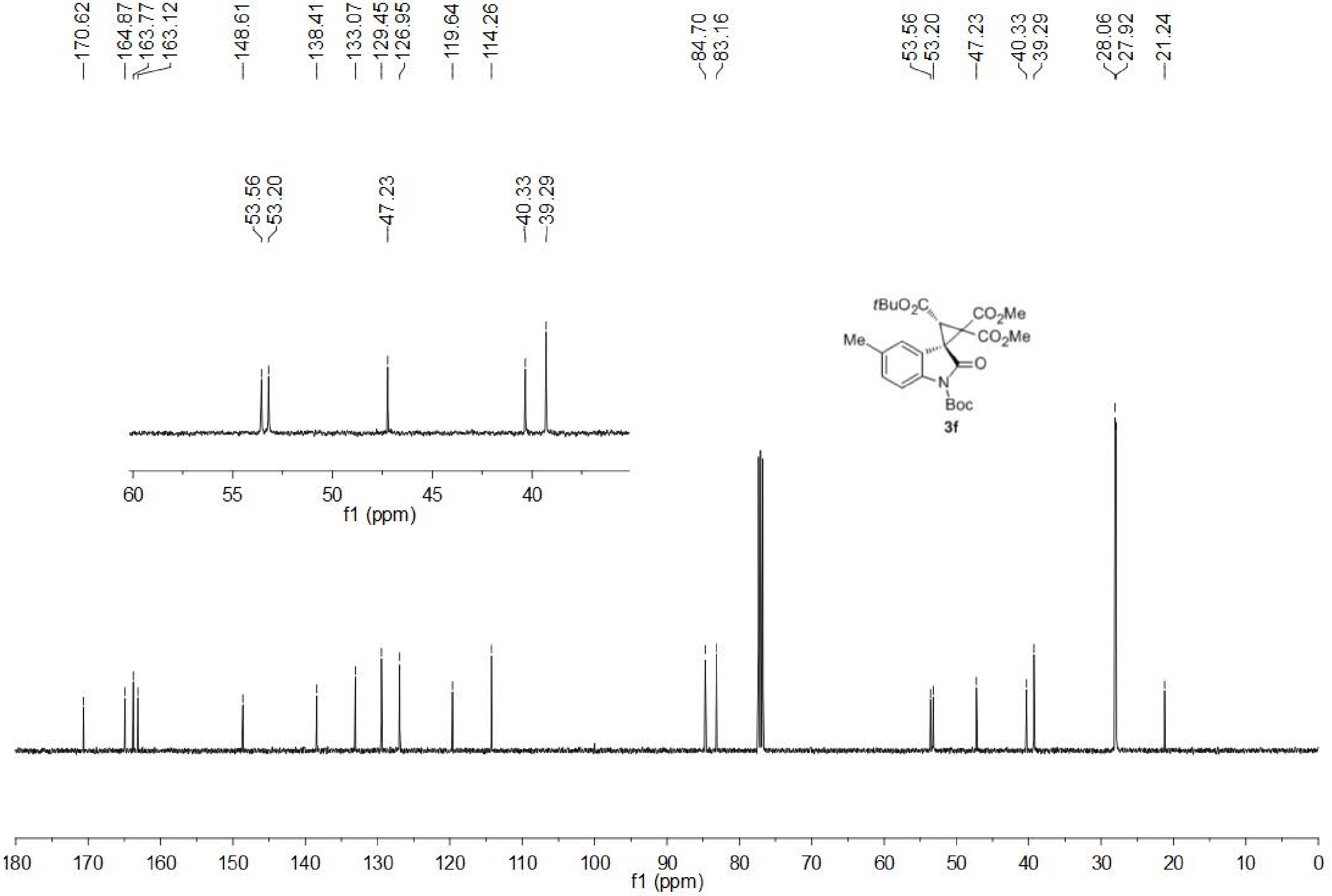

170.62
164.87
163.77
163.12
148.61
138.41
133.07
129.45
126.95
119.64
114.26
84.70
83.16
53.56
53.20
47.23
40.33
39.29
28.06
27.92
21.24
tBuO2C
CO2Me
CO2Me
Me
O
N
Boc
3f
f1 (ppm)

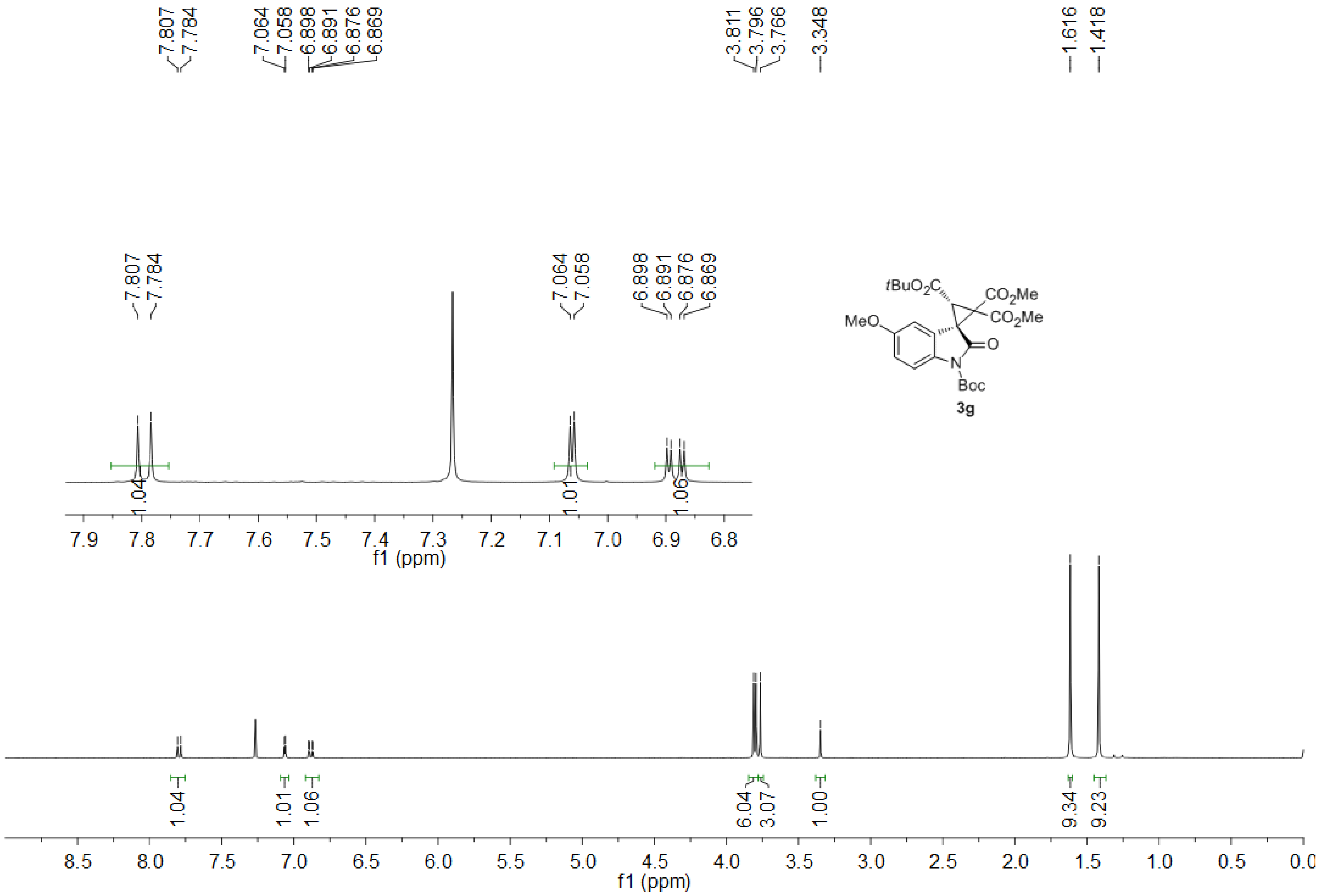
7.807
7.784
7.064
7.058
6.898
6.891
6.876
6.869
3.811
3.796
3.766
3.348
1.616
1.418
1.04
1.01
1.06
7.9
7.8
7.7
7.6
7.5
7.4
7.3
7.2
7.1
7.0
6.9
6.8
f1 (ppm)
tBuO2C
CO2Me
CO2Me
MeO
O
N
Boc
3g
1.04
1.01
1.06
6.04
3.07
1.00
9.34
9.23
8.5
8.0
7.5
7.0
6.5
6.0
5.5
5.0
4.5
4.0
3.5
3.0
2.5
2.0
1.5
1.0
0.5
0.0
f1 (ppm)

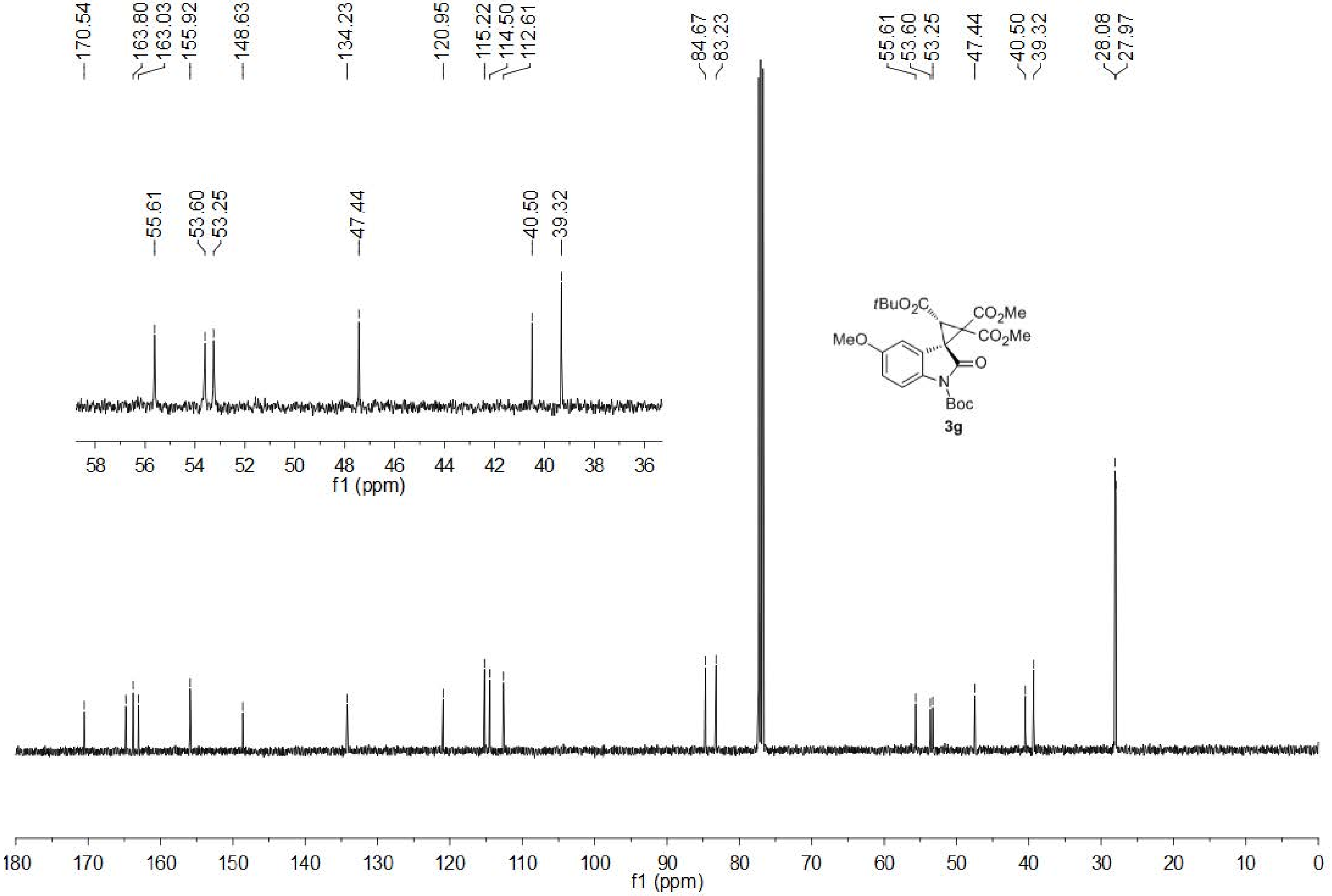

170.54
163.80
163.03
155.92
148.63
134.23
120.95
115.22
114.50
112.61
84.67
83.23
55.61
53.60
53.25
47.44
40.50
39.32
28.08
27.97
tBuO2C
CO2Me
CO2Me
MeO
N
Boc
O
3g
58 56 54 52 50 48 46 44 42 40 38 36
f1 (ppm)
180 170 160 150 140 130 120 110 100 90 80 70 60 50 40 30 20 10 0
f1 (ppm)

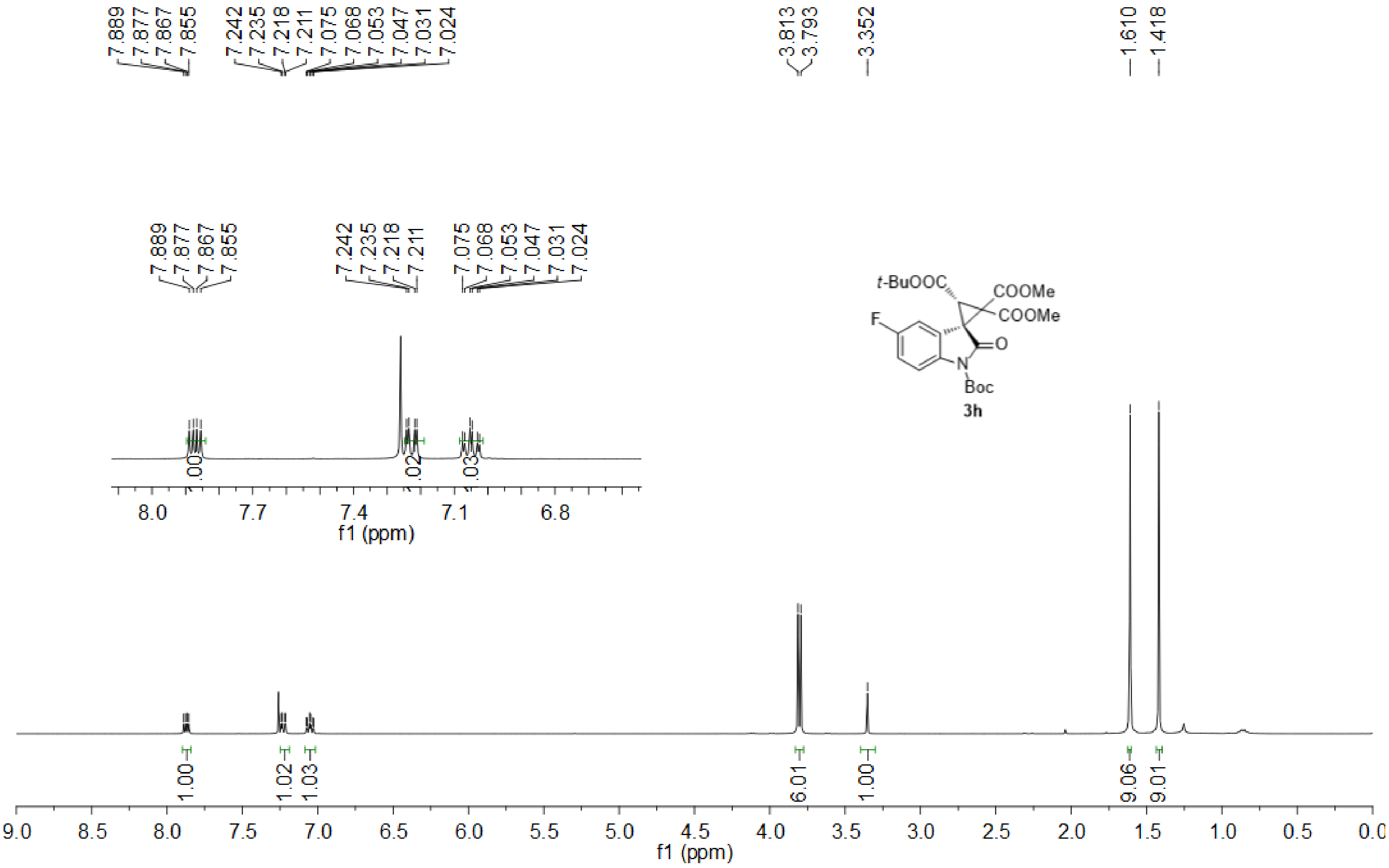

7.889
7.877
7.867
7.855
7.242
7.235
7.218
7.211
7.075
7.068
7.053
7.047
7.031
7.024
3.813
3.793
3.352
1.610
1.418
t-BuOOC
COOMe
COOMe
F
N
O
Boc
3h
8.0
7.7
7.4
7.1
6.8
f1 (ppm)
1.00
1.02
1.03
6.01
1.00
9.06
9.01
9.0
8.5
8.0
7.5
7.0
6.5
6.0
5.5
5.0
4.5
4.0
3.5
3.0
2.5
2.0
1.5
1.0
0.5
0.0
f1 (ppm)

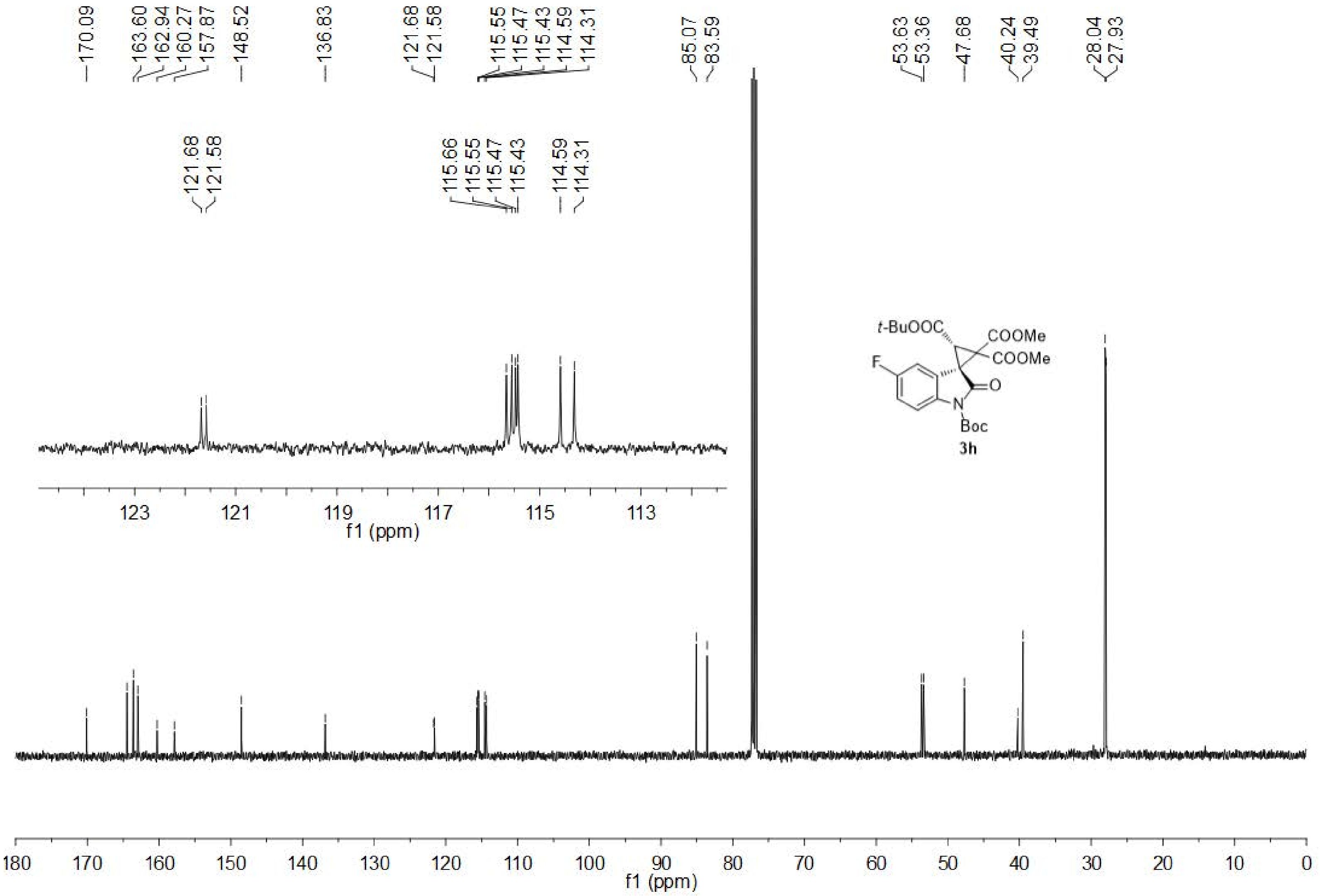

170.09
163.60
162.94
160.27
157.87
148.52
136.83
121.68
121.58
115.55
115.47
115.43
114.59
114.31
85.07
83.59
53.63
53.36
47.68
40.24
39.49
28.04
27.93
121.68
121.58
115.66
115.55
115.47
115.43
114.59
114.31
123
121
119
117
115
113
f1 (ppm)
t-BuOOC
COOMe
COOMe
F
O
N
Boc
3h
180
170
160
150
140
130
120
110
100
90
80
70
60
50
40
30
20
10
0
f1 (ppm)

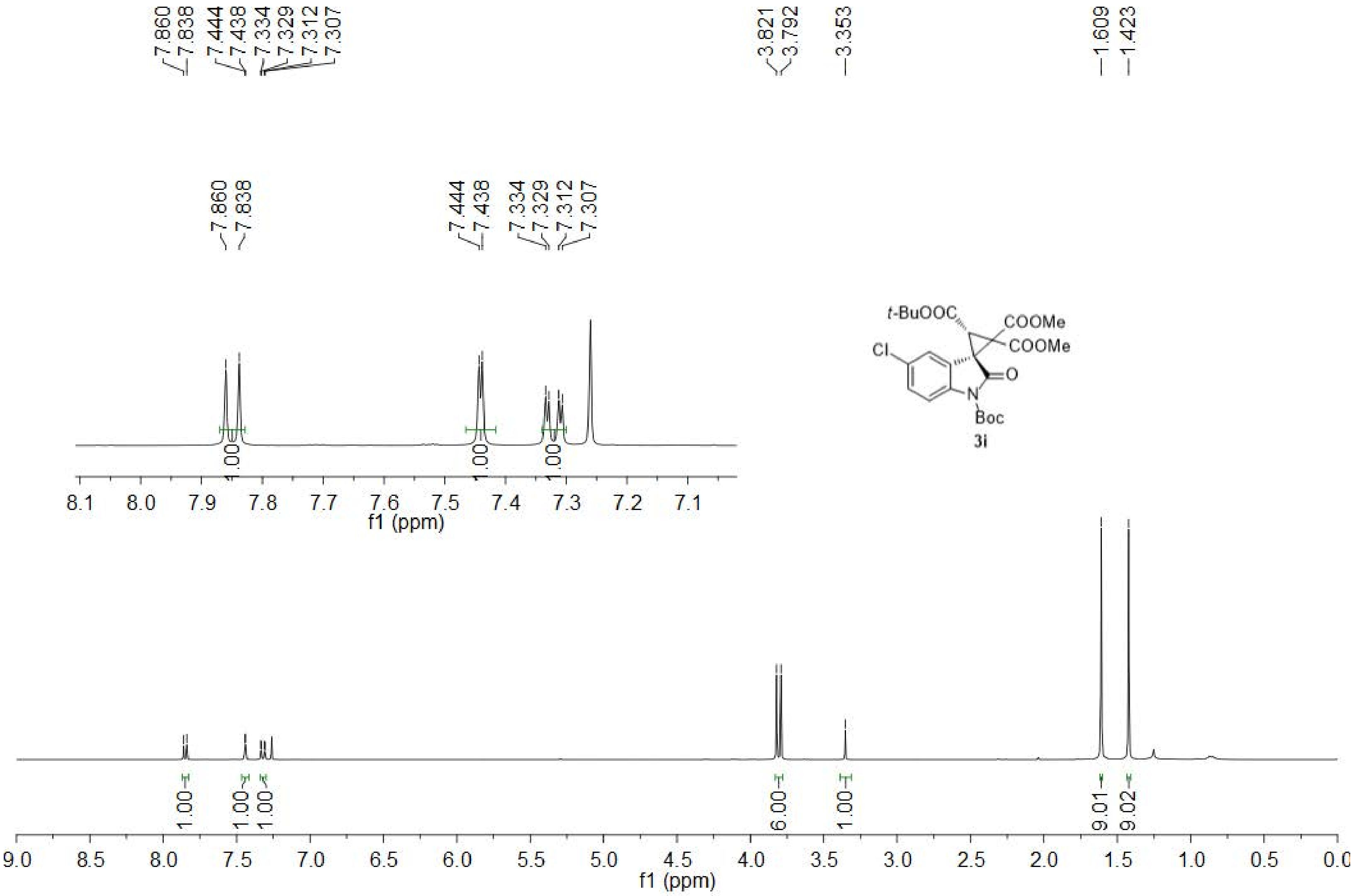
7.860
7.838
7.444
7.438
7.334
7.329
7.312
7.307
3.821
3.792
3.353
1.609
1.423
t-BuOOC
COOMe
COOMe
Cl
O
N
Boc
3i
1.00
1.00
1.00
6.00
1.00
9.01
9.02
f1 (ppm)

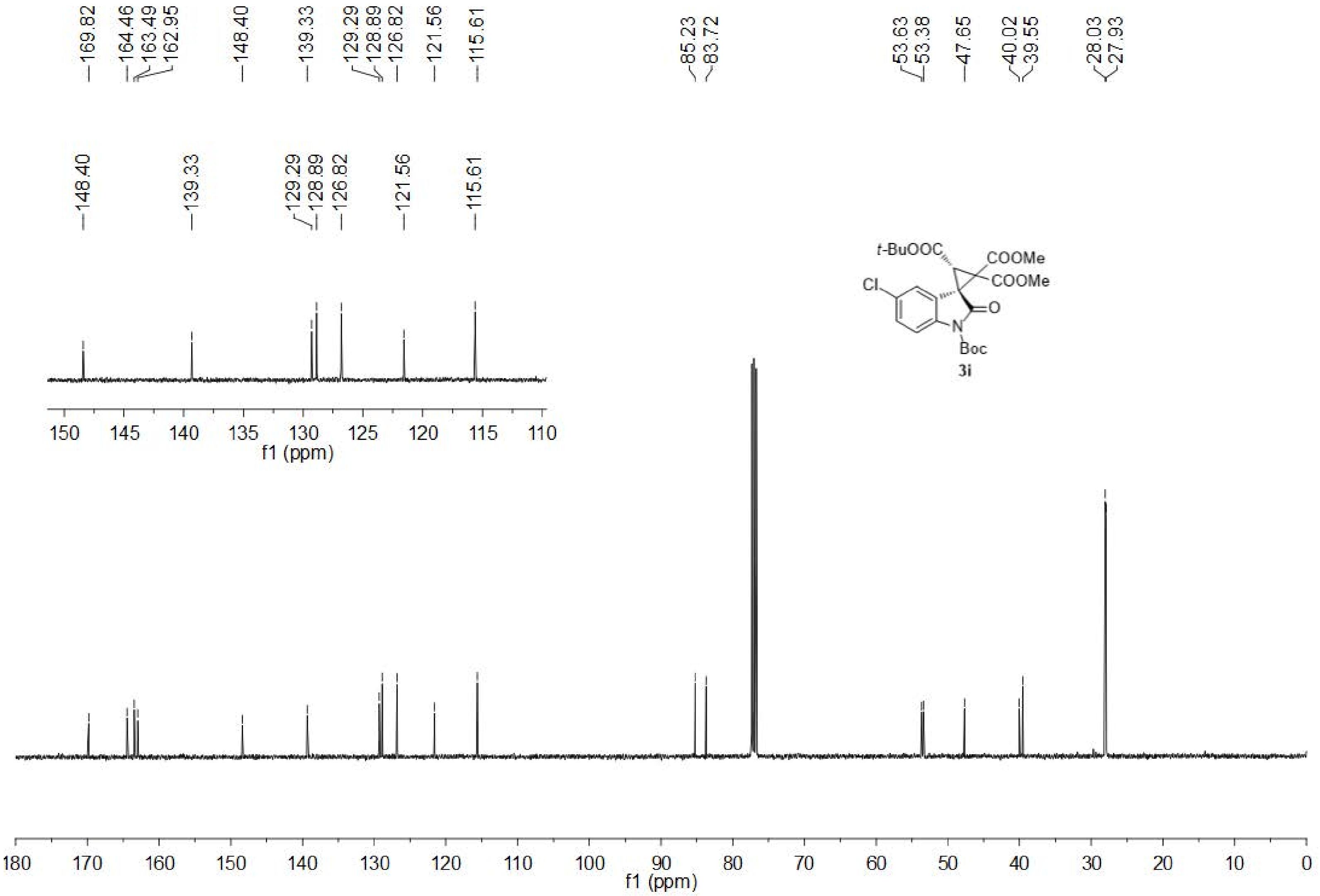
169.82
164.46
163.49
162.95
148.40
139.33
129.29
128.89
126.82
121.56
115.61
85.23
83.72
53.63
53.38
47.65
40.02
39.55
28.03
27.93
t-BuOOC
COOMe
COOMe
Cl
O
N
Boc
3i
150 145 140 135 130 125 120 115 110
f1 (ppm)
180 170 160 150 140 130 120 110 100 90 80 70 60 50 40 30 20 10 0
f1 (ppm)

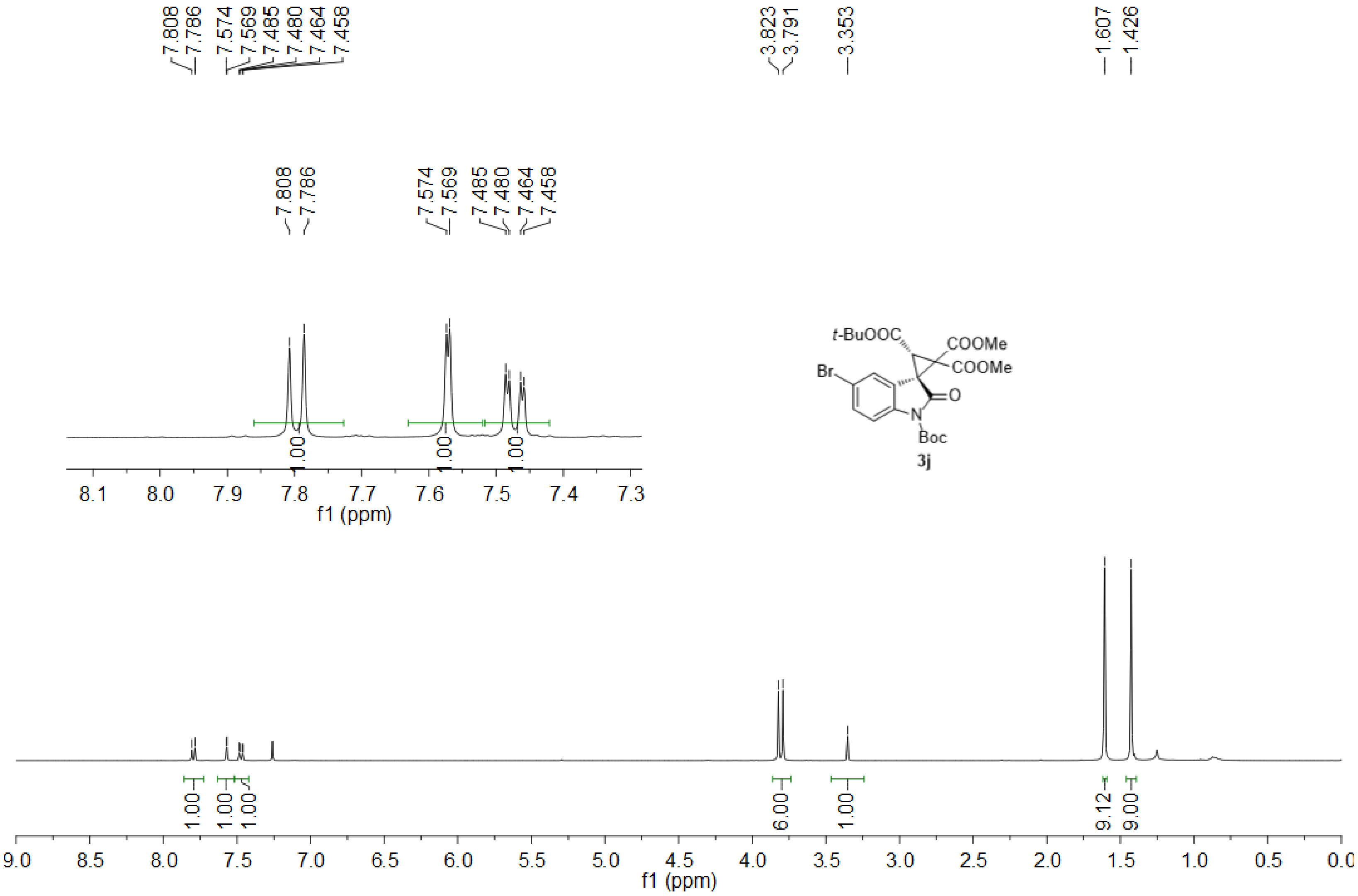
7.808
7.786
7.574
7.569
7.485
7.480
7.464
7.458
3.823
3.791
3.353
1.607
1.426
1.00
1.00
1.00
6.00
1.00
9.12
9.00
f1 (ppm)
t-BuOOC
COOMe
COOMe
Br
N
O
Boc
3j

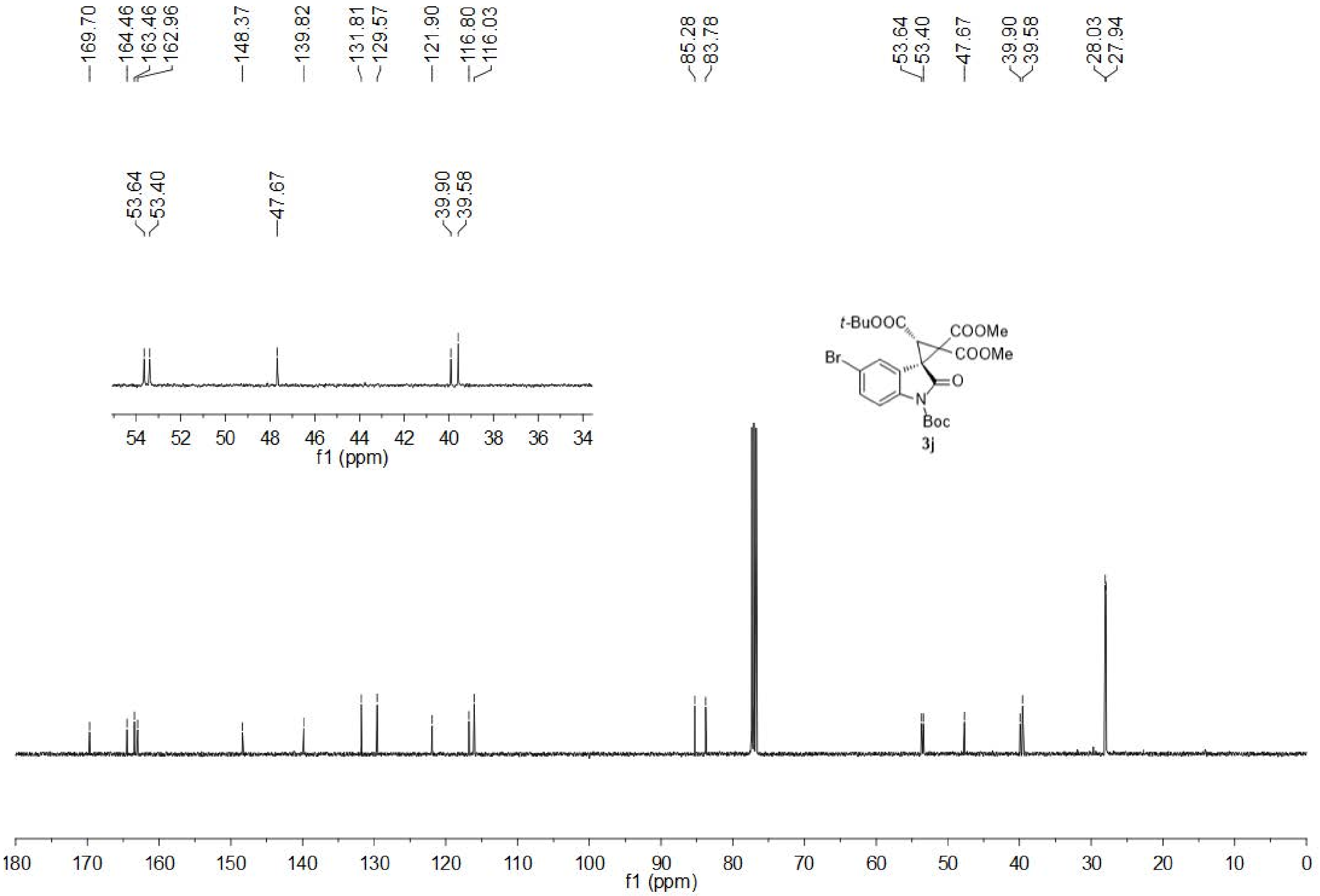
169.70
164.46
163.46
162.96
148.37
139.82
131.81
129.57
121.90
116.80
116.03
85.28
83.78
53.64
53.40
47.67
39.90
39.58
28.03
27.94
53.64
53.40
47.67
39.90
39.58
54 52 50 48 46 44 42 40 38 36 34
f1 (ppm)
t-BuOOC
COOMe
COOMe
Br
O
N
Boc
3j
180 170 160 150 140 130 120 110 100 90 80 70 60 50 40 30 20 10 0
f1 (ppm)

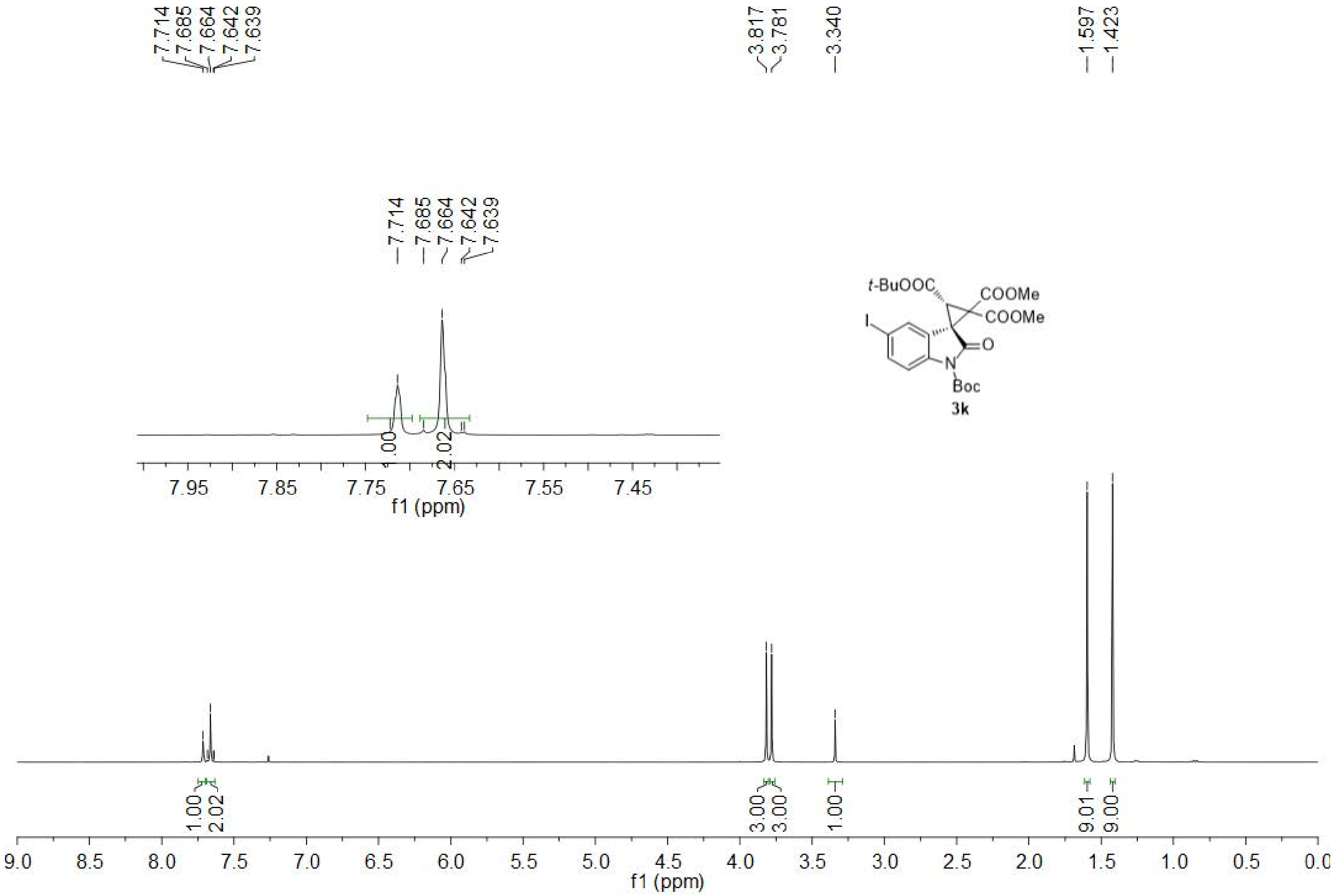
7.714
7.685
7.664
7.642
7.639
3.817
3.781
3.340
1.597
1.423
t-BuOOC
COOMe
COOMe
I
O
N
Boc
3k
1.00
2.02
7.95
7.85
7.75
7.65
7.55
7.45
f1 (ppm)
1.00
2.02
3.00
3.00
1.00
9.01
9.00
9.0
8.5
8.0
7.5
7.0
6.5
6.0
5.5
5.0
4.5
4.0
3.5
3.0
2.5
2.0
1.5
1.0
0.5
0.0
f1 (ppm)

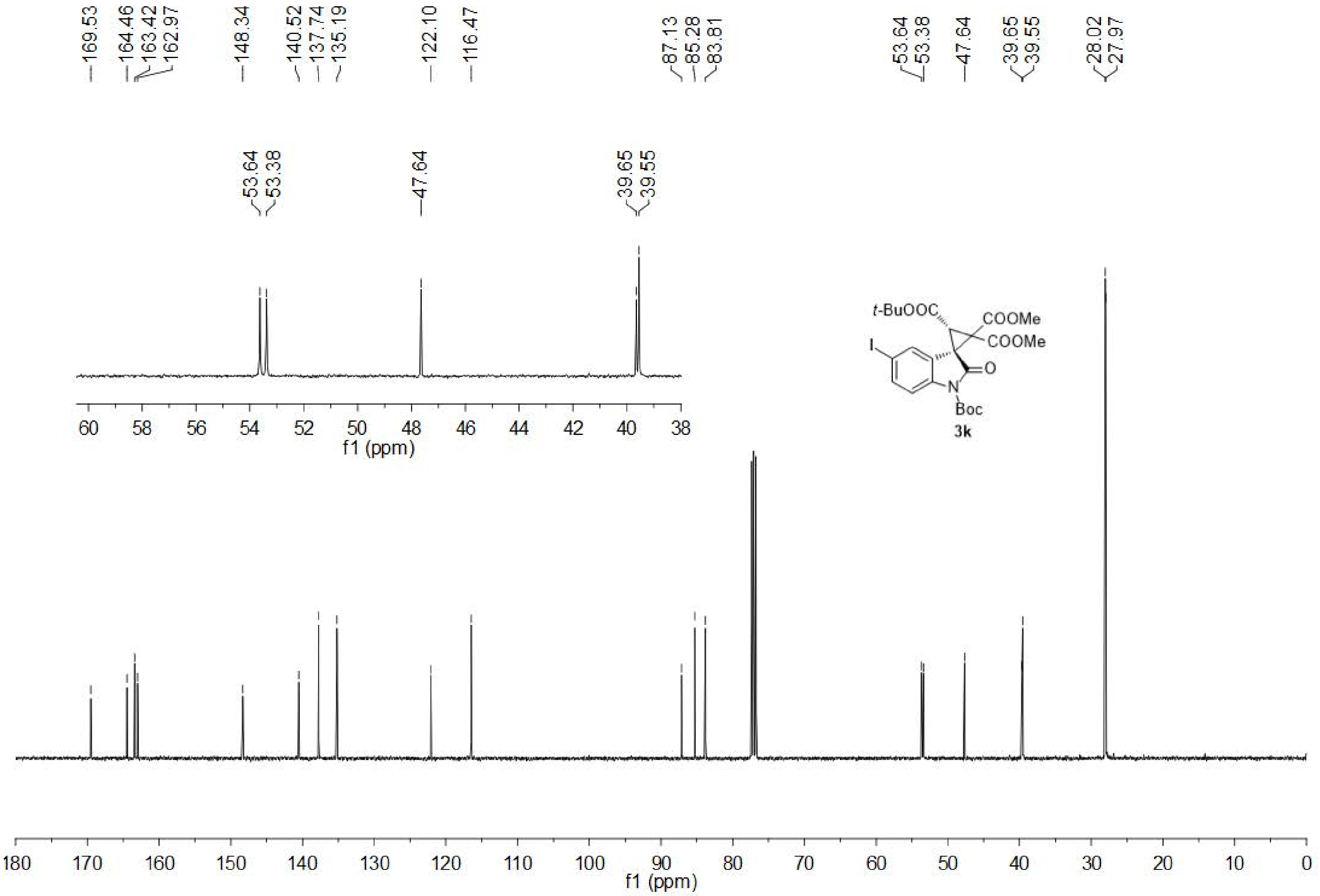
169.53
164.46
163.42
162.97
148.34
140.52
137.74
135.19
122.10
116.47
87.13
85.28
83.81
53.64
53.38
47.64
39.65
39.55
28.02
27.97
t-BuOOC
COOMe
COOMe
I
O
N
Boc
3k
60 58 56 54 52 50 48 46 44 42 40 38
f1 (ppm)
180 170 160 150 140 130 120 110 100 90 80 70 60 50 40 30 20 10 0
f1 (ppm)

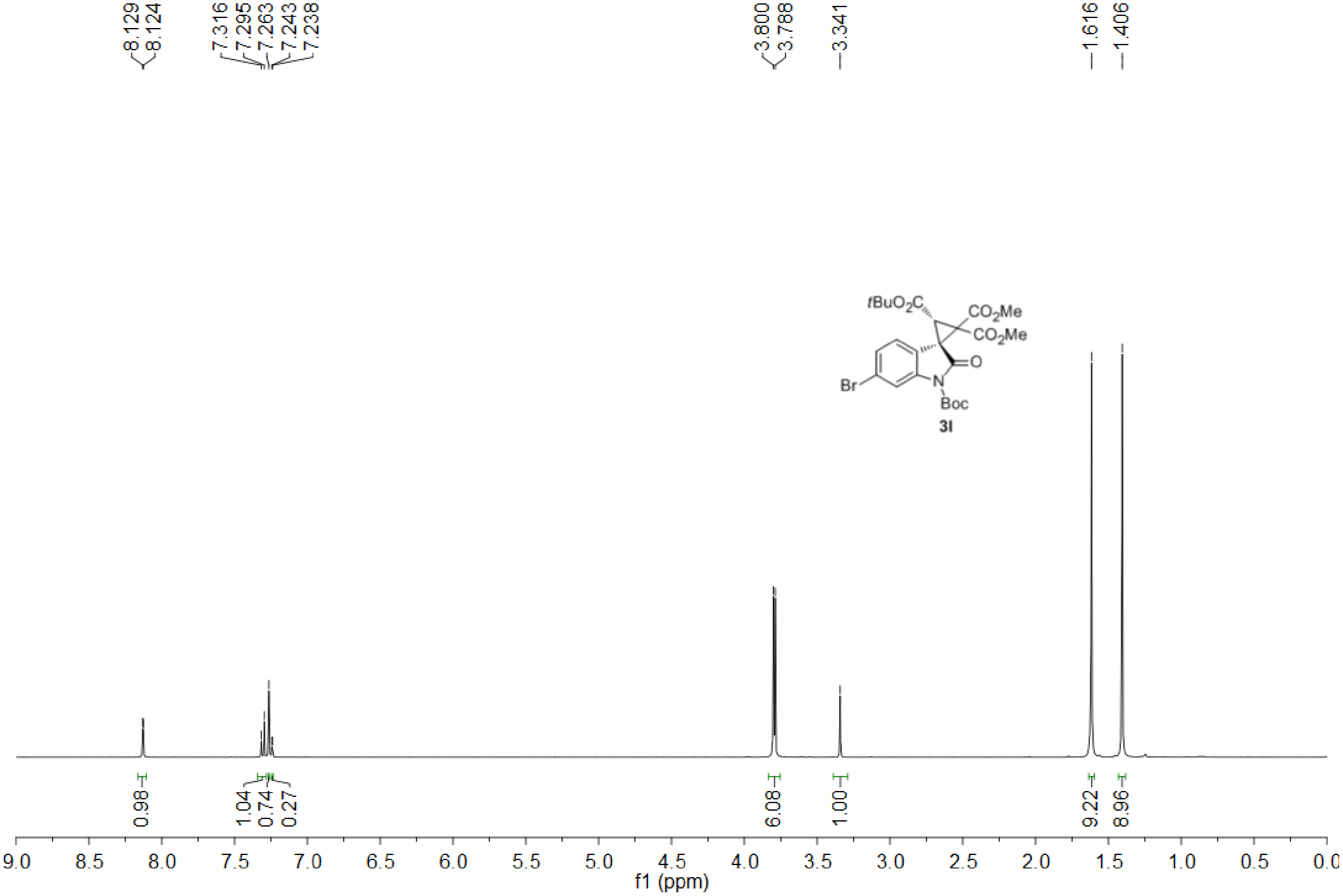

8.129
8.124
7.316
7.295
7.263
7.243
7.238
3.800
3.788
3.341
1.616
1.406
tBuO2C
CO2Me
CO2Me
O
Br
N
Boc
3l
0.98
1.04
0.74
0.27
6.08
1.00
9.22
8.96
9.0 8.5 8.0 7.5 7.0 6.5 6.0 5.5 5.0 4.5 4.0 3.5 3.0 2.5 2.0 1.5 1.0 0.5 0.0
f1 (ppm)

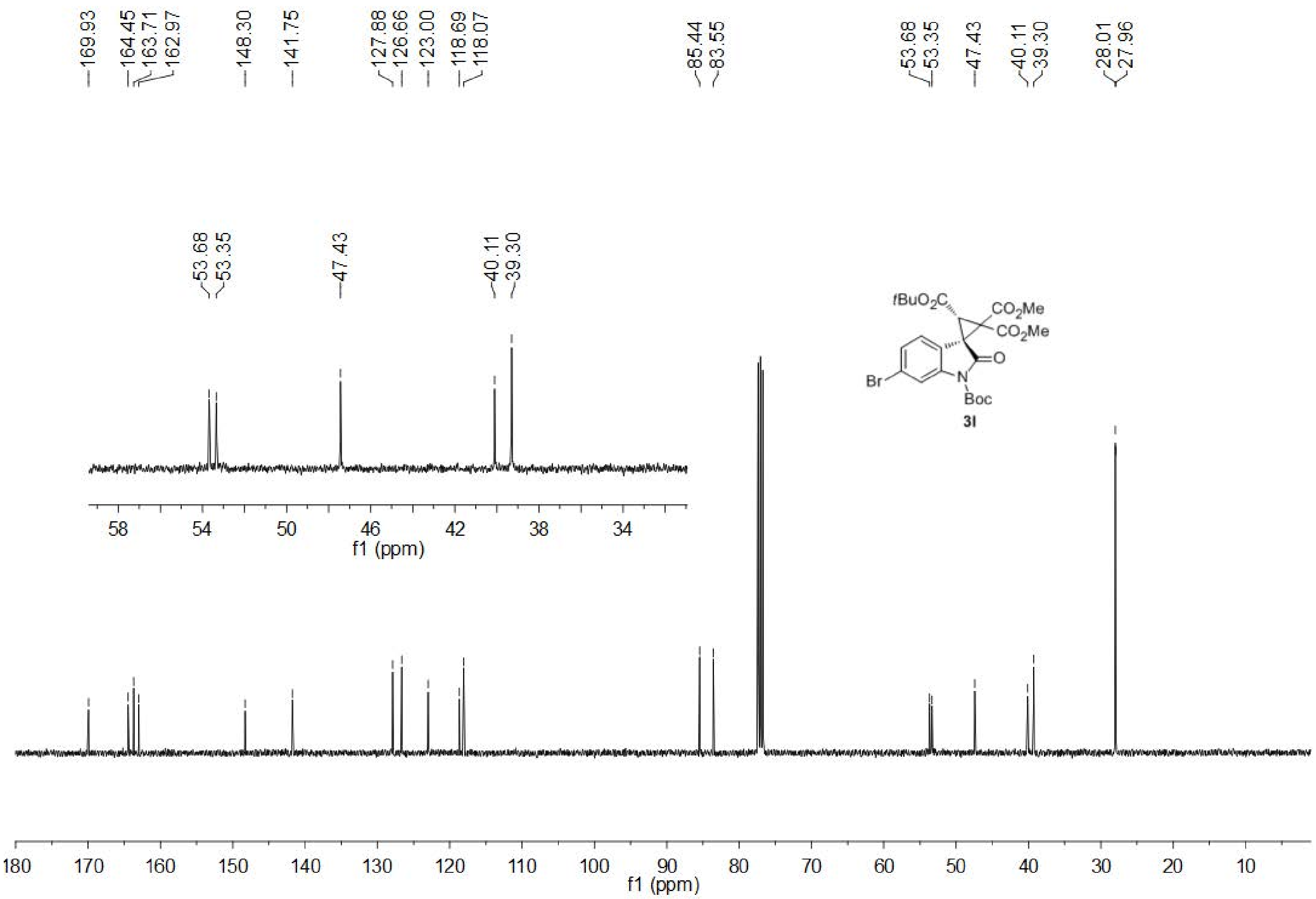
169.93
164.45
163.71
162.97
148.30
141.75
127.88
126.66
123.00
118.69
118.07
85.44
83.55
53.68
53.35
47.43
40.11
39.30
28.01
27.96
tBuO2C
CO2Me
CO2Me
Br
N
Boc
O
3l
58
54
50
46
42
38
34
f1 (ppm)
180
170
160
150
140
130
120
110
100
90
80
70
60
50
40
30
20
10
f1 (ppm)

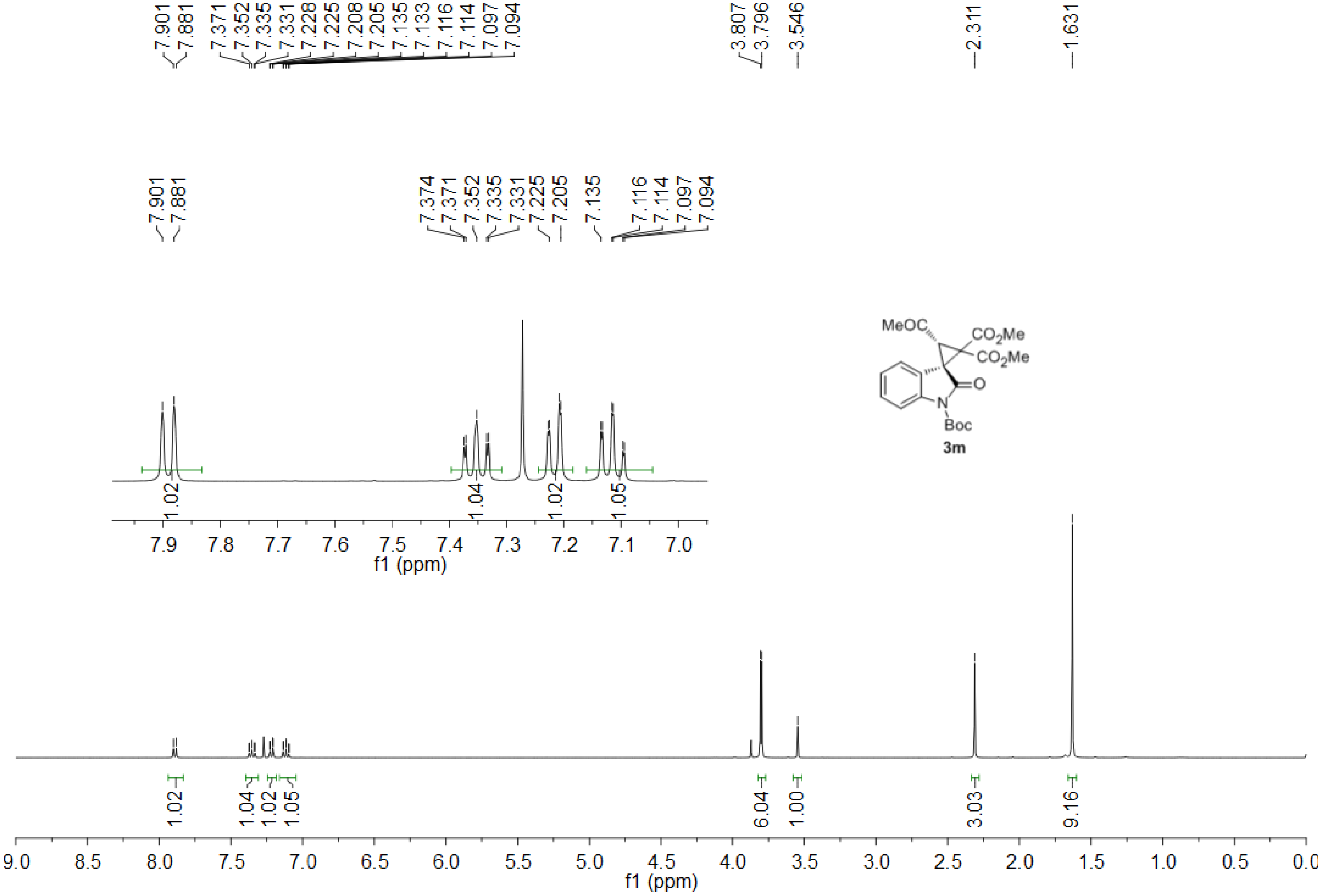

7.901
7.881
7.371
7.352
7.335
7.331
7.228
7.225
7.208
7.205
7.135
7.133
7.116
7.114
7.097
7.094
3.807
3.796
3.546
2.311
1.631
7.374
MeOC
CO2Me
CO2Me
O
N
Boc
3m
1.02
1.04
1.02
1.05
6.04
1.00
3.03
9.16
f1 (ppm)

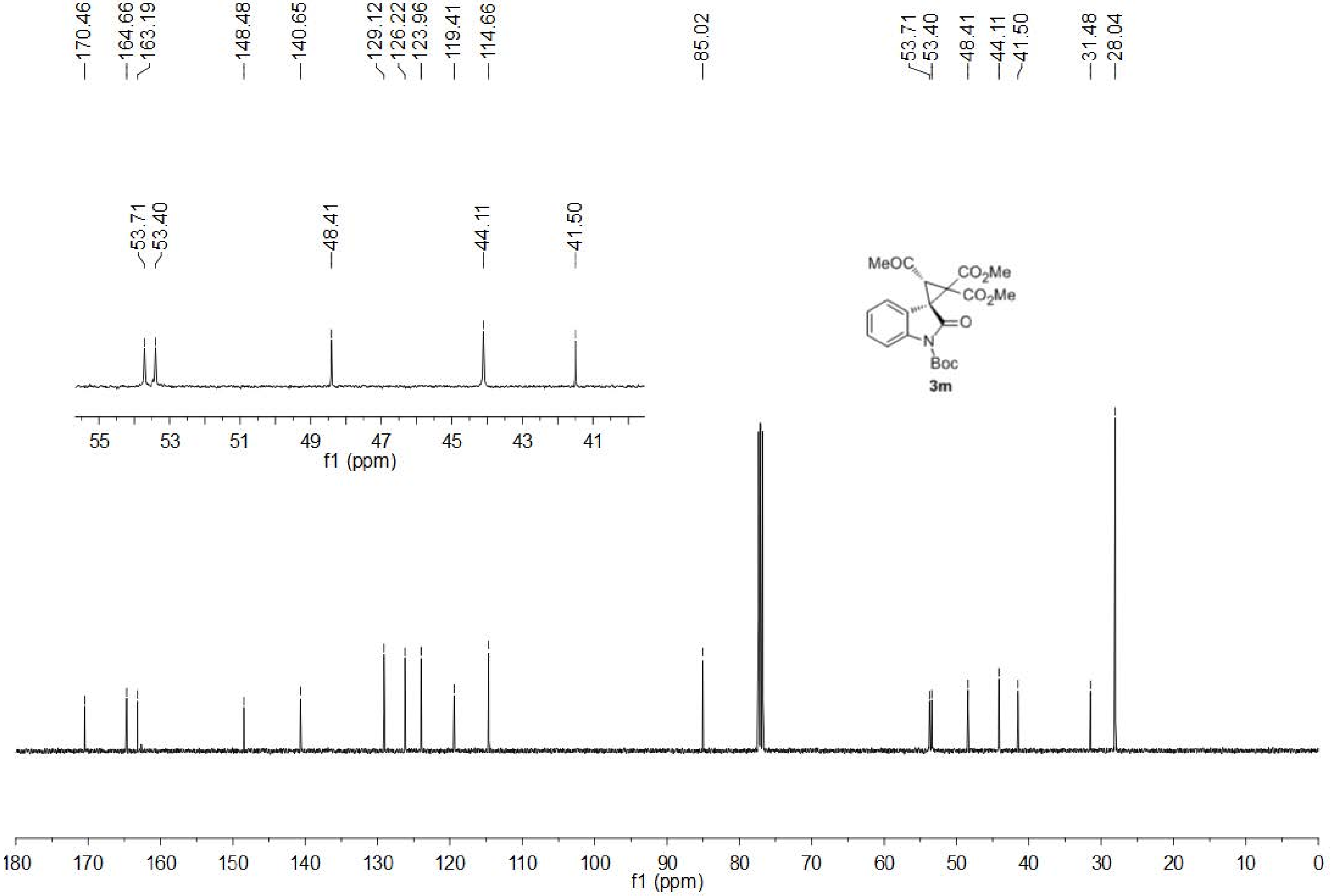
170.46
164.66
163.19
148.48
140.65
129.12
126.22
123.96
119.41
114.66
85.02
53.71
53.40
48.41
44.11
41.50
31.48
28.04
MeOC
CO2Me
CO2Me
O
N
Boc
3m
55 53 51 49 47 45 43 41
f1 (ppm)
180 170 160 150 140 130 120 110 100 90 80 70 60 50 40 30 20 10 0
f1 (ppm)

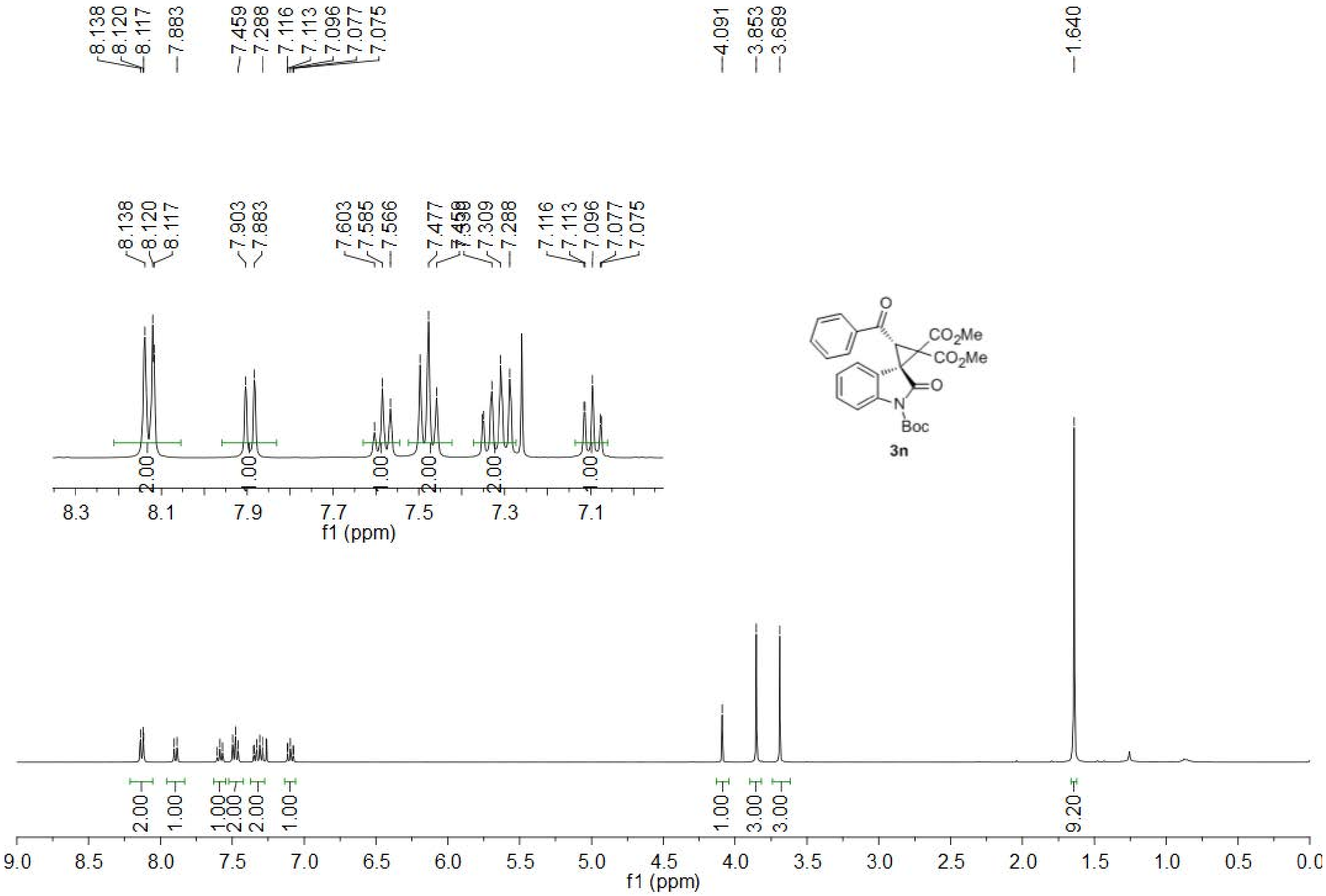

8.138
8.120
8.117
7.883
7.459
7.288
7.116
7.113
7.096
7.077
7.075
4.091
3.853
3.689
1.640
8.138
8.120
8.117
7.903
7.883
7.603
7.585
7.566
7.477
7.309
7.288
7.116
7.113
7.096
7.077
7.075
2.00
1.00
1.00
2.00
2.00
1.00
8.3
8.1
7.9
7.7
7.5
7.3
7.1
f1 (ppm)
CO_2Me
CO_2Me
O
O
N
Boc
3n
2.00
1.00
1.00
2.00
2.00
1.00
1.00
3.00
3.00
9.20
9.0
8.5
8.0
7.5
7.0
6.5
6.0
5.5
5.0
4.5
4.0
3.5
3.0
2.5
2.0
1.5
1.0
0.5
0.0
f1 (ppm)

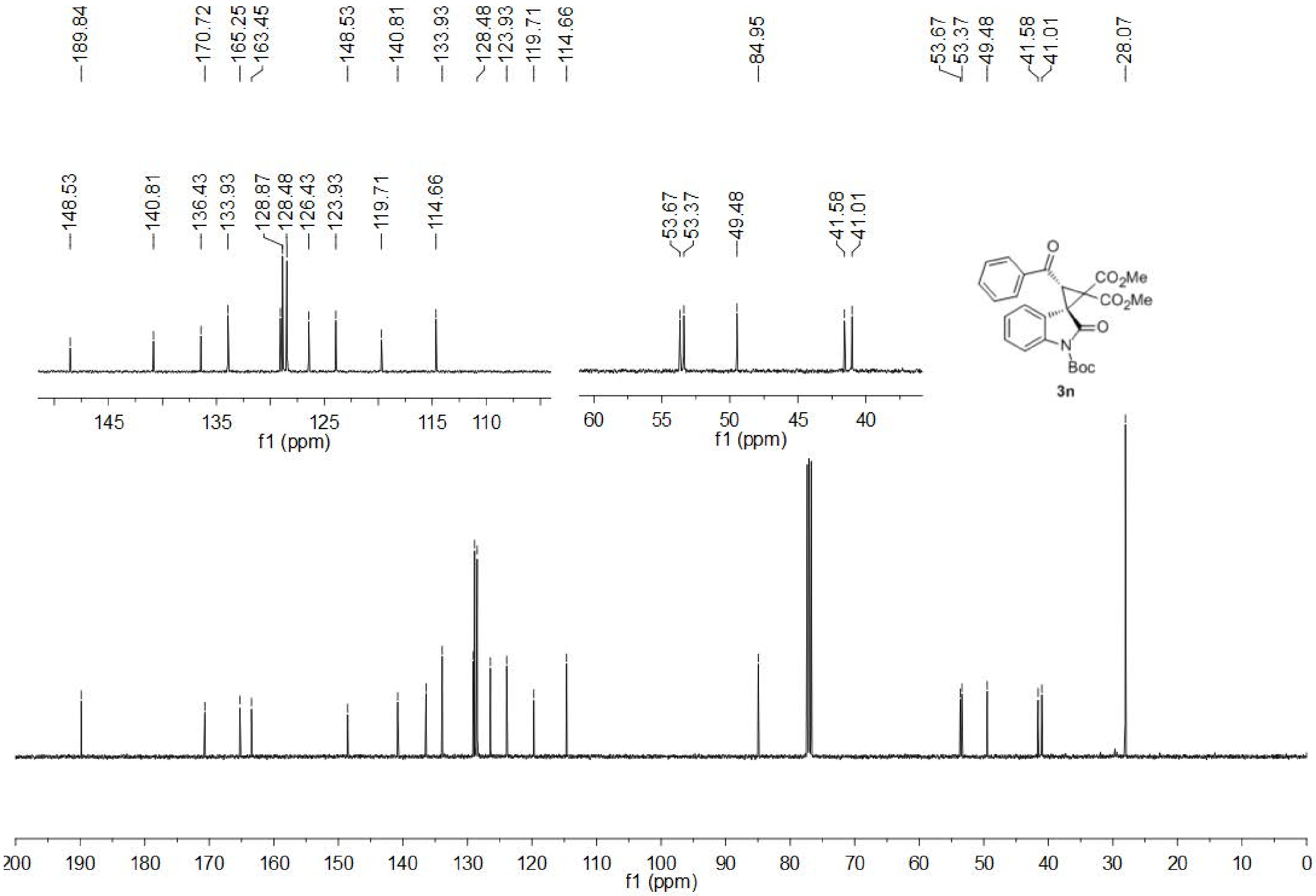

189.84
170.72
165.25
163.45
148.53
140.81
133.93
128.48
123.93
119.71
114.66
84.95
53.67
53.37
49.48
41.58
41.01
28.07
148.53
140.81
136.43
133.93
128.87
128.48
126.43
123.93
119.71
114.66
53.67
53.37
49.48
41.58
41.01
O
CO2Me
CO2Me
O
N
Boc
3n
f1 (ppm)

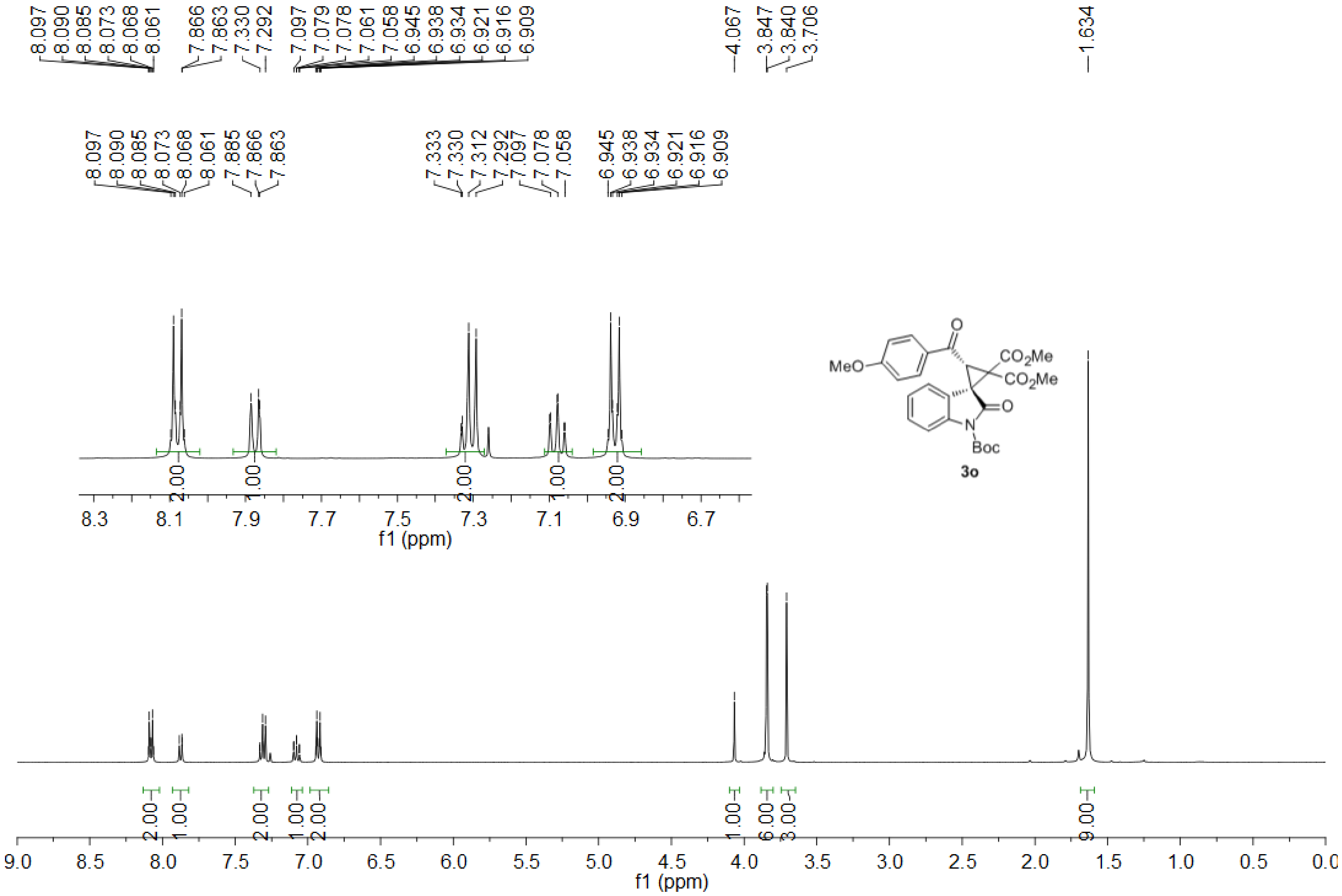

8.097
8.090
8.085
8.073
8.068
8.061
7.866
7.863
7.330
7.292
7.097
7.079
7.078
7.061
7.058
6.945
6.938
6.934
6.921
6.916
6.909
4.067
3.847
3.840
3.706
1.634
8.097
8.090
8.085
8.073
8.068
8.061
7.885
7.866
7.863
7.333
7.330
7.312
7.292
7.097
7.078
7.058
6.945
6.938
6.934
6.921
6.916
6.909
2.00
1.00
2.00
1.00
2.00
f1 (ppm)
MeO
O
CO2Me
CO2Me
O
N
Boc
2.00
1.00
2.00
1.00
2.00
1.00
6.00
3.00
9.00
f1 (ppm)

3o

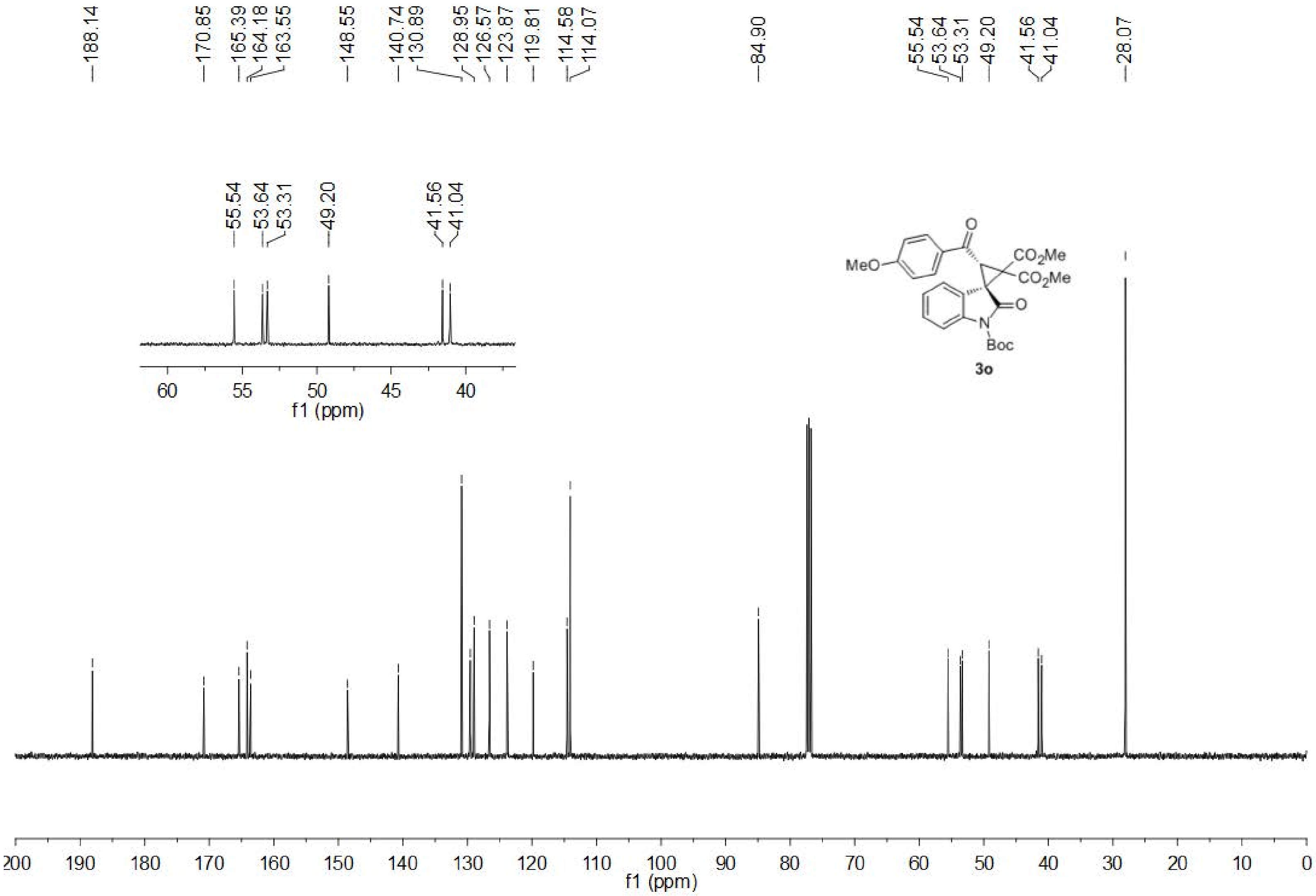
188.14
170.85
165.39
164.18
163.55
148.55
140.74
130.89
128.95
126.57
123.87
119.81
114.58
114.07
84.90
55.54
53.64
53.31
49.20
41.56
41.04
28.07
MeO
O
CO2Me
CO2Me
N
O
Boc
3o
200 190 180 170 160 150 140 130 120 110 100 90 80 70 60 50 40 30 20 10 0
f1 (ppm)
60 55 50 45 40
f1 (ppm)

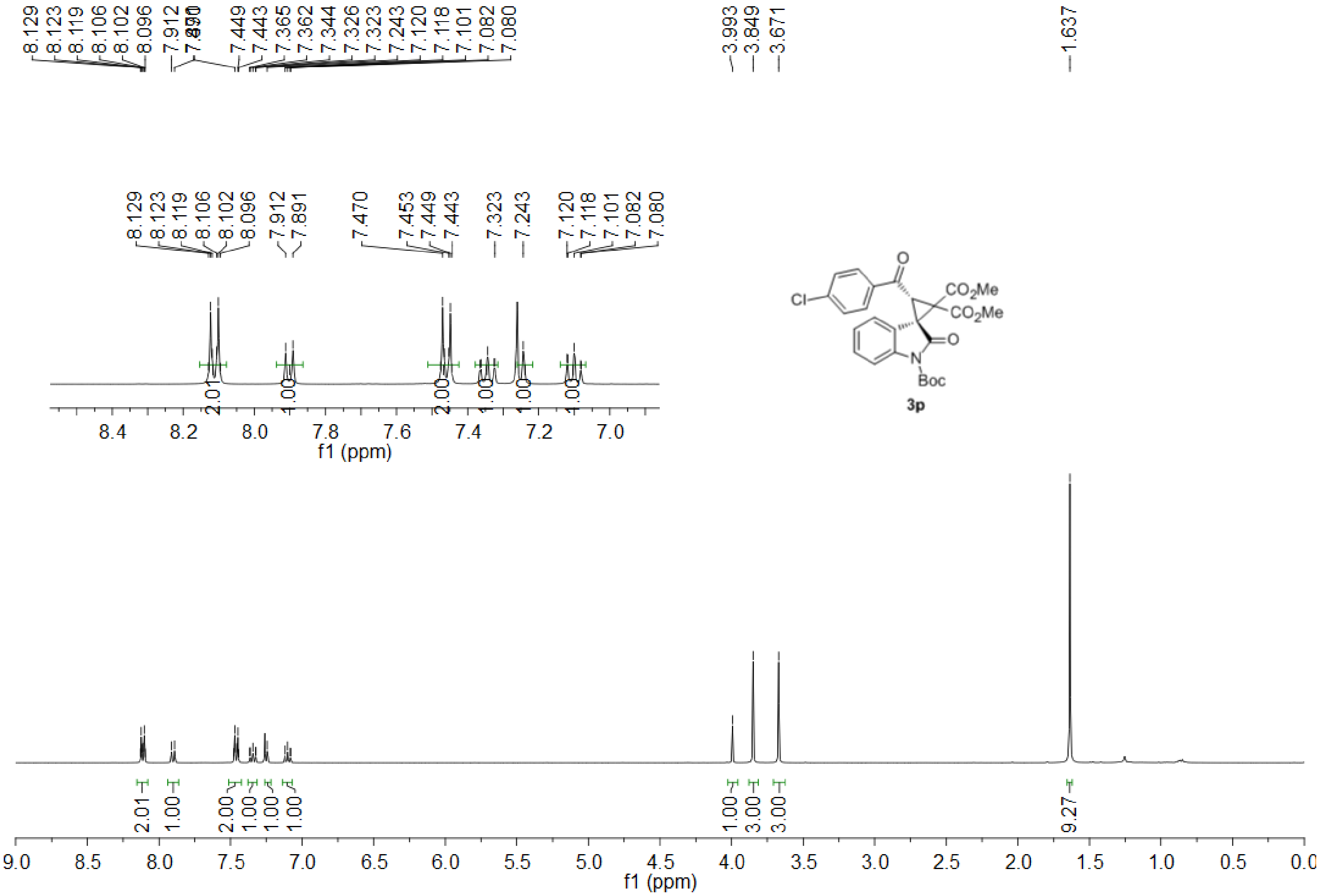
8.129
8.123
8.119
8.106
8.102
8.096
7.912
7.891
7.449
7.443
7.365
7.362
7.344
7.326
7.323
7.243
7.120
7.118
7.101
7.082
7.080
3.993
3.849
3.671
1.637
7.470
7.453
Cl
O
CO2Me
CO2Me
N
Boc
3p
2.01
1.00
2.00
1.00
1.00
1.00
1.00
3.00
3.00
9.27
f1 (ppm)

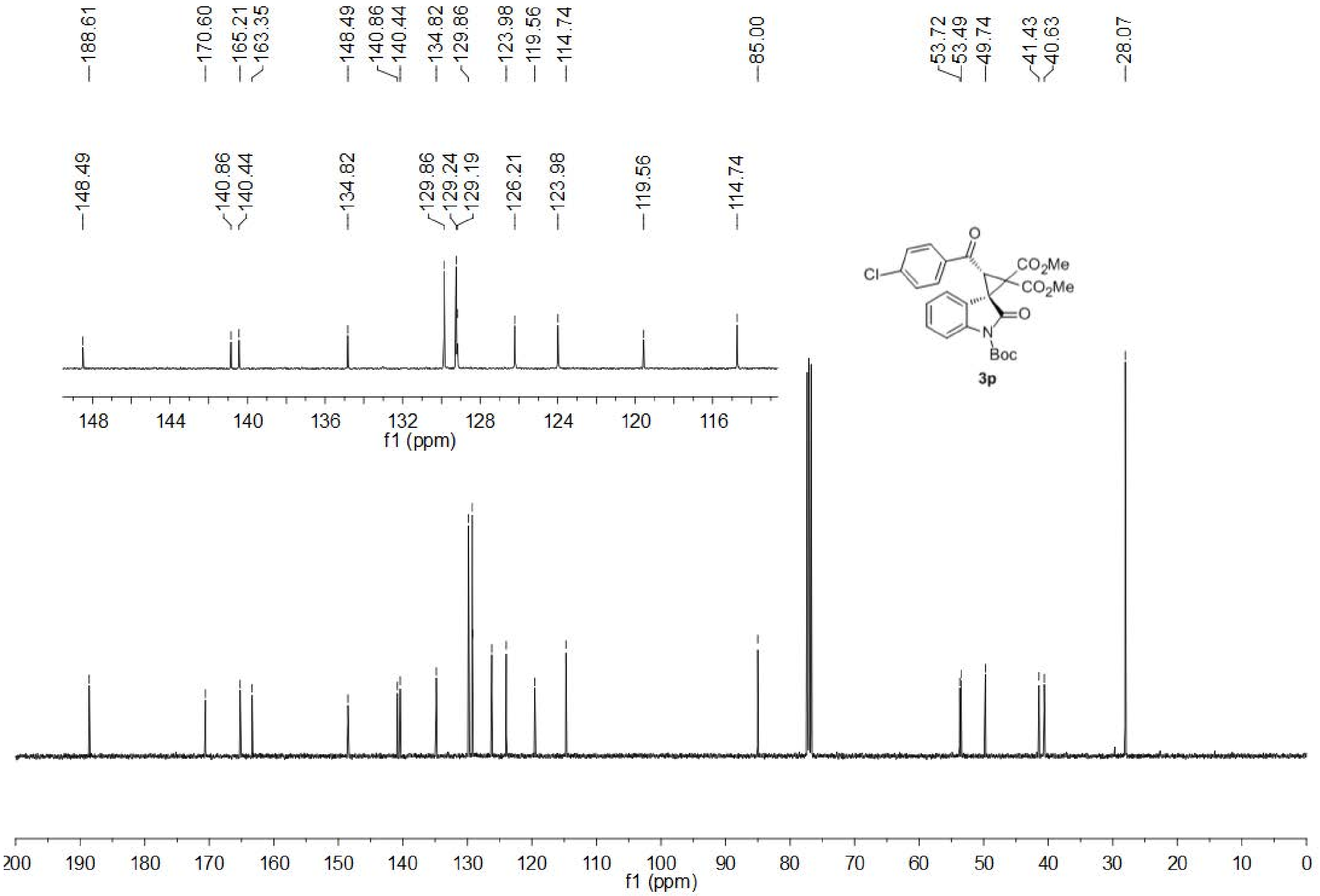

188.61
170.60
165.21
163.35
148.49
140.86
140.44
134.82
129.86
123.98
119.56
114.74
85.00
53.72
53.49
49.74
41.43
40.63
28.07
148.49
140.86
140.44
134.82
129.86
129.24
129.19
126.21
123.98
119.56
114.74
148
144
140
136
132
128
124
120
116
f1 (ppm)
Cl
O
CO2Me
CO2Me
O
N
Boc
200
190
180
170
160
150
140
130
120
110
100
90
80
70
60
50
40
30
20
10
0
f1 (ppm)

3p

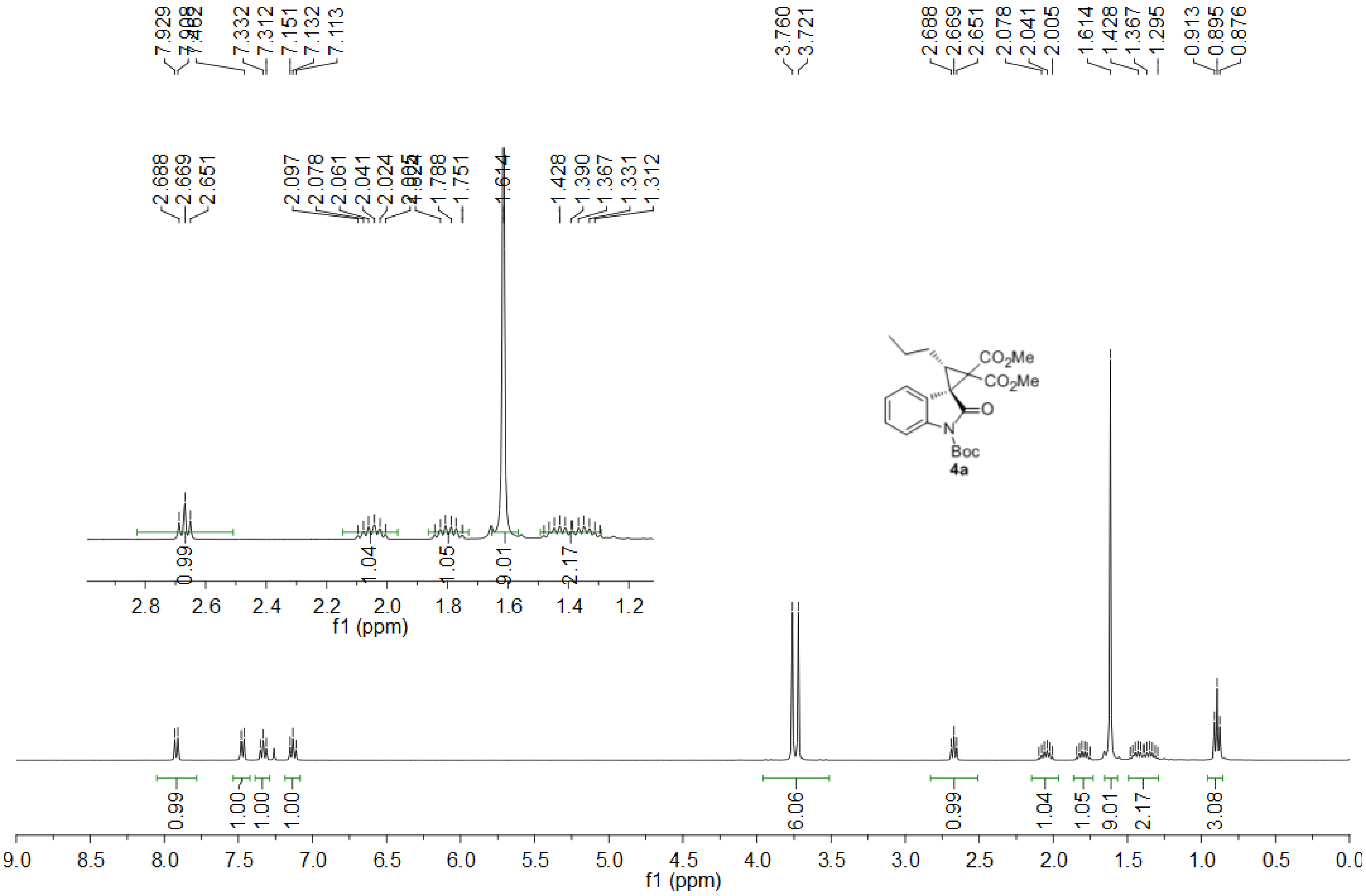

7.929
7.908
7.482
7.332
7.312
7.151
7.132
7.113
3.760
3.721
2.688
2.669
2.651
2.078
2.041
2.005
1.614
1.428
1.367
1.295
0.913
0.895
0.876
2.688
2.669
2.651
2.097
2.078
2.061
2.041
2.024
1.614
1.428
1.390
1.367
1.331
1.312
0.99
1.04
1.05
9.01
2.17
f1 (ppm)
CO2Me
CO2Me
O
N
Boc
0.99
1.00
1.00
1.00
6.06
0.99
1.04
1.05
9.01
2.17
3.08
f1 (ppm)

4a

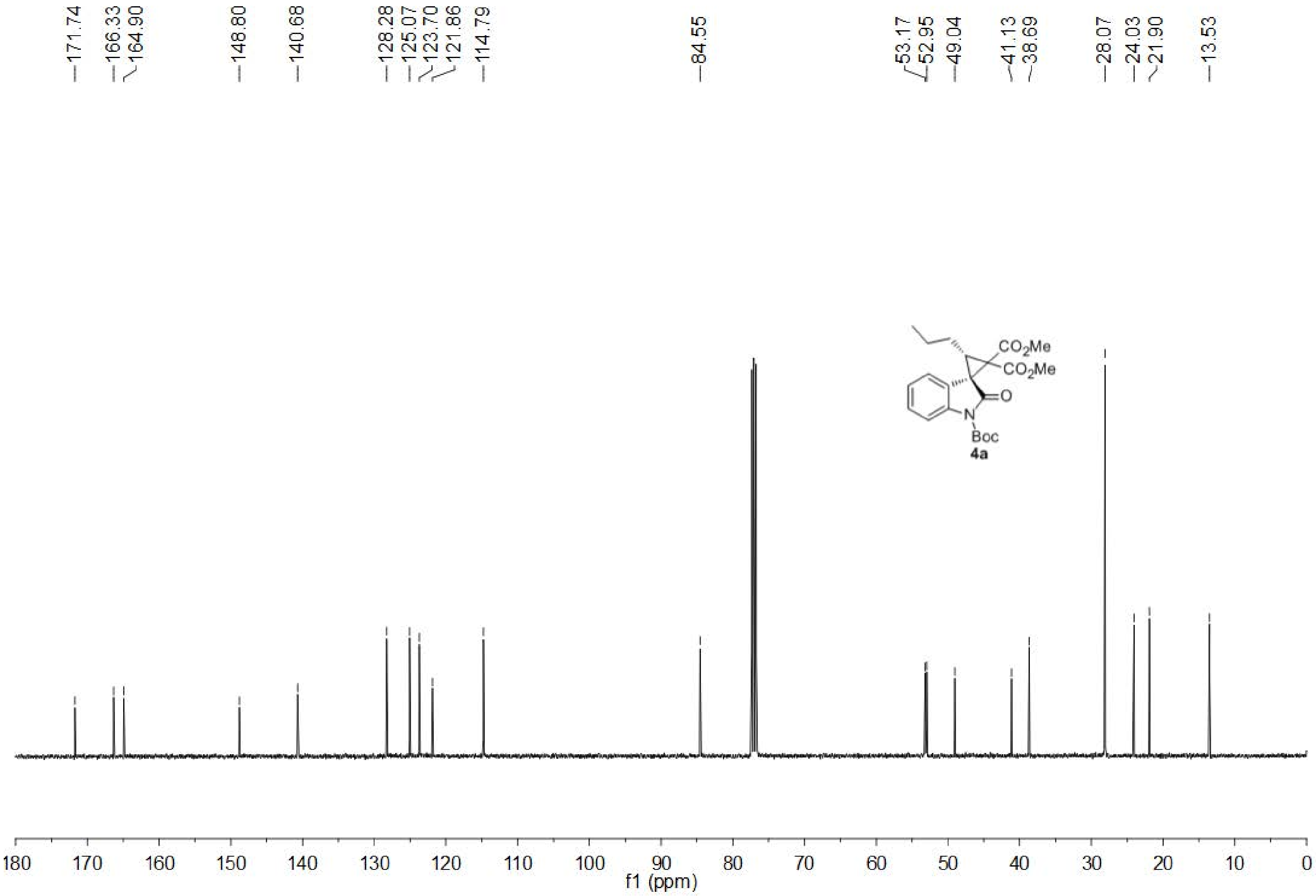
171.74
166.33
164.90
148.80
140.68
128.28
125.07
123.70
121.86
114.79
84.55
53.17
52.95
49.04
41.13
38.69
28.07
24.03
21.90
13.53
CO2Me
CO2Me
O
N
Boc
4a
180 170 160 150 140 130 120 110 100 90 80 70 60 50 40 30 20 10 0
f1 (ppm)

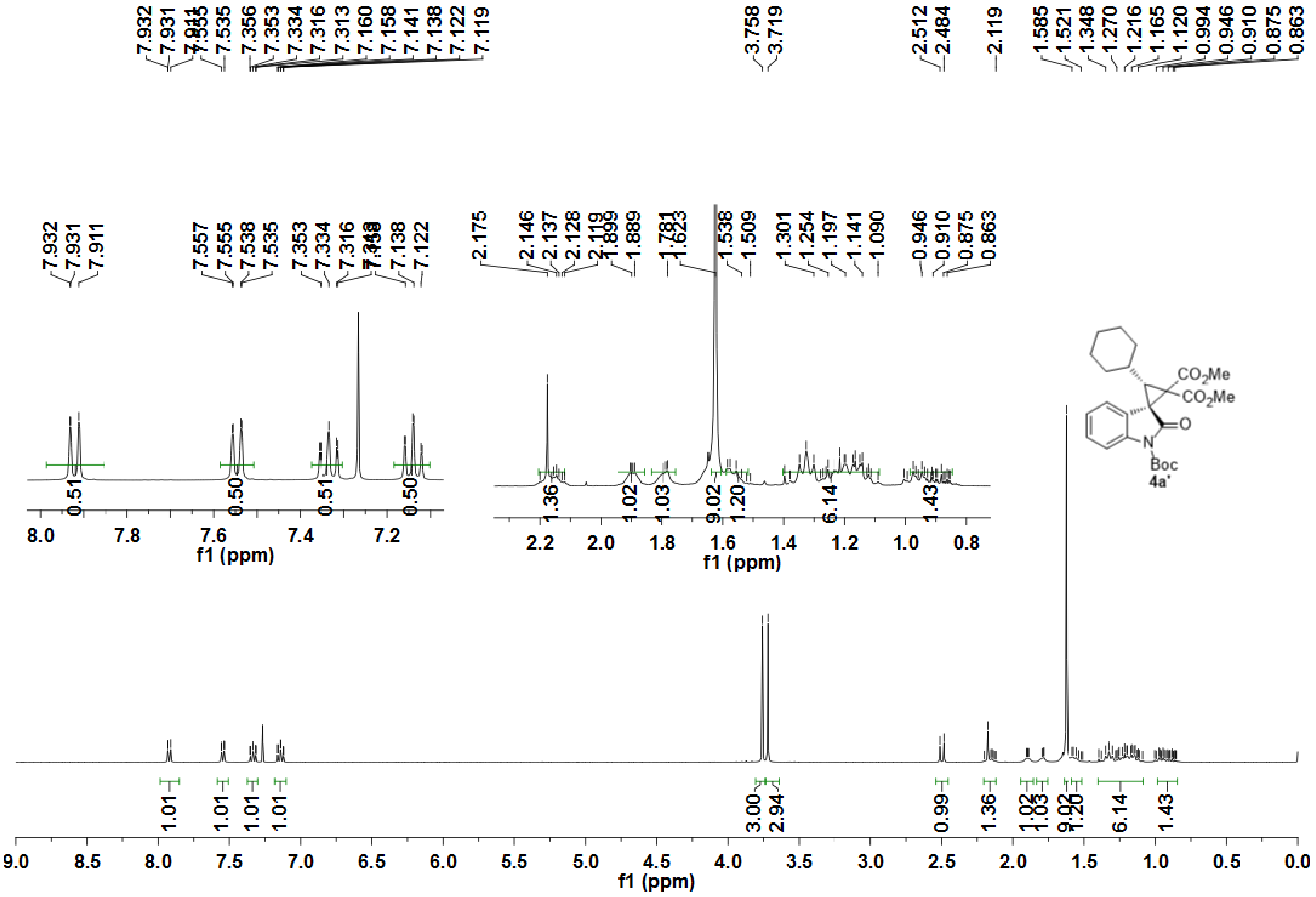

CO2Me
CO2Me
N
O
Boc
4a'
f1 (ppm)

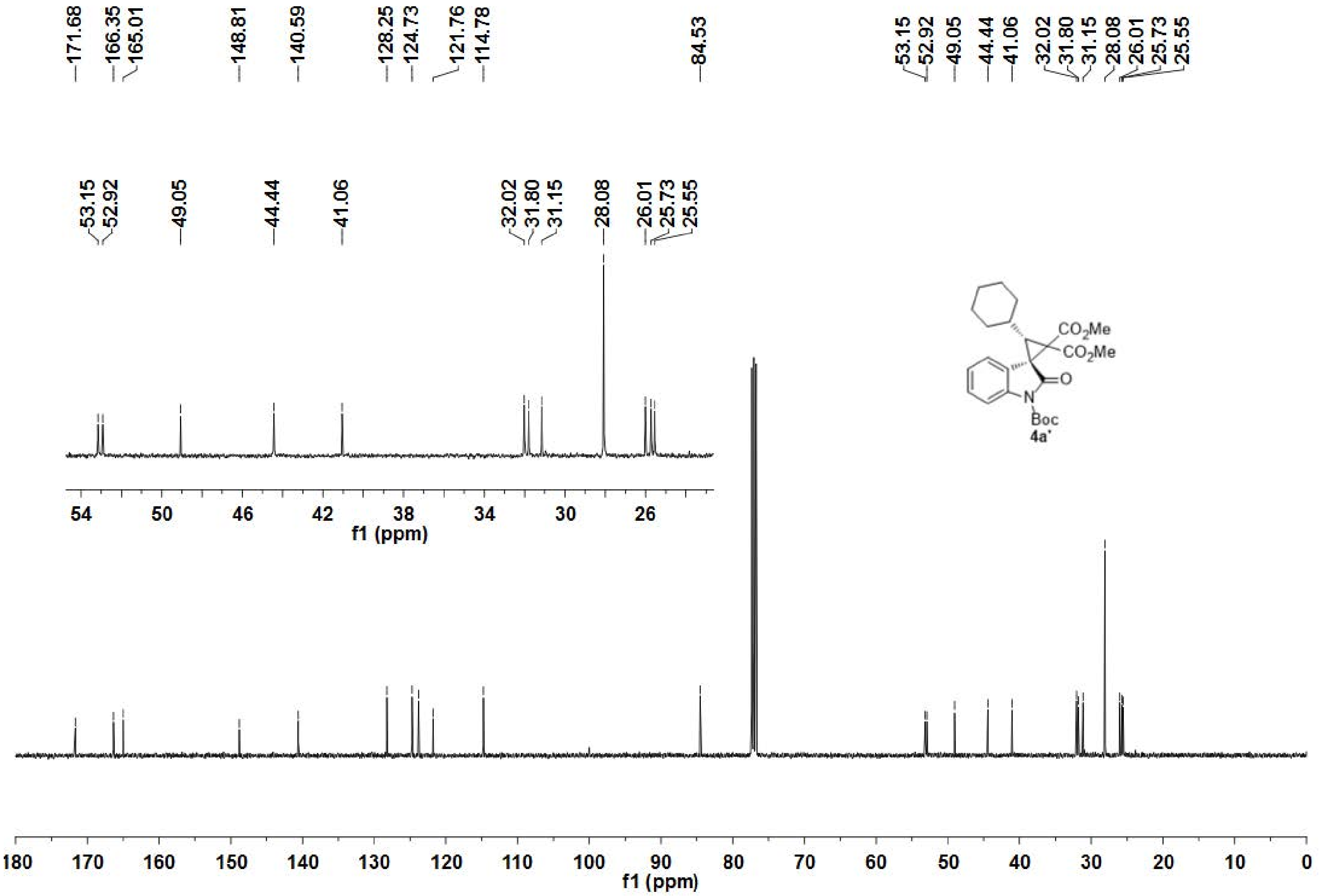
171.68
166.35
165.01
148.81
140.59
128.25
124.73
121.76
114.78
84.53
53.15
52.92
49.05
44.44
41.06
32.02
31.80
31.15
28.08
26.01
25.73
25.55
CO2Me
CO2Me
O
N
Boc
4a'
54 50 46 42 38 34 30 26
f1 (ppm)
180 170 160 150 140 130 120 110 100 90 80 70 60 50 40 30 20 10 0
f1 (ppm)

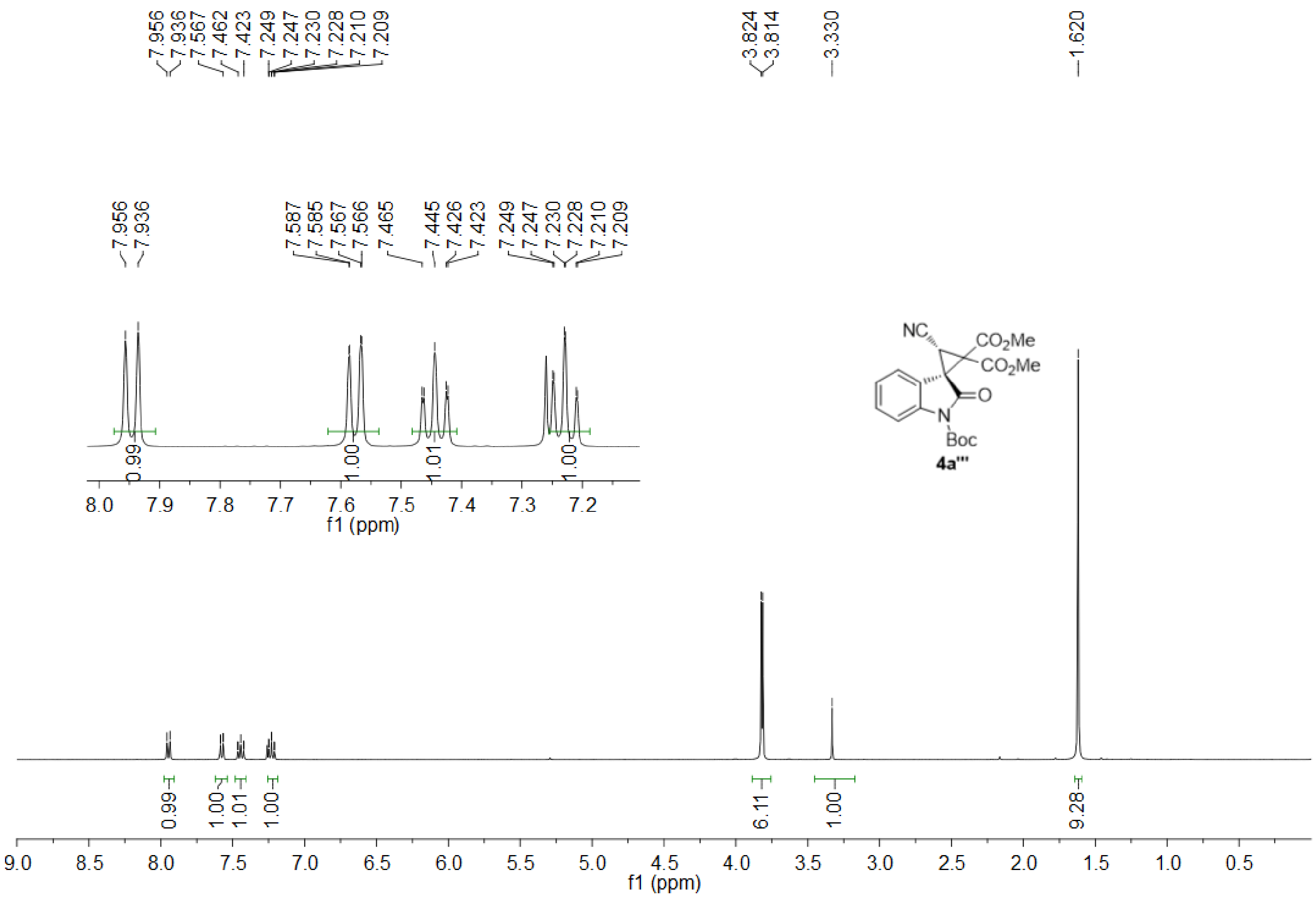

7.956
7.936
7.567
7.462
7.423
7.249
7.247
7.230
7.228
7.210
7.209
3.824
3.814
3.330
1.620
7.956
7.936
7.587
7.585
7.567
7.566
7.465
7.445
7.426
7.423
7.249
7.247
7.230
7.228
7.210
7.209
0.99
1.00
1.01
1.00
8.0 7.9 7.8 7.7 7.6 7.5 7.4 7.3 7.2
f1 (ppm)
NC
CO_2Me
CO_2Me
O
N
Boc
4a'''
0.99
1.00
1.01
1.00
6.11
1.00
9.28
9.0 8.5 8.0 7.5 7.0 6.5 6.0 5.5 5.0 4.5 4.0 3.5 3.0 2.5 2.0 1.5 1.0 0.5
f1 (ppm)

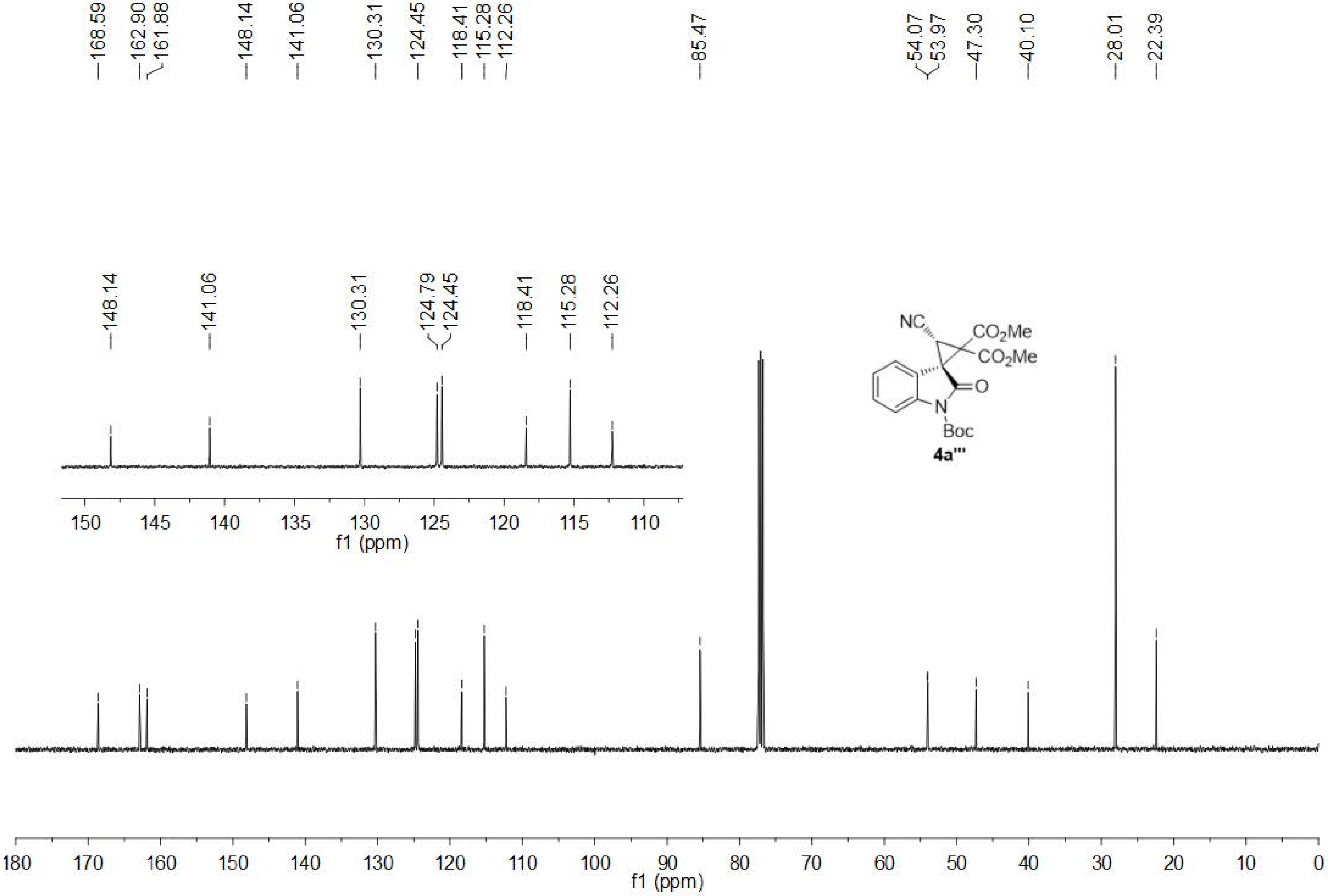

168.59
162.90
161.88
148.14
141.06
130.31
124.45
118.41
115.28
112.26
85.47
54.07
53.97
47.30
40.10
28.01
22.39
148.14
141.06
130.31
124.79
124.45
118.41
115.28
112.26
150 145 140 135 130 125 120 115 110
f1 (ppm)
NC
CO2Me
CO2Me
O
N
Boc
4a'''
180 170 160 150 140 130 120 110 100 90 80 70 60 50 40 30 20 10 0
f1 (ppm)

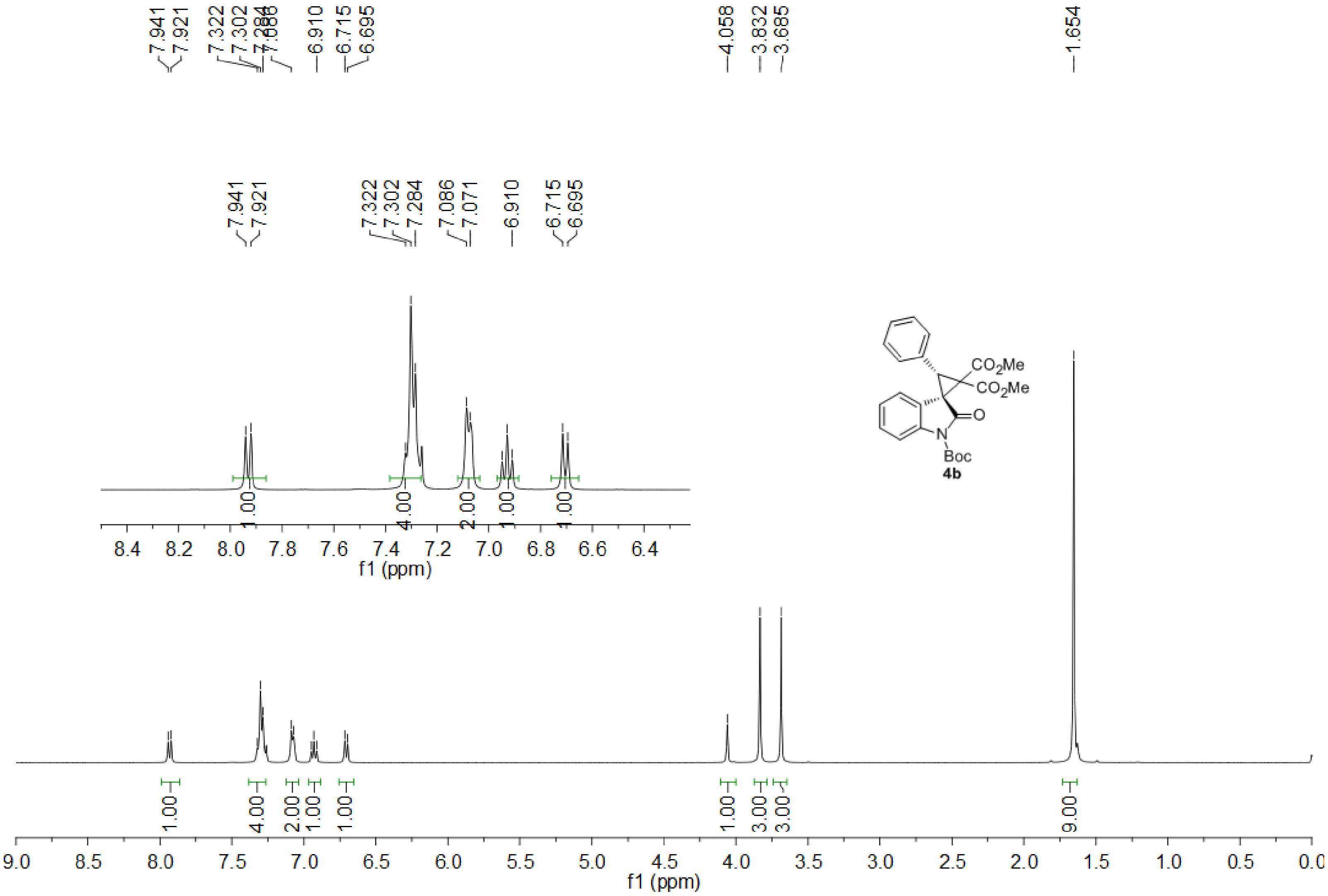

7.941
7.921
7.322
7.302
7.284
7.086
7.071
6.910
6.715
6.695
4.058
3.832
3.685
1.654
CO2Me
CO2Me
N
O
Boc
4b
1.00
4.00
2.00
1.00
1.00
3.00
3.00
9.00
8.4 8.2 8.0 7.8 7.6 7.4 7.2 7.0 6.8 6.6 6.4
f1 (ppm)
9.0 8.5 8.0 7.5 7.0 6.5 6.0 5.5 5.0 4.5 4.0 3.5 3.0 2.5 2.0 1.5 1.0 0.5 0.0
f1 (ppm)

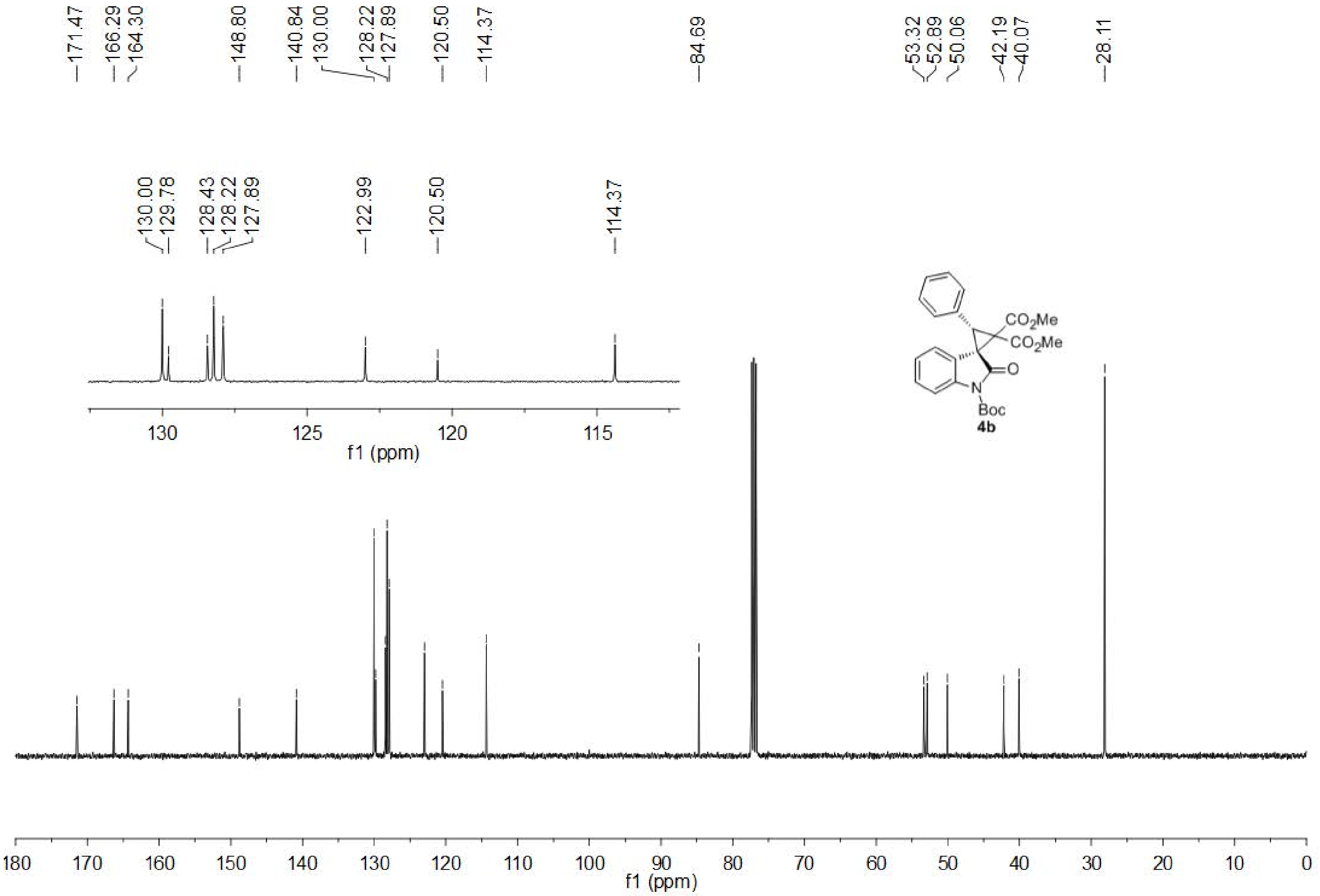
171.47
166.29
164.30
148.80
140.84
130.00
128.22
127.89
120.50
114.37
84.69
53.32
52.89
50.06
42.19
40.07
28.11
130.00
129.78
128.43
128.22
127.89
122.99
120.50
114.37
130
125
120
115
f1 (ppm)
CO2Me
CO2Me
O
N
Boc
4b
180 170 160 150 140 130 120 110 100 90 80 70 60 50 40 30 20 10 0
f1 (ppm)

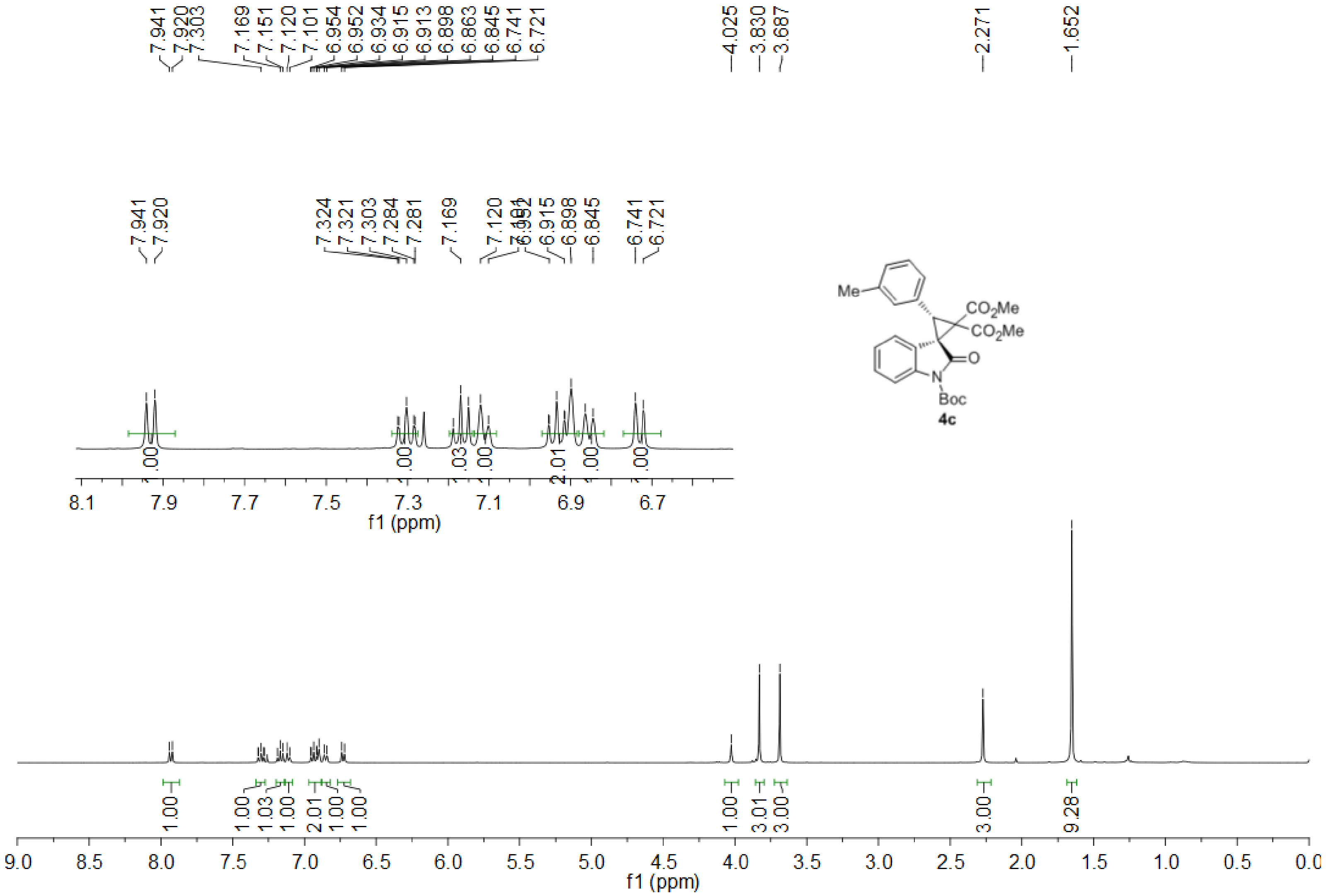
7.941
7.920
7.303
7.169
7.151
7.120
7.101
6.954
6.952
6.934
6.915
6.913
6.898
6.863
6.845
6.741
6.721
4.025
3.830
3.687
2.271
1.652
7.324
7.321
7.284
7.281
Me
CO_2Me
CO_2Me
O
N
Boc
4c
f1 (ppm)
1.00
1.03
2.01
3.01
3.00
9.28
9.0 8.5 8.0 7.5 7.0 6.5 6.0 5.5 5.0 4.5 4.0 3.5 3.0 2.5 2.0 1.5 1.0 0.5 0.0
8.1 7.9 7.7 7.5 7.3 7.1 6.9 6.7

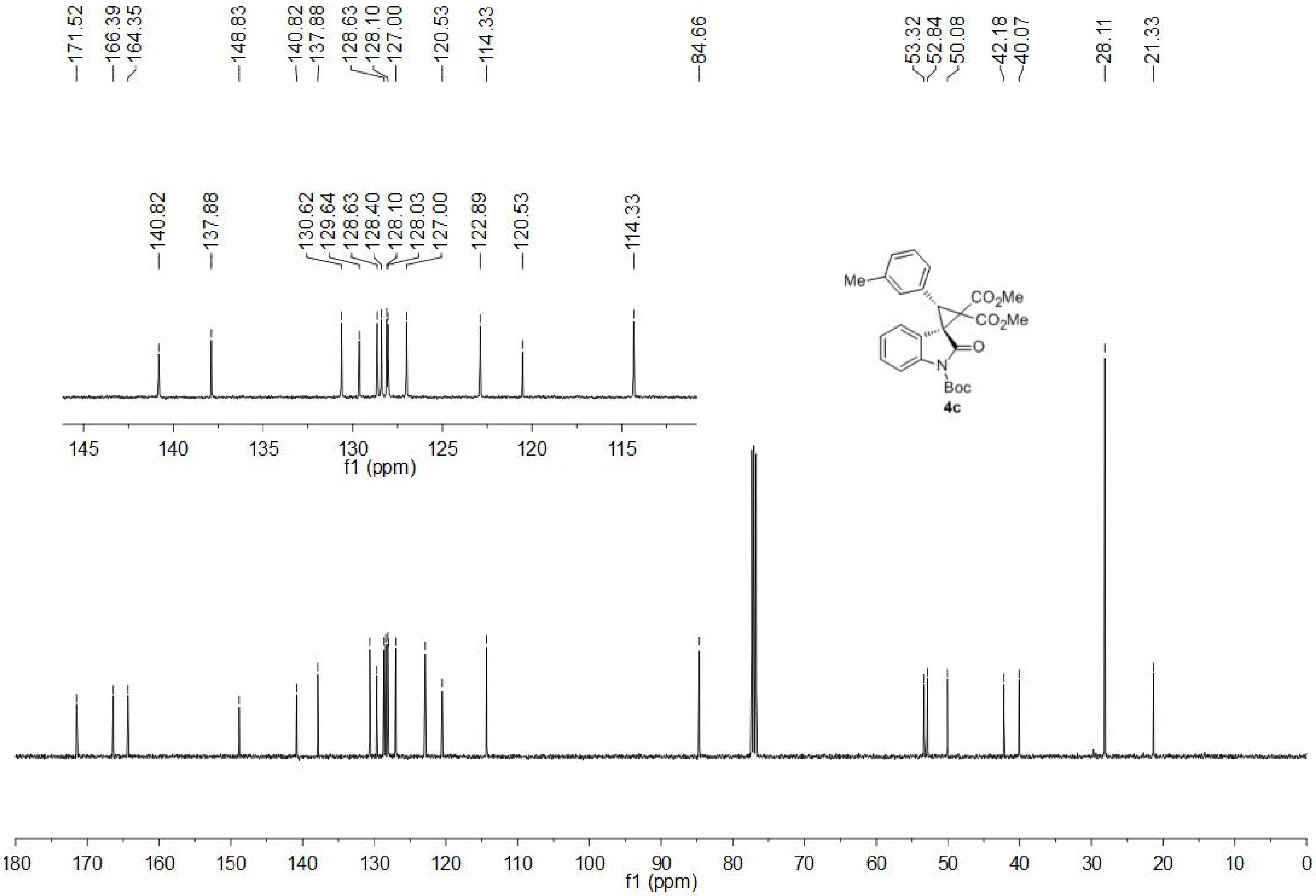

171.52
166.39
164.35
148.83
140.82
137.88
128.63
128.10
127.00
120.53
114.33
84.66
53.32
52.84
50.08
42.18
40.07
28.11
21.33
140.82
137.88
130.62
129.64
128.63
128.40
128.10
128.03
127.00
122.89
120.53
114.33
145
140
135
130
125
120
115
f1 (ppm)
Me
CO_2Me
CO_2Me
O
N
Boc
4c
180
170
160
150
140
130
120
110
100
90
80
70
60
50
40
30
20
10
0
f1 (ppm)

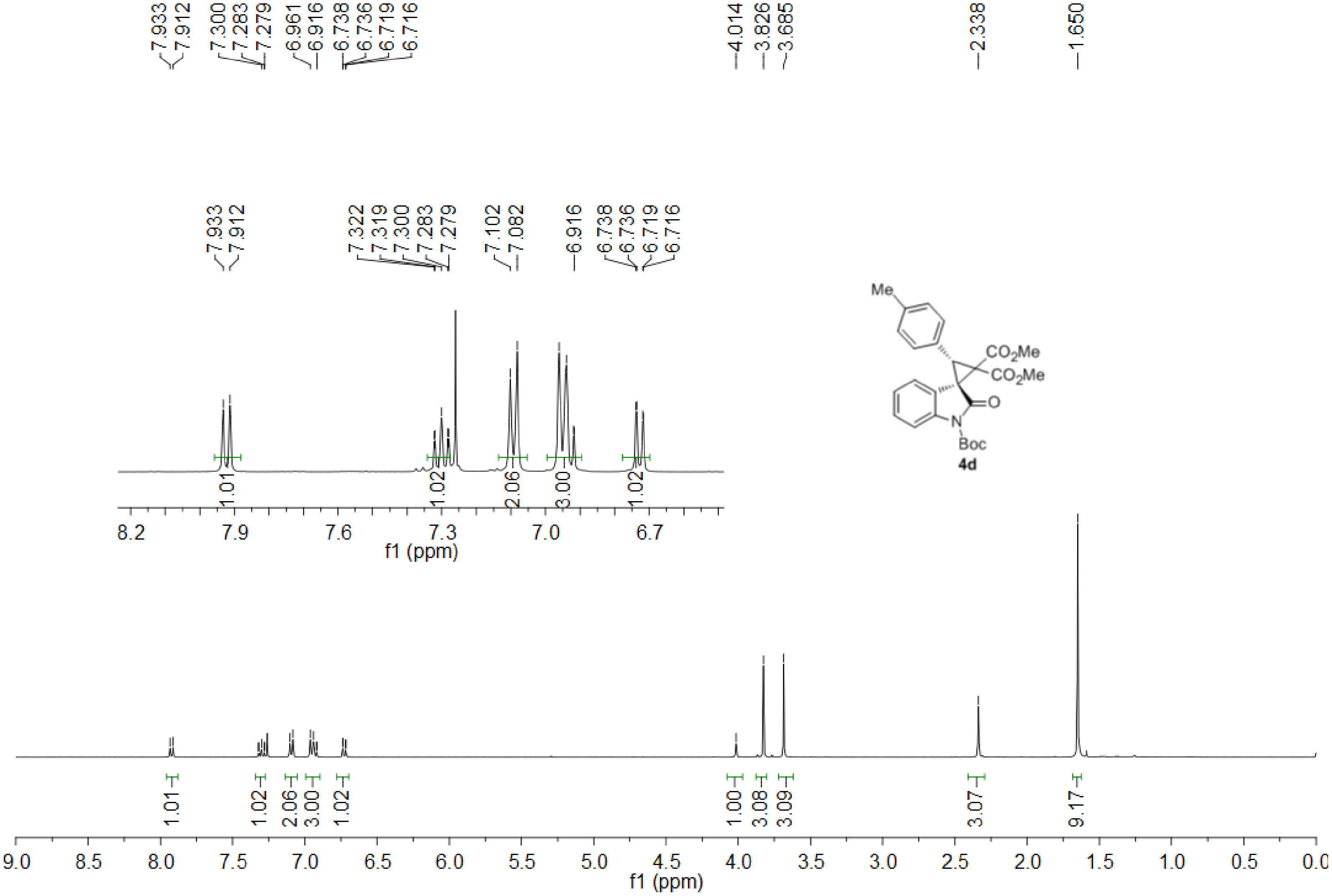
7.933
7.912
7.300
7.283
7.279
6.961
6.916
6.738
6.736
6.719
6.716
4.014
3.826
3.685
2.338
1.650
7.933
7.912
7.322
7.319
7.300
7.283
7.279
7.102
7.082
6.916
6.738
6.736
6.719
6.716
Me
CO2Me
CO2Me
O
N
Boc
4d
1.01
1.02
2.06
3.00
1.02
8.2
7.9
7.6
7.3
7.0
6.7
f1 (ppm)
1.01
1.02
2.06
3.00
1.02
1.00
3.08
3.09
3.07
9.17
9.0
8.5
8.0
7.5
7.0
6.5
6.0
5.5
5.0
4.5
4.0
3.5
3.0
2.5
2.0
1.5
1.0
0.5
0.0
f1 (ppm)

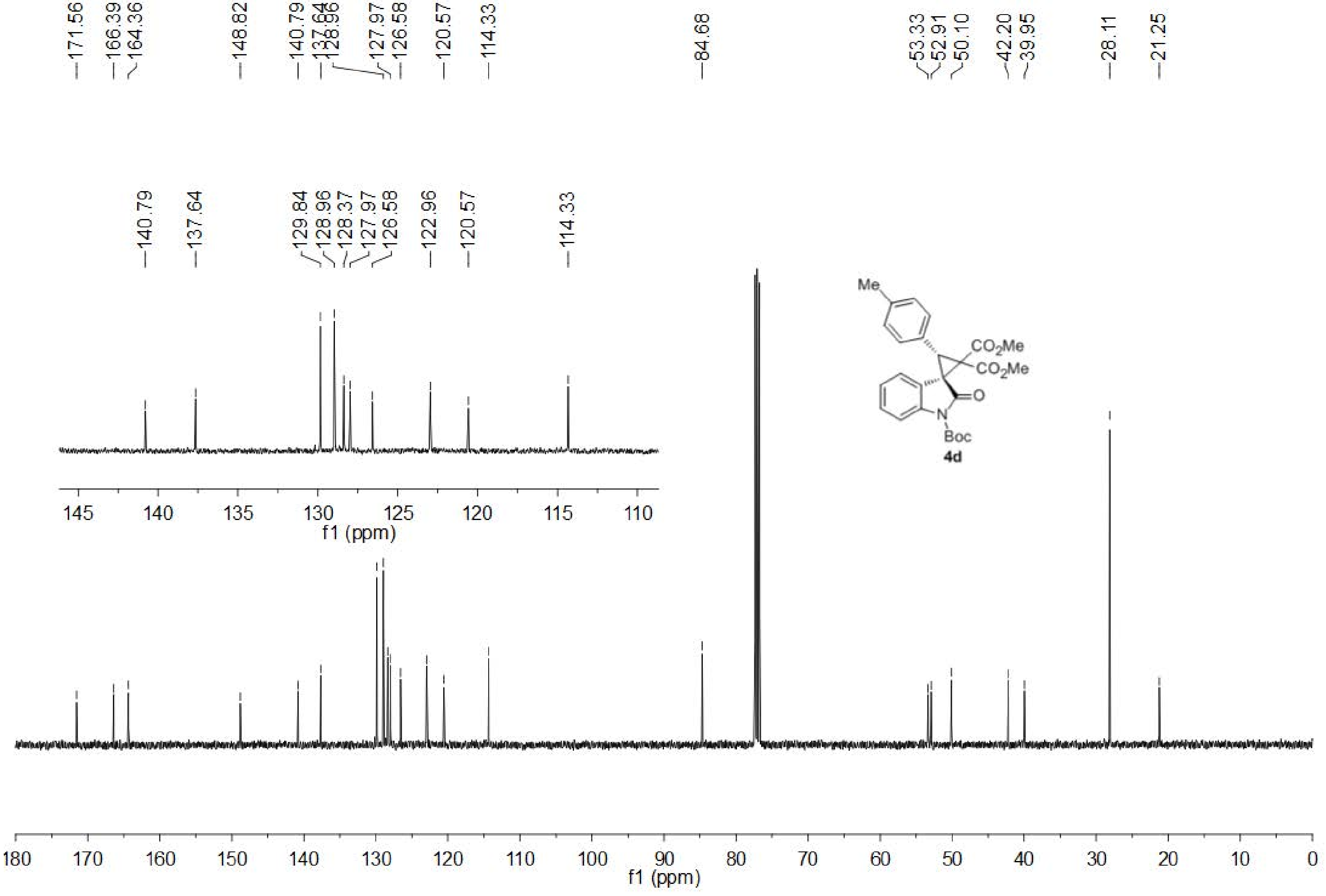
171.56
166.39
164.36
148.82
140.79
137.64
128.96
127.97
126.58
120.57
114.33
84.68
53.33
52.91
50.10
42.20
39.95
28.11
21.25
129.84
128.37
122.96
Me
CO2Me
CO2Me
O
N
Boc
4d
f1 (ppm)

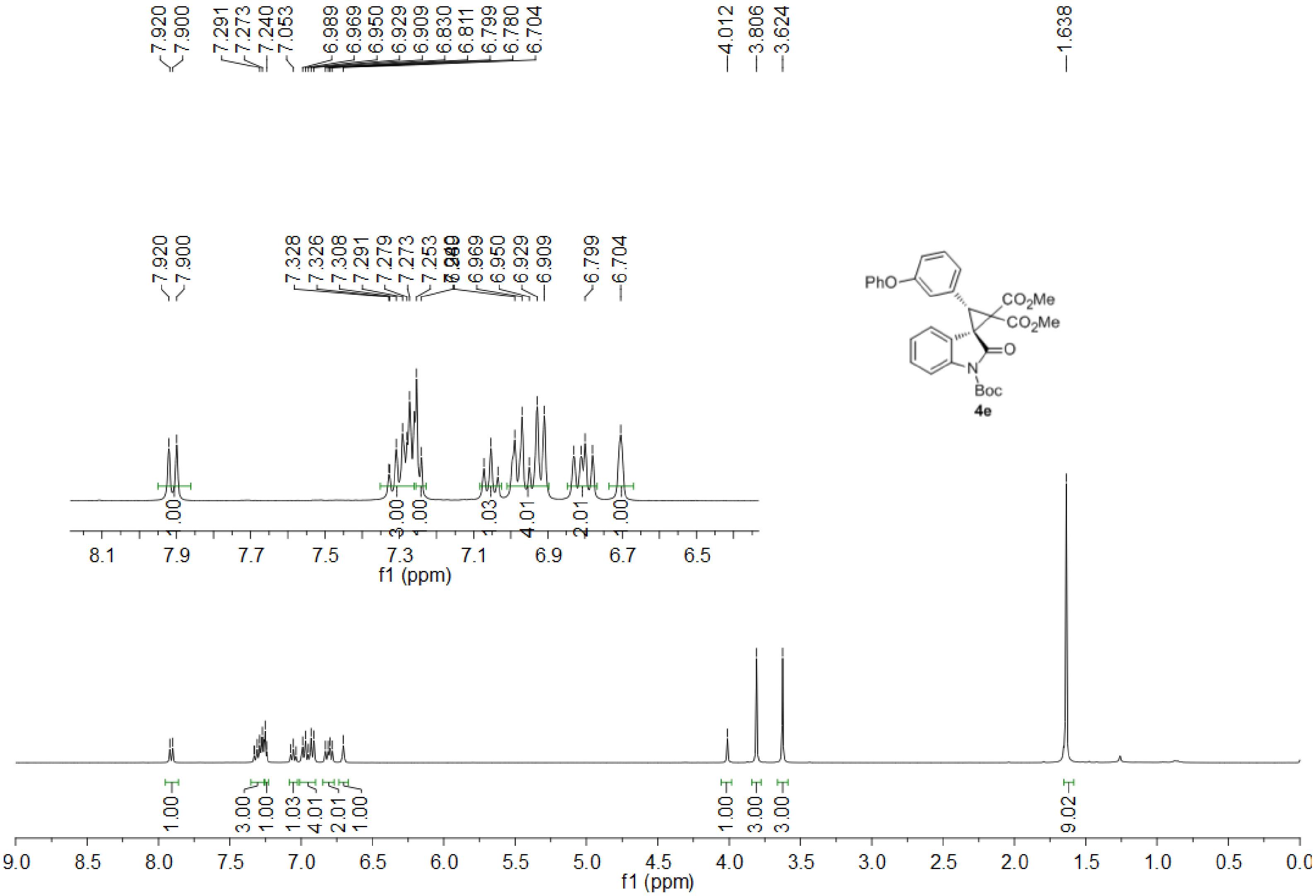

7.920
7.900
7.291
7.273
7.240
7.053
6.989
6.969
6.950
6.929
6.909
6.830
6.811
6.799
6.780
6.704
4.012
3.806
3.624
1.638
PhO
CO_2Me
CO_2Me
O
N
Boc
4e
f1 (ppm)

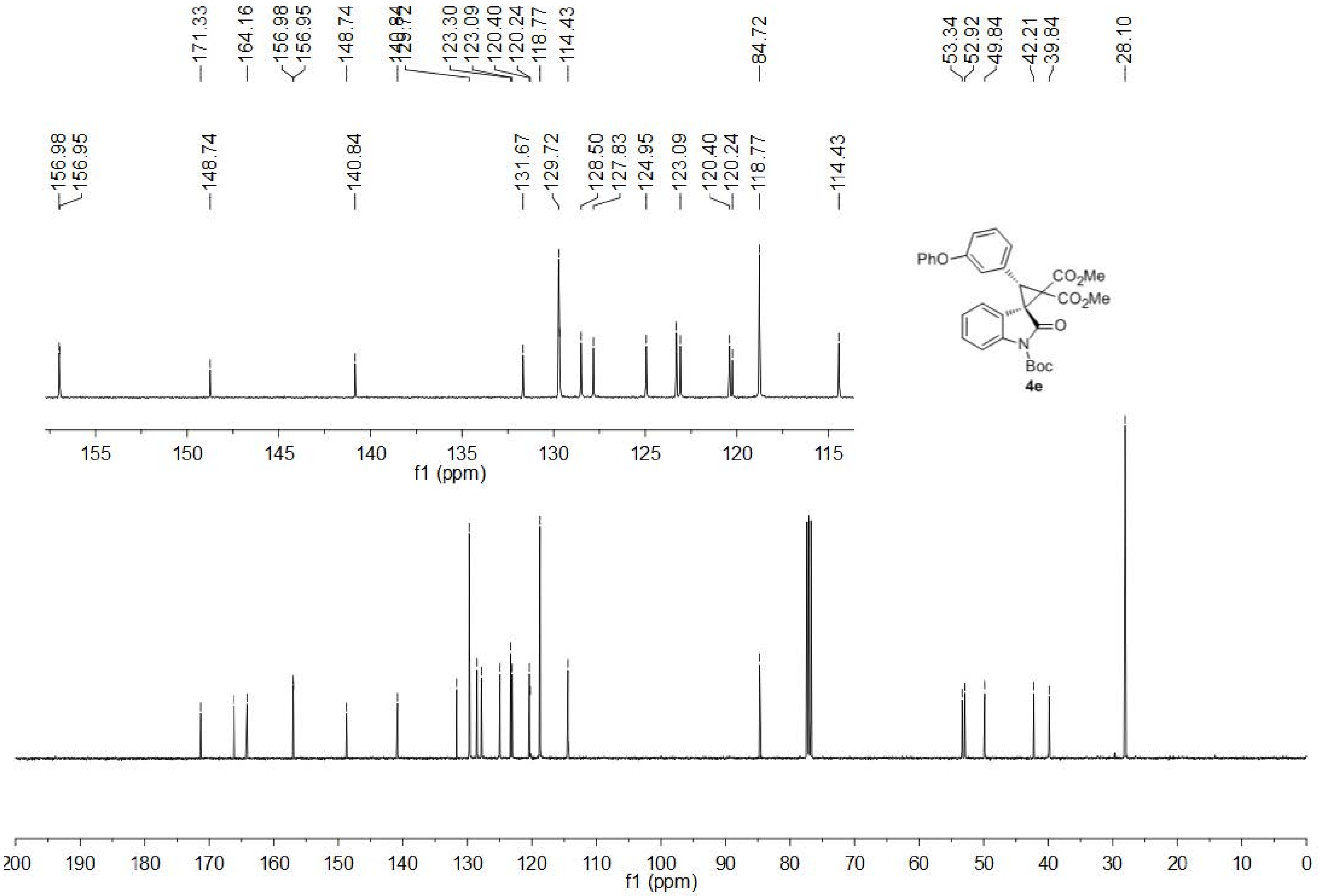

171.33
164.16
156.98
156.95
148.74
140.84
129.72
123.30
123.09
120.40
120.24
118.77
114.43
84.72
53.34
52.92
49.84
42.21
39.84
28.10
156.98
156.95
148.74
140.84
131.67
129.72
128.50
127.83
124.95
123.09
120.40
120.24
118.77
114.43
PhO
CO2Me
CO2Me
O
N
Boc
4e
155
150
145
140
135
130
125
120
115
f1 (ppm)
200
190
180
170
160
150
140
130
120
110
100
90
80
70
60
50
40
30
20
10
0
f1 (ppm)

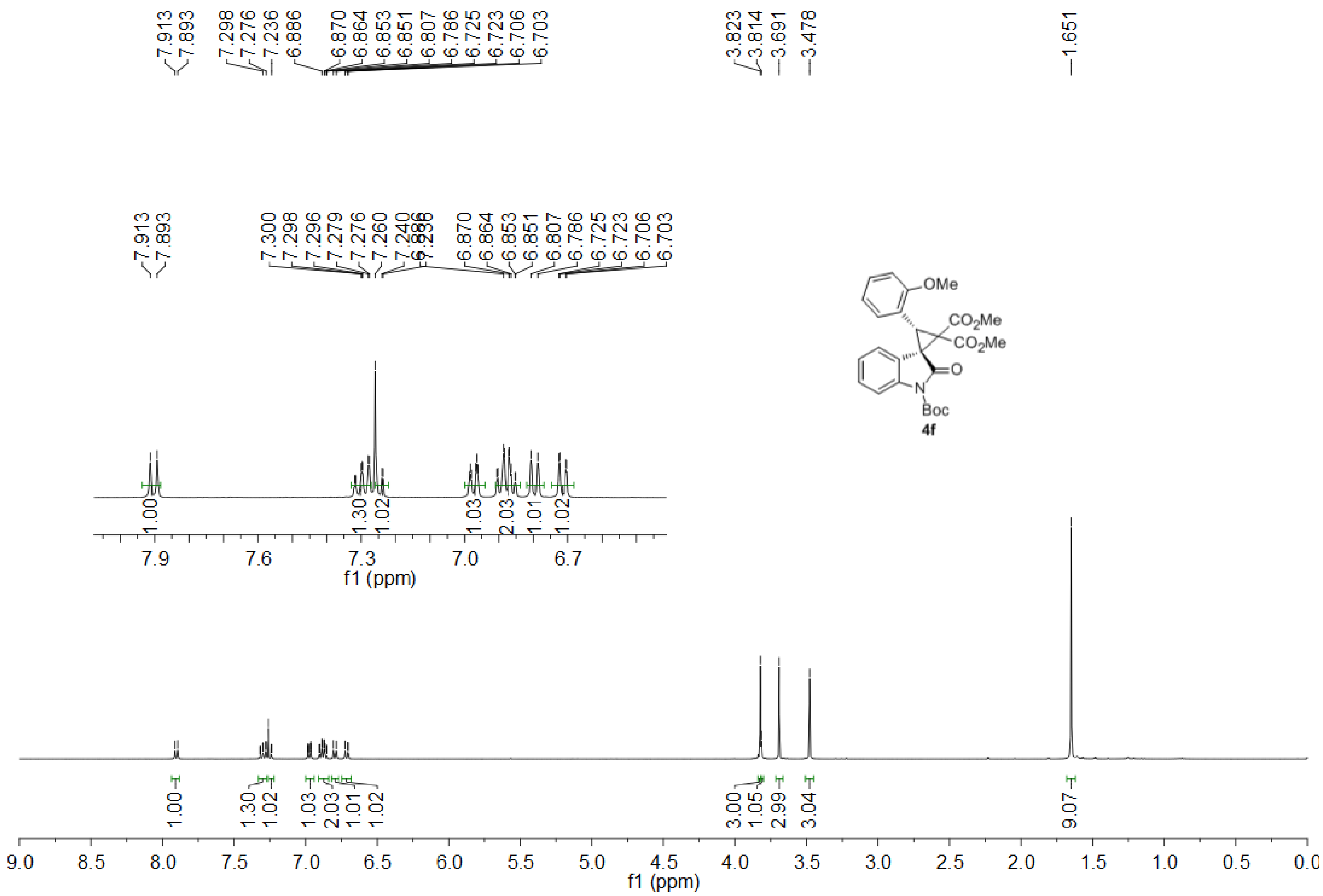
7.913
7.893
7.298
7.276
7.236
6.886
6.870
6.864
6.853
6.851
6.807
6.786
6.725
6.723
6.706
6.703
3.823
3.814
3.691
3.478
1.651
OMe
CO2Me
CO2Me
N
O
Boc
4f
1.00
1.30
1.02
1.03
2.03
1.01
1.02
3.00
1.05
2.99
3.04
9.07
f1 (ppm)

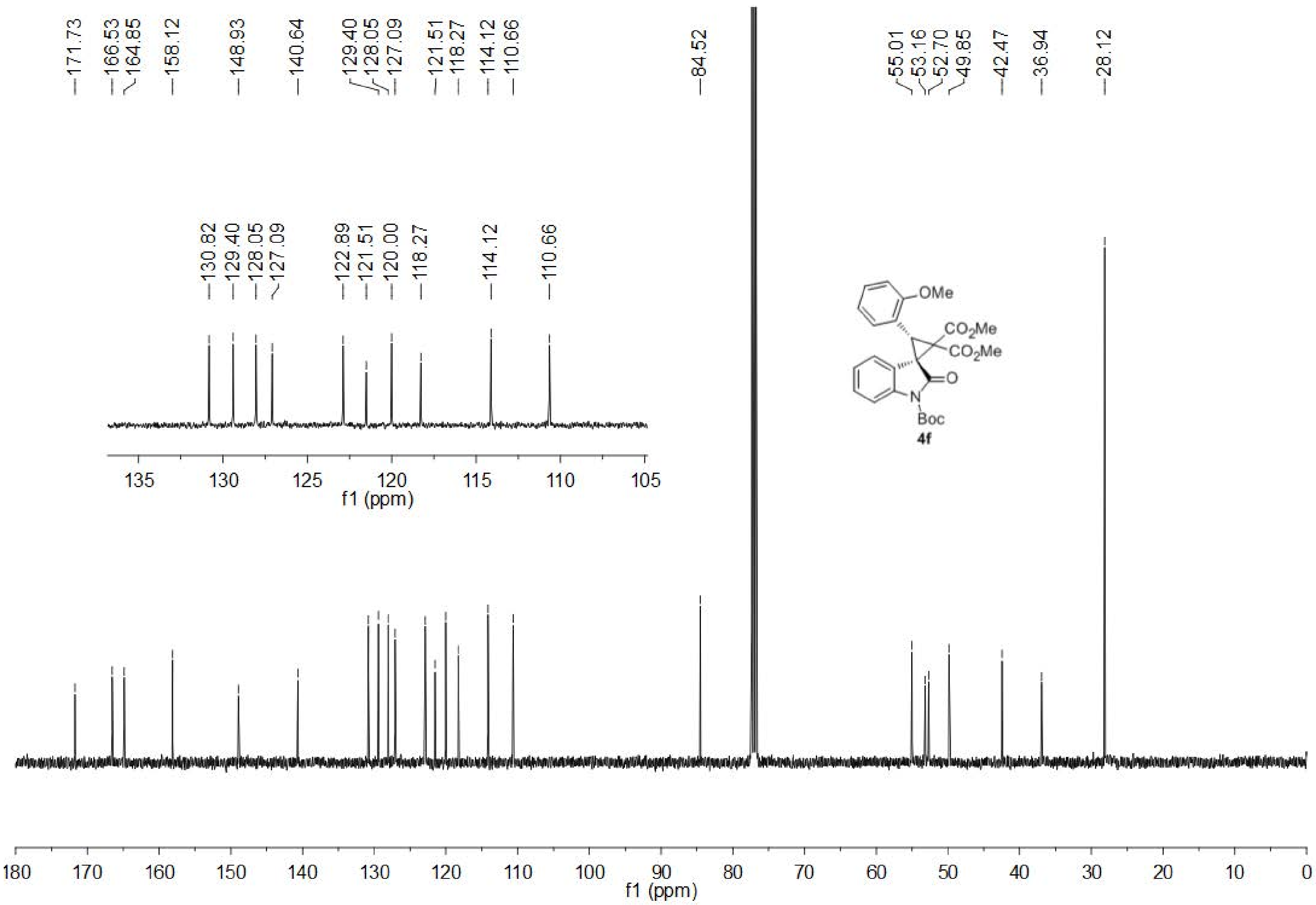
171.73
166.53
164.85
158.12
148.93
140.64
129.40
128.05
127.09
121.51
118.27
114.12
110.66
84.52
55.01
53.16
52.70
49.85
42.47
36.94
28.12
130.82
129.40
128.05
127.09
122.89
121.51
120.00
118.27
114.12
110.66
OMe
CO_2Me
CO_2Me
N
O
Boc
4f
f1 (ppm)
f1 (ppm)

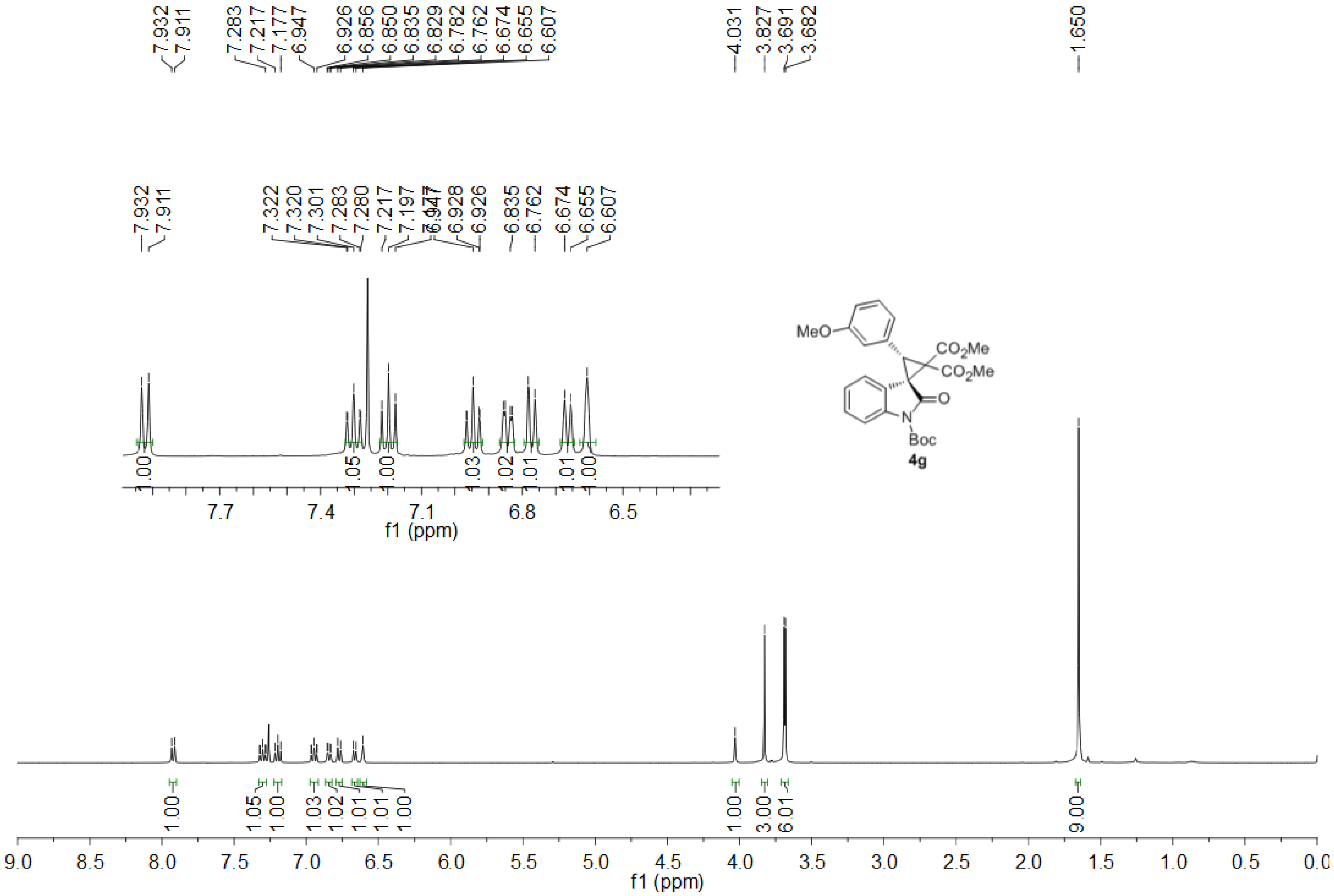

MeO
CO2Me
CO2Me
O
N
Boc
4g
f1 (ppm)

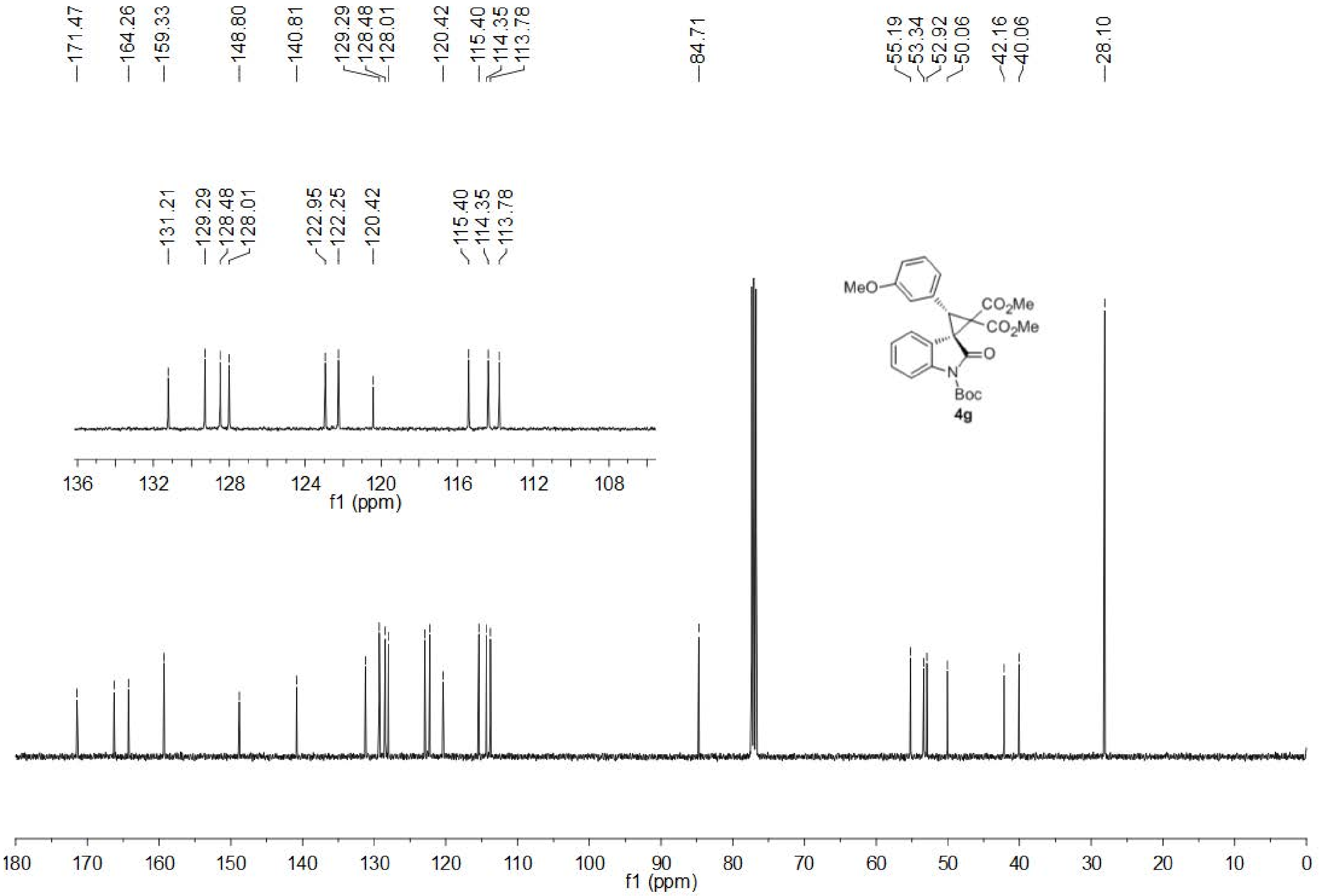

171.47
164.26
159.33
148.80
140.81
129.29
128.48
128.01
120.42
115.40
114.35
113.78
84.71
55.19
53.34
52.92
50.06
42.16
40.06
28.10
131.21
129.29
128.48
128.01
122.95
122.25
120.42
115.40
114.35
113.78
MeO
CO_2Me
CO_2Me
O
N
Boc
4g
136
132
128
124
120
116
112
108
f1 (ppm)
180
170
160
150
140
130
120
110
100
90
80
70
60
50
40
30
20
10
0
f1 (ppm)

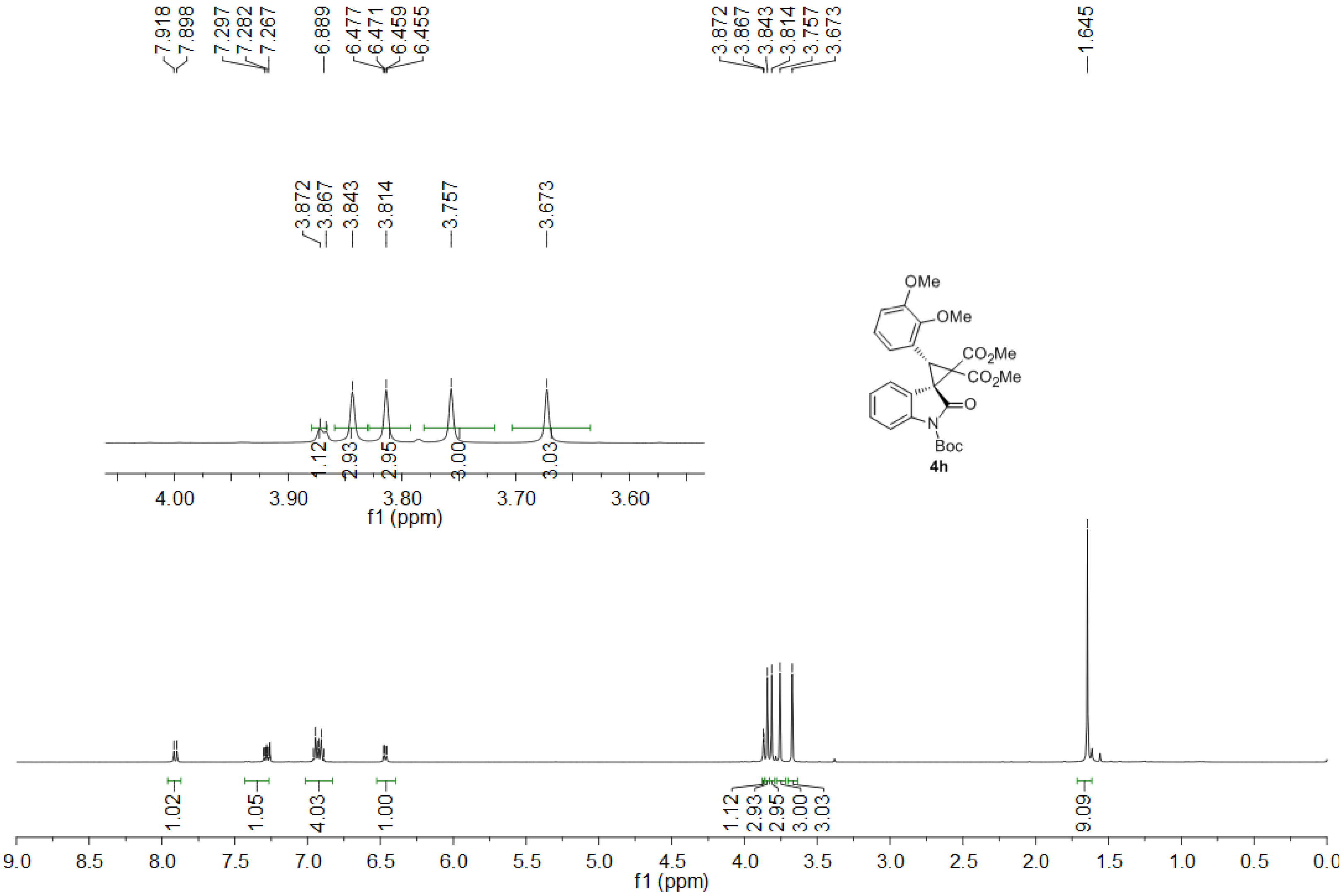

7.918
7.898
7.297
7.282
7.267
6.889
6.477
6.471
6.459
6.455
3.872
3.867
3.843
3.814
3.757
3.673
1.645
OMe
OMe
CO2Me
CO2Me
O
N
Boc
4h
1.12
2.93
2.95
3.00
3.03
4.00
3.90
3.80
3.70
3.60
f1 (ppm)
1.02
1.05
4.03
1.00
9.09
9.0
8.5
8.0
7.5
7.0
6.5
6.0
5.5
5.0
4.5
4.0
3.5
3.0
2.5
2.0
1.5
1.0
0.5
0.0
f1 (ppm)

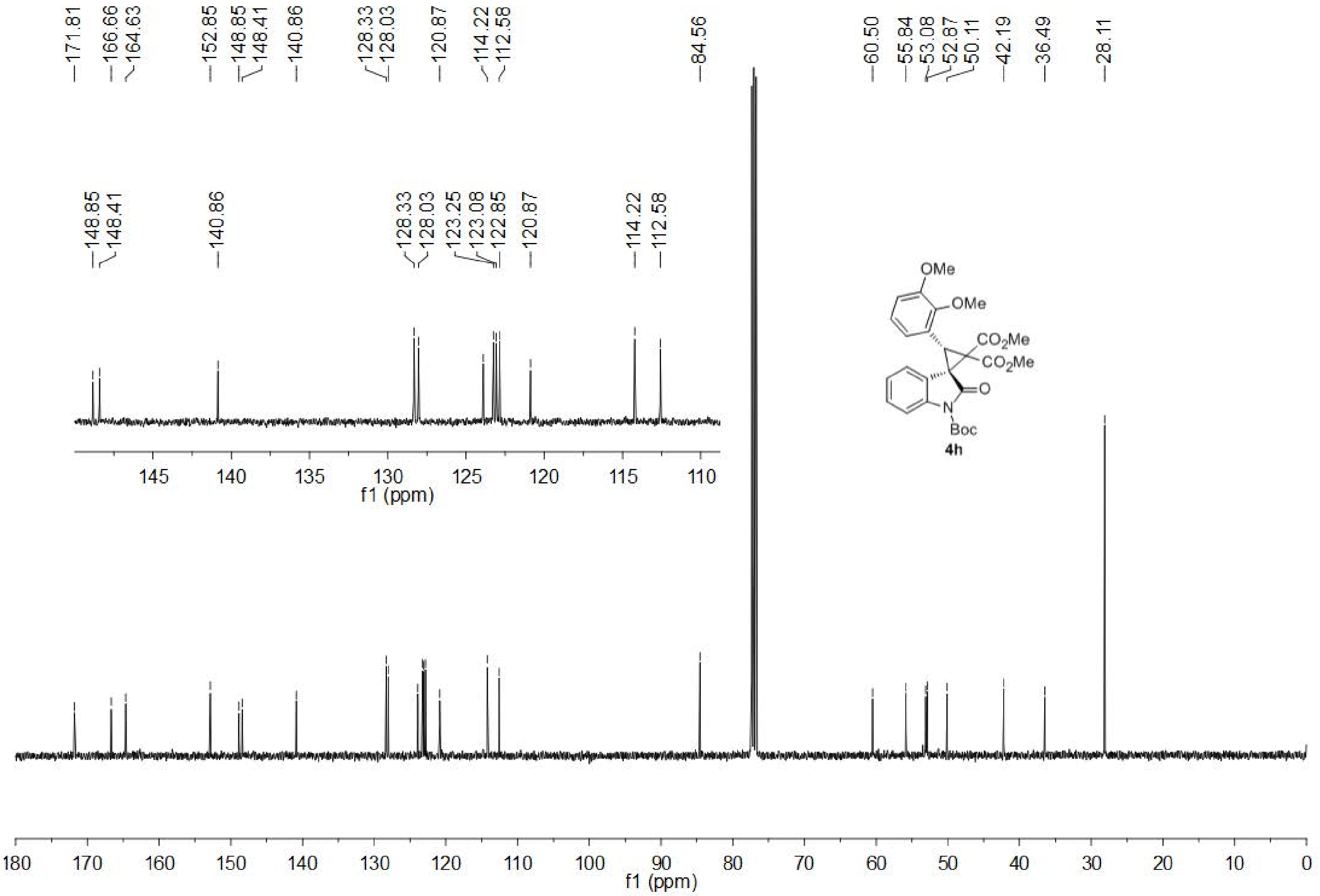
171.81
166.66
164.63
152.85
148.85
148.41
140.86
128.33
128.03
120.87
114.22
112.58
84.56
60.50
55.84
53.08
52.87
50.11
42.19
36.49
28.11
148.85
148.41
140.86
128.33
128.03
123.25
123.08
122.85
120.87
114.22
112.58
145 140 135 130 125 120 115 110
f1 (ppm)
OMe
OMe
CO2Me
CO2Me
O
N
Boc
4h
180 170 160 150 140 130 120 110 100 90 80 70 60 50 40 30 20 10 0
f1 (ppm)

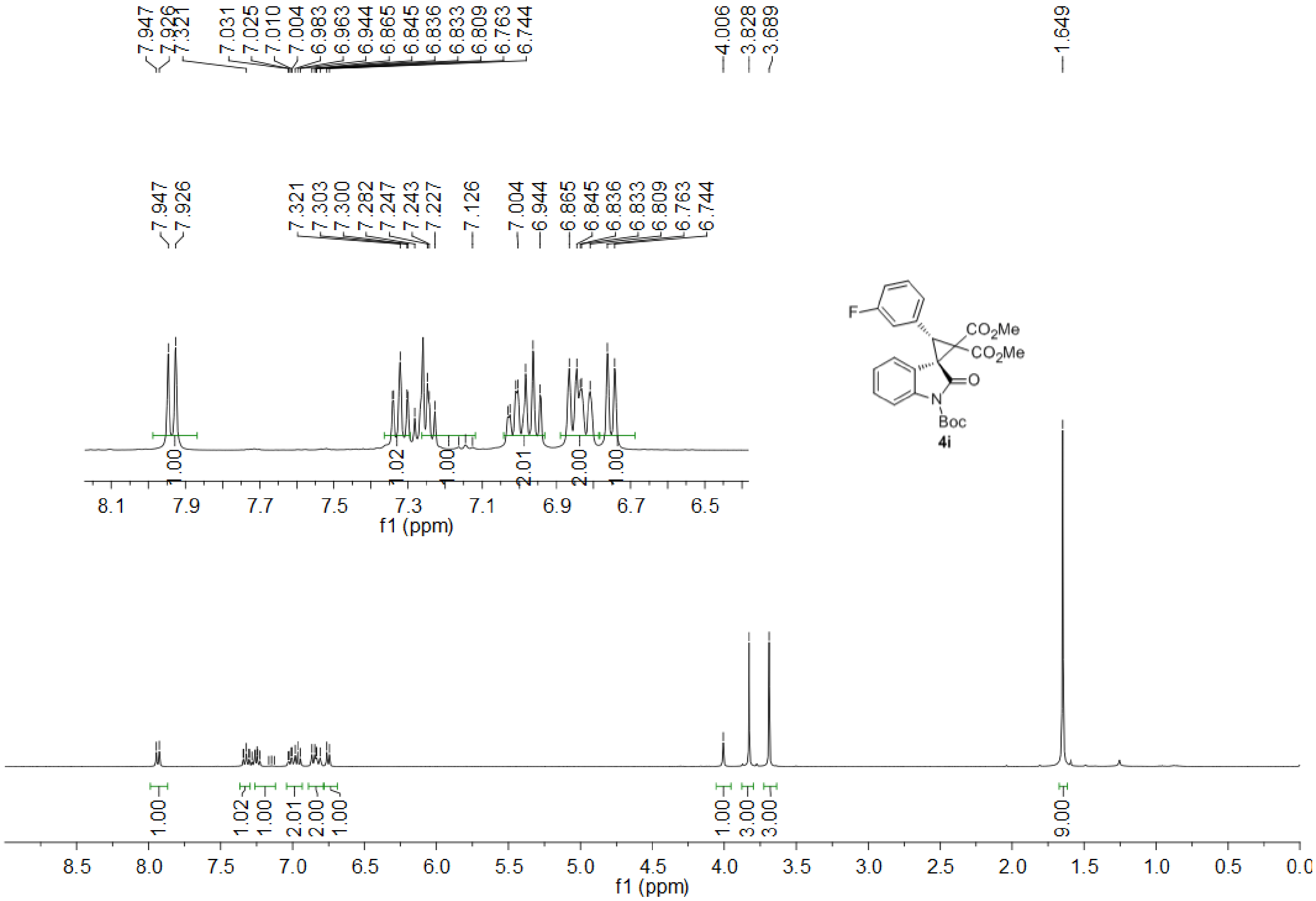
7.947
7.926
7.321
7.031
7.025
7.010
7.004
6.983
6.963
6.944
6.865
6.845
6.836
6.833
6.809
6.763
6.744
4.006
3.828
3.689
1.649
7.947
7.926
7.321
7.303
7.300
7.282
7.247
7.243
7.227
7.126
7.004
6.944
6.865
6.845
6.836
6.833
6.809
6.763
6.744
F
CO2Me
CO2Me
O
N
Boc
4i
1.00
1.02
1.00
2.01
2.00
1.00
8.1
7.9
7.7
7.5
7.3
7.1
6.9
6.7
6.5
f1 (ppm)
1.00
1.02
1.00
2.01
2.00
1.00
1.00
3.00
3.00
9.00
8.5
8.0
7.5
7.0
6.5
6.0
5.5
5.0
4.5
4.0
3.5
3.0
2.5
2.0
1.5
1.0
0.5
0.0
f1 (ppm)

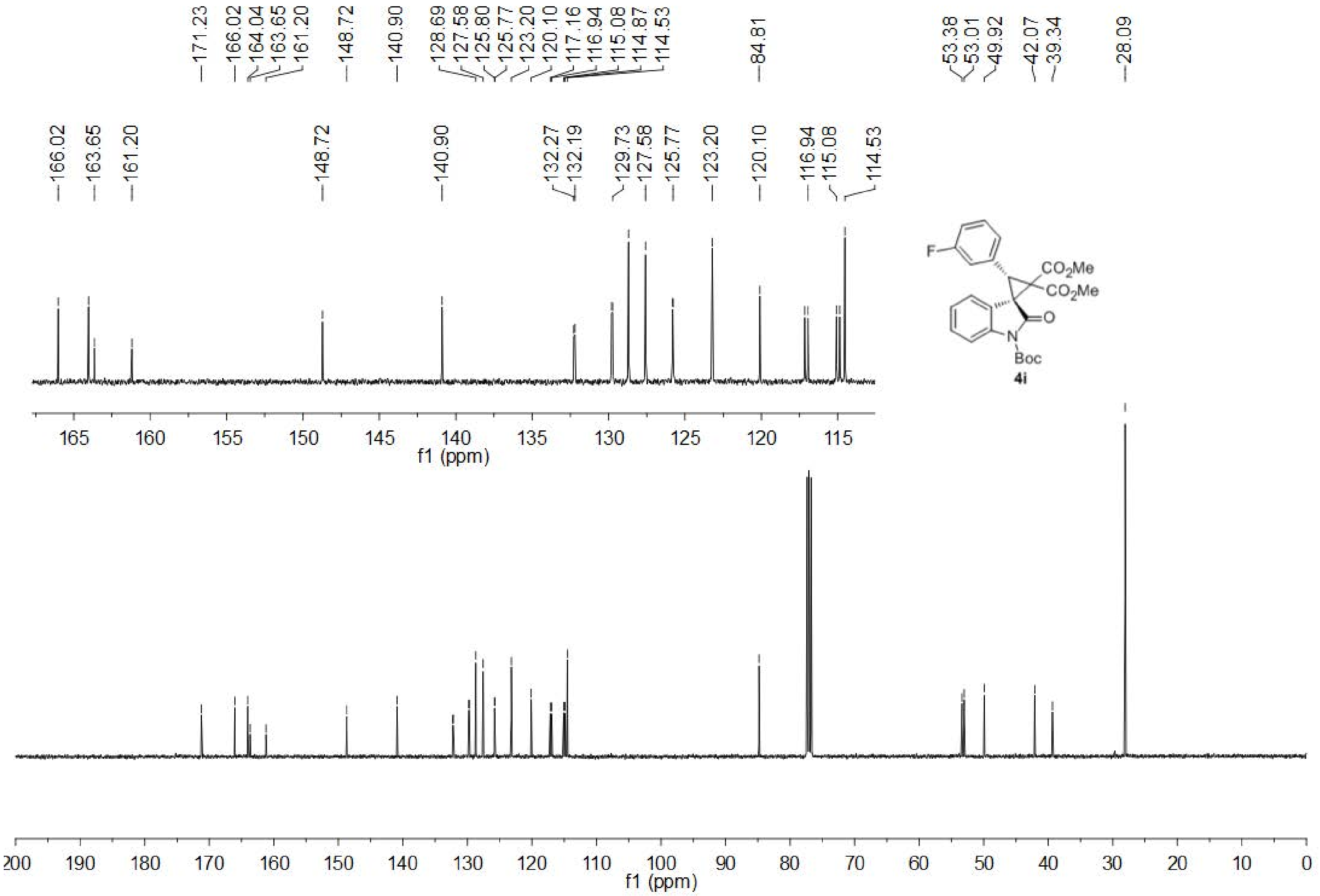

171.23
166.02
164.04
163.65
161.20
148.72
140.90
128.69
127.58
125.80
125.77
123.20
120.10
117.16
116.94
115.08
114.87
114.53
84.81
53.38
53.01
49.92
42.07
39.34
28.09
166.02
163.65
161.20
148.72
140.90
132.27
132.19
129.73
127.58
125.77
123.20
120.10
116.94
115.08
114.53
F
CO2Me
CO2Me
O
N
Boc
4i
165 160 155 150 145 140 135 130 125 120 115
f1 (ppm)
200 190 180 170 160 150 140 130 120 110 100 90 80 70 60 50 40 30 20 10 0
f1 (ppm)

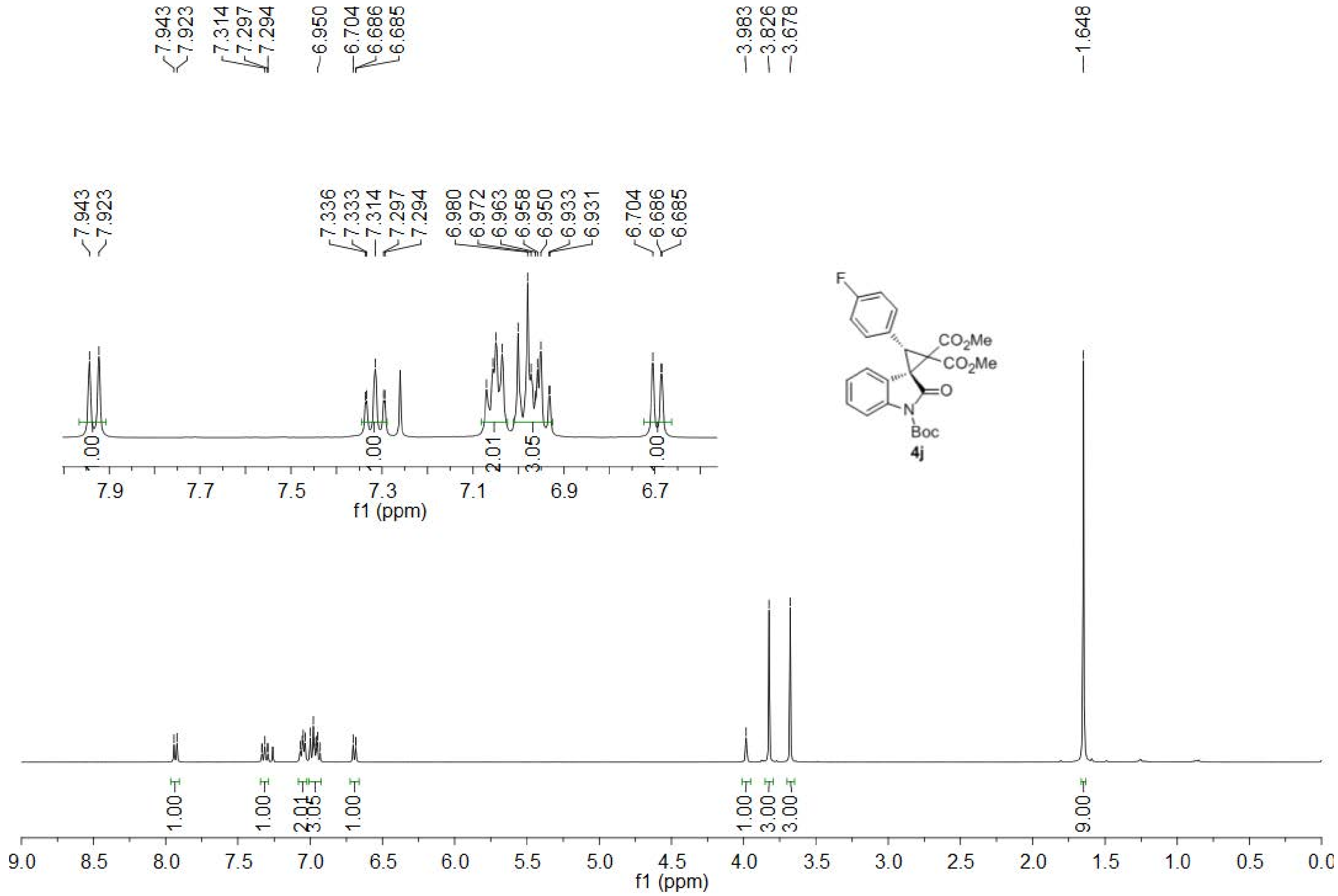

7.943
7.923
7.314
7.297
7.294
6.950
6.704
6.686
6.685
3.983
3.826
3.678
1.648
7.336
7.333
6.980
6.972
6.963
6.958
6.933
6.931
F
CO2Me
CO2Me
O
N
Boc
4j
1.00
2.01
3.05
3.00
9.00
f1 (ppm)

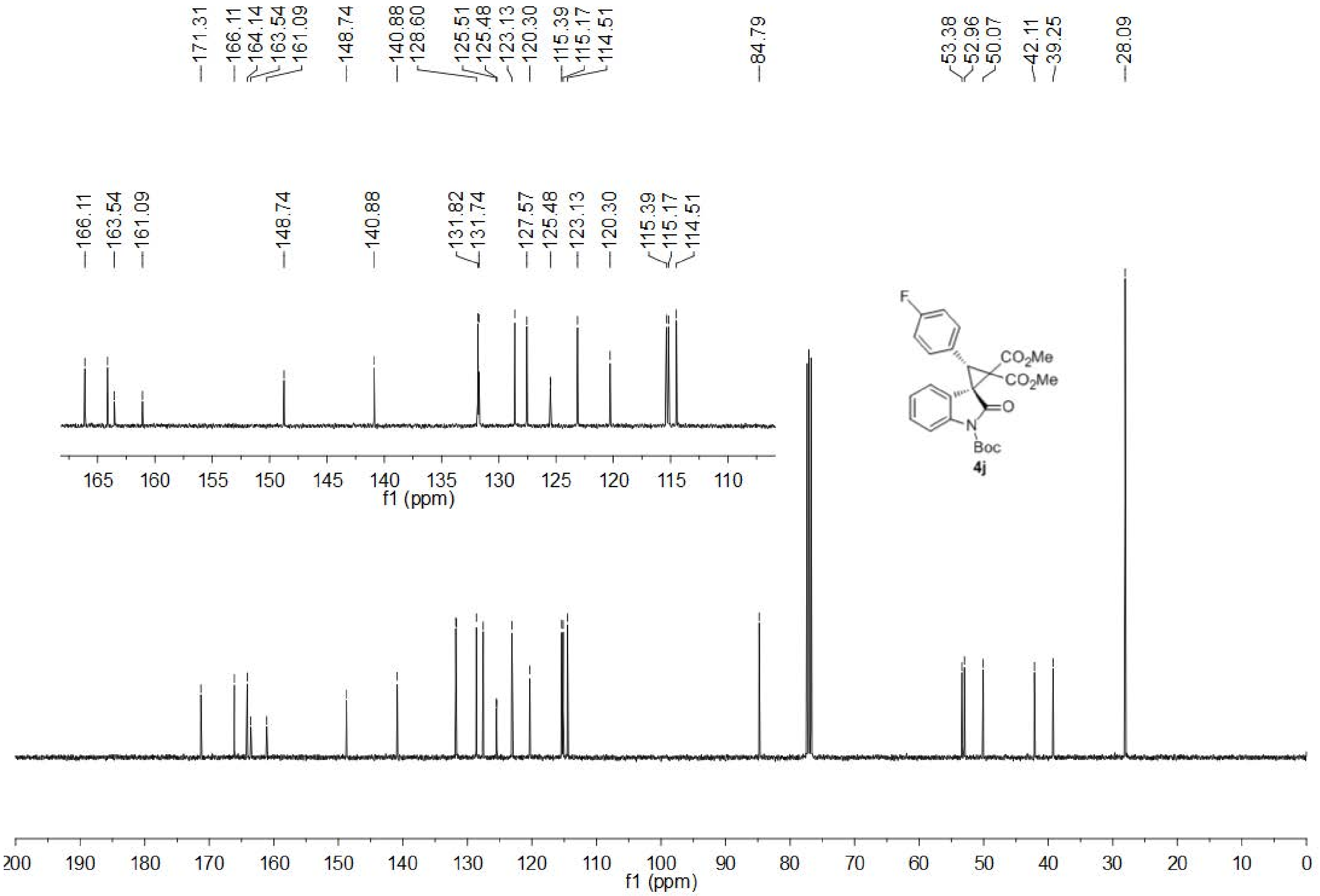

171.31
166.11
164.14
163.54
161.09
148.74
140.88
128.60
125.51
125.48
123.13
120.30
115.39
115.17
114.51
84.79
53.38
52.96
50.07
42.11
39.25
28.09
166.11
163.54
161.09
148.74
140.88
131.82
131.74
127.57
125.48
123.13
120.30
115.39
115.17
114.51
F
CO_2Me
CO_2Me
O
N
Boc
4j
f1 (ppm)
f1 (ppm)

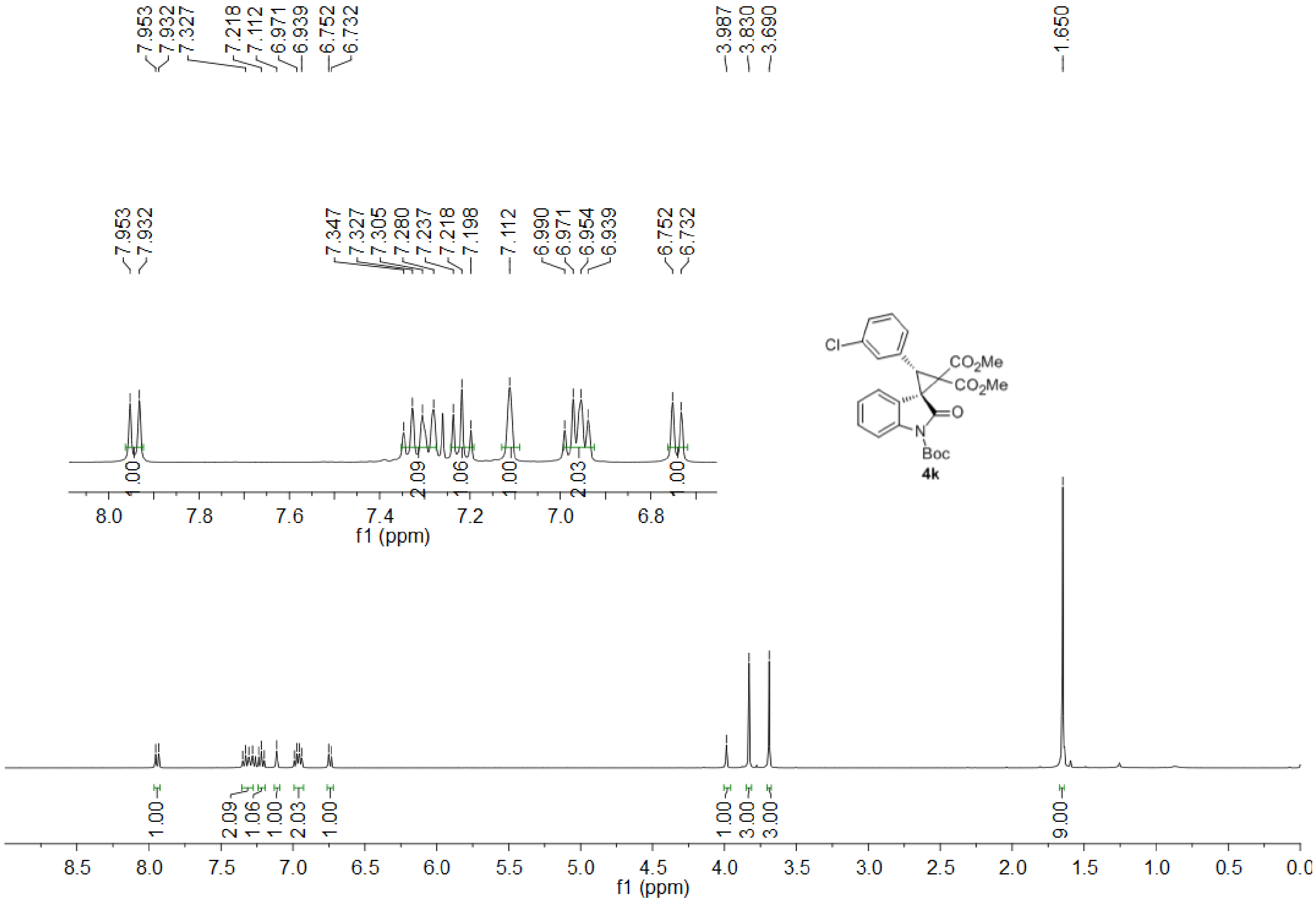

7.953
7.932
7.327
7.218
7.112
6.971
6.939
6.752
6.732
3.987
3.830
3.690
1.650
7.953
7.932
7.347
7.327
7.305
7.280
7.237
7.218
7.198
7.112
6.990
6.971
6.954
6.939
6.752
6.732
Cl
CO2Me
CO2Me
O
N
Boc
4k
1.00
2.09
1.06
1.00
2.03
1.00
8.0
7.8
7.6
7.4
7.2
7.0
6.8
f1 (ppm)
1.00
2.09
1.06
1.00
2.03
1.00
1.00
3.00
3.00
9.00
8.5
8.0
7.5
7.0
6.5
6.0
5.5
5.0
4.5
4.0
3.5
3.0
2.5
2.0
1.5
1.0
0.5
0.0
f1 (ppm)

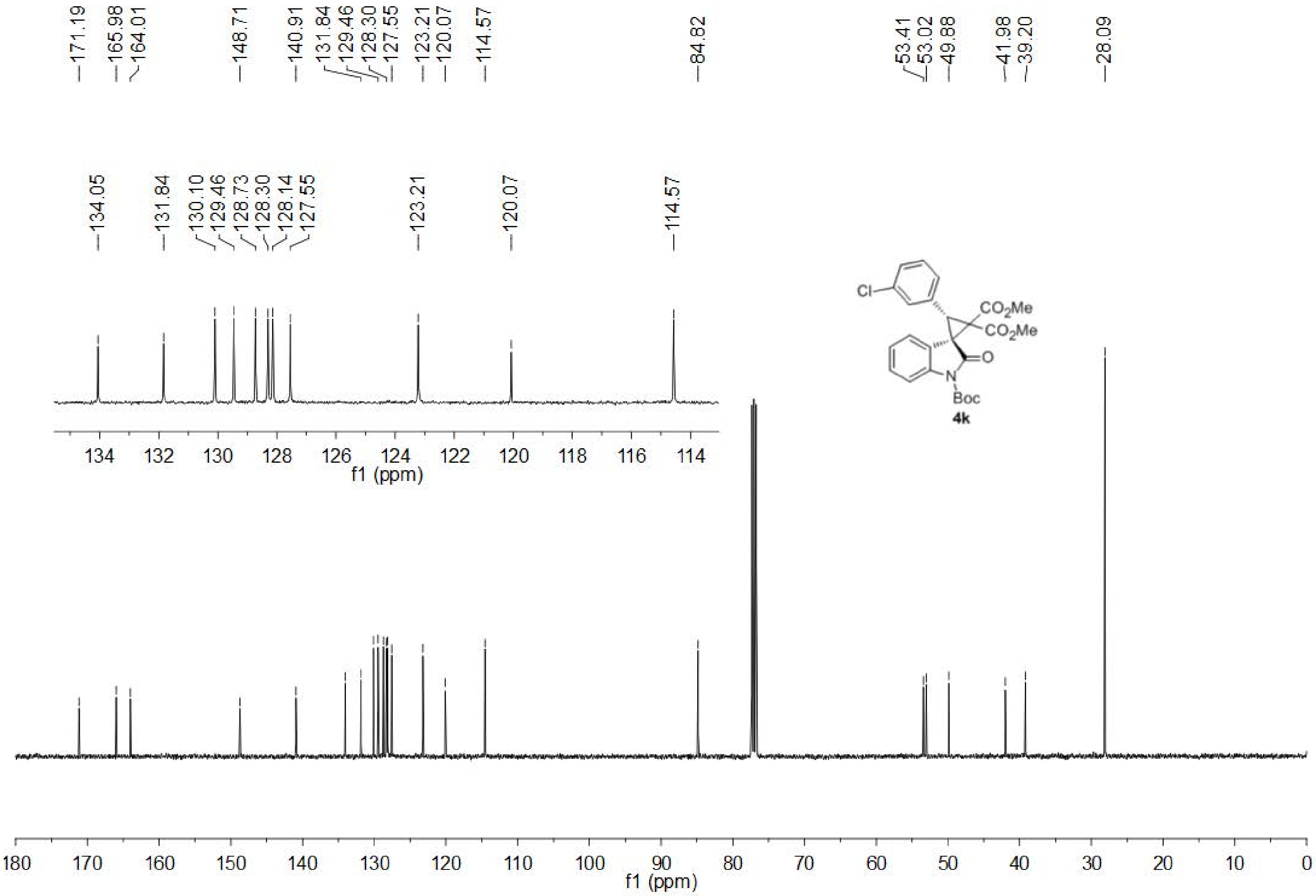
171.19
165.98
164.01
148.71
140.91
131.84
129.46
128.30
127.55
123.21
120.07
114.57
84.82
53.41
53.02
49.88
41.98
39.20
28.09
134.05
131.84
130.10
129.46
128.73
128.30
128.14
127.55
123.21
120.07
114.57
134 132 130 128 126 124 122 120 118 116 114
f1 (ppm)
Cl
CO2Me
CO2Me
O
N
Boc
4k
180 170 160 150 140 130 120 110 100 90 80 70 60 50 40 30 20 10 0
f1 (ppm)

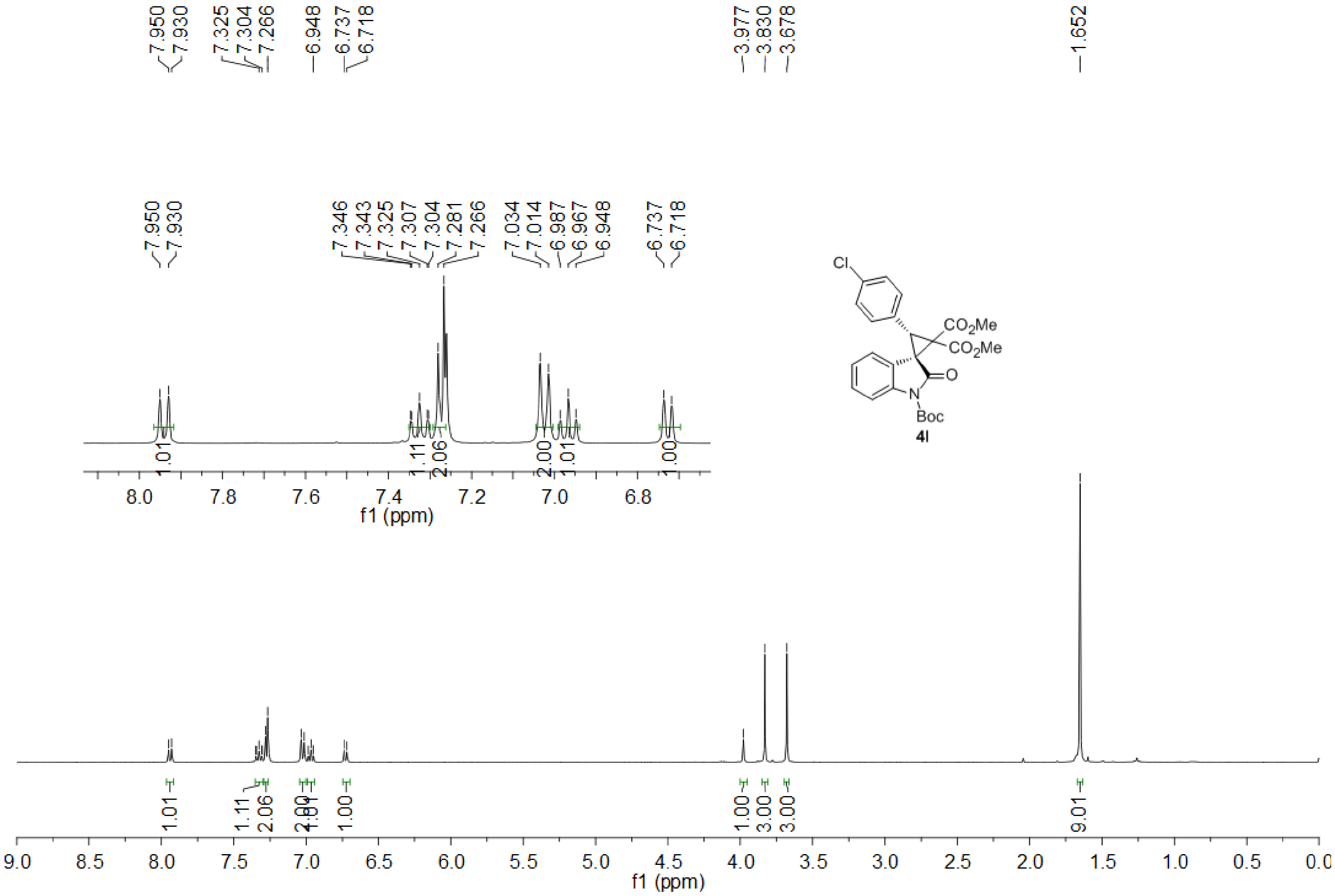
7.950
7.930
7.325
7.304
7.266
6.948
6.737
6.718
3.977
3.830
3.678
1.652
7.346
7.343
7.307
7.281
7.034
7.014
6.987
6.967
Cl
CO2Me
CO2Me
O
N
Boc
1.01
1.11
2.06
2.00
1.01
1.00
3.00
3.00
9.01
f1 (ppm)
4l

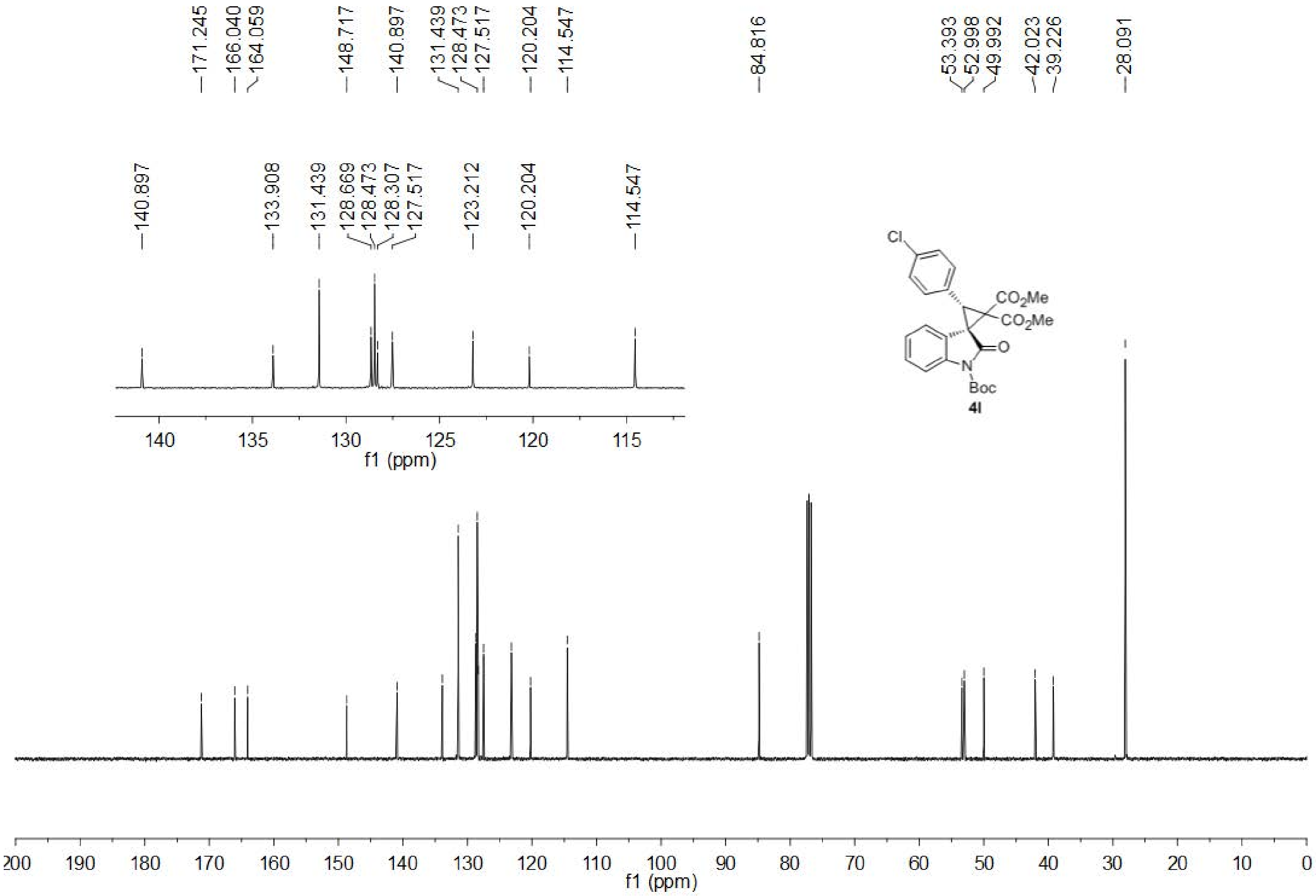
171.245
166.040
164.059
148.717
140.897
131.439
128.473
127.517
120.204
114.547
84.816
53.393
52.998
49.992
42.023
39.226
28.091
140.897
133.908
131.439
128.669
128.473
128.307
127.517
123.212
120.204
114.547
f1 (ppm)
Cl
CO2Me
CO2Me
O
N
Boc
4l
f1 (ppm)

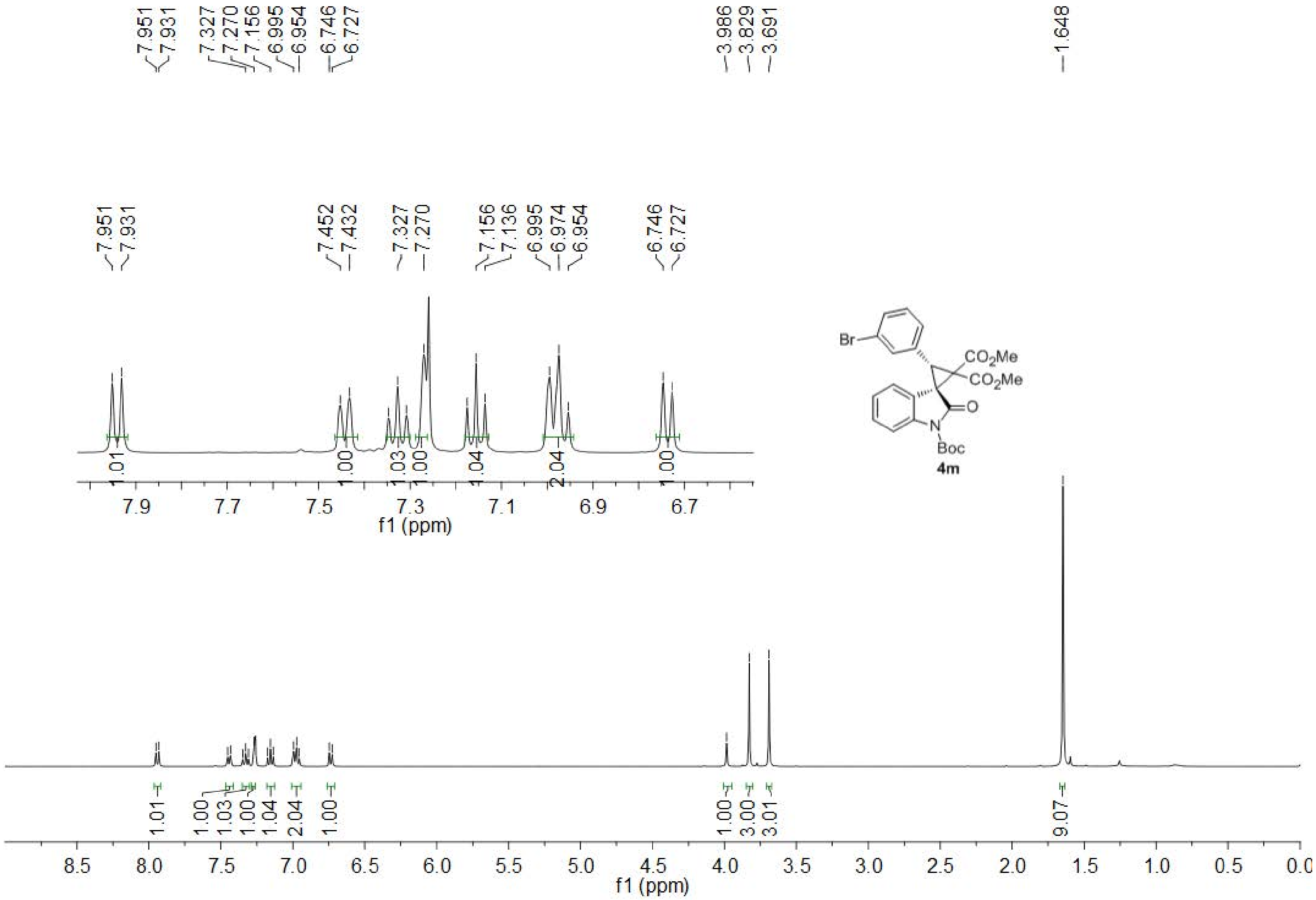
7.951
7.931
7.327
7.270
7.156
6.995
6.954
6.746
6.727
3.986
3.829
3.691
1.648
7.951
7.931
7.452
7.432
7.327
7.270
7.156
7.136
6.995
6.974
6.954
6.746
6.727
1.01
1.00
1.03
1.00
1.04
2.04
1.00
7.9
7.7
7.5
7.3
7.1
6.9
6.7
f1 (ppm)
Br
CO_2Me
CO_2Me
O
N
Boc
4m
1.01
1.00
1.03
1.00
1.04
2.04
1.00
1.00
3.00
3.01
9.07
8.5
8.0
7.5
7.0
6.5
6.0
5.5
5.0
4.5
4.0
3.5
3.0
2.5
2.0
1.5
1.0
0.5
0.0
f1 (ppm)

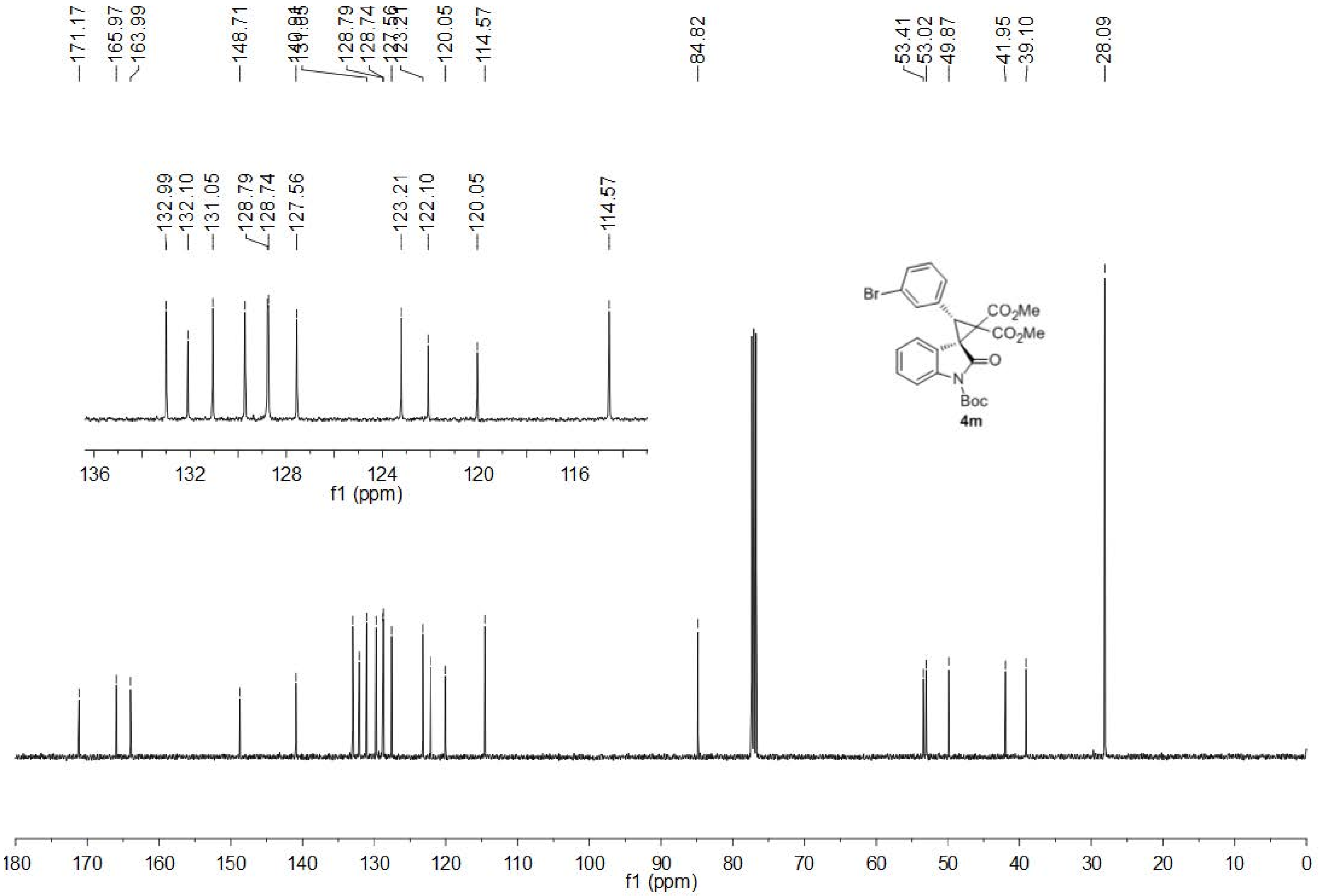
171.17
165.97
163.99
148.71
128.79
128.74
120.05
114.57
84.82
53.41
53.02
49.87
41.95
39.10
28.09
132.99
132.10
131.05
128.79
128.74
127.56
123.21
122.10
120.05
114.57
Br
CO_2Me
CO_2Me
O
N
Boc
4m
136
132
128
124
120
116
f1 (ppm)
180
170
160
150
140
130
120
110
100
90
80
70
60
50
40
30
20
10
0
f1 (ppm)

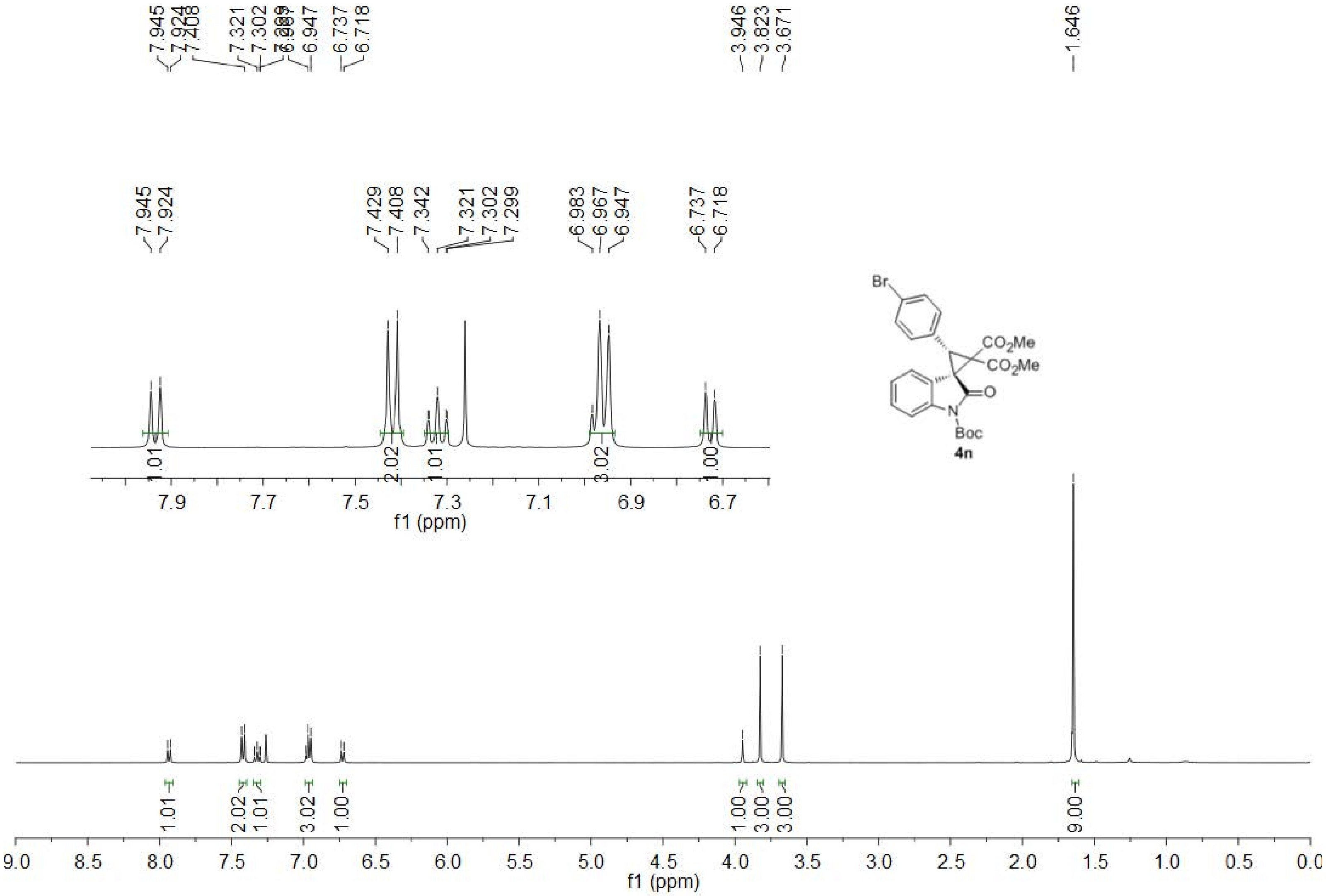

7.945
7.924
7.408
7.321
7.302
6.947
6.737
6.718
3.946
3.823
3.671
1.646
7.945
7.924
7.429
7.408
7.342
7.321
7.302
7.299
6.983
6.967
6.947
6.737
6.718
1.01
2.02
1.01
3.02
1.00
7.9
7.7
7.5
7.3
7.1
6.9
6.7
f1 (ppm)
Br
CO2Me
CO2Me
O
N
Boc
4n
1.01
2.02
1.01
3.02
1.00
1.00
3.00
3.00
9.00
9.0
8.5
8.0
7.5
7.0
6.5
6.0
5.5
5.0
4.5
4.0
3.5
3.0
2.5
2.0
1.5
1.0
0.5
f1 (ppm)

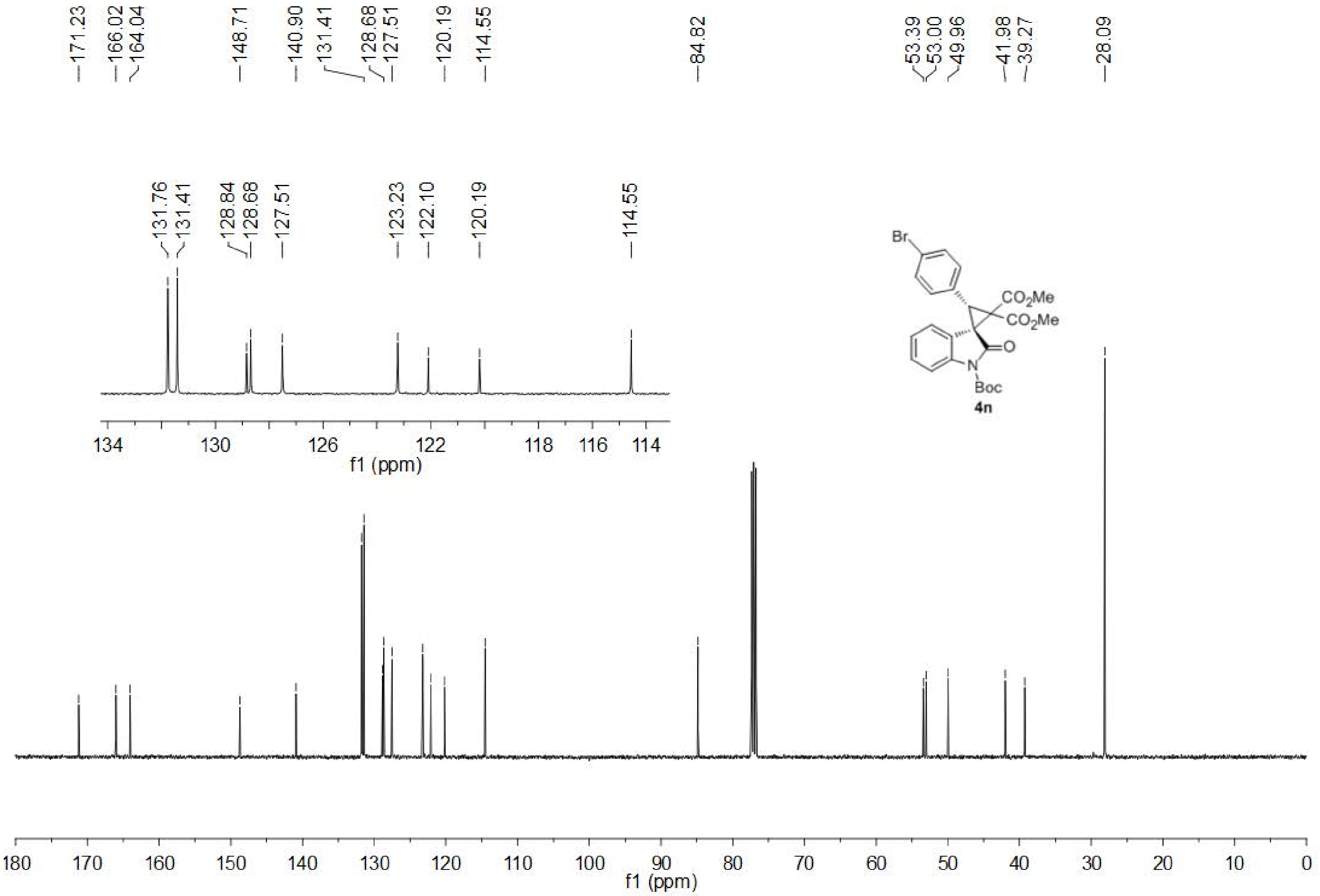

171.23
166.02
164.04
148.71
140.90
131.41
128.68
127.51
120.19
114.55
84.82
53.39
53.00
49.96
41.98
39.27
28.09
131.76
131.41
128.84
128.68
127.51
123.23
122.10
120.19
114.55
134
130
126
122
118
116
114
f1 (ppm)
Br
CO_2Me
CO_2Me
N
O
Boc
4n
180
170
160
150
140
130
120
110
100
90
80
70
60
50
40
30
20
10
0
f1 (ppm)

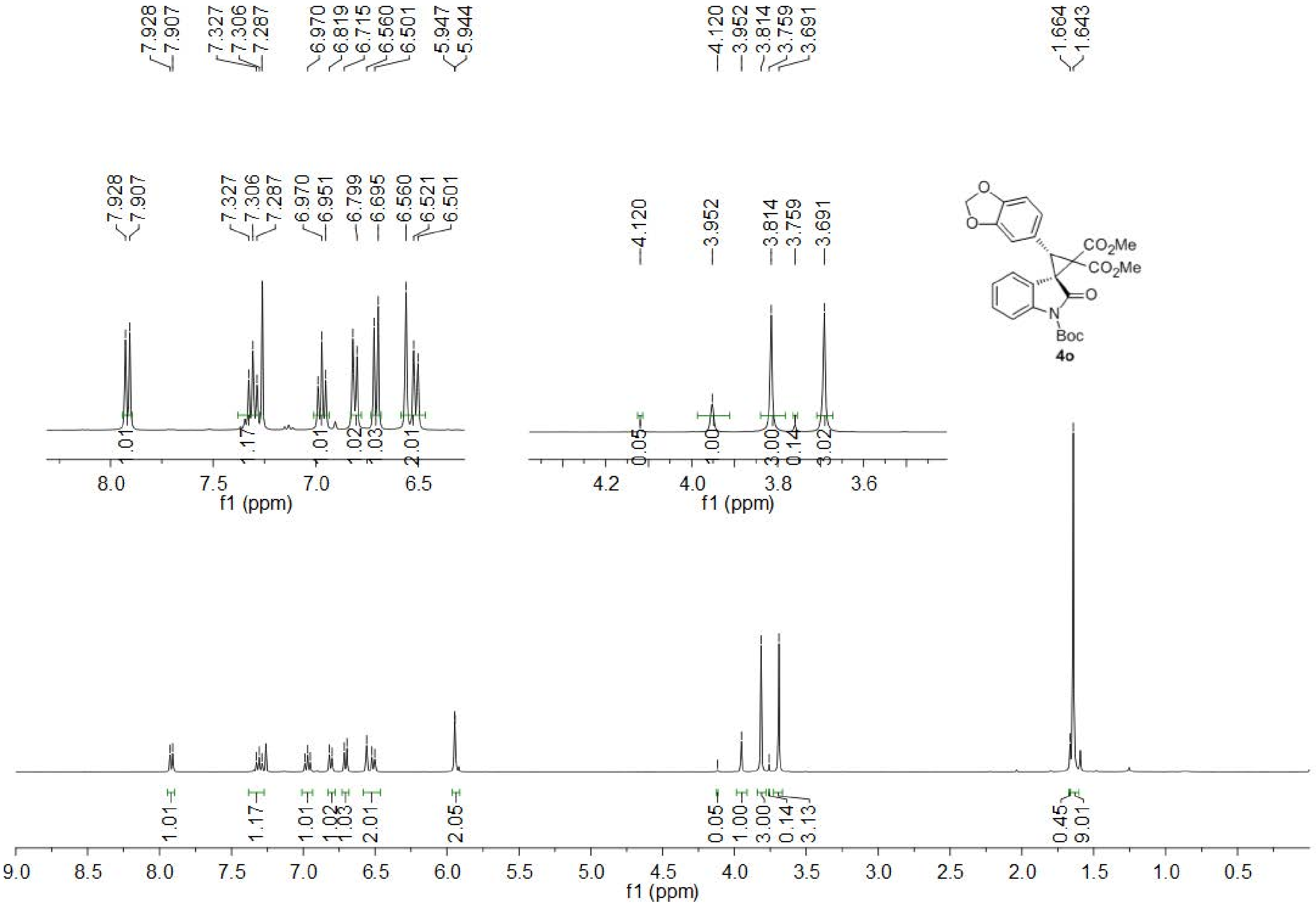

7.928
7.907
7.327
7.306
7.287
6.970
6.819
6.715
6.560
6.501
5.947
5.944
4.120
3.952
3.814
3.759
3.691
1.664
1.643
CO2Me
CO2Me
O
N
Boc
4o
1.01
1.17
1.01
1.02
1.03
2.01
2.05
0.05
1.00
3.00
0.14
3.13
0.45
9.01
f1 (ppm)

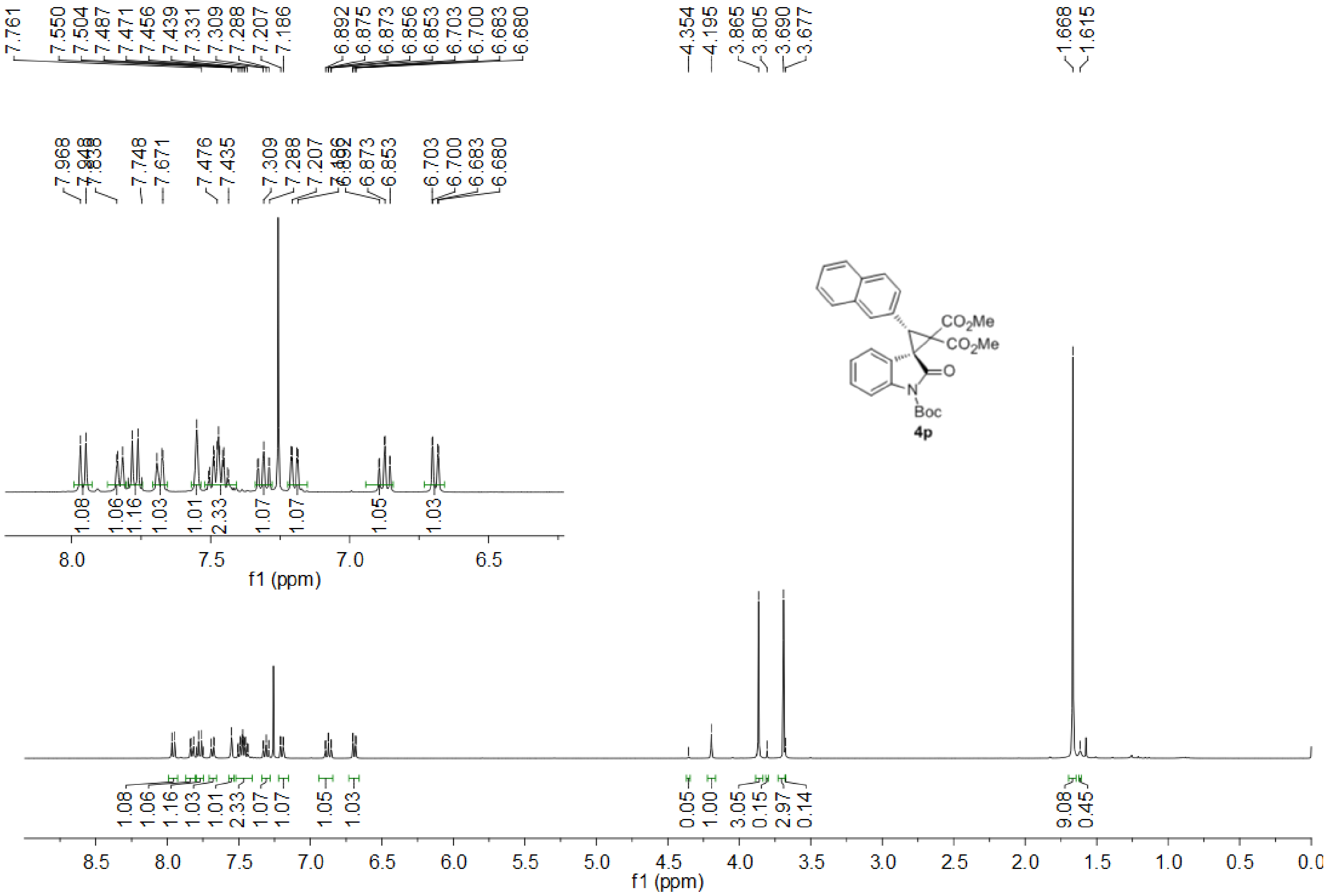

7.761
7.550
7.504
7.487
7.471
7.456
7.439
7.331
7.309
7.288
7.207
7.186
6.892
6.875
6.873
6.856
6.853
6.703
6.700
6.683
6.680
4.354
4.195
3.865
3.805
3.690
3.677
1.668
1.615
7.968
7.748
7.671
7.476
7.435
CO2Me
CO2Me
N
O
Boc
4p
1.08
1.06
1.16
1.03
1.01
2.33
1.07
1.07
1.05
1.03
f1 (ppm)
0.05
1.00
3.05
0.15
2.97
0.14
9.08
0.45
f1 (ppm)

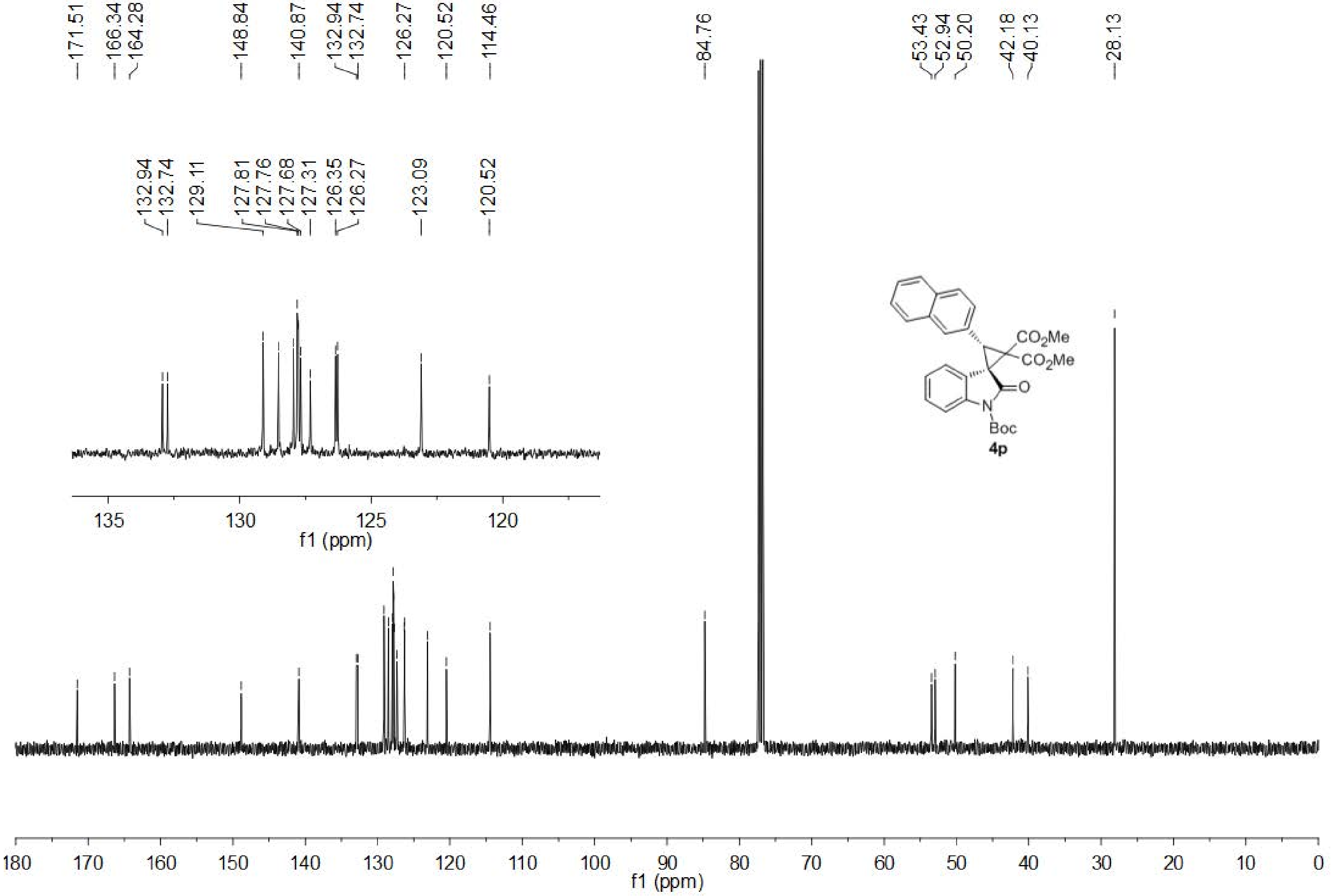

171.51
166.34
164.28
148.84
140.87
132.94
132.74
126.27
120.52
114.46
84.76
53.43
52.94
50.20
42.18
40.13
28.13
132.94
132.74
129.11
127.81
127.76
127.68
127.31
126.35
126.27
123.09
120.52
135
130
125
120
f1 (ppm)
CO2Me
CO2Me
O
N
Boc
4p
180
170
160
150
140
130
120
110
100
90
80
70
60
50
40
30
20
10
0
f1 (ppm)

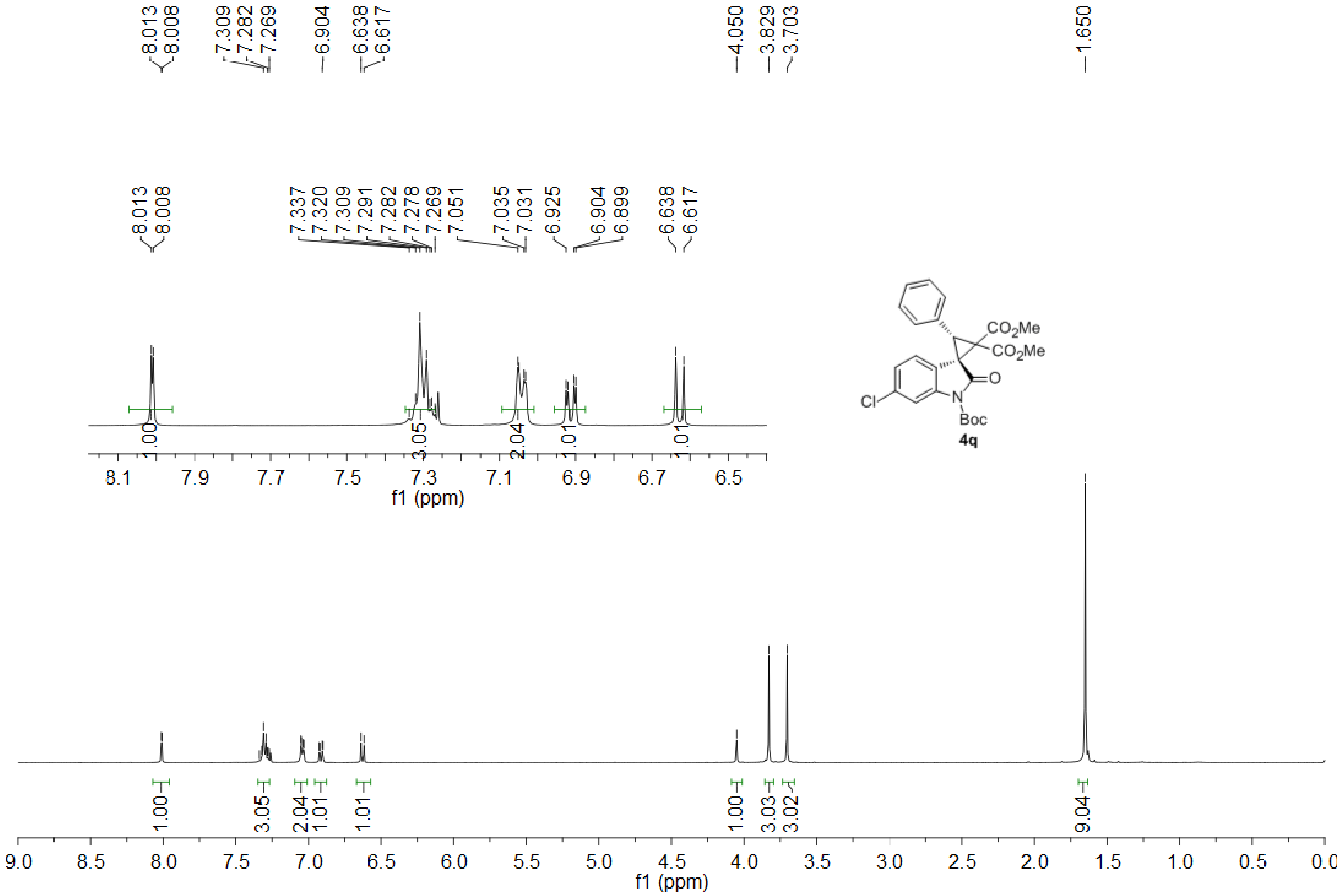
8.013
8.008
7.309
7.282
7.269
6.904
6.638
6.617
4.050
3.829
3.703
1.650
7.337
7.320
7.291
7.278
7.051
7.035
7.031
6.925
6.899
CO2Me
CO2Me
Cl
N
O
Boc
4q
f1 (ppm)

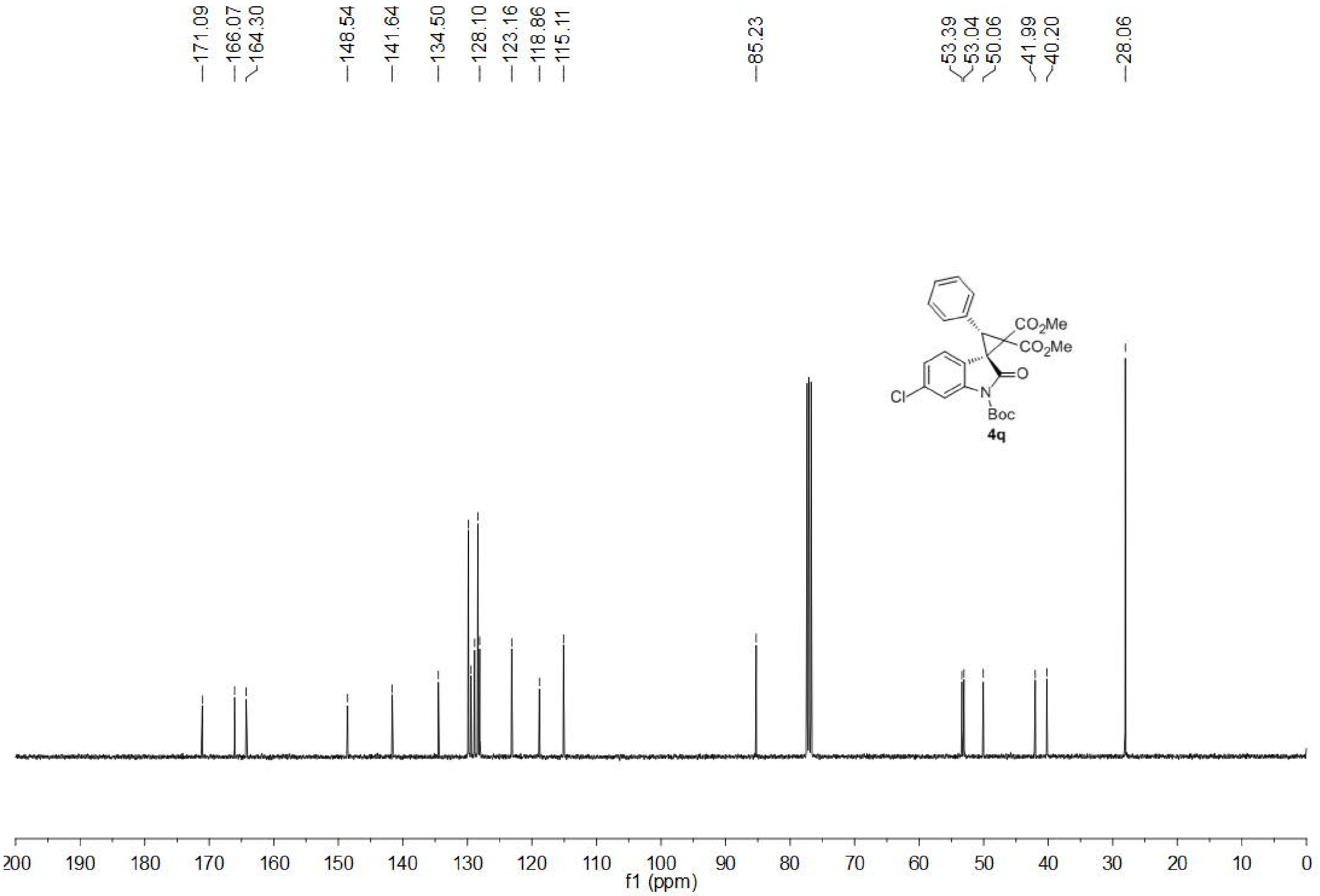

171.09
166.07
164.30
148.54
141.64
134.50
128.10
123.16
118.86
115.11
85.23
53.39
53.04
50.06
41.99
40.20
28.06
CO2Me
CO2Me
O
Cl
N
Boc
4q
200 190 180 170 160 150 140 130 120 110 100 90 80 70 60 50 40 30 20 10 0
f1 (ppm)

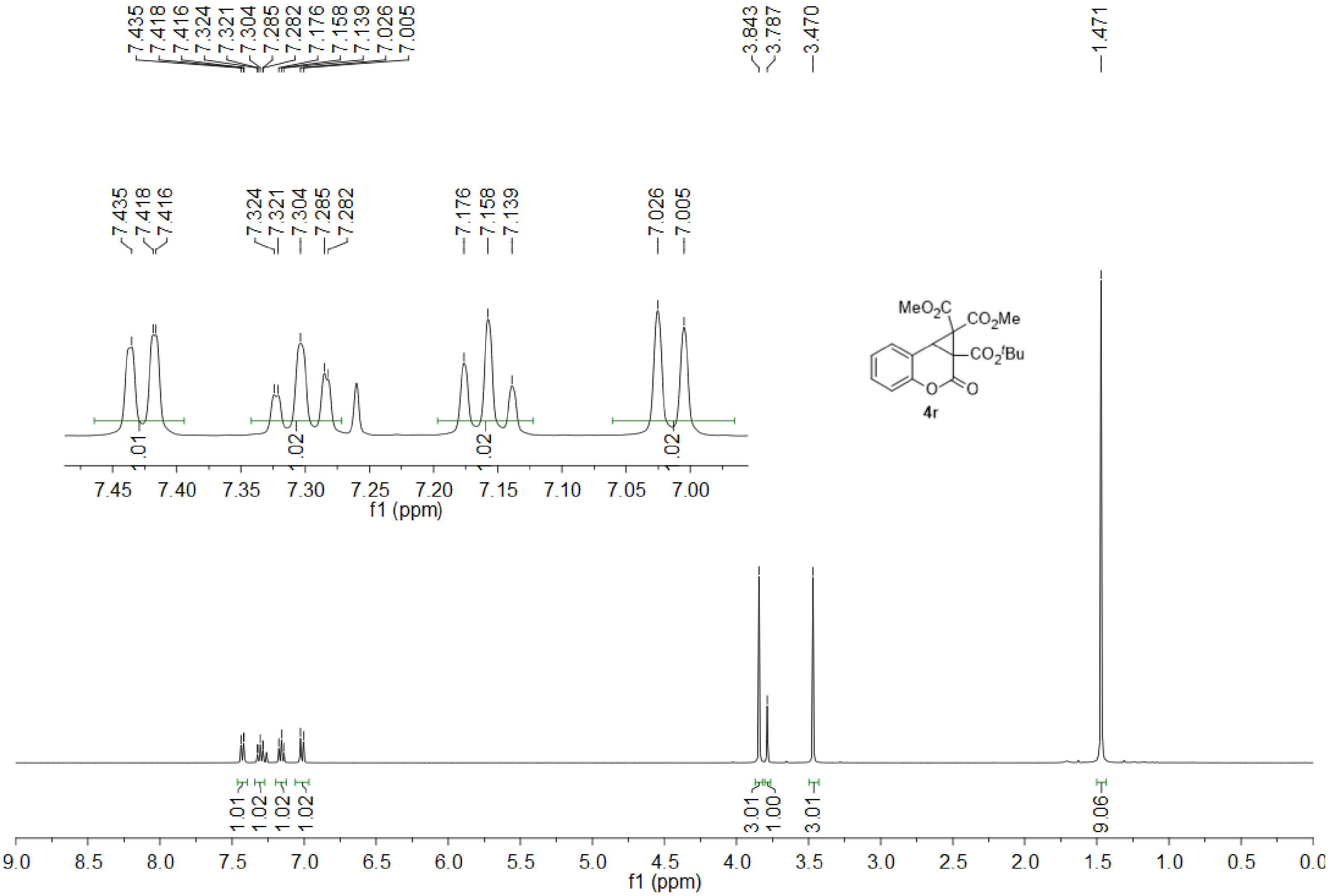
7.435
7.418
7.416
7.324
7.321
7.304
7.285
7.282
7.176
7.158
7.139
7.026
7.005
3.843
3.787
3.470
1.471
MeO2C
CO2Me
CO2tBu
O
O
4r
1.01
1.02
1.02
1.02
3.01
1.00
3.01
9.06
f1 (ppm)

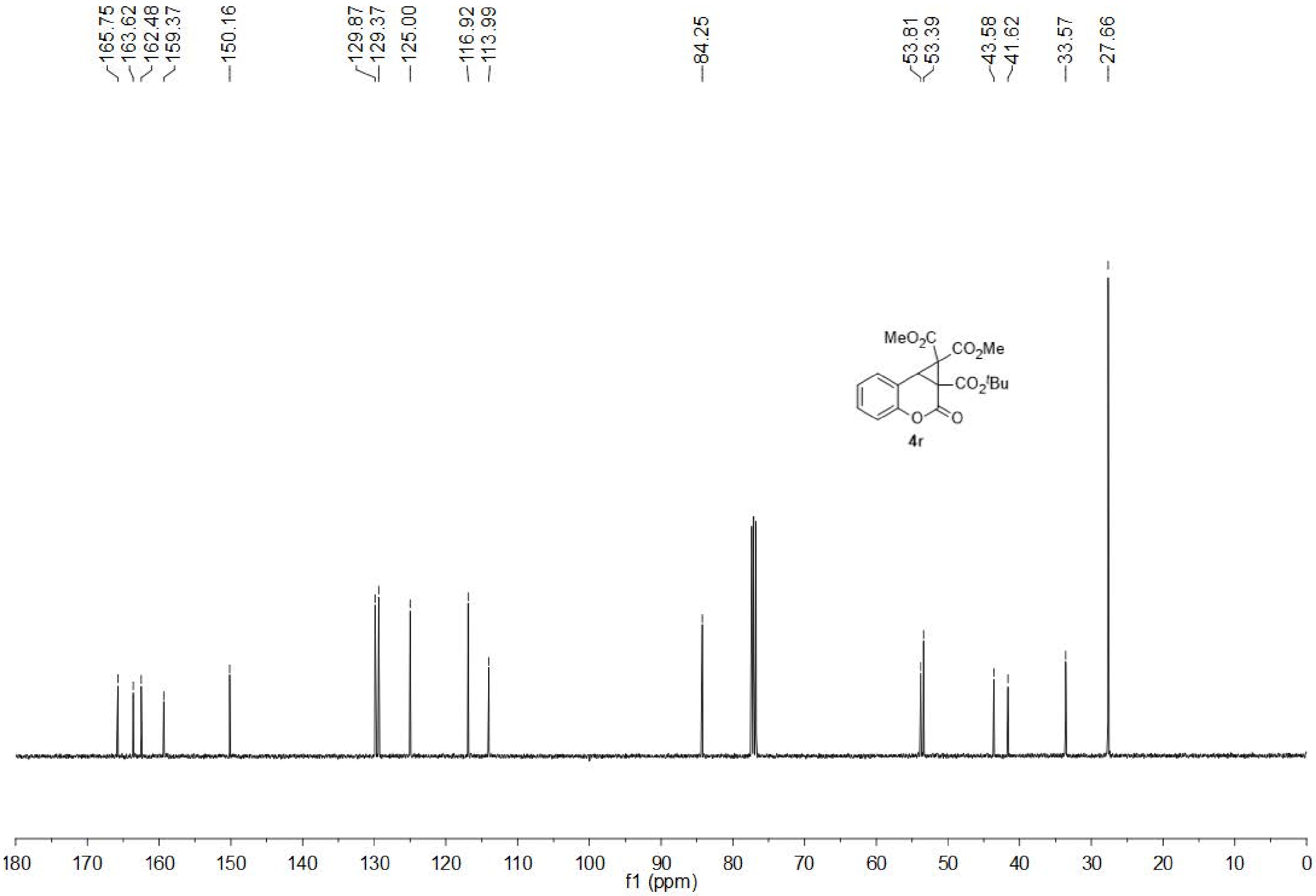

165.75
163.62
162.48
159.37
150.16
129.87
129.37
125.00
116.92
113.99
84.25
53.81
53.39
43.58
41.62
33.57
27.66
MeO2C
CO2Me
CO2tBu
O
O
4r
180
170
160
150
140
130
120
110
100
90
80
70
60
50
40
30
20
10
0
f1 (ppm)

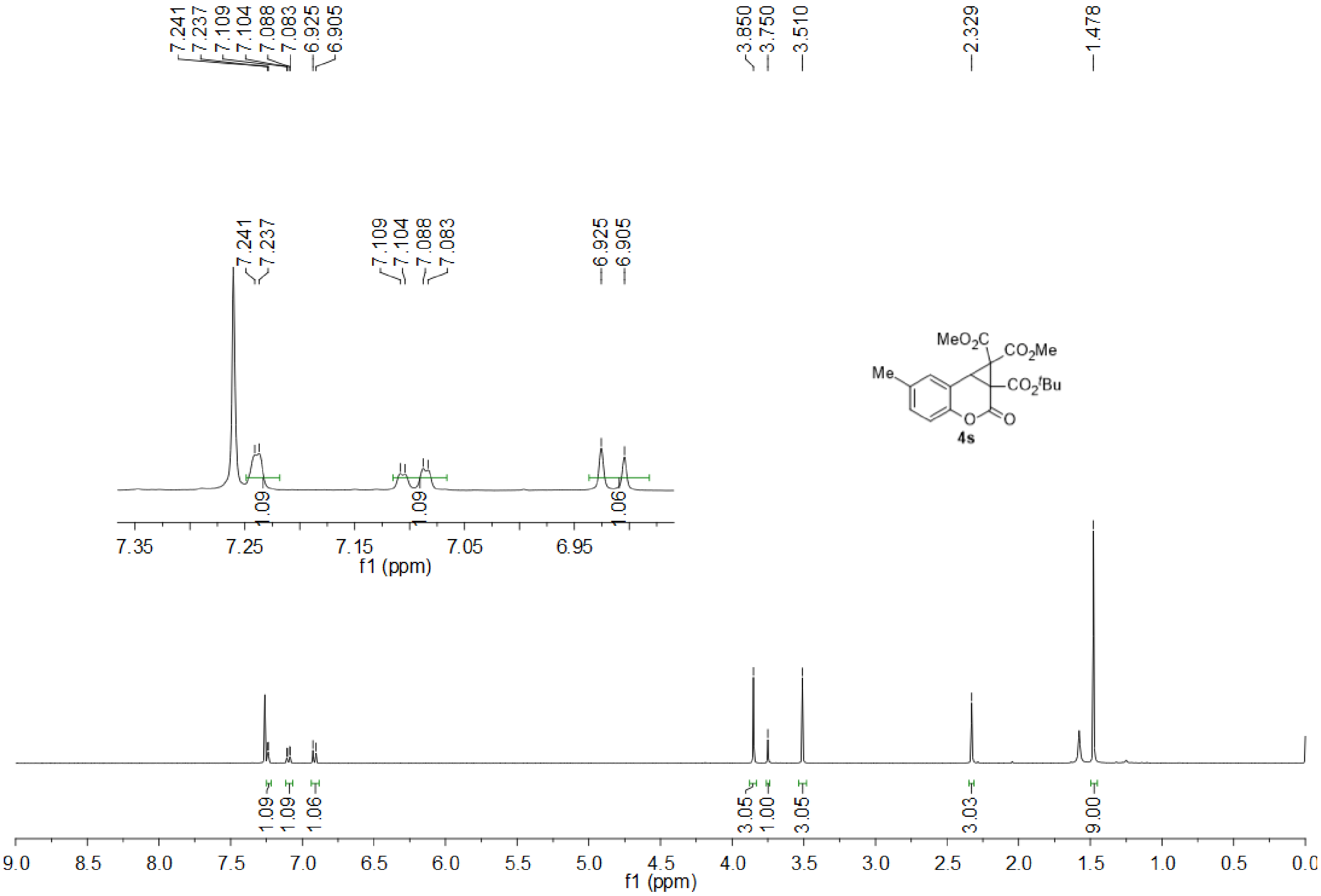

7.241
7.237
7.109
7.104
7.088
7.083
6.925
6.905
3.850
3.750
3.510
2.329
1.478
MeO2C
CO2Me
Me
CO2tBu
O
O
4s
1.09
1.09
1.06
7.35
7.25
7.15
7.05
6.95
f1 (ppm)
1.09
1.09
1.06
3.05
1.00
3.05
3.03
9.00
9.0
8.5
8.0
7.5
7.0
6.5
6.0
5.5
5.0
4.5
4.0
3.5
3.0
2.5
2.0
1.5
1.0
0.5
0.0
f1 (ppm)

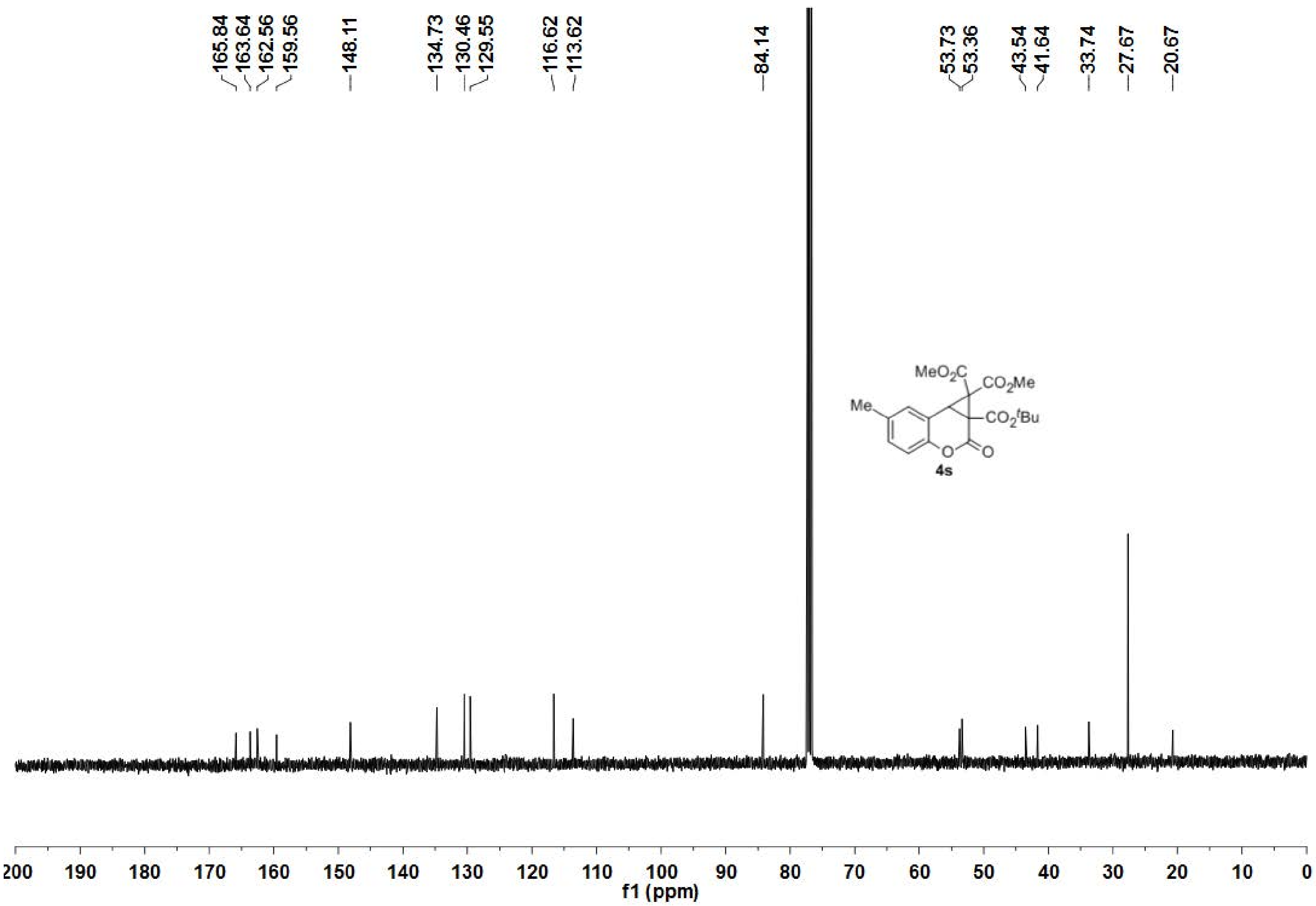
165.84
163.64
162.56
159.56
148.11
134.73
130.46
129.55
116.62
113.62
84.14
53.73
53.36
43.54
41.64
33.74
27.67
20.67
MeO2C
CO2Me
Me
CO2tBu
O
O
4s
200 190 180 170 160 150 140 130 120 110 100 90 80 70 60 50 40 30 20 10 0
f1 (ppm)

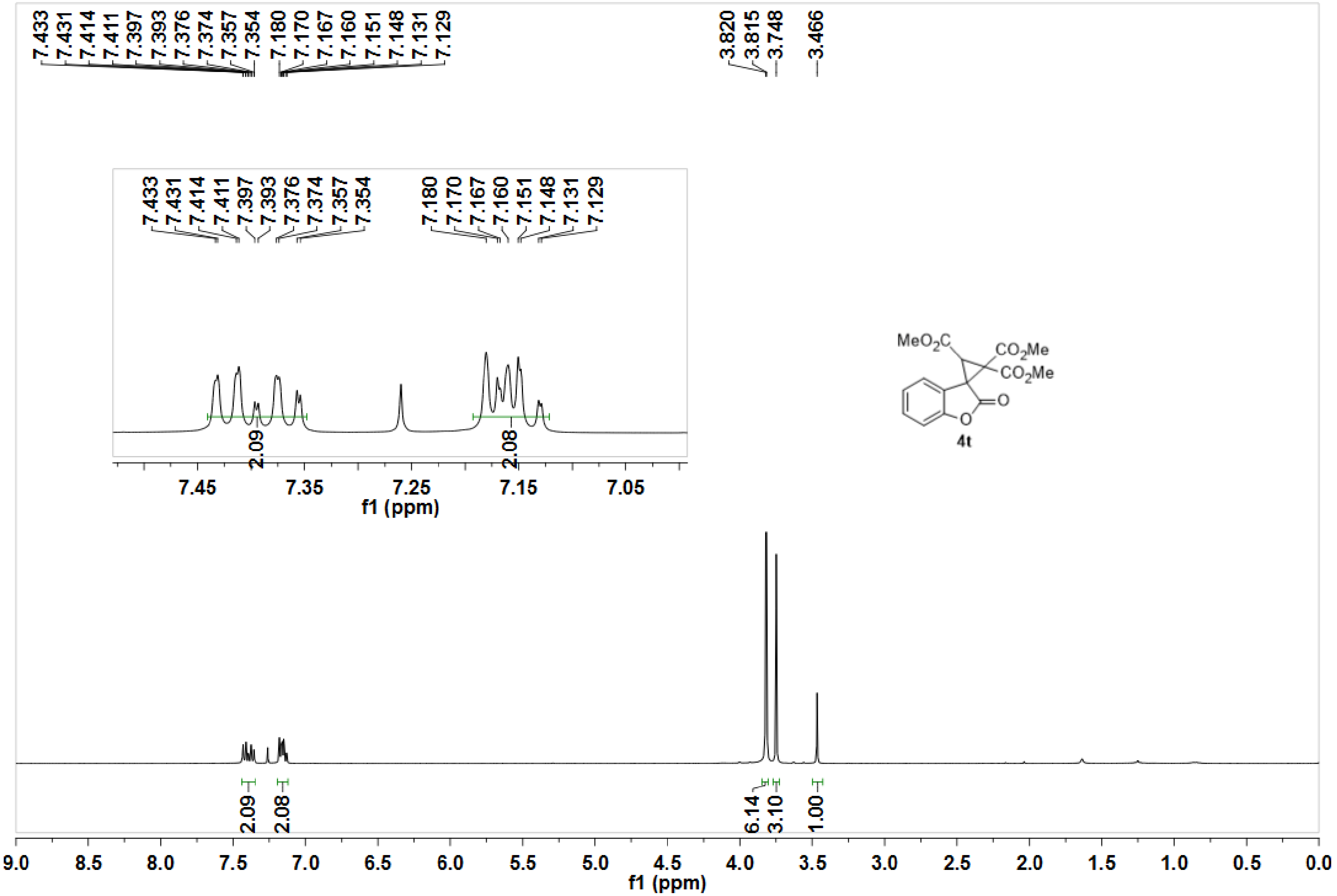
7.433
7.431
7.414
7.411
7.397
7.393
7.376
7.374
7.357
7.354
7.180
7.170
7.167
7.160
7.151
7.148
7.131
7.129
3.820
3.815
3.748
3.466
MeO2C
CO2Me
CO2Me
O
O
4t
2.09
2.08
7.45
7.35
7.25
7.15
7.05
f1 (ppm)
2.09
2.08
6.14
3.10
1.00
9.0
8.5
8.0
7.5
7.0
6.5
6.0
5.5
5.0
4.5
4.0
3.5
3.0
2.5
2.0
1.5
1.0
0.5
0.0
f1 (ppm)

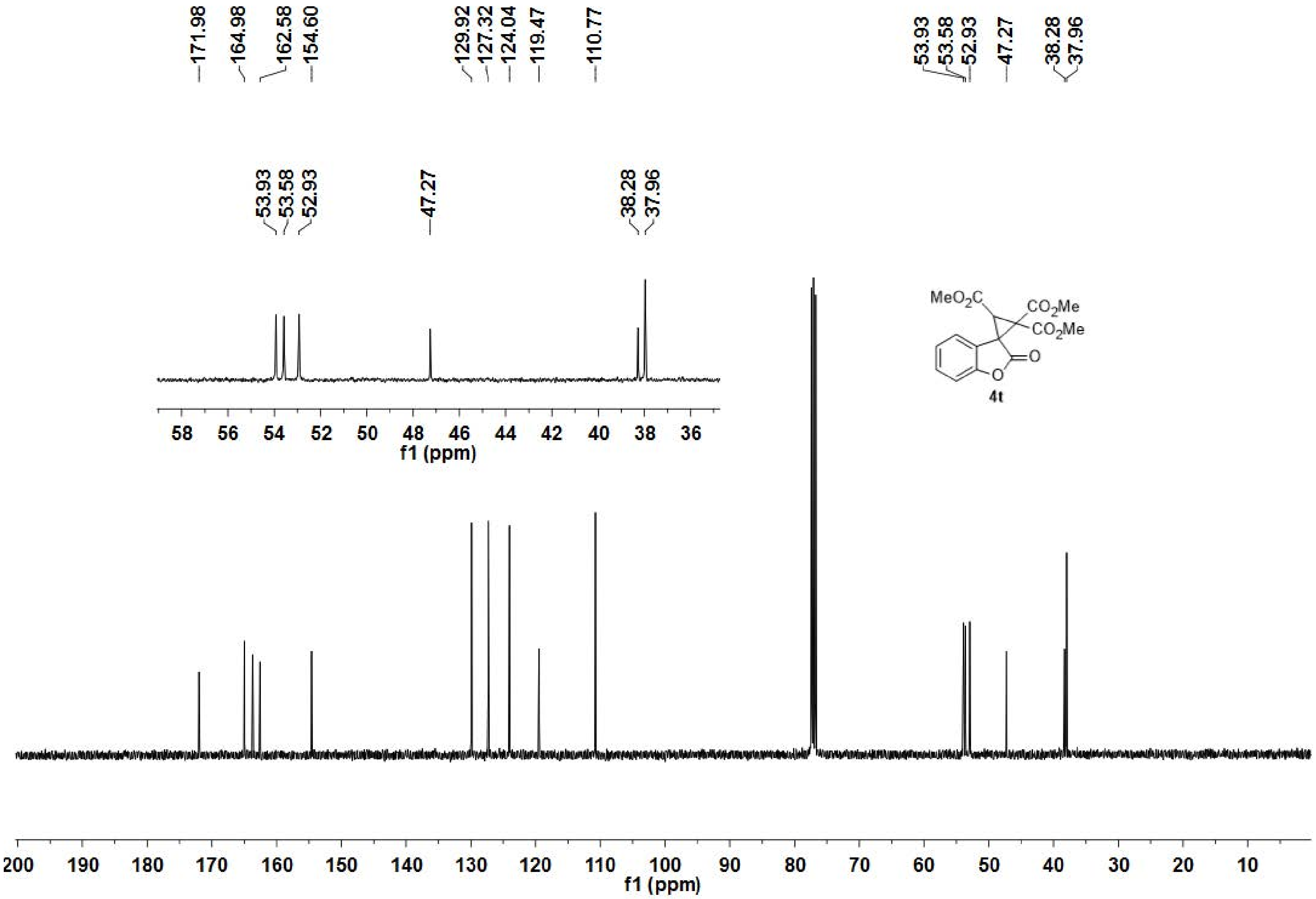

171.98
164.98
162.58
154.60
129.92
127.32
124.04
119.47
110.77
53.93
53.58
52.93
47.27
38.28
37.96
MeO2C
CO2Me
CO2Me
O
O
4t
58 56 54 52 50 48 46 44 42 40 38 36
f1 (ppm)
200 190 180 170 160 150 140 130 120 110 100 90 80 70 60 50 40 30 20 10
f1 (ppm)

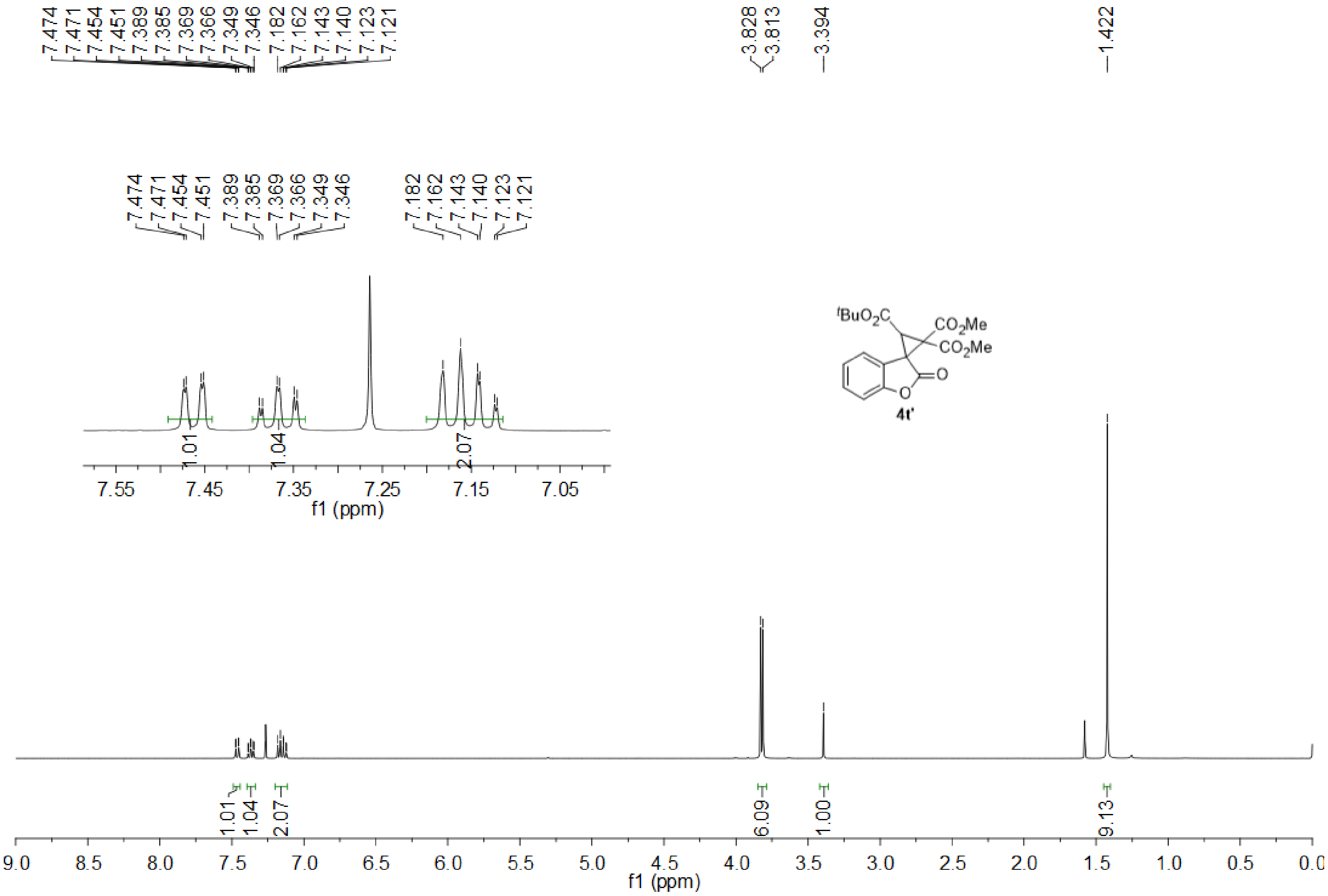

7.474
7.471
7.454
7.451
7.389
7.385
7.369
7.366
7.349
7.346
7.182
7.162
7.143
7.140
7.123
7.121
3.828
3.813
3.394
1.422
1.01
1.04
2.07
7.55
7.45
7.35
7.25
7.15
7.05
f1 (ppm)
ᵗBuO₂C
CO₂Me
CO₂Me
O
O
4t'
6.09
1.00
9.13
9.0
8.5
8.0
7.5
7.0
6.5
6.0
5.5
5.0
4.5
4.0
3.5
3.0
2.5
2.0
1.5
1.0
0.5
0.0
f1 (ppm)

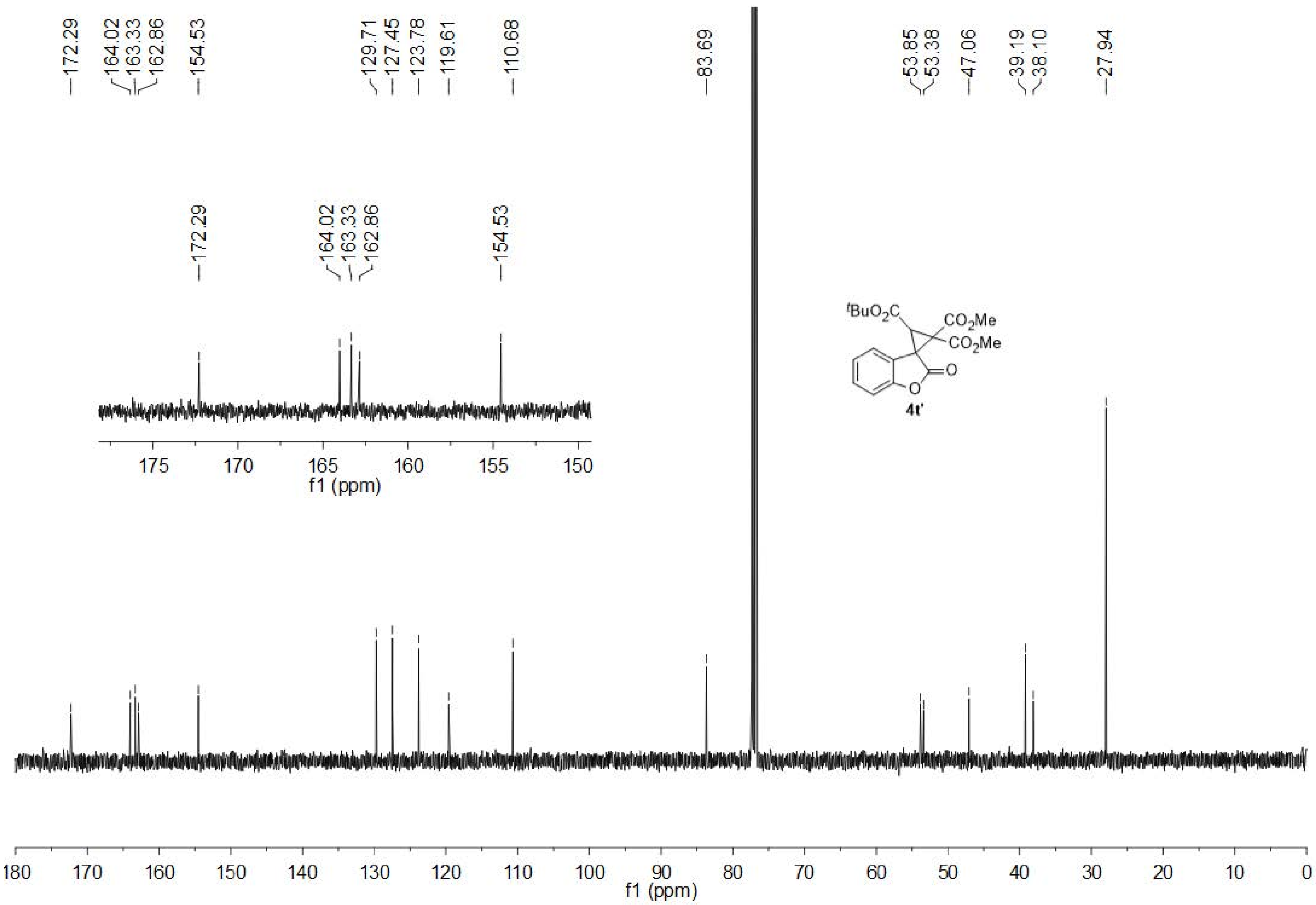
172.29
164.02
163.33
162.86
154.53
129.71
127.45
123.78
119.61
110.68
83.69
53.85
53.38
47.06
39.19
38.10
27.94
tBuO2C
CO2Me
CO2Me
O
O
4t'
175
170
165
160
155
150
f1 (ppm)
180
170
160
150
140
130
120
110
100
90
80
70
60
50
40
30
20
10
0
f1 (ppm)

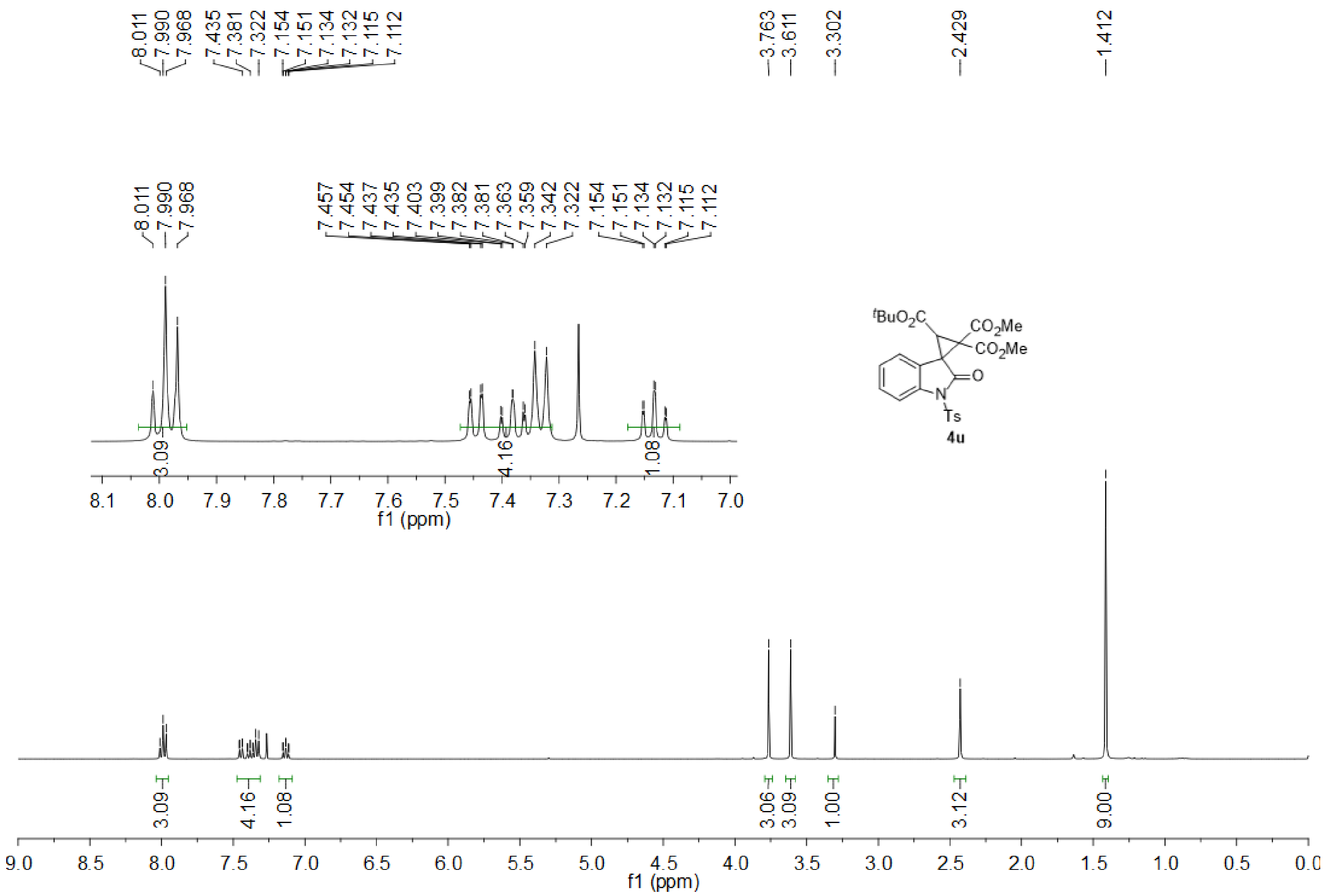

8.011
7.990
7.968
7.435
7.381
7.322
7.154
7.151
7.134
7.132
7.115
7.112
3.763
3.611
3.302
2.429
1.412
7.457
7.454
7.437
7.435
7.403
7.399
7.382
7.381
7.363
7.359
7.342
7.322
3.09
4.16
1.08
3.06
3.09
1.00
3.12
9.00
f1 (ppm)
f1 (ppm)
$^{t}BuO_2C$
CO_2Me
CO_2Me
O
N
Ts
4u

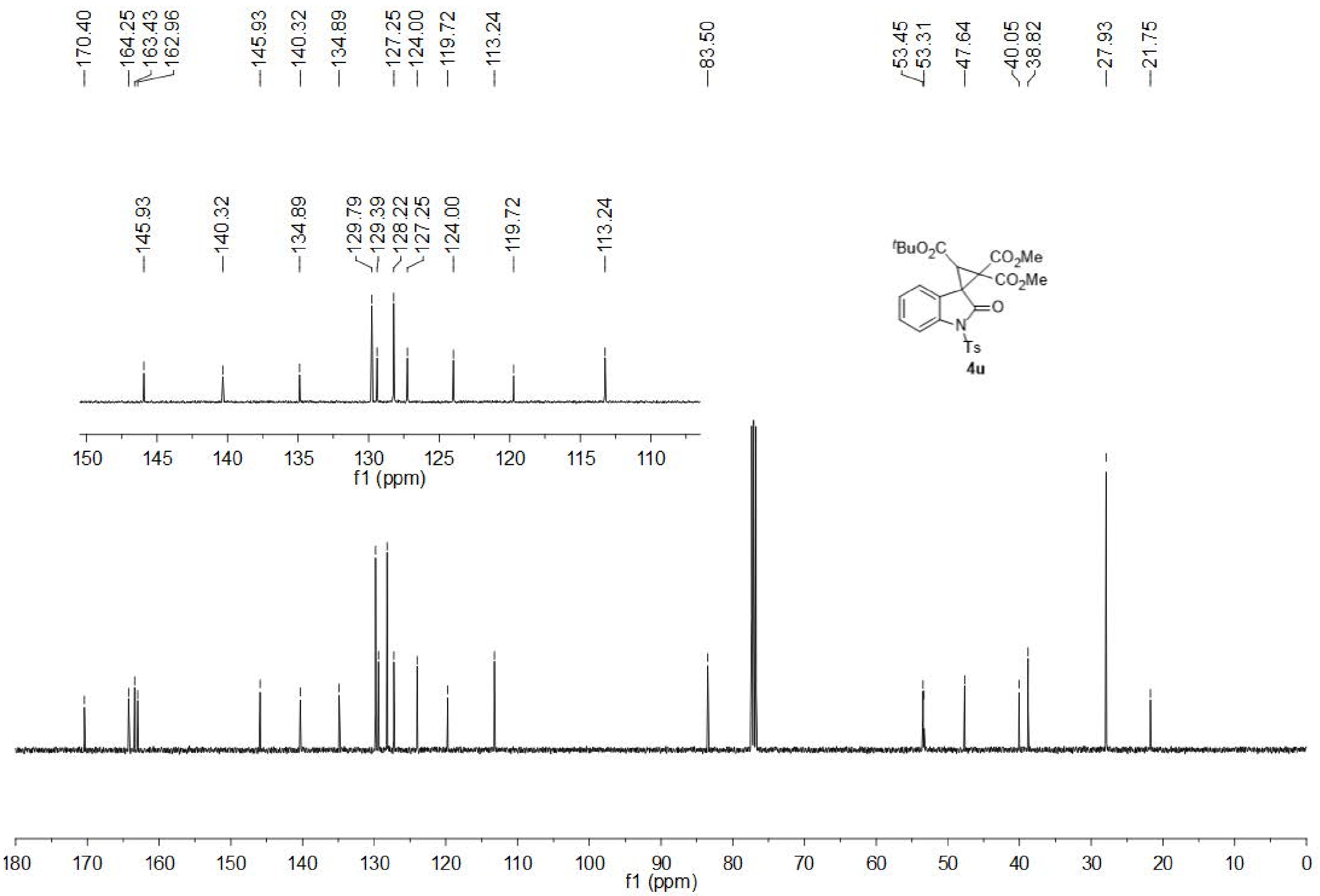

170.40
164.25
163.43
162.96
145.93
140.32
134.89
127.25
124.00
119.72
113.24
83.50
53.45
53.31
47.64
40.05
38.82
27.93
21.75
145.93
140.32
134.89
129.79
129.39
128.22
127.25
124.00
119.72
113.24
150 145 140 135 130 125 120 115 110
f1 (ppm)
tBuO2C
CO2Me
CO2Me
O
N
Ts
4u
180 170 160 150 140 130 120 110 100 90 80 70 60 50 40 30 20 10 0
f1 (ppm)

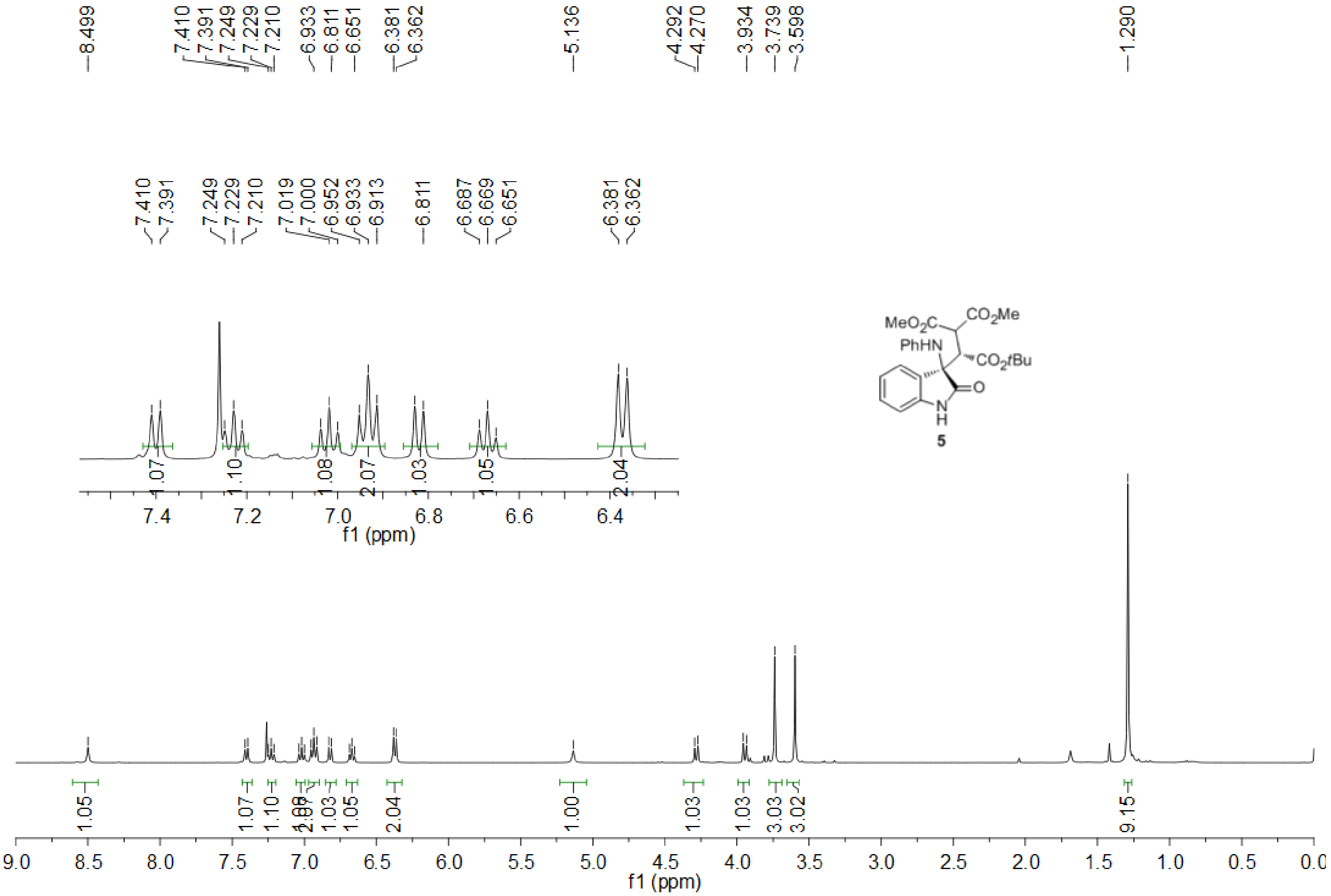

8.499
7.410
7.391
7.249
7.229
7.210
6.933
6.811
6.651
6.381
6.362
5.136
4.292
4.270
3.934
3.739
3.598
1.290
7.019
7.000
6.952
6.913
6.687
6.669
MeO2C
CO2Me
PhHN
CO2tBu
O
N
H
5
f1 (ppm)

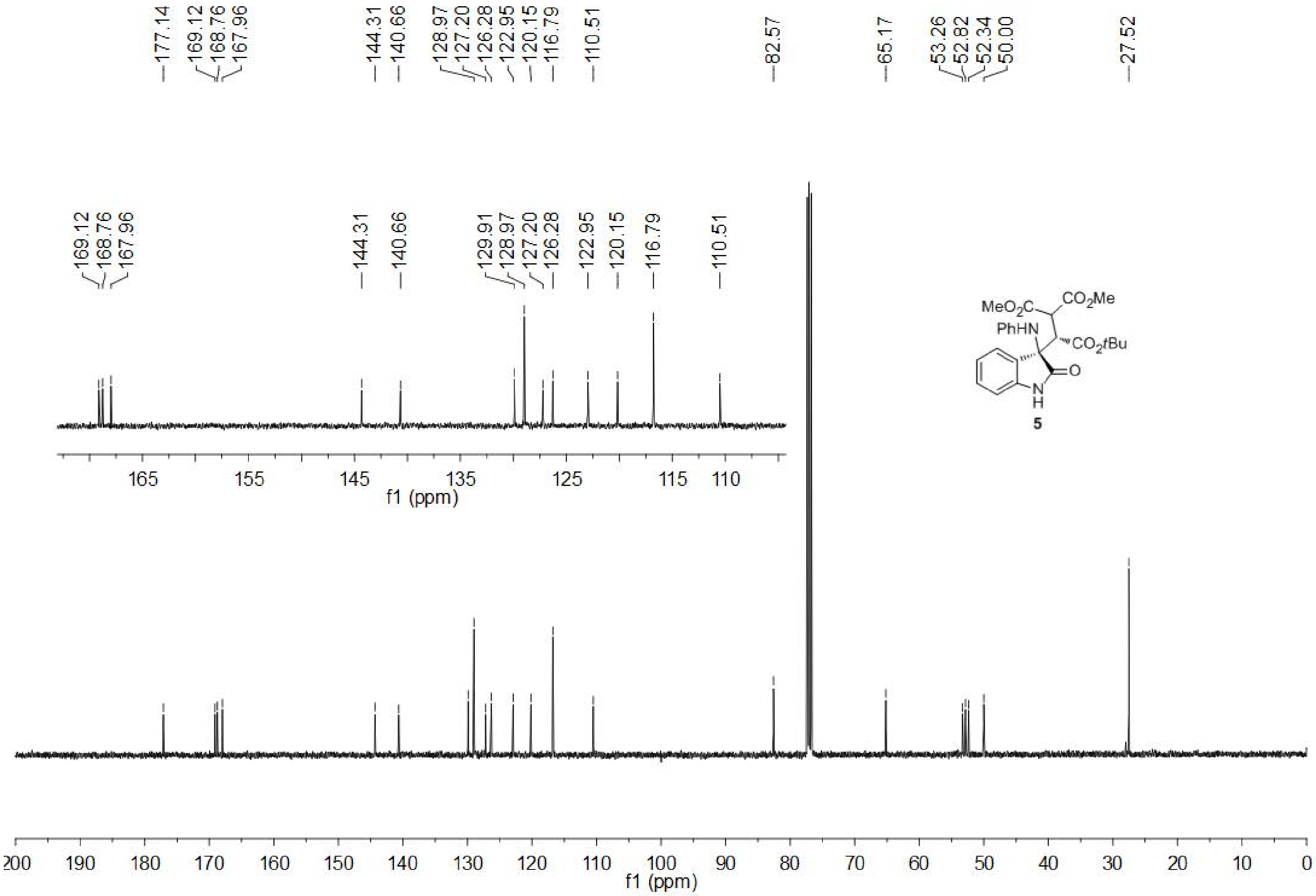

177.14
169.12
168.76
167.96
144.31
140.66
128.97
127.20
126.28
122.95
120.15
116.79
110.51
82.57
65.17
53.26
52.82
52.34
50.00
27.52
169.12
168.76
167.96
144.31
140.66
129.91
128.97
127.20
126.28
122.95
120.15
116.79
110.51
165
155
145
135
125
115
110
f1 (ppm)
MeO2C
CO2Me
PhHN
CO2tBu
O
N
H
5
200
190
180
170
160
150
140
130
120
110
100
90
80
70
60
50
40
30
20
10
0
f1 (ppm)

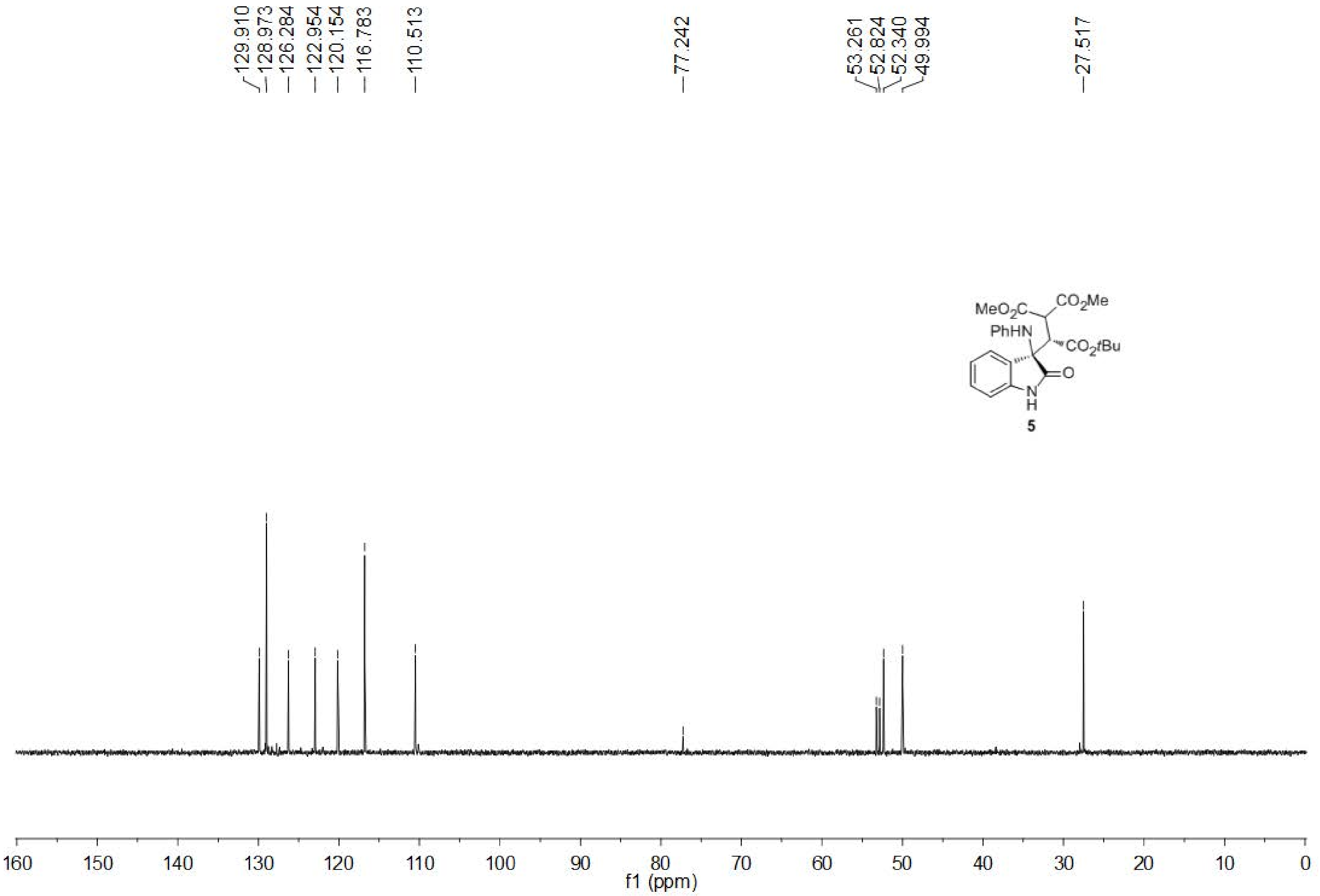

129.910
128.973
126.284
122.954
120.154
116.783
110.513
77.242
53.261
52.824
52.340
49.994
27.517
MeO2C
CO2Me
PhHN
CO2tBu
O
N
H
5
160
150
140
130
120
110
100
90
80
70
60
50
40
30
20
10
0
f1 (ppm)
